# A highly site-selective radical sp^3^ C–H amination of azaheterocycles†
†Electronic supplementary information (ESI) available. See DOI: 10.1039/c8sc00590g


**DOI:** 10.1039/c8sc00590g

**Published:** 2018-07-10

**Authors:** Keith W. Bentley, Krysta A. Dummit, Jeffrey F. Van Humbeck

**Affiliations:** a Department of Chemistry , Massachusetts Institute of Technology , Cambridge MA 02139 , USA; b Department of Chemistry , University of Calgary , Calgary , AB T2N 1N4 , Canada . Email: jeffrey.vanhumbec1@ucalgary.ca

## Abstract

This report describes the development of a novel C–H amination strategy using both a Cu(ii) Lewis acid and an organic hydrogen atom transfer catalyst to activate benzylic C–H bonds adjacent to aromatic N-heterocycles. This simple methodology demonstrates very high selectivity towards azaheterocycles without using exogenous directing groups and affords excellent site selectivity in substrates with more than one reactive position. A wide range of heterocyclic structures not compatible with previously reported catalytic systems have proven to be amenable to this approach. Mechanistic investigations strongly support a radical-mediated H-atom abstraction, which explains the observed contrast to known closed-shell Lewis acid catalyzed processes.

## Abstract 2

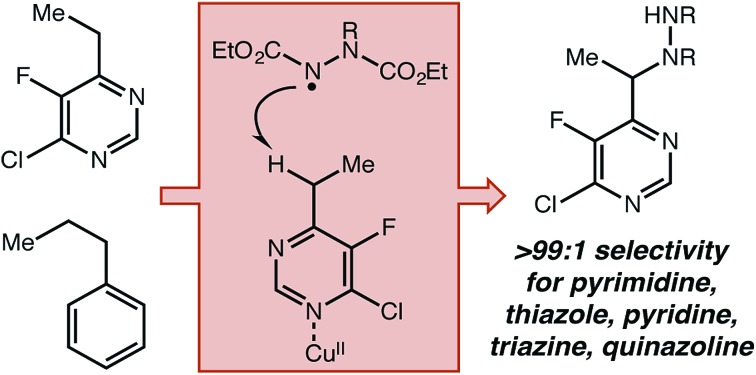
This report describes the development of a novel C–H amination strategy using both a Cu(ii) Lewis acid and an organic hydrogen atom transfer catalyst to activate benzylic C–H bonds adjacent to aromatic N-heterocycles.

## Introduction

**Fig. 1:**
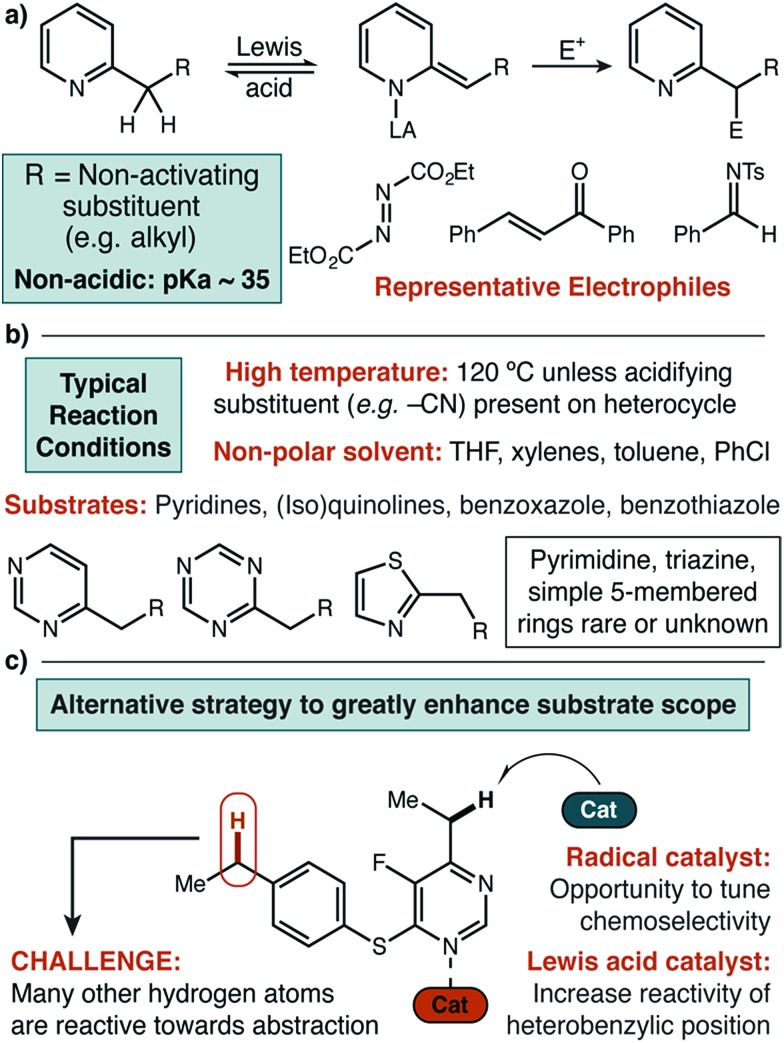
(a) Current Lewis acid catalyzed approaches to direct functionalization of heterocycles using a closed-shell mechanism. (b) Limitations of current approach with respect to reaction conditions and substrate scope. (c) Design plan for the development of a dual catalytic strategy to significantly broaden substrate scope.

Nitrogen-containing heterocycles are pervasive in natural products,^1^ functional materials,^2^ agrochemicals,^3^ and small molecule pharmaceuticals.^4^ In particular, aromatic azaheterocycles are of special importance for medicinal chemistry, being found in nearly 25% of all FDA approved small molecule drugs. These diverse structures include both 5- and 6-membered rings and can feature one or several nitrogen atoms per ring.^5^ The resulting breadth of electronic character has made the development of universal methodologies for the functionalization of entire classes of azaheterocycles very challenging.^6^ Benzylic sp^3^ C–H functionalization of azaheterocycles is particularly important, with various substitution patterns found in this position in biologically relevant small molecules.^7^ Herein we report a radical abstraction strategy that is selective for the benzylic position of a broad range of azaheterocycles, even in the presence of other C–H bonds of similar bond dissociation energy (Fig. 1c).

Formation of sp^3^ C–N bonds *via* radical intermediates is an active field of research,^8^ with several complementary approaches emerging to supplement well-established systems such as those based on rhodium. First row transition metal nitrenes based on iron, manganese, cobalt, copper, and nickel have been studied fundamentally and to deliver new synthetic methods.8a–f Fully organic sequences that leverage C–H abstraction/cyclization pathways have also been recently reported.8*h* Despite the increasing number of parallel strategies, substrates that feature aromatic azaheterocycles are rarely reported.

Previously, more common approaches to benzylic functionalization for heterocycles relied on Lewis acid-promoted ionic mechanisms. These pathways successfully yield new C–C, C–O and C–N bonds (Fig. 1a).^9^ Specifically in the case of copper, the Huang and Guo groups used diethyl azodicarboxylate (DEAD) for pyridine amination, where the mechanism was proposed to involve a dearomatized nucleophile that forges the new C–N bond by a two electron pathway.9h,9i Similarly, the Rueping group developed a Cu(ii) system for C–C bond formation that was amenable to several classes of heterocycles.9*g* In general, for all types of catalysts relatively forcing conditions involving high reaction temperatures and non-polar solvents have been required unless acidifying substituents are present.9*k* It was our view that highly desirable polar and functionalized molecules may not have sufficient solubility or stability under such conditions. Further, while extended aromatics (*e.g.* quinoline, benzothiazole) and pyridine-type substrates are widely reported in the Lewis acid catalyzed protocols, simple five-membered rings and highly substituted or electron-withdrawn pyrimidines and triazines are rarely reported or unknown.

To our knowledge, no broadly successful strategy for the activation of these more difficult heterocycles has been reported. Therefore, we set out to design a new approach to the benzylic functionalization of a wide variety of azaheterocycles in a polar solvent at a modest temperature. Focusing on radical-based mechanisms, we envisioned using the demonstrated ability of transition metals to modify both the thermodynamic strength and homolytic reactivity of C–H, C–C, and X–H bonds in substrates coordinated to the metal center.^10^ Ideally, the resulting increase in reactivity would be limited to positions in strong electronic communication with the coordinated metal. High site-selectivity in substrates with other abstractable C–H bonds could potentially follow. We further believed that site selectivity could be additionally enhanced by including (and optimizing) an organic hydrogen atom transfer (HAT) catalyst.^11^ We recently demonstrated that iron tris(pyrazole)borate catalysts could be used to direct methylene oxidation towards heterobenzylic positions in combination with an organic HAT catalyst.^12^

## Results and discussion

### Catalyst system discovery

Underpinning our own work, the Inoue group reported the use of *N*-hydroxypthalimide (NHPI, **1**) as a HAT catalyst for benzylic C–N bond formation in alkyl benzenes using DEAD as the nitrogen source.^13^ However, the conditions developed for relatively electron-rich benzenes, unsurprisingly, are not particularly effective for electron-poor heterocycles (*vide infra*). As a starting point, we selected two examples of difficult substrates (a thiazole and an electron-deficient pyrimidine) for initial investigation. In addition to NHPI, two other commercially available organic small molecules with similar X–H bond strengths^14^ were chosen as potential HAT catalysts—1-hydroxy-7-azabenzotriazole (HOAt, **2**) and diethyl hydrazinedicarboxylate (H_2_DEAD, **3**). A starting selection of five metal triflate catalysts (Ni(ii), Zn(ii), Fe(ii), Cu(ii), Sc(iii)) was used to create the combinatorial set of fifteen catalyst pairs.

**Scheme 1:**
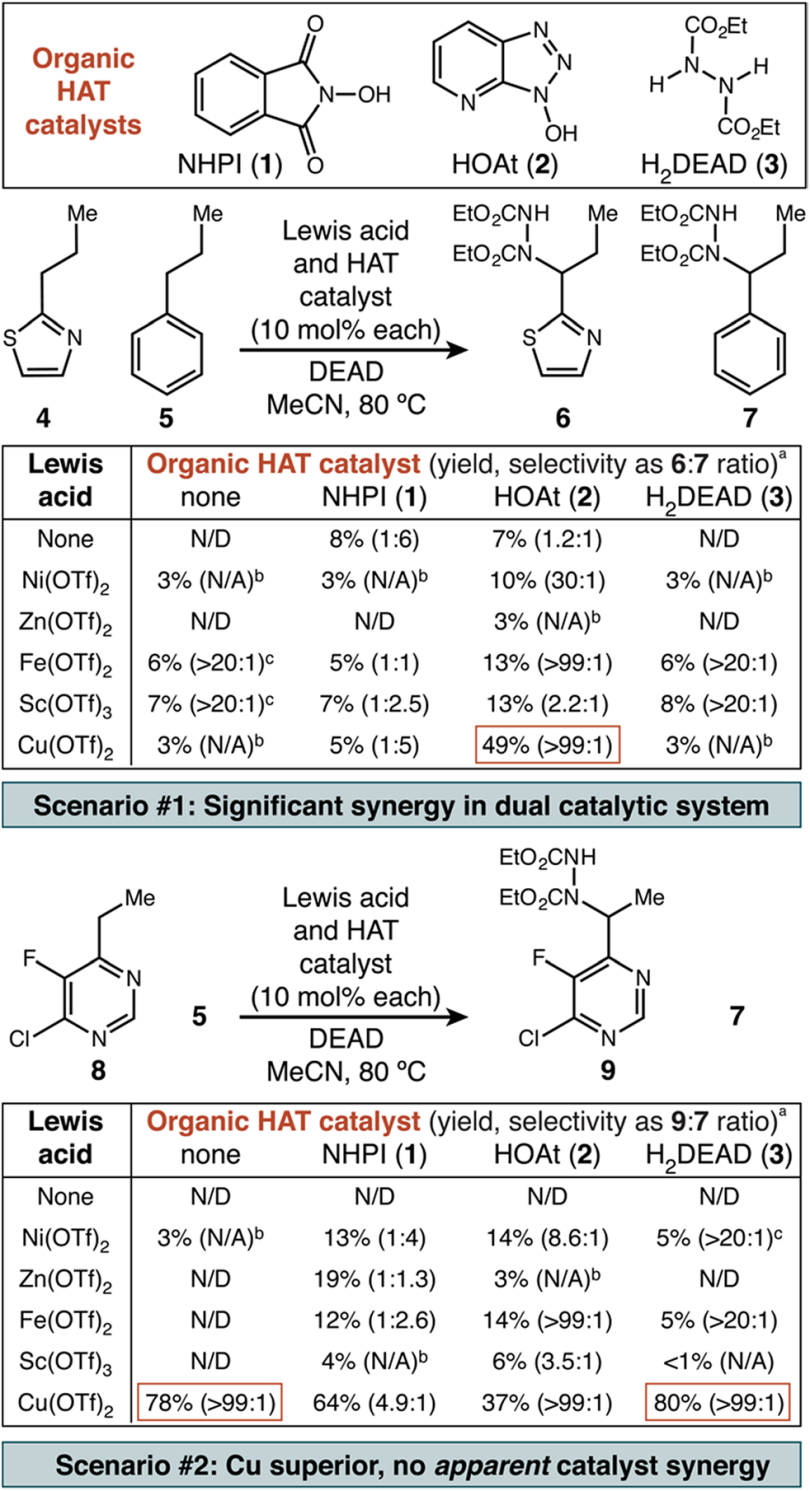
Effect of Lewis acid and organic catalyst structure on C–H amination of challenging substrates. N/D = not detected. ^a^Determined by direct GC analysis of crude reaction mixture. ^b^Selectivity was not estimated on reactions with <5% yield. ^c^For reactions with <10% yield, the maximum observable site selectivity was conservatively limited to >20 : 1.

The first set of experiments used 2-propylthiazole **4** as the heterocycle and propylbenzene **5** as a competitive substrate to give an initial assessment of potential site selectivity (Scheme 1, scenario #1). The difficult nature of this heterocycle type was immediately apparent. When no Lewis acid was added, the organic HAT catalysts on their own were not able to deliver more than 8% desired product (**6**) and even those small amounts were contaminated with significant amounts of the undesired functionalized benzene product (**7**). Similarly, in the absence of a HAT catalyst the Lewis acids alone were not able to deliver more than 7% desired product, though site selectivity did seem to be high. In fourteen of the fifteen catalyst pairs, little improvement was observed. Excitingly, however, a distinct synergistic effect was seen between Cu(OTf)_2_ and HOAt, where a significant enhancement in chemical yield was observed (49%), with exceptional selectivity for functionalization of thiazole in preference to benzene (>99 : 1).

Similarly encouraging results were obtained in the case of pyrimidine (Scheme 1, scenario #2). While organic HAT catalysts in the absence of Lewis acid were wholly unreactive, we found excellent reactivity was uniquely possible with Cu(OTf)_2_. Surprisingly, in this case, no significant synergistic effect was observed, which would seemingly imply that a simple Lewis acid mechanism was still at play. Mechanistic studies described below strongly suggest that this is not the case, and that a radical pathway is actually operative with thiazole **4** and pyrimidine **8** and allows for the success of both of these difficult substrates (*vide infra*).

### Substrate scope – intermolecular competition experiments

**Scheme 2:**
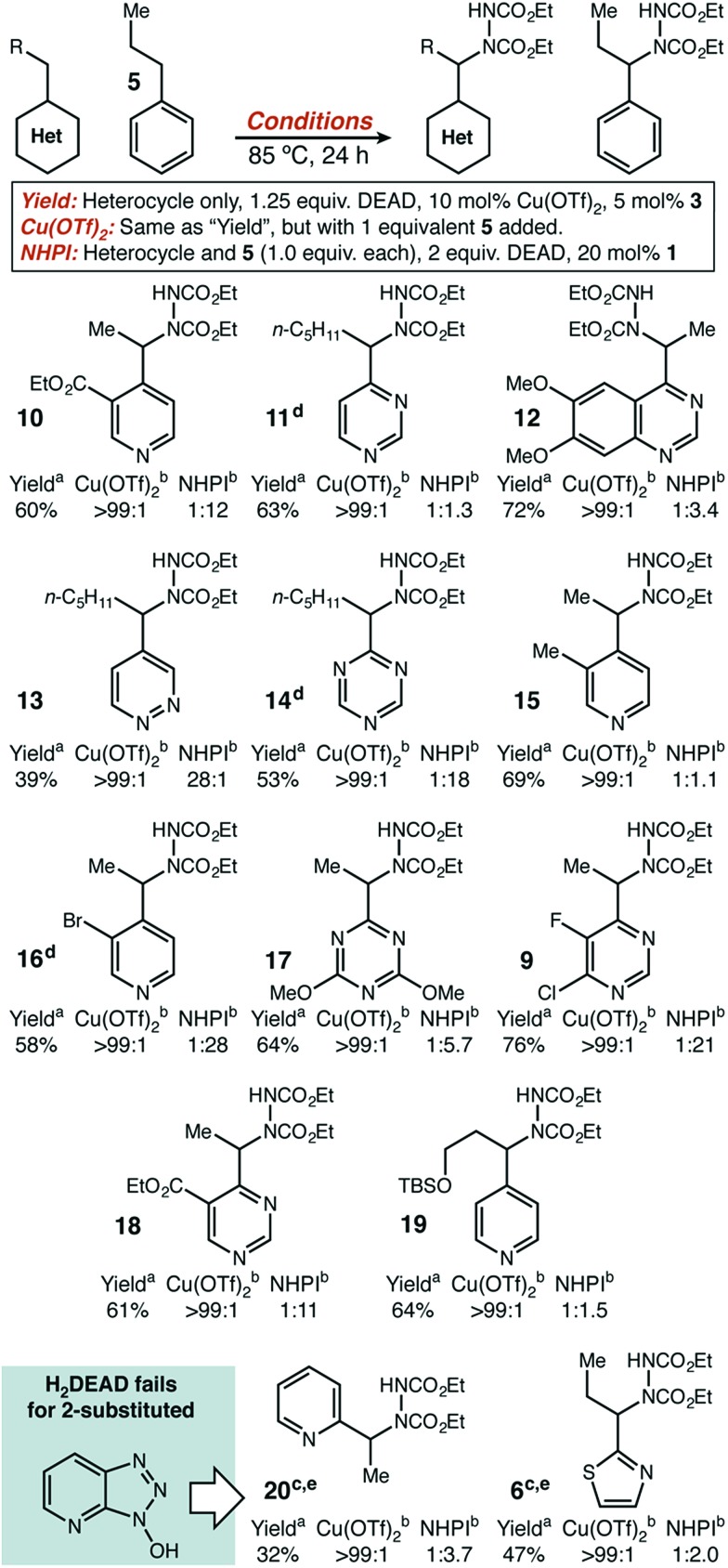
Diversity of compatible heterocycles and level of site selectivity as judged from competition with propylbenzene. ^a^Isolated yield. ^b^Determined by direct GC analysis of crude reaction mixture. ^c^HOAt (10 mol%) used in place of H_2_DEAD. ^d^2 equivalents DEAD used. ^e^1 equivalent DEAD used.

**Fig. 2:**
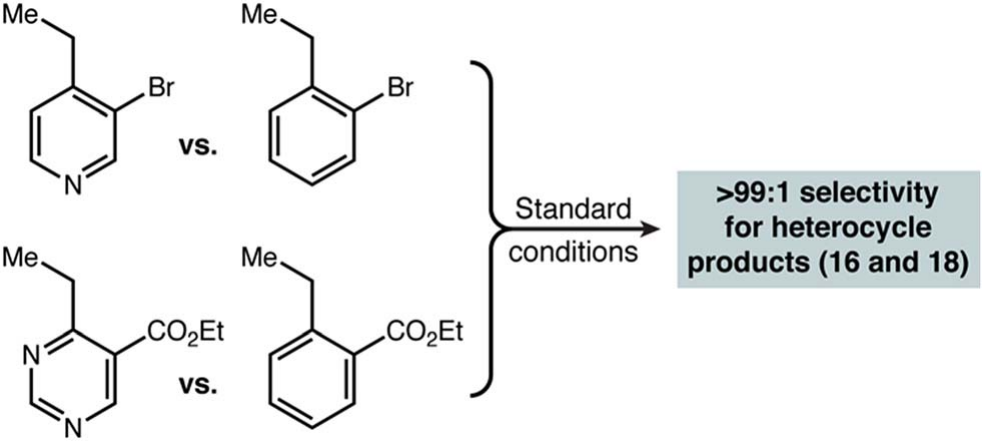
Additional competition experiments that support the validity of using propylbenzene as a substrate for intermolecular competition experiments.

With initial results that suggested Cu(ii)/HAT catalysis could be a broadly applicable technology for radical amination, we next determined the scope of heterocycles compatible (Scheme 2). Under our reaction conditions, amination was successful for many classes of substrates, including mono-, di-, and tri-azines. Good functional group tolerance was observed, with the exception of substituents that can strongly chelate Cu(ii) (*e.g.* 2-acetamido) or have nucleophilic groups that react directly with DEAD (*e.g.* free amines, active methylene groups^15^). Products derived from 2-alkyl substituted heterocycles, such as **6** and **20** could only be delivered with HOAt (**2**) as an organic co-catalyst. In all cases, we used direct competition with **5** to give an initial indication of site selectivity. No significant formation of **7** was observed in any case, universally giving >99 : 1 selectivity for amination at the heterocycle. We also compared our developed conditions to the established radical mechanism reported by Inoue (Scheme 2, “NHPI” conditions). In agreement with our initial screens, catalytic Cu(OTf)_2_ dramatically changes chemoselectivity, driving reaction adjacent to the heterocycle with an orders-of-magnitude change in selectivity. To confirm that these high levels of selectivity were not due to the other functional groups present in the azaheterocycle substrates, we competed a sample pyridine and pyrimidine substrate against its all-carbon aromatic analogue and observed identical selectivity (Fig. 2). In many cases shown in Scheme 2, the yields of the reaction are modest. In general, purification was simple. The main reaction byproducts appeared to be highly colored—and likely over-oxidized—species that were not mobile on silica gel.

### Substrate scope – intramolecular competition experiments

**Scheme 3:**
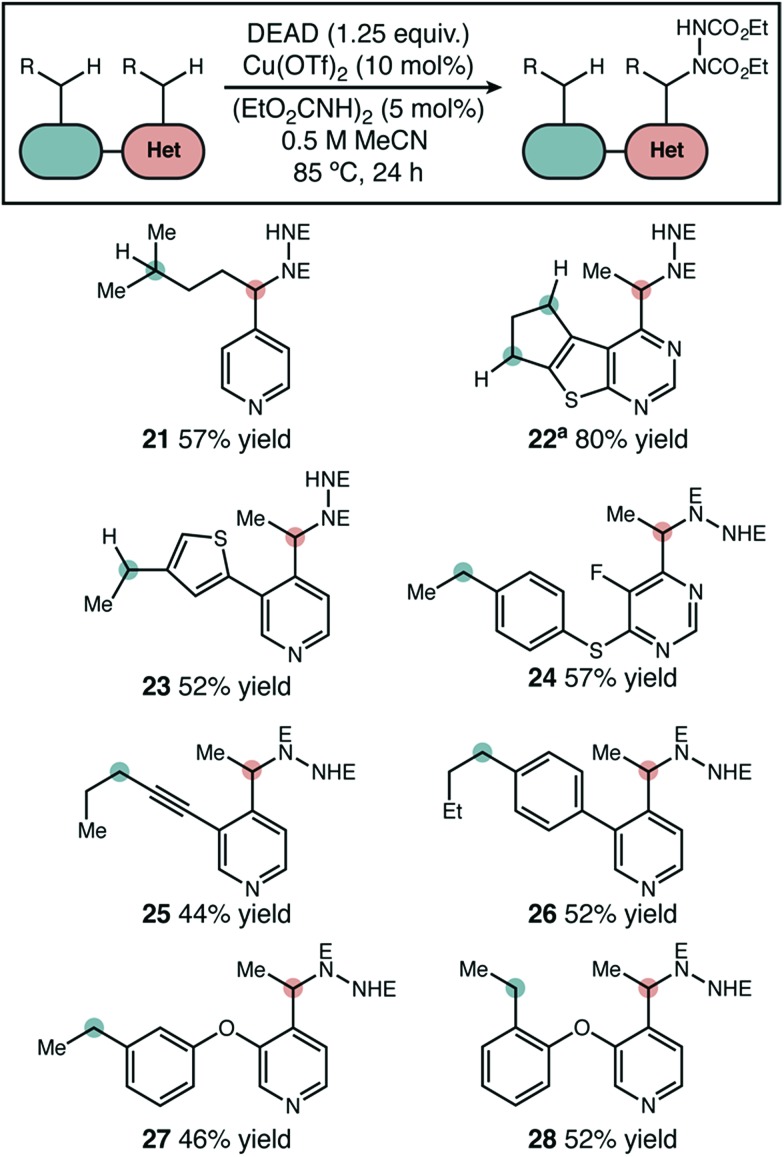
Site-selective amination of azaheterocycles. The products shown were the only regioisomers isolated and yields shown are isolated yields. “E” = CO_2_Et. ^a^1 equivalent of DEAD used.

In substrates with multiple reactive positions, the high level of selectivity delivered by our protocol has an important benefit. In some cases, separation of regioisomeric products may be difficult or impossible, so the complete suppression of undesired HAT is key. A set of representative substrates featuring at least two positions known to be reactive in radical amination was used to demonstrate this explicitly (Scheme 3). Reaction adjacent to the aza-heterocycle was observed as the sole regioisomer in every product isolated. Other heterocycles, simple benzylic positions, tertiary, and propargylic positions proved to be unreactive. The observed yields and selectivities broadly reflect the range seen for our intermolecular competition studies, suggesting that a site-selective reaction should be possible adjacent to many heterocycle types.

### Mechanistic studies

In order to better understand what separated substrates that showed significant synergistic effects with the organic HAT catalyst (*e.g.* thiazole **4**) from those that did not (*e.g.* pyrimidine **8**), we launched a number of mechanistic investigations. As one component of these investigations, we wanted to compare the behavior of our newly developed conditions with a system that operated along the traditional ionic mechanistic pathway. Given that none of the other Lewis acids we investigated were significantly reactive towards thiazole **4** or pyrimidine **8**, we investigated 4-propylpyridine as a less challenging substrate. In that case, we found that Sc(OTf)_3_ was highly reactive in the same solvent and temperature system as our copper-based protocol (MeCN, 80–85 °C) and it was selected for mechanistic comparison (see ESI†).

**Fig. 3:**
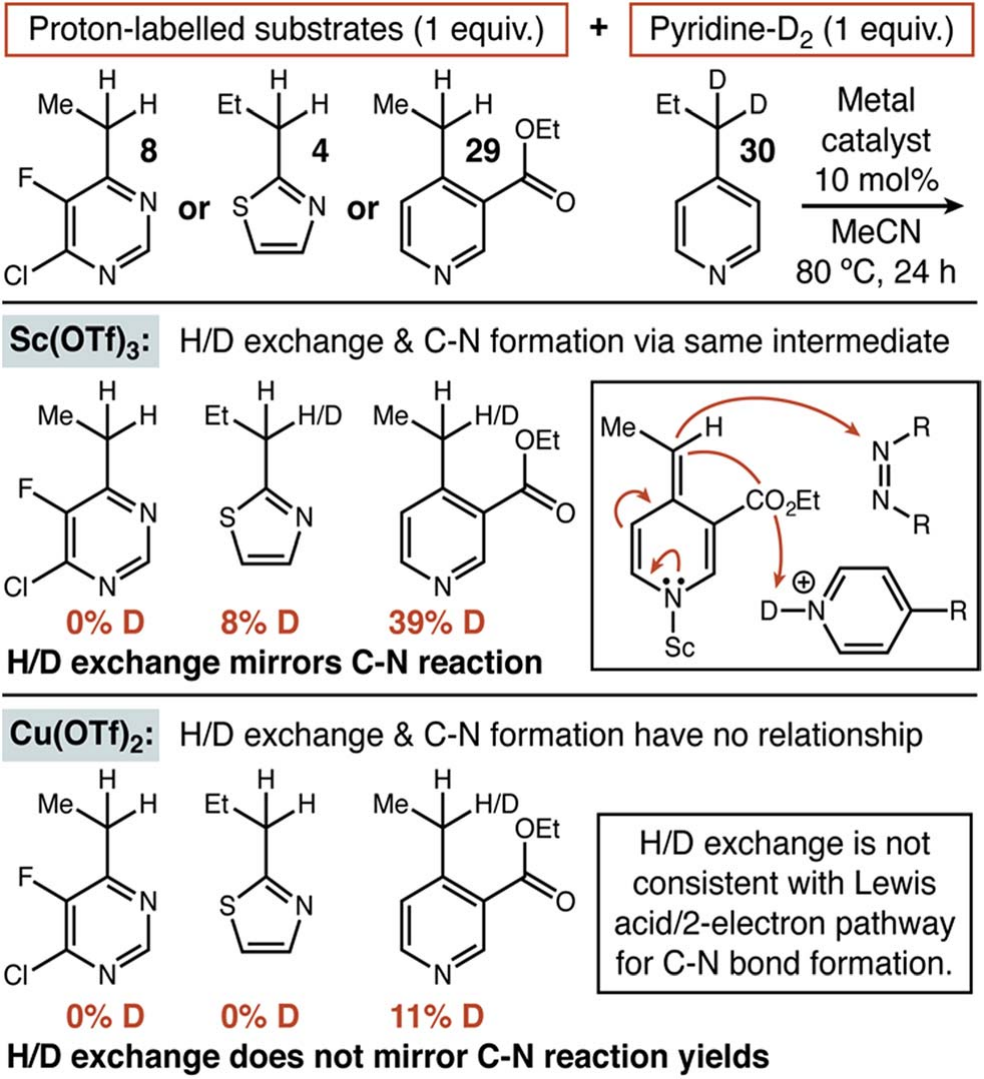
Heterocycle isotope exchange catalyzed by Sc(OTf)_3_ and Cu(OTf)_2_.

Under the established ionic pathway, a Lewis acid–pyridine complex is deprotonated and dearomatized to yield a metal-bound nucleophile as shown in Fig. 1. So, in this mechanistic regime, a mixture of two heterocycles—with one deuterium labelled at the benzylic position—should be able to exchange proton and deuterium if the trapping electrophile is excluded. We measured exactly this isotope exchange, for both Sc(OTf)_3_ and Cu(OTf)_2_ by recovering starting materials that remained after 24 hours of exposure to otherwise typical reaction conditions that lacked DEAD (Fig. 3). Sc(OTf)_3_ demonstrated the expected behaviour for the ionic mechanism. Pyrimidine, thiazole, and pyridine had shown zero, low, and high reactivity, respectively, for C–N bond formation using Sc(OTf)_3_. These three heterocycle types displayed the same relative reactivity toward isotope exchange, consistent with both reactions proceeding through the same intermediate.

When these experiments were repeated with Cu(OTf)_2_, no exchange was observed with either **4** or **8**, despite the fact that these substrates gave significant yield for C–N bond formation. Exchange in pyridine **29** was also much lower, as compared to Sc(OTf)_3_ or to the yield observed for C–N bond formation. These data strongly suggest that C–N bond formation does not occur through a dearomatized intermediate when Cu(OTf)_2_ is used. The results of these isotope exchange measurements raised an obvious question: if Cu(OTf)_2_ cannot dearomatize a difficult substrate such as **8**, how does it deliver high yield in the absence of added HAT catalyst?

**Fig. 4:**
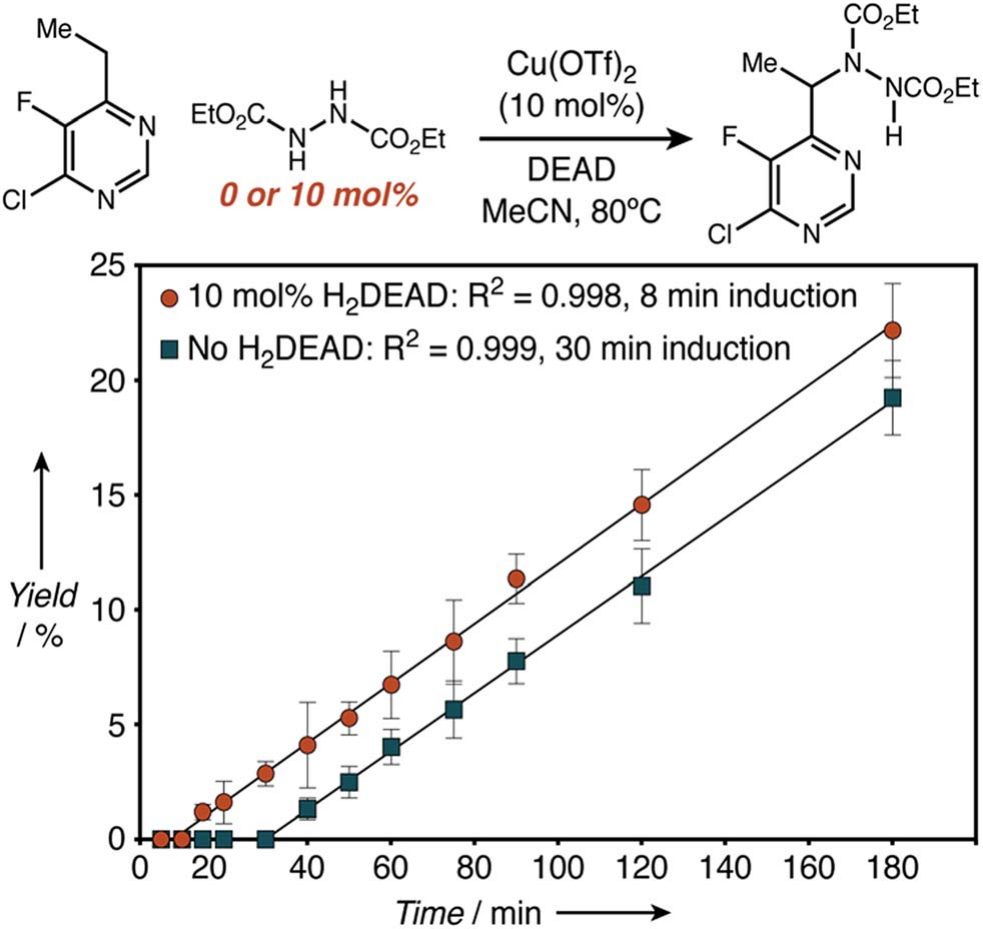
Initial rate kinetics as observed for a C–H amination reaction with H_2_DEAD either added or excluded.

**Fig. 5:**
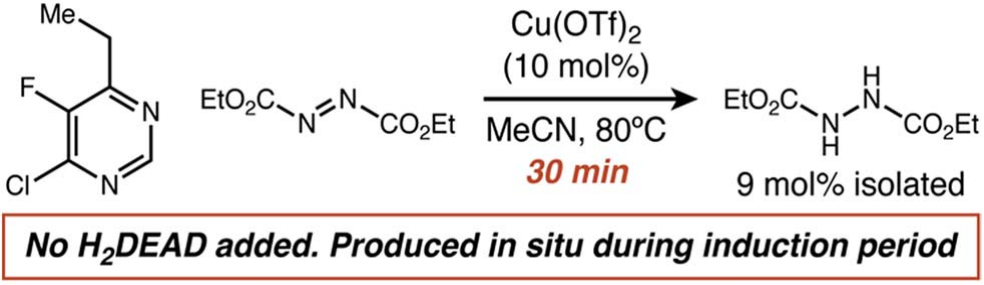
Isolation of H_2_DEAD from *in situ* generation during induction period.

A comparison of the kinetics of two aminations of **8**, with or without H_2_DEAD added, allowed us to rectify these observations (Fig. 4). While both reactions eventually reached an identical initial rate and delivered nearly identical amounts of product, H_2_DEAD reduced the induction period. Further, when an experiment using no H_2_DEAD was stopped after 30 minutes, a significant quantity of it (9 mol%) were isolated (Fig. 5). In fact, observing H_2_DEAD in the crude reaction mixtures for the reactions featuring copper during the direct GC analysis to gather data for Scheme 1 was the original reason we included it as a putative HAT catalyst in our screens. This allowed us to explain the behaviour of copper: even in cases with no exogenous HAT catalyst added, *in situ* reduction of DEAD to H_2_DEAD provides the necessary HAT catalyst and allows a radical process to operate. Anecdotally, for many substrates other than **8** we have found the addition of small amounts of H_2_DEAD at the outset of the reaction reproducibly delivered 5–10% higher yields. Additionally, the reaction could be completely inhibited by the addition of one equivalent of TEMPO, which is consistent with our proposal of a radical reaction pathway. Functionalization of alkylpyridines by an ionic pathway has previously been demonstrated to be insensitive to this additive.9*d* Reactions catalyzed by both copper (*k*_H_/*k*_D_ = 3.3) and scandium (*k*_H_/*k*_D_ = 5.9) displayed primary kinetic isotope effects. Unfortunately, the magnitude of these effects cannot be used to differentiate between radical and ionic pathways. Values ranging from 2.2 to 11 have been observed for radical reactions using heteroatom abstractors such as NHPI,^16^ while Lewis-acid catalyzed ionic pathways have demonstrated values ranging from 1.5 to 7.6.^17^

## Conclusions

Taken together, all the results herein present a consistent mechanistic picture for why this dual catalytic system is able to display a significantly expanded reaction scope. Cu(OTf)_2_ does not have the ability to access the dearomatized intermediates necessary for reaction through an ionic pathway under our specific reaction conditions, which feature relatively low temperatures and a polar solvent. However, its combination with an organic HAT catalyst—either H_2_DEAD or HOAt—enables a radical mechanism for C–N bond formation and allows for highly selective HAT adjacent to azaheterocycles. When no organic catalyst is added at the outset of the reaction, H_2_DEAD is formed *in situ* during an initial induction period. Our discovery that H_2_DEAD/Cu(OTf)_2_ is the most appropriate catalyst combination for 4-alkyl substituted heterocycles, while HOAt/Cu(OTf)_2_ is necessary to observe even moderate yields with 2-substitution, further suggests that new organic catalyst design will be useful in crafting even more highly reactive systems.

## Conflicts of interest

There are no conflicts to declare.

## Supplementary Material

Supplementary information
