# Supplementary material for: A highly site-selective radical sp3 C–H amination of azaheterocycles
Source: Chem Sci. 2018 Jul 10;9(30):6440–5. doi: 10.1039/c8sc00590g (PMC6115697; doi:10.1039/c8sc00590g)
Supplement: Supplementary file 1 [file SC-009-C8SC00590G-s001.pdf]

Electronic Supplementary Material (ESI) for Chemical Science.
This journal is © The Royal Society of Chemistry 2018

Supporting Information for:

A Highly Site-Selective Radical sp^3 Amination of Azaheterocycles

Dr. Keith W. Bentley, Krysta A. Dummit, Prof. Dr. Jeffrey F. Van Humbeck

Department of Chemistry, Massachusetts Institute of Technology, Cambridge, MA 02139, United States
Department of Chemistry, University of Calgary, Calgary, AB T2N 1N4, Canada

E-mail: jeffrey.vanhumbec1@ucalgary.ca

Contents:

1. General Information S2
2. Synthesis and Characterization of Azaheterocycles S2
3. Initial metal and organic catalyst screening experiments S46
4. Amination of *N*-heterocycles with diethyl azodicarboxylate (DEAD) S49
5. Isotope exchange measurements S114
6. Kinetic measurements, isotope effects, and supporting experiments S117
7. References S119

1. General Information

All reagents were purified by initially drying over acidic alumina for one hour under nitrogen atmosphere, then filtering directly into 1L Schlenk bottles containing 3Å molecular sieves, and degassing with bubbling nitrogen. Sovents for extraction and filtration were ACS grade and used without further purification. All reagents for substrate synthesis were used as received unless otherwise noted. Commercially available liquid reagents used for amination were freshly distilled and stored under N_2 in 100 mL Schlenk flasks. Diethyl azodicarboxylate was purchased pure and redistilled into a 100 mL Schlenk flask and stored under N_2 at 0 °C. ^{1}H and ^{13}C NMR spectra were acquired on Varian 300 and 500 MHz spectrometers.

2. Synthesis and Characterization of Azaheterocycles

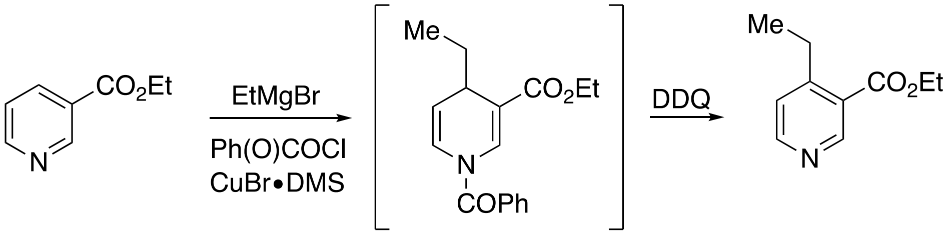

Ethyl 4-ethylnicotinate [1] A flame-dried 2-neck flask under N_2 was charged with CuBr•DMS (272 mg, 1.3 mmol), ethyl nicotinate (2g, 13.2 mmol) and 40 mL anhydrous THF. The mixture was cooled to -78 °C and phenyl chloroformate (1.8 mL, 14.5 mmol) was added dropwise. The reaction mixture was stirred at -78 °C for 30 min., followed by dropwise addition of EtMgBr (3M in diethyl ether, 4.9 mL, 14.5 mmol). The reaction mixture was stirred at -78 °C for 90 min. and quenched at -78 °C with aqueous NH_4Cl (10 mL). The mixture was warmed to room temperature, extracted with EtOAc, and washed with 1M HCl. The organic layer was concentrated *in vacuo* and the crude reaction mixture was used without purification. A flame-dried 2-neck flask under N_2 was charged with the crude reaction mixture and 30 mL anhydrous EtOAc. DDQ (2.1 g, 9.3 mmol) was added in a single portion and stirred exactly 10 min. The reaction was quenched with 1M HCl and the organic layer was extracted three times with 1M HCl. The combined aqueous layers were washed once with EtOAc, then basified with 1M NaOH to pH 10, resulting in a milky mixture. The aqueous layer was extracted three times with EtOAc and the combined organic layers were dried over $MgSO_4$ and concentrated *in vacuo*. Colum chromatography over silica gel (1:1 hexane:EtOAc) afforded 1.04 g (5.8 mmol, 44%) of a light yellow oil.
^{1}H NMR ($CDCl_3$) δ = 9.04 (s, 1H), 8.59 (d, J = 5.1 Hz, 1H), 7.21 (d, J = 5.1 Hz, 1H), 4.40 (q, J = 7.1 Hz, 2H), 3.01 (q, J = 7.5 Hz, 2H), 1.41 (t, J = 7.1 Hz, 3H), 1.25 (t, J = 7.5 Hz, 3H)

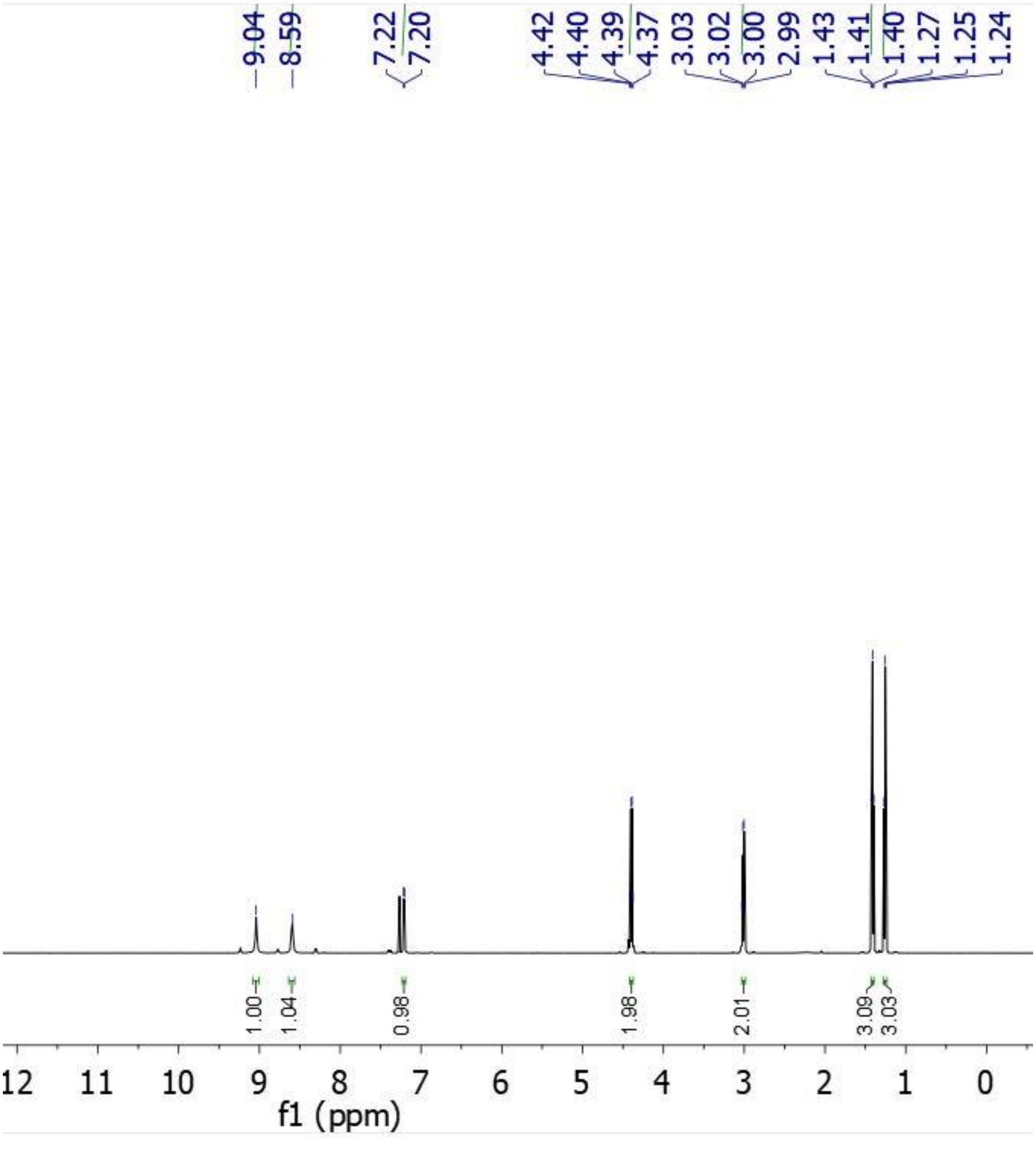

Figure S1: ^{1}H NMR of ethyl 4-ethylnicotinate

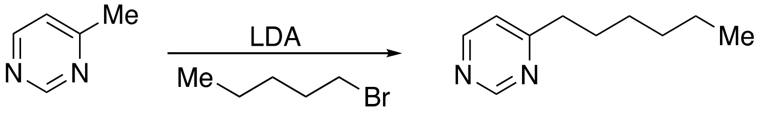

4-Hexylpyrimidine A flame-dried 2-neck flask under N_2 was charged with 3-methylpyrimidine (0.9 mL, 10 mmol) and 12 mL anhydrous THF, and the mixture was cooled to -78 °C. Freshly prepared LDA (12 mmol in 13 mL anhydrous THF) was added dropwise, and the reaction mixture was stirred at -78 °C for 30 min. 1-Bromopentane (1.2 mL, 10 mmol) was slowly added, and the reaction was warmed to room temperature and stirred for 10 minutes. The reaction was quenched with 1M HCl to pH 7.5 and extracted with dichloromethane. The combined organic layers were dried over $MgSO_4$ and concentrated *in vacuo*. Column chromatography over silica gel (9:1 hexane:EtOAc) afforded 590 mg (3.59 mmol, 36%) of a light yellow oil.

^{1}H NMR ($CDCl_3$) δ = 8.99 (d, *J* = 1.4 Hz, 1H), 8.46 (d, *J* = 5.2 Hz, 1H), 7.05 (dd, *J* = 5.3, 1.4 Hz, 1H), 2.64-2.61 (m, 2H), 1.63-1.57 (m, 2H), 1.25-1.16 (m, 6H), 0.76-0.72 (m, 3H).). ^{13}C NMR ($CDCl_3$) δ = 170.9, 158.6, 156.6, 120.4, 37.8, 31.6, 28.9, 28.8, 22.5, 14.0. HRMS m/z calcd. for $C_{10}H_{16}N_2$ $[M+H]^+$ 165.1386, found 165.1384.

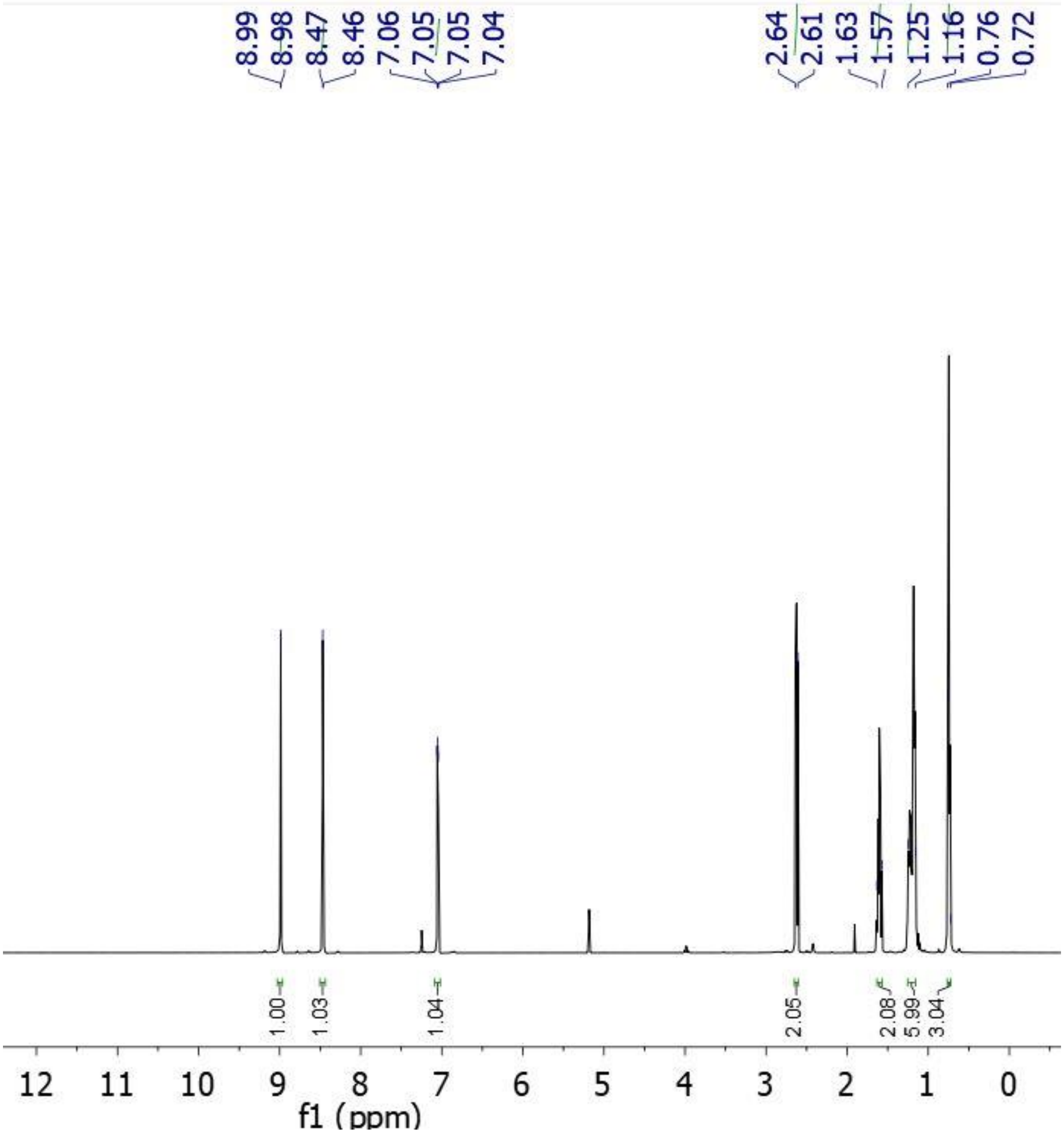

Figure S2: ^{1}H NMR of 4-hexylpyrimidine

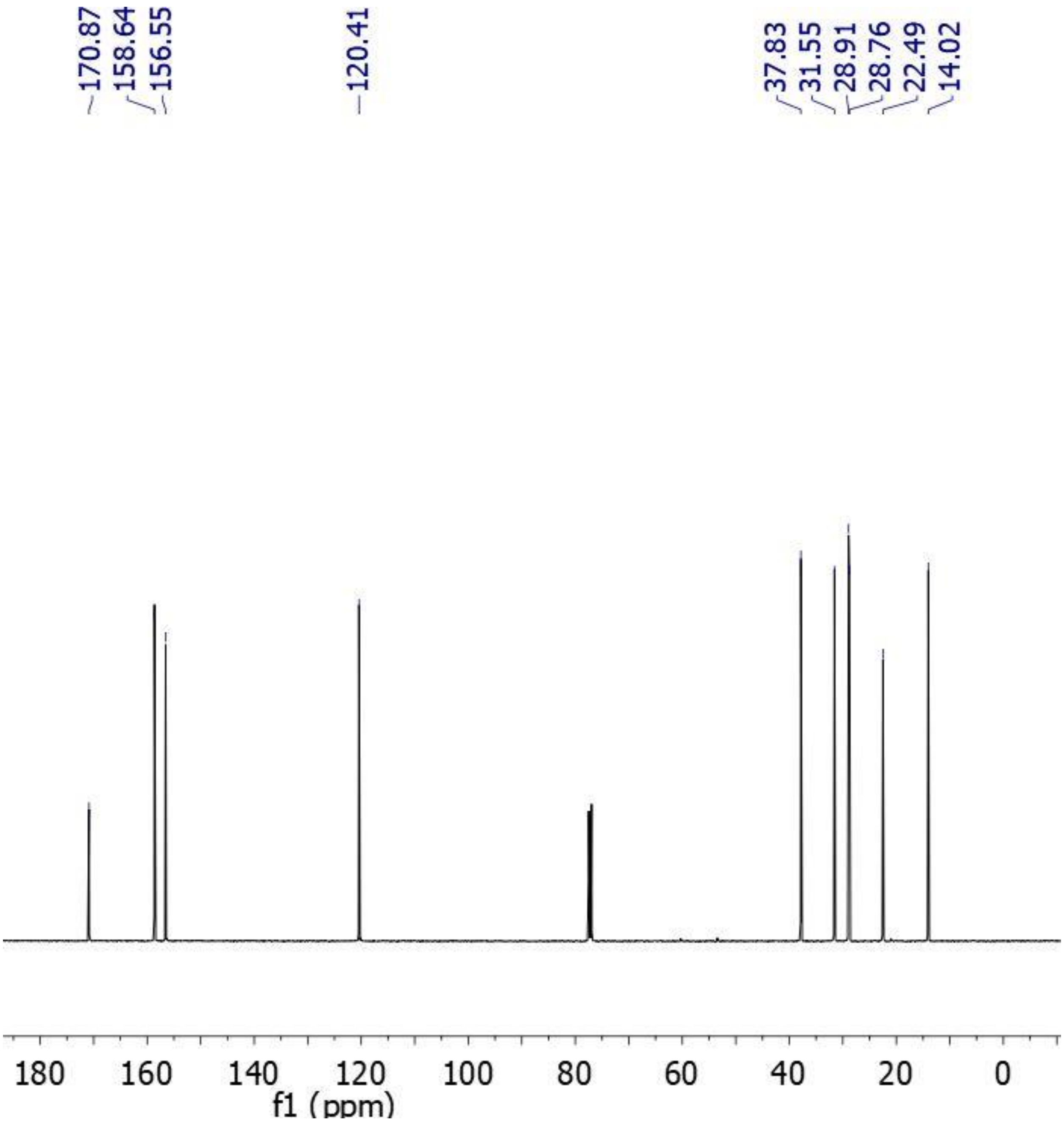

Figure S3: ^{13}C NMR of 4-hexylpyrimidine

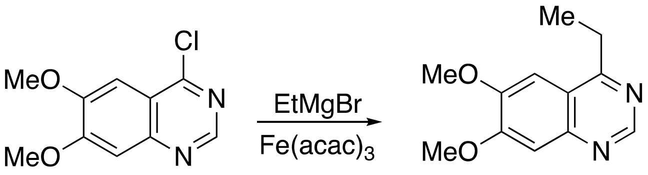

4-Ethyl-6,7-Dimethoxyquinazoline[2] A flame-dried 2-neck flask under N_2 was charged with 4-chloro-6,7-dimethoxyquinazoline (1g, 4.5 mmol), $Fe(acac)_3$ (78.6 mg, 0.23 mmol), 12 mL anhydrous THF and 1 mL anhydrous NMP. To this stirred mixture at room temperature was added dropwise EtMgBr (3M in diethyl ether, 1.8 mL, 5.3 mmol). The reaction mixture was stirred for 1 hour, then quenched with H_2O. The resulting mixture was extracted with EtOAc, and the combined organic layers were washed once with aqueous NaCl, once with 1M aqueous sodium ascorbate, and four times with H_2O. The organic layer was dried over $MgSO_4$ and concentrated *in vacuo*. Column chromatography over silica gel (2:1 hexane:EtOAc) afforded 728 mg (3.6 mmol, 80%) of a white solid.

^{1}H NMR ($CDCl_3$) δ = 9.04 (s, 1H), 7.30 (s, 1H), 7.23 (s, 1H), 4.03 (d, J = 2.2 Hz, 6H), 3.20 (q, J = 7.6 Hz, 2H), 1.44 (t, J = 7.5 Hz, 3H)

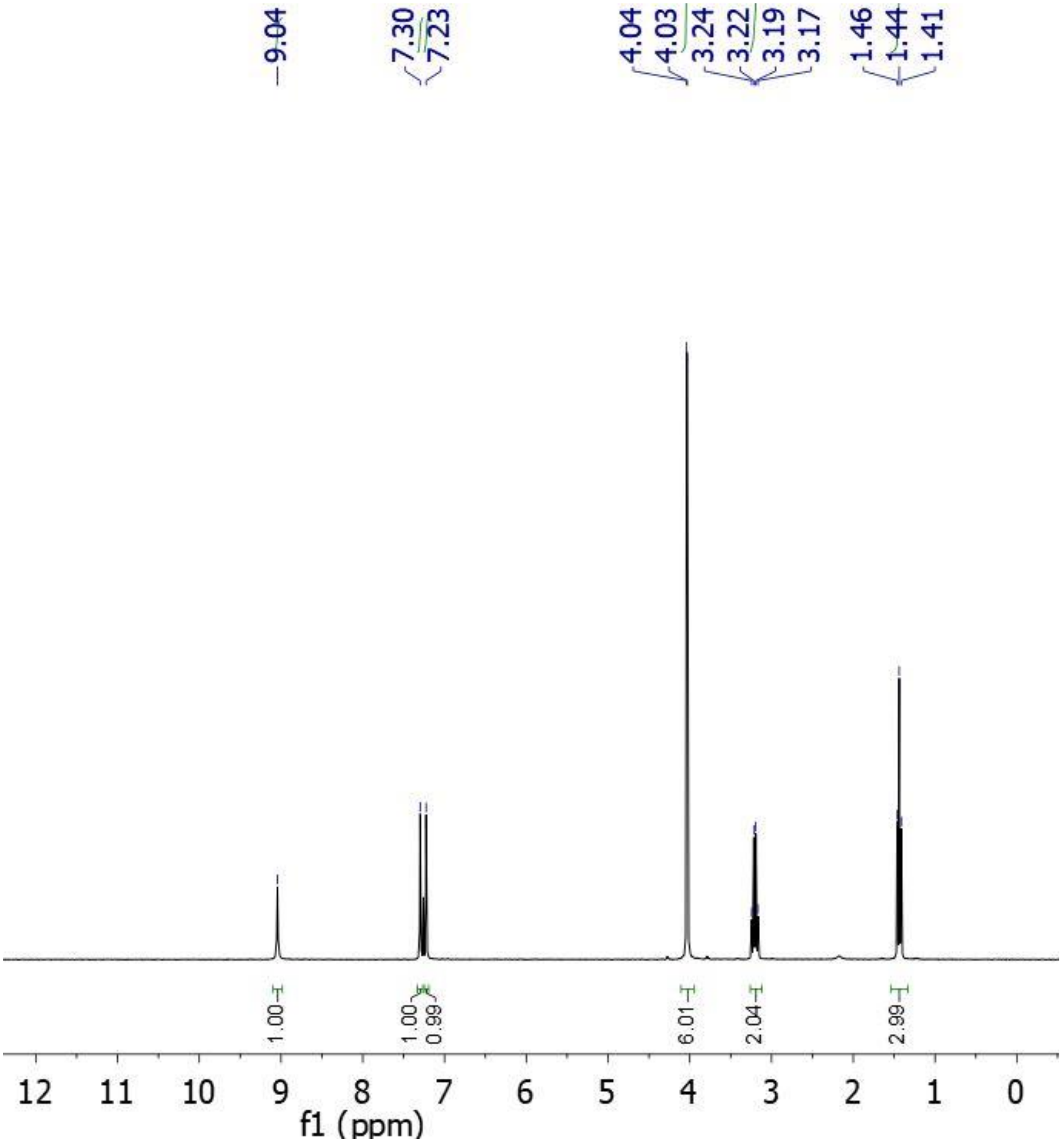

Figure S4: ^{1}H NMR of 4-Ethyl-6,7-Dimethoxyquinazoline

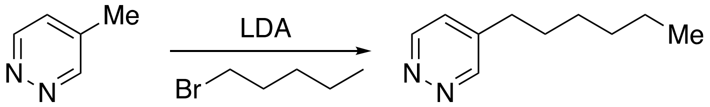

4-Hexylpyridazine[3] A flame dried 2-neck flask under N_2 was charged with 4-methylpyridazine (3g, 31 mmol) and 150 mL anhydrous THF. The mixture was cooled to -78 °C and freshly-prepared LDA (33 mmol in 15 mL THF) was added dropwise. The reaction mixture was stirred at -78 °C for 1 hour, then 1-bromopentane (4.8g, 33 mmol) was added. The reaction mixture was then warmed to room temperature and stirred overnight. The reaction was quenched with aqueous NH_4Cl and extracted with EtOAc. The combined organic layers were dried over $MgSO_4$ and concentrated *in vacuo*. Column chromatography over silica gel (20:1 EtOAc:MeOH) afforded 2.22 g (13.0 mmol, 42%) of a light yellow oil.

^{1}H NMR ($CDCl_3$) δ = 9.09-8.99 (m, 2H), 7.30-7.25 (m, 1H), 2.65-2.57 (m, 2H), 1.69-1.57 (m, 2H), 1.39-1.25 (m, 6H), 0.93-0.80 (m, 3H).

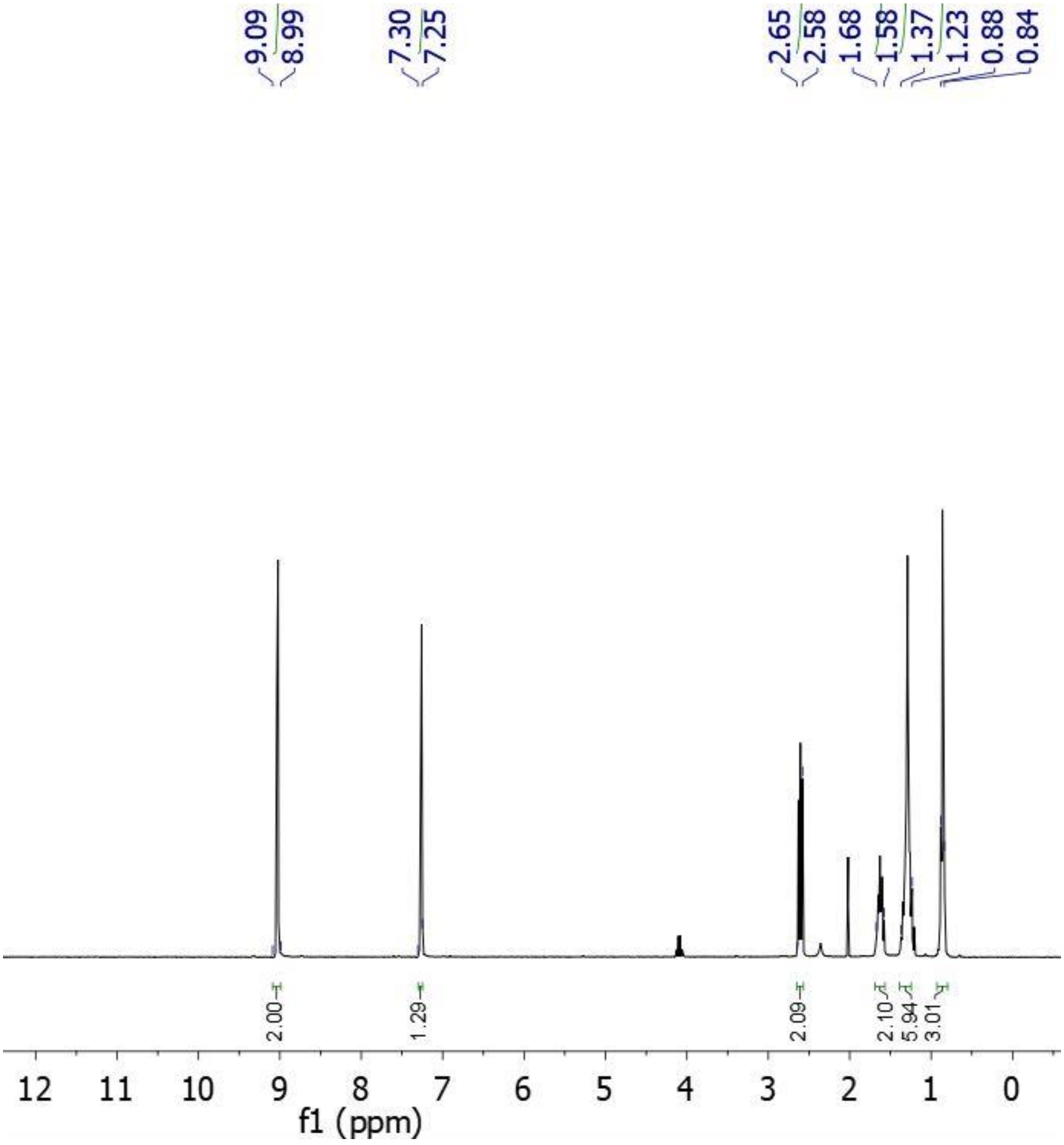

Figure S5: ^{1}H NMR of 4-Hexylpyridazine

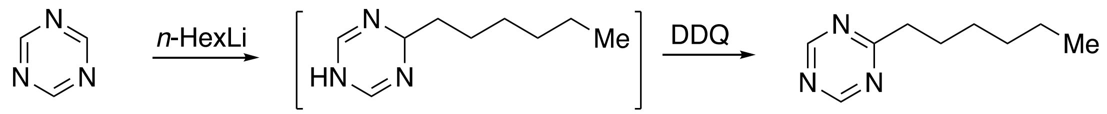

2-Hexyl-1,3,5-triazine A flame dried 3-neck flask under N_2 was charged with 1,3,5-triazine (810 mg, 10 mmol) and 20 mL anhydrous diethyl ether. The solution was cooled to -40 °C and *n*-hexyllithium (2.3 M in hexane, 4.3 mL, 10 mmol) was added dropwise. The reaction was left to warm to room temperature and stirred for 4 hours. The reaction mixture was quenched with H_2O and stirred for an additional hour and was then extracted with dichloromethane. The combined organic layers were dried over $MgSO_4$ and concentrated *in vacuo*. The crude reaction mixture was redissolved in anhydrous dichloromethane and transferred to a flame-dried 2-neck flask under N_2. MnO_2 (4.3 g, 50 mmol) was added in several portions and the mixture was heated to reflux overnight. After cooling to room temperature, the mixture was filtered over celite and concentrated *in vacuo.* Column chromatography over silica gel (3:1 hexane:EtOAc) afforded 437 mg (2.9 mmol, 29%) of a colorless oil.

^{1}H NMR ($CDCl_3$) δ = 9.07 (s, 2H), 2.94-2.84 (m, 2H), 1.86-1.76 (m, 2H), 1.42-1.24 (m, 6H), 0.94-0.79 (m, 3H). ^{13}C NMR ($CDCl_3$) δ = 180.0, 165.8, 39.0, 31.6, 29.0, 27.7, 22.5, 14.1. HRMS m/z calcd. for $C_9H_{15}N_3$ $[M+H]^+$ 166.1339, found 166.1338.

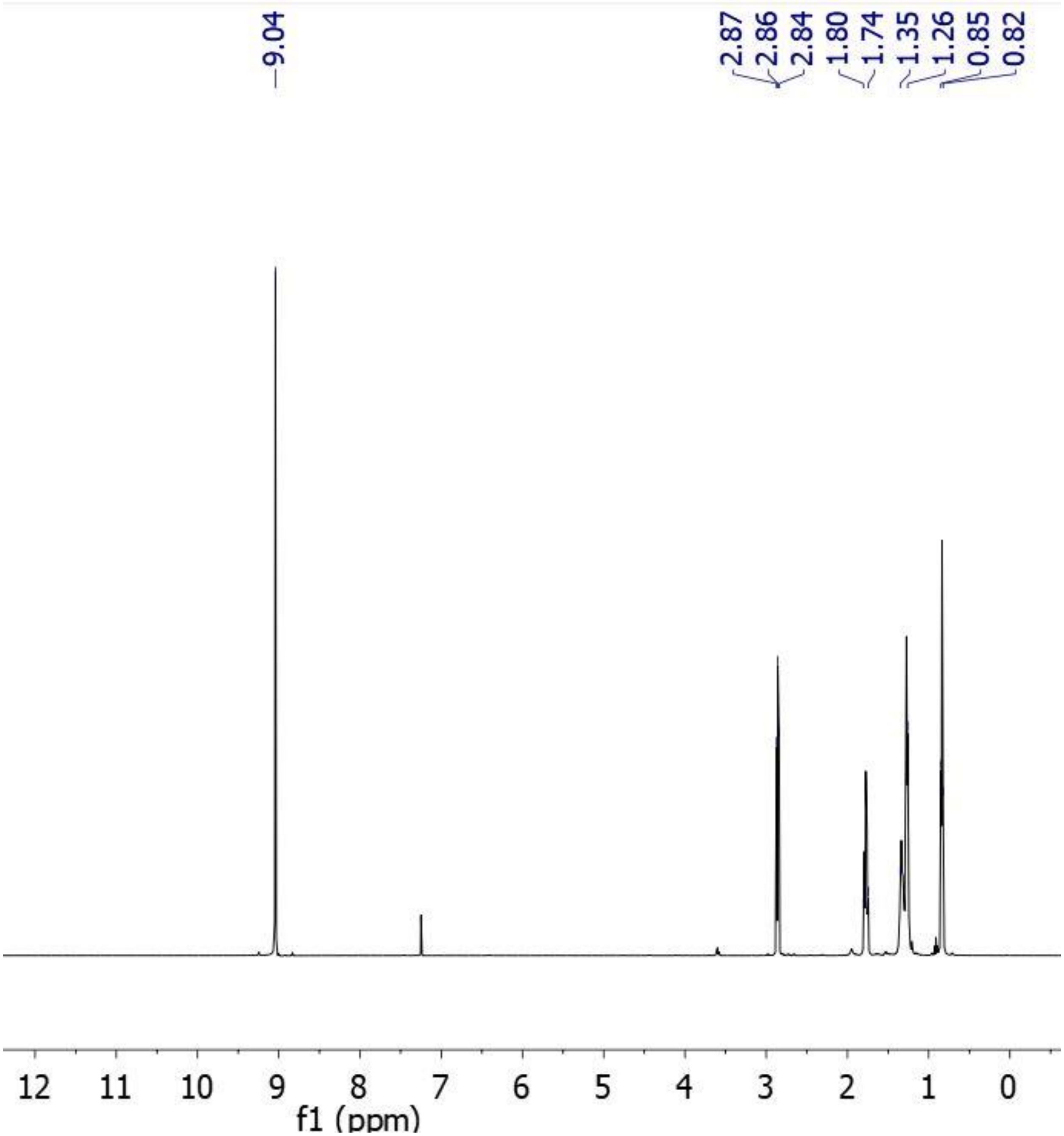

Figure S6: ^{1}H NMR of 2-Hexyl-1,3,5-triazine

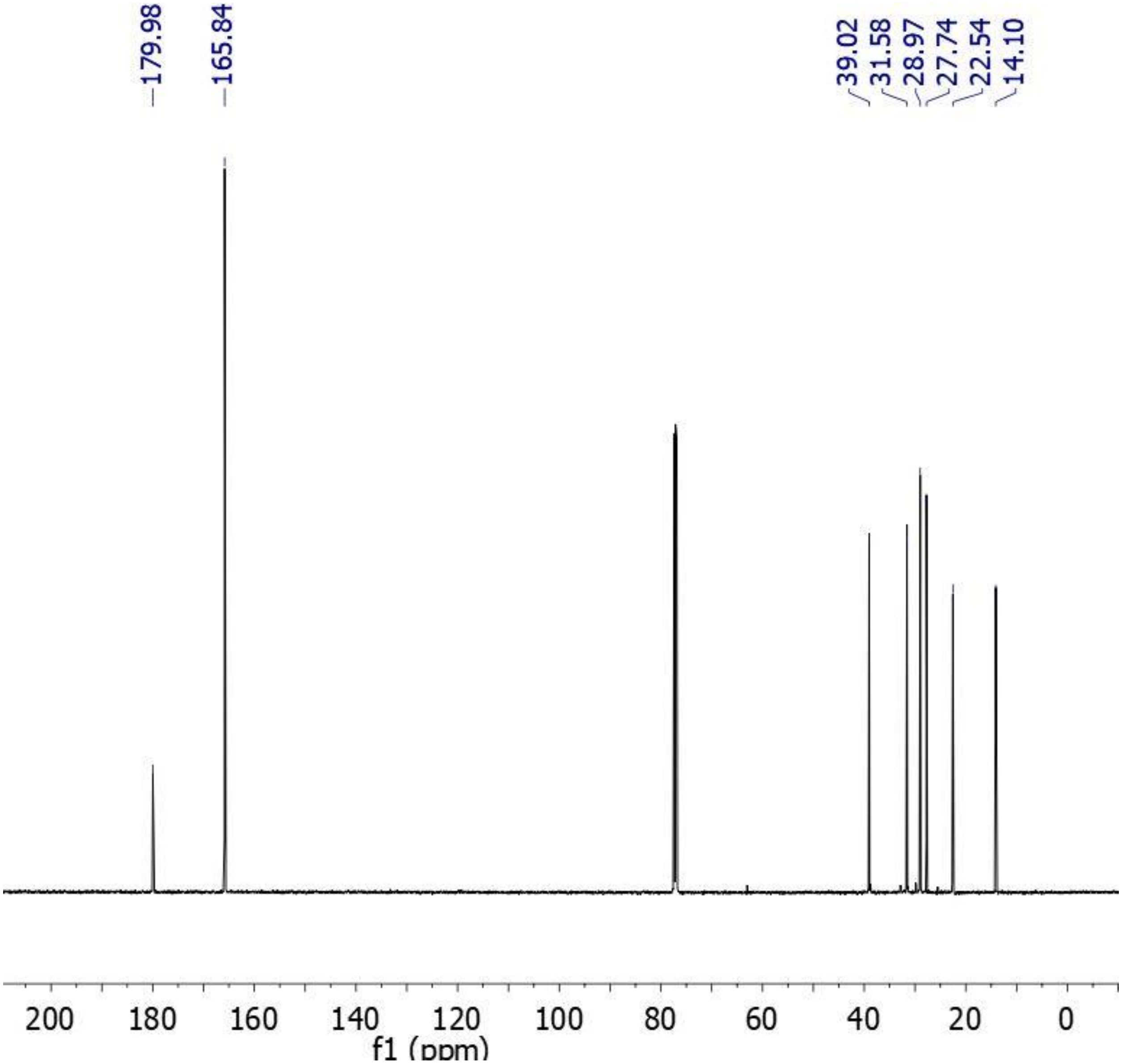

Figure S7: ^{13}C NMR of 2-Hexyl-1,3,5-triazine

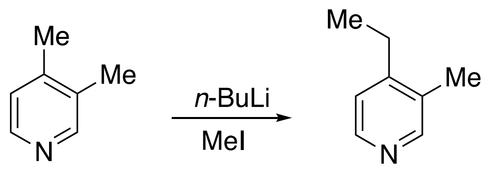

4-Ethyl-3-methylpyridine[4] A flame-dried 2-neck flask under N_2 was charged with 3,4-lutidine (1g, 9.7 mmol) and 30 mL anhydrous THF. The stirred solution was cooled to -78 °C, and *n*-BuLi (2.5 M in hexanes, 4.5 mL, 10.7 mmol) was added dropwise, resulting in a clear, orange mixture. The reaction mixture was heated to 45 °C for 2 hours, then cooled to 0 °C in an ice bath. The mixture was transferred via cannula to a separate 3-neck flask under N_2 containing MeI (0.67 mL, 10.7 mmol) and 10 mL anhydrous THF at -78 °C. After the transfer was complete, the reaction mixture was warmed to room temperature and stirred overnight. The reaction was quenched with H_2O and extracted with dichloromethane. The organic layer was washed with aqueous $NaHCO_3$ and NH_4Cl. The combined organic layers were dried over $MgSO_4$ and concentrated *in vacuo*. Column chromatography over silica gel (4:1 EtOAc:hexane) afforded 690 mg (5.7 mmol, 58%) of a light yellow oil.

^{1}H NMR ($CDCl_3$) δ = 8.35-8.32 (m, 2H), 7.06 (d, *J* = 5.0 Hz, 1H), 2.61 (q, *J* = 7.5 Hz, 2H), 2.27 (s, 3H), 1.22 (t, *J* = 7.6 Hz, 3H)

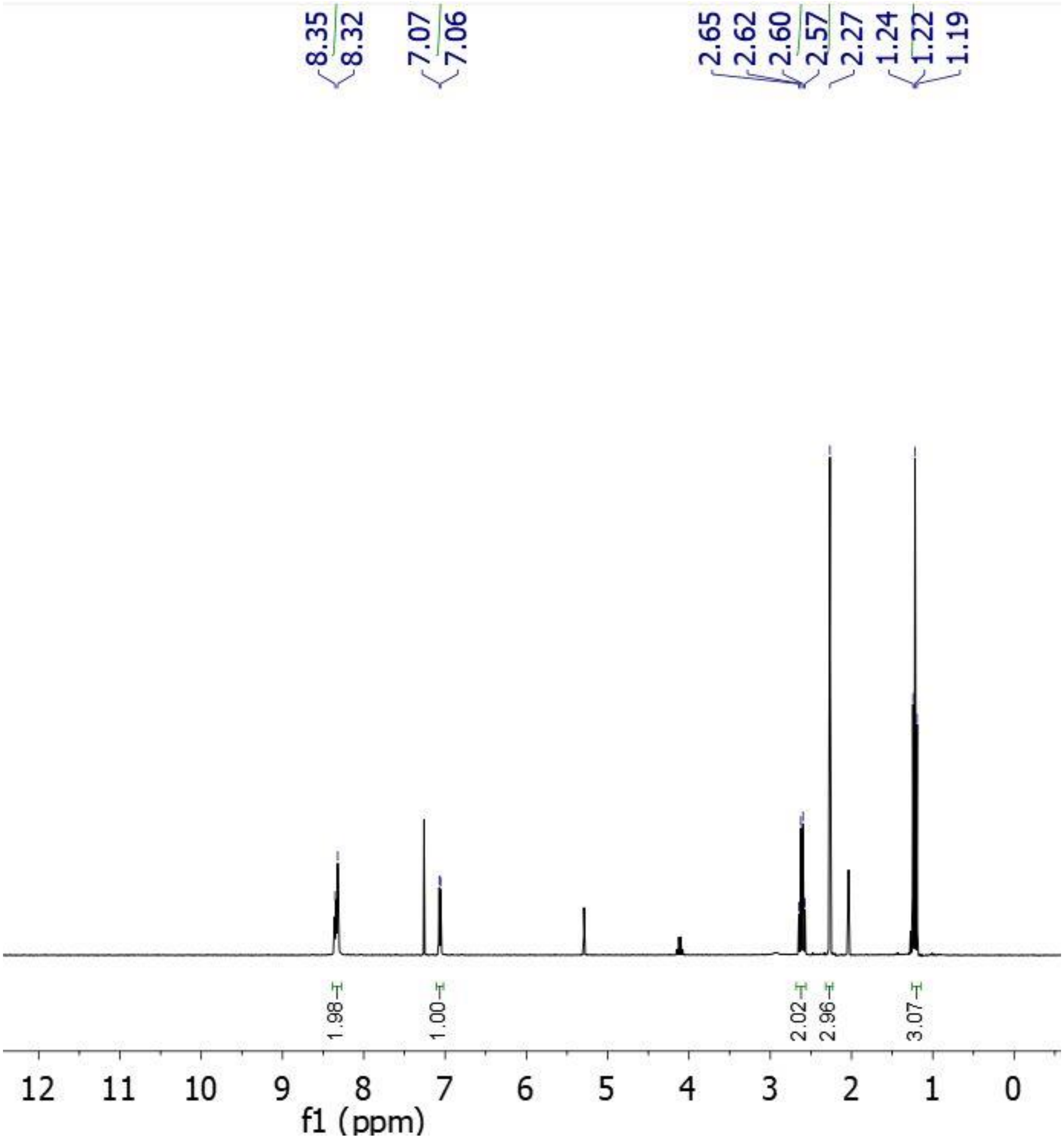

Figure S8: ^{1}H NMR of 4-Ethyl-3-methylpyridine

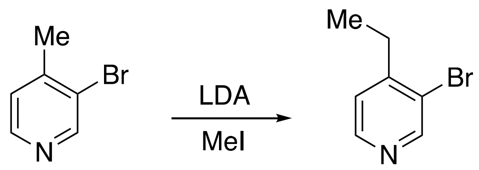

3-Bromo-4-Ethylpyridine[5] A flame dried flask 2-neck flask under N_2 was charged with 3-bromo-4-methylpyridine (5g, 29.1 mmol) and 100 mL anhydrous THF. The stirred solution was cooled to -78 °C, and freshly-prepared lithium diisopropyl amide (LDA) (34.8 mmol) was added dropwise. The reaction was stirred at -78 °C for 30 min., followed by addition of MeI (2.4 mL, 37.8 mmol). The reaction mixture was stirred for an additional 30 min. at -78 °C, and was then warmed to room temperature and stirred for 3 hours. The reaction mixture was quenched with $NaHCO_3$ and extracted with dichloromethane. The organic layer was dried over $MgSO_4$ and concentrated *in vacuo*. Column chromatography over silica gel (2:1 hexane:EtOAc) afforded 5.3 g (28.8 mmol, 98%) of a light yellow oil.
^{1}H NMR ($CDCl_3$) δ = 8.65 (s, 1H), 8.42 (d, J = 5.0 Hz, 1H), 7.19 (d, J = 4.9 Hz, 1H), 2.76 (q, J = 7.5 Hz, 2H), 1.25 (t, J = 7.5 Hz, 3H)

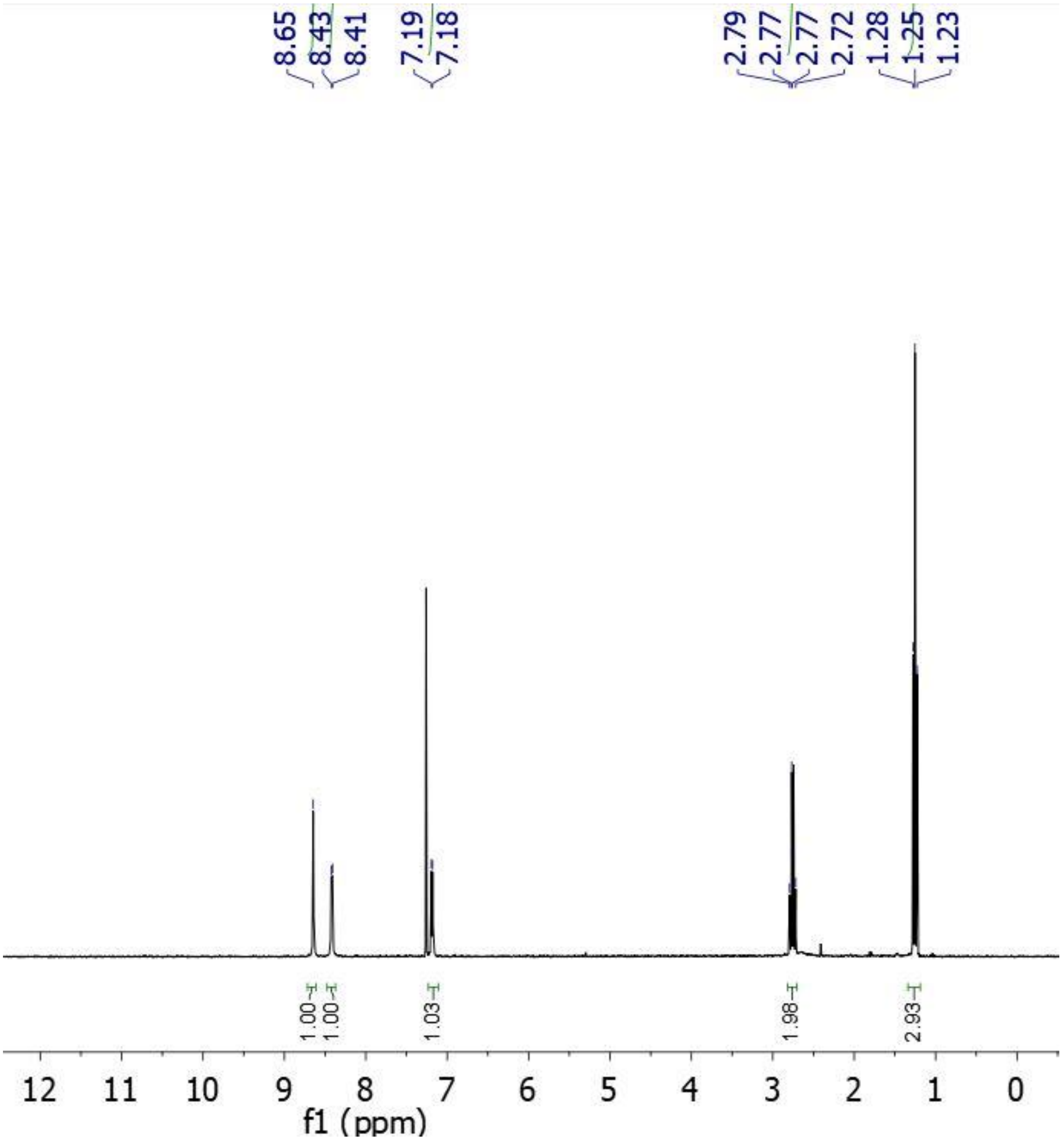

Figure S9: ^{1}H NMR of 3-Bromo-4-Ethylpyridine

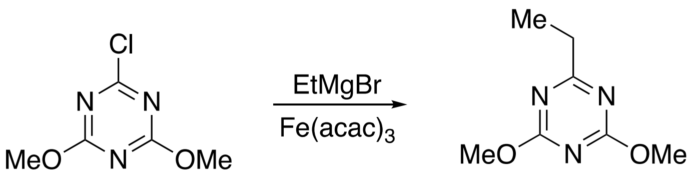

4-Ethyl-2,6-dimethoxy-1,3,5-triazine[6] A flame-dried 2-neck flask under N_2 was charged with 2-chloro-4,6-dimethoxy-1,3,5-triazine (1.3 g, 7.5 mmol), $Fe(acac)_3$ (132 mg, 0.38 mmol), 40 mL anhydrous THF, and 10 mL anhydrous NMP. To this stirred reaction mixture at room temperature was added EtMgBr (3M in diethyl ether, 3 mL, 9 mmol) dropwise. The reaction mixture was stirred for 1 hour at room temperature. The reaction was quenched with H_2O and extracted with EtOAc. The combined organic layers were washed once with aqueous NaCl, once with 1M aqueous sodium ascorbate, and four times with H_2O. The organic layer was dried over $MgSO_4$ and concentrated *in vacuo*. Column chromatography over silica gel (2:1 hexane:EtOAc) afforded 719 mg (4.3 mmol, 57%) of a white solid.
^{1}H NMR ($CDCl_3$) δ = 4.03 (s, 6H), 2.76 (q, J = 7.6 Hz, 2H), 1.31 (t, J = 7.6 Hz, 3H).

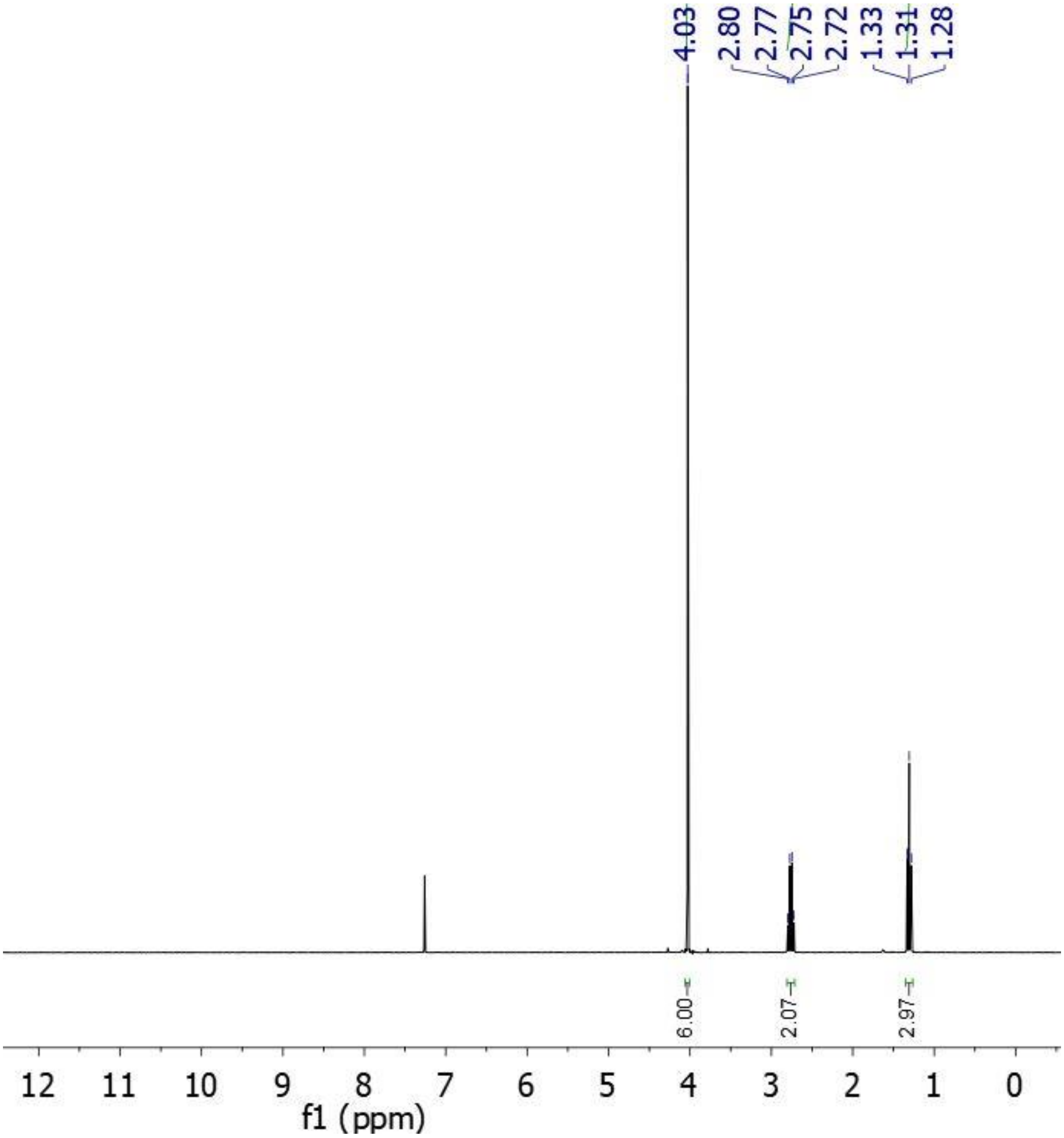

Figure S10: ^{1}H NMR of 4-Ethyl-2,6-dimethoxy-1,3,5-triazine

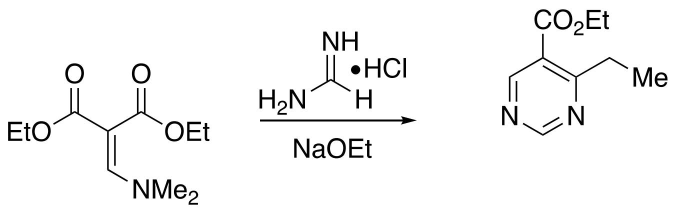

4-Ethylpyrimidine-3-ethylester[7] A flame dried 2-neck flask under N_2 was charged with ethyl 2-[(dimethylamino)methylidine]-3-oxopentanoate (1g, 5.8 mmol), formamidine HCl (470 mg, 5.8 mmol), NaOEt (397 mg, 5.8 mmol) and 30 mL anhydrous EtOH. The reaction mixture was heated to 80 °C and stirred overnight. After cooling to room temperature, the reaction mixture was quenched with H_2O and extracted with dichloromethane. The organic layer was dried over $MgSO_4$ and concentrated *in vacuo*. Column chromatography over silica gel (3:1 hexane:EtOAc) afforded 500 mg (2.8 mmol, 47%) of a light yellow oil.

^{1}H NMR ($CDCl_3$) δ = 9.19 (s, 1H), 9.11 (s, 1H), 4.41 (q, *J* = 7.1 Hz, 2H), 3.17 (q, *J* = 2H), 1.41(t, *J* = 7.1 Hz, 3H), 1.31 (t, *J* = 7.5 Hz, 3H)

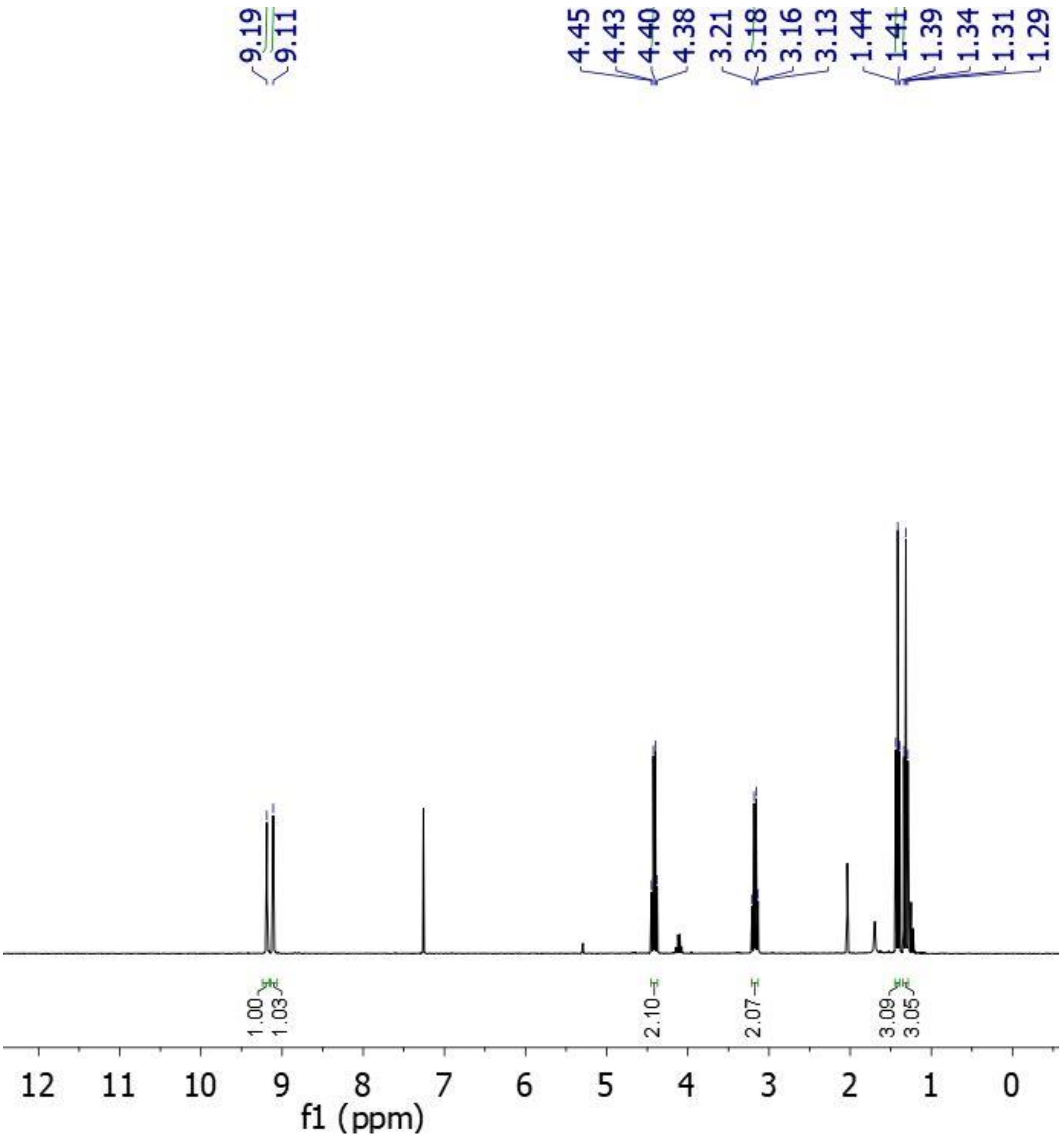

Figure S11: ^{1}H NMR of 4-Ethylpyrimidine-3-ethyl ester

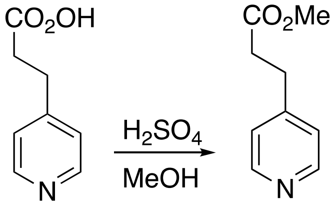

4-pyridinepropionic acid methyl ester[8] A 2-neck flask was charged with 4-pyridinepropionic acid (500 mg, 3.31 mmol), 0.4 mL H_2SO_4 (0.4 mL, 6.62 mmol) and 15 mL MeOH. The reaction mixture was heated to 65 °C and stirred 6 hours. The reaction was carefully quenched with aqueous $NaHCO_3$ and extracted with dichloromethane. The combined organic layers were dried over $MgSO_4$ and concentrated *in vacuo* to afford 464 mg (2.81 mmol, 85%) of a colorless oil.
^{1}H NMR ($CDCl_3$) δ = 8.53-8.48 (m, 2H), 7.16-7.10 (m, 2H), 3.67 (s, 3H), 2.94 (t, *J* = 7.6 Hz, 2H), 2.65 (t, *J* = 7.5 Hz, 2H).

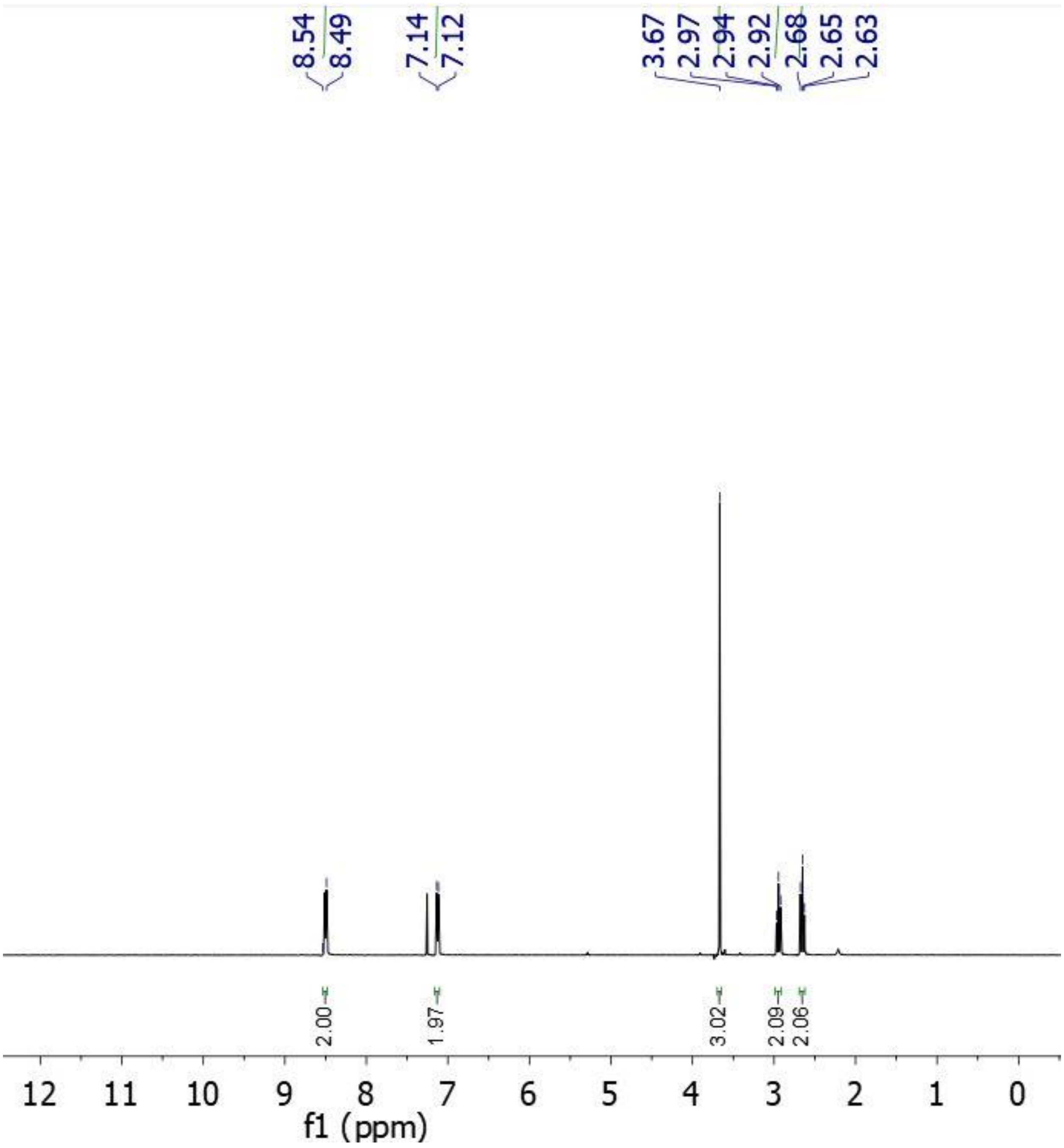

Figure S12: ^{1}H NMR of 4-pyridinepropionic acid methyl ester

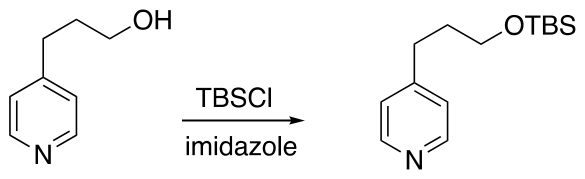

4-(3’-Propyl-*tert*-butyldimethylether)pyridine[9] A flame-dried 2-neck flask under N_2 was charged with 4-pyridinepropanol (1g, 7.3 mmol), imidazole (744 mg, 10.9 mmol) and 12 mL anhydrous dichloromethane. To this stirred solution at room temperature was added *tert*-butyldimethylsilyl chloride (1.8 g, 12.0 mmol). The reaction mixture was stirred overnight. The reaction was quenched with water and extracted with dichloromethane. The combined organic layers were dried over $MgSO_4$ and concentrated *in vacuo*. Column chromatography over silica gel (1:1 hexane:EtOAc) afforded 2.50 g (9.9 mmol, 90%) of a colorless oil.

^{1}H NMR ($CDCl_3$) δ = 8.47-8.41 (m, 2H), 7.11-7.04 (m, 2H), 3.58 (td, *J* = 6.1, 1.2 Hz, 2H), 2.64 (dd, *J* = 8.7, 6.9 Hz, 2H), 1.84-1.74 (m, 2H), 0.86 (d, *J* = 1.6 Hz, 9H), 0.01 (s, 6H).

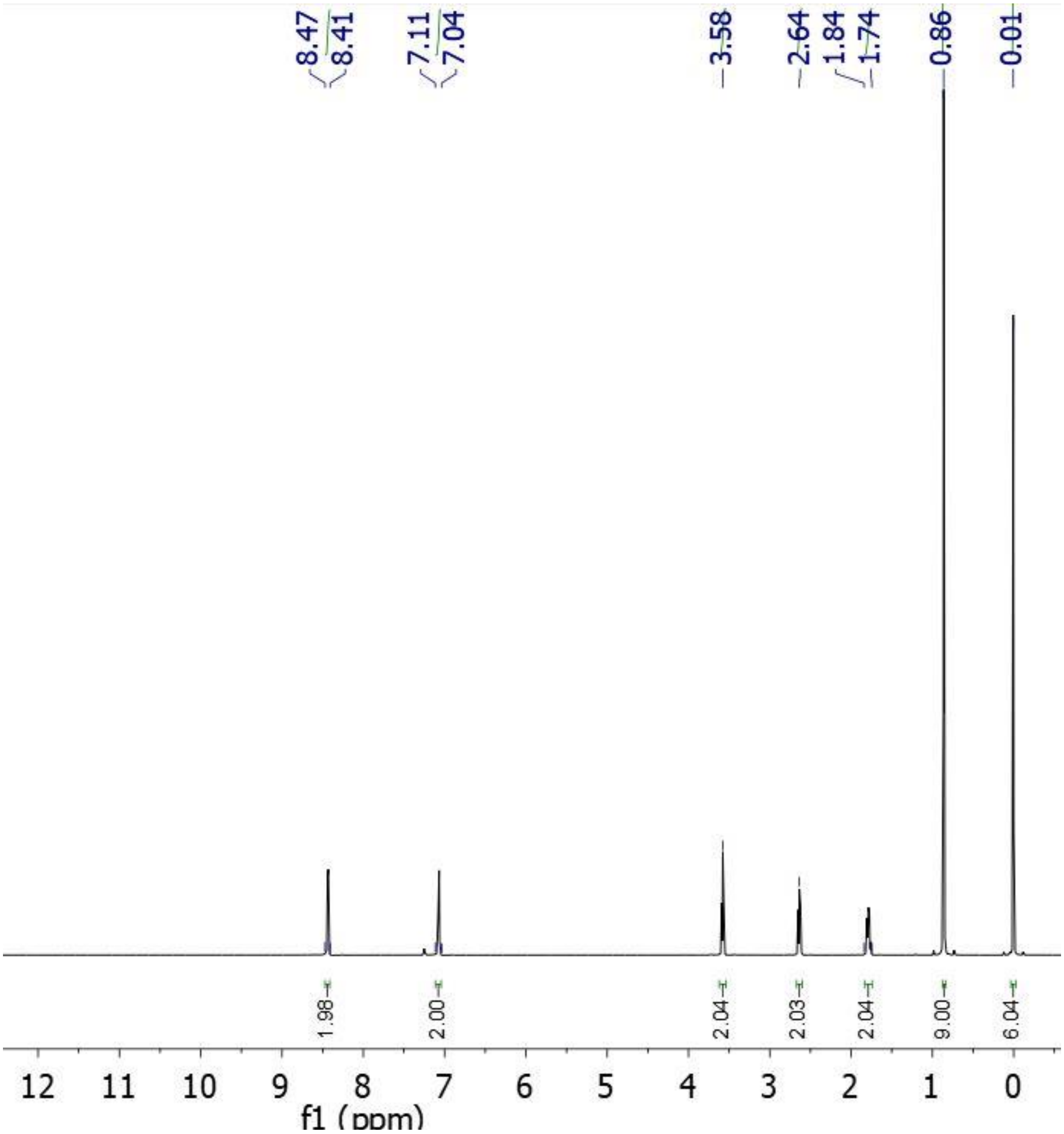

Figure S13: ^{1}H NMR of 4-(3’-Propyl-*tert*-butyldimethylether)pyridine

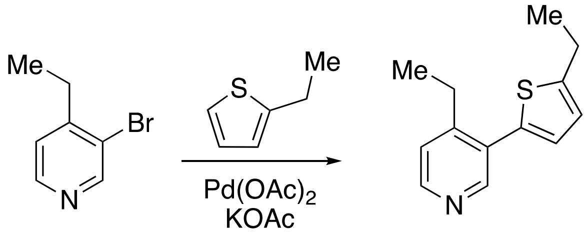

4-Ethyl-3-(5'-ethyl-2'-thiopheneyl)pyridine A flame dried 2-neck flask under N_2 was charged with 3-bromo-4-ethylpyridine (500 mg, 2.69 mmol), 2-ethylthiophene (0.61 mL, 5.37 mmol), $Pd(OAc)_2$ (30.2 mg, 0.13 mmol), KOAc (527 mg, 5.37 mmol) and 12 mL DMA. The reaction mixture was heated to 150 °C and stirred overnight. After cooling to room temperature, the reaction was quenched with H_2O and extracted with dichloromethane. The combined organic layers were dried over $MgSO_4$ and concentrated *in vacuo*. Column chromatography over silica gel (2:1 hexane:EtOAc) afforded 320 mg (1.47 mmol, 55%) of a colorless oil.
^{1}H NMR ($CDCl_3$) δ = 8.54 (s, 1H), 8.44 (d, *J* = 5.1 Hz, 1H), 7.20 (d, *J* = 5.1 Hz, 1H), 6.88 (d, *J* = 3.4 Hz, 1H), 6.80 (dt, *J* = 3.5 Hz, 1.0 Hz, 1H), 2.89 (q, *J* = 7.5 Hz, 2H), 2.79 (q, *J* = 7.5 Hz, 2H), 1.35 (t, *J* = 7.5 Hz, 3H), 1.25-1.18 (m, 3H). ^{13}C NMR ($CDCl_3$) δ = 151.02, 150.66, 148.71, 135.83, 130.62, 127.19, 123.74, 123.41, 26.09, 23.51, 15.94, 14.63. HRMS m/z calcd. for $C_{13}H_{15}NS$ $[M+H]^+$ 218.0998, found 218.0992.

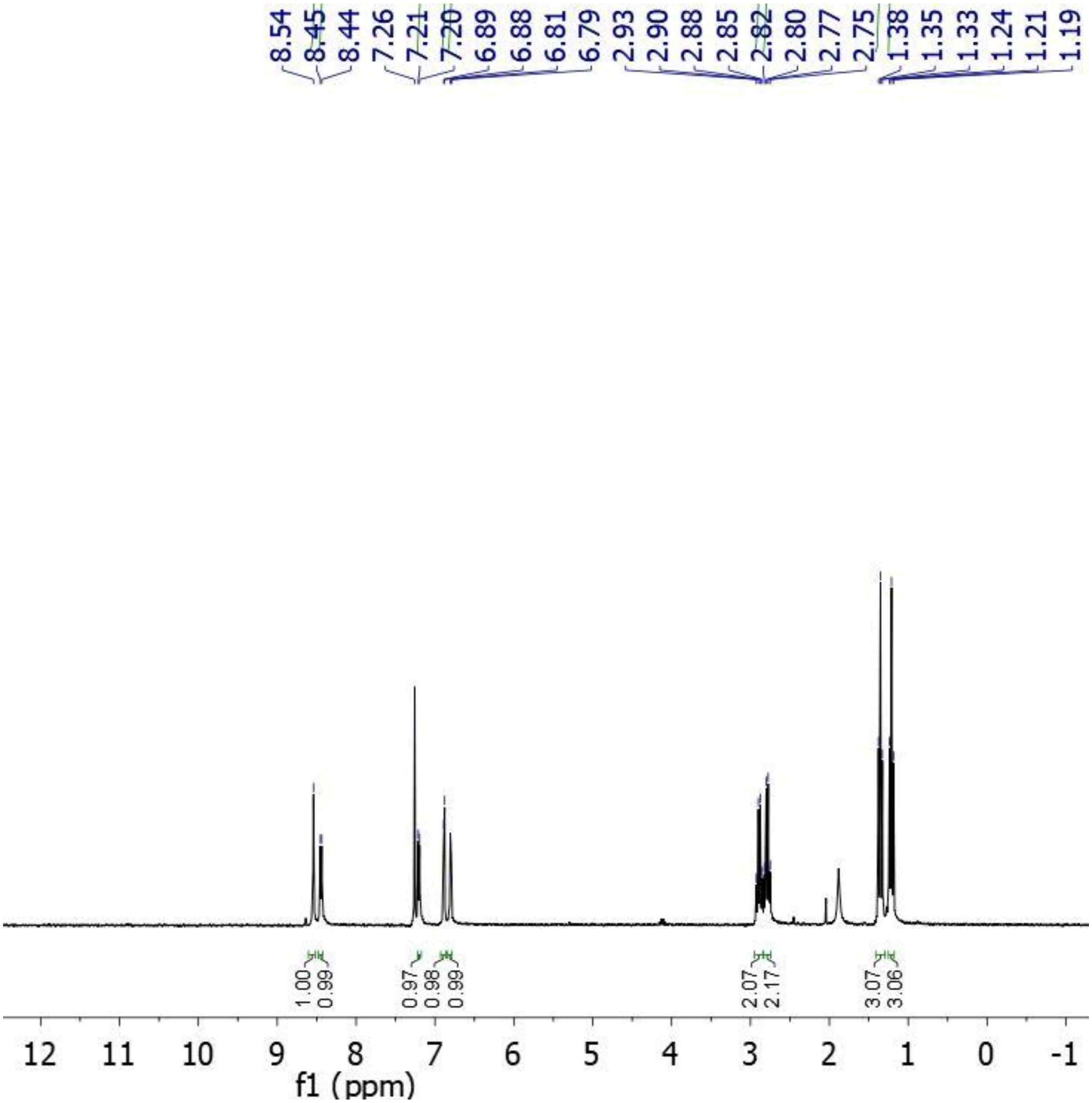

Figure S14: ^{1}H NMR of 4-Ethyl-3-(5'-ethyl-2'-thiopheneyl)pyridine

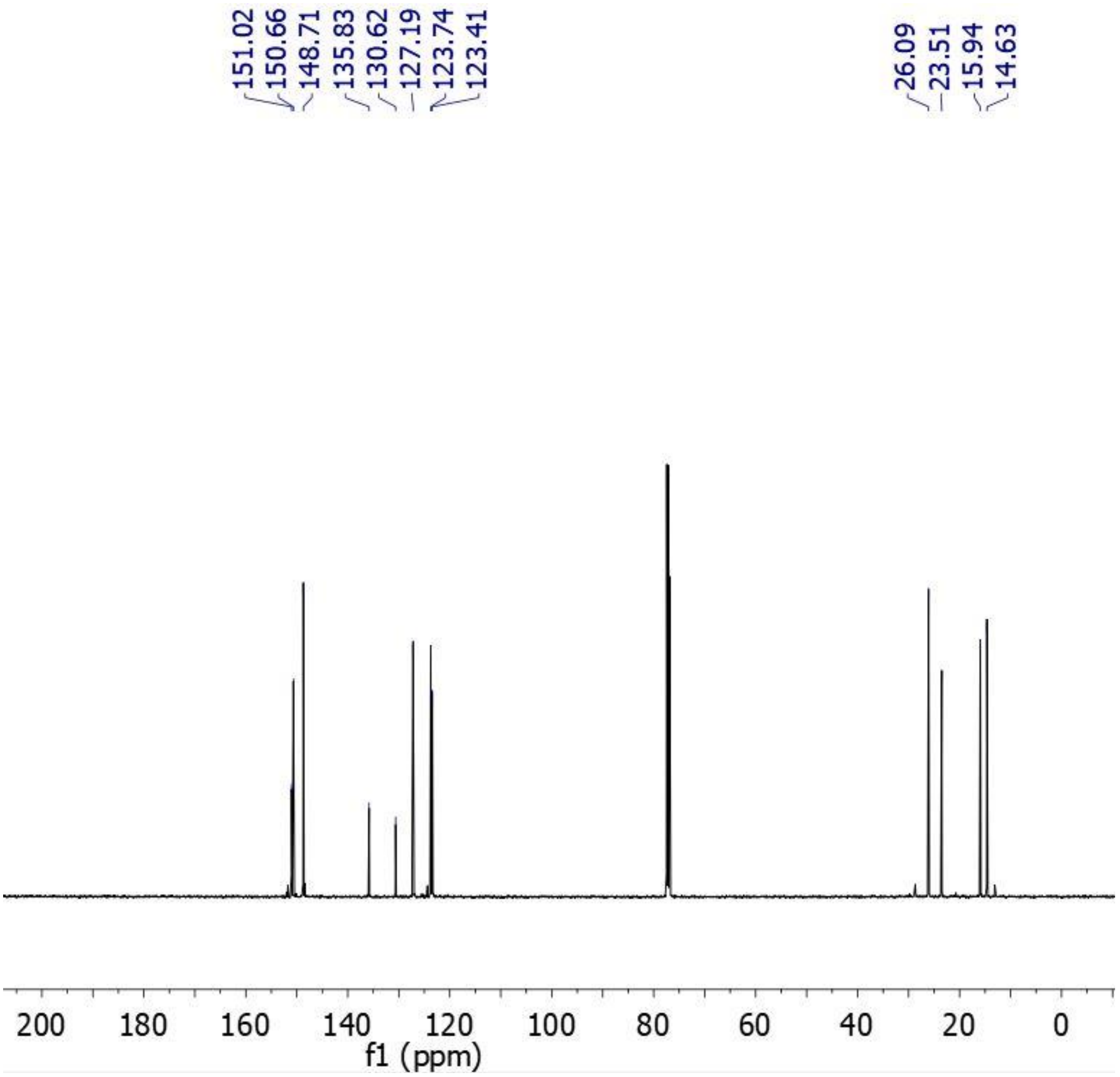

Figure S15: ^{13}C NMR of 4-Ethyl-3-(5’-ethyl-2’-thiopheneyl)pyridine

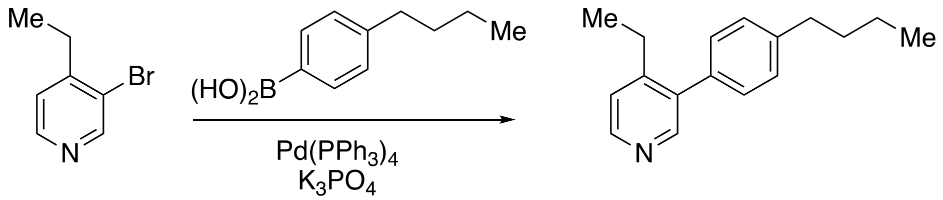

3-(4'-butylphenyl)-4-ethylpyridine A flame-dried 50 mL two-neck round bottom flask under N_2 was charged with 4-butylphenylboronic acid (758 mg, 4.3 mmol), $Pd(PPh_3)_4$ (164 mg, 0.14 mmol) and K_3PO_4 (1.5 g, 7.1 mmol). 3-bromo-4-ethylpyridine (528 mg, 2.8 mmol) was then added dissolved in 12 mL anhydrous toluene, followed by 12 mL of a 2:1 mixture of EtOH:H_2O that had been degassed with argon. The reaction mixture was heated to 75 °C and stirred for 72 hours. After cooling to room temperature, the reaction mixture was quenched with H_2O and extracted with dichloromethane. The combined organic layers were dried over $MgSO_4$ and concentrated *in vacuo*. Column chromatography over silica gel (2:1 EtOAc:hexane) afforded 678 mg (2.8 mmol, 100%) of a golden oil.

^{1}H NMR ($CDCl_3$) δ = 8.46 (d, *J* = 4.8 Hz, 1H), 8.39 (s, 1H), 7.25 – 7.17 (m, 5H), 2.66 (t, *J* = 7.8 Hz, 2H), 2.61 (q, *J* = 7.5 Hz, 2H), 1.64 (p, *J* = 7.7 Hz, 2H), 1.39 (h, *J* = 7.4 Hz, 2H), 1.12 (t, *J* = 7.6 Hz, 3H), 0.95 (t, *J* = 7.5 Hz, 3H). ^{13}C NMR ($CDCl_3$) δ = 150.53, 150.34, 148.51, 142.43, 137.49, 135.24, 129.30, 128.52, 123.28, 35.51, 33.73, 25.65, 22.57, 14.63, 14.13. HRMS m/z calcd. for $C_{17}H_{21}N$ $[M+H]^+$ 240.1747, found 240.1735

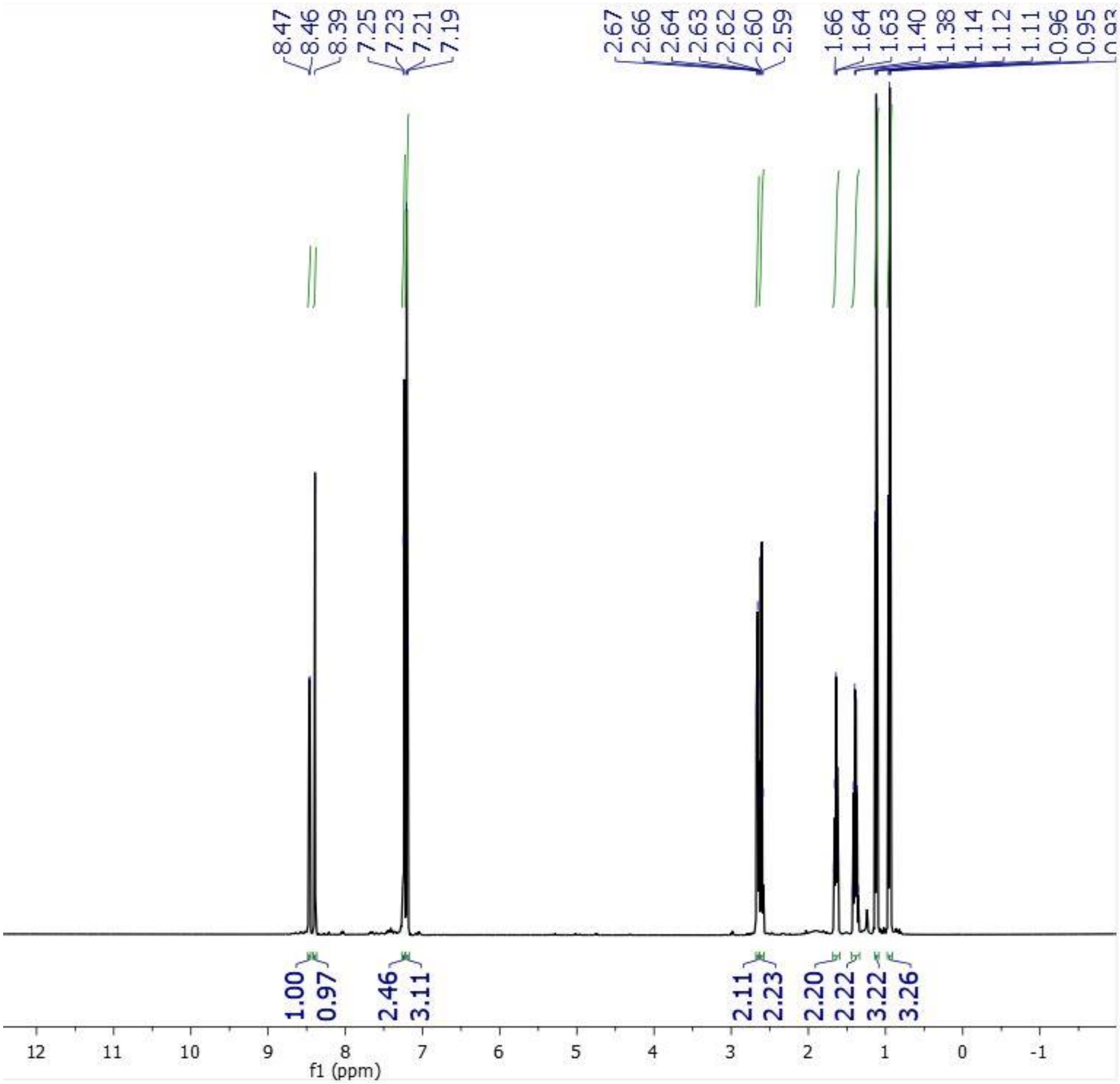

Figure S16: ^{1}H NMR of 3-(4'-butylphenyl)-4-ethylpyridine

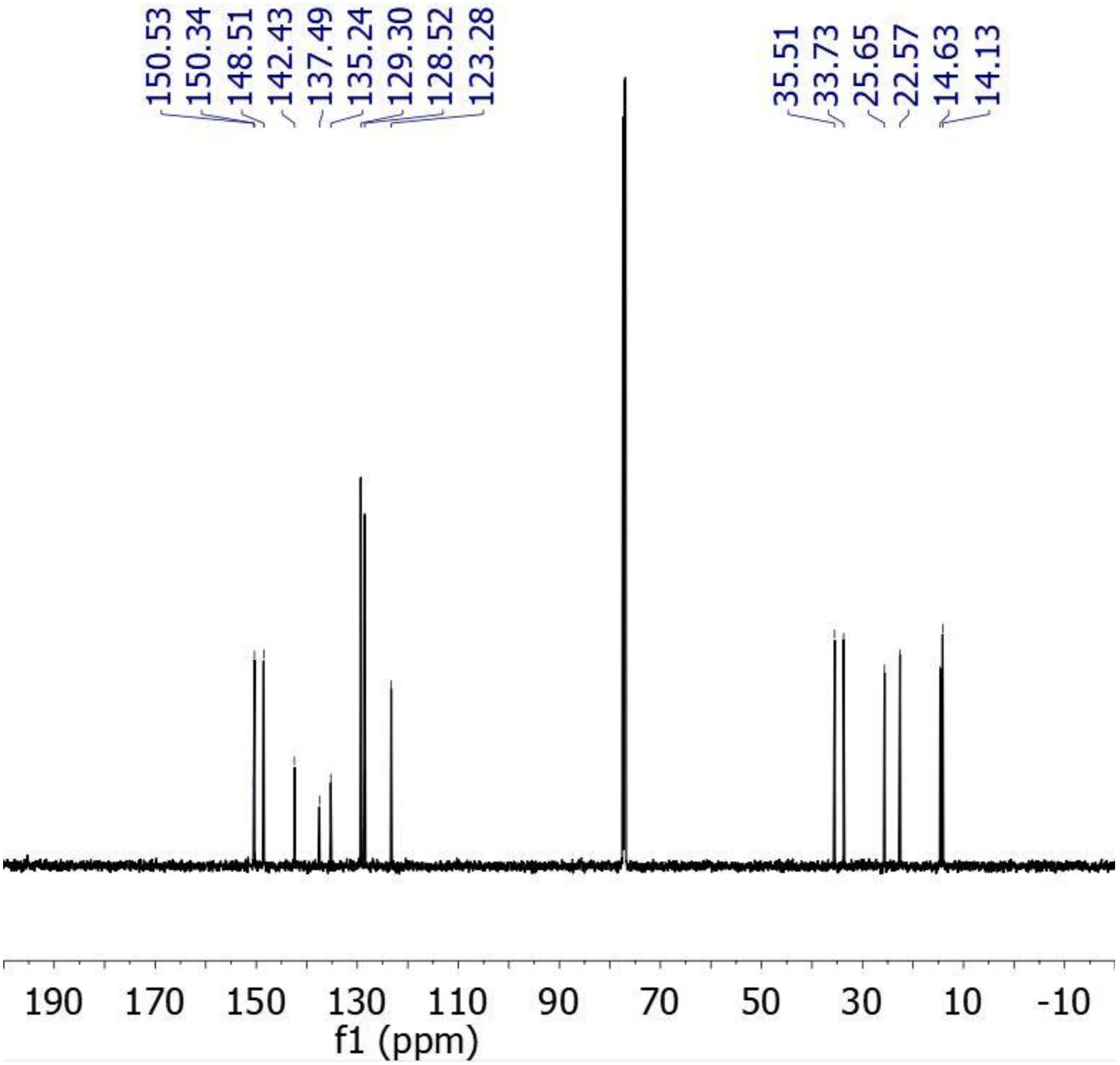

Figure S17: ^{13}C NMR of 3-(4'-butylphenyl)-4-ethylpyridine

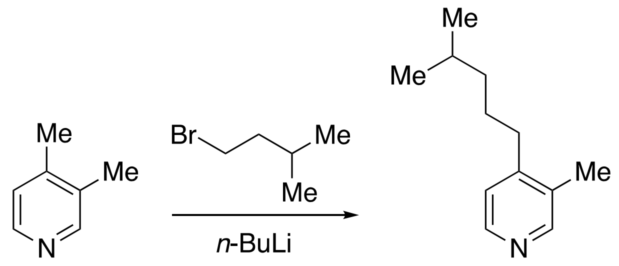

3-methyl-4-(4-methylpentyl)pyridine[10] A flame dried 2-neck flask under N_2 was charged with 3,4-lutidine (500 µL, 4.45 mmol) and 2 mL anhydrous THF. The mixture was cooled to -78 °C and *n*-BuLi (2.5 M in hexanes, 2 mL, 5 mmol) was added dropwise. The solution was then allowed to warm to room temperature for five minutes and then heated to 45 °C for two hours. The reaction was then cooled to 0 °C and 5 mL of anhydrous THF were added to dissolve the resulting orange slurry. The mixture was transferred via cannula to a separate flame dried 2-neck flask under N_2 at -78 °C that was charged with 1-bromo-3-methylbutane (586 µL, 4.9 mmol) and 1 mL anhydrous THF. The reaction mixture was warmed to room temperature and stirred overnight. The reaction mixture was then quenched with 300 µL of water and extracted three times with EtOAc. The combined organic layers were washed with water and brine, dried over $MgSO_4$, and concentrated *in vacuo*. Column chromatography over silica gel (1:1 hexane:EtOAc) afforded 738 mg (4.1 mmol, 93%) of a yellow oil.

^{1}H NMR ($CDCl_3$) δ = 8.34 – 8.25 (m, 2H), 7.01 (d, *J* = 5.0 Hz, 1H), 2.51 (t, *J* = 7.9 Hz, 2H), 2.24 (s, 3H), 1.64 – 1.47 (m, 3H), 1.28 – 1.19 (m, 2H), 0.86 (d, *J* = 6.6 Hz, 6H).

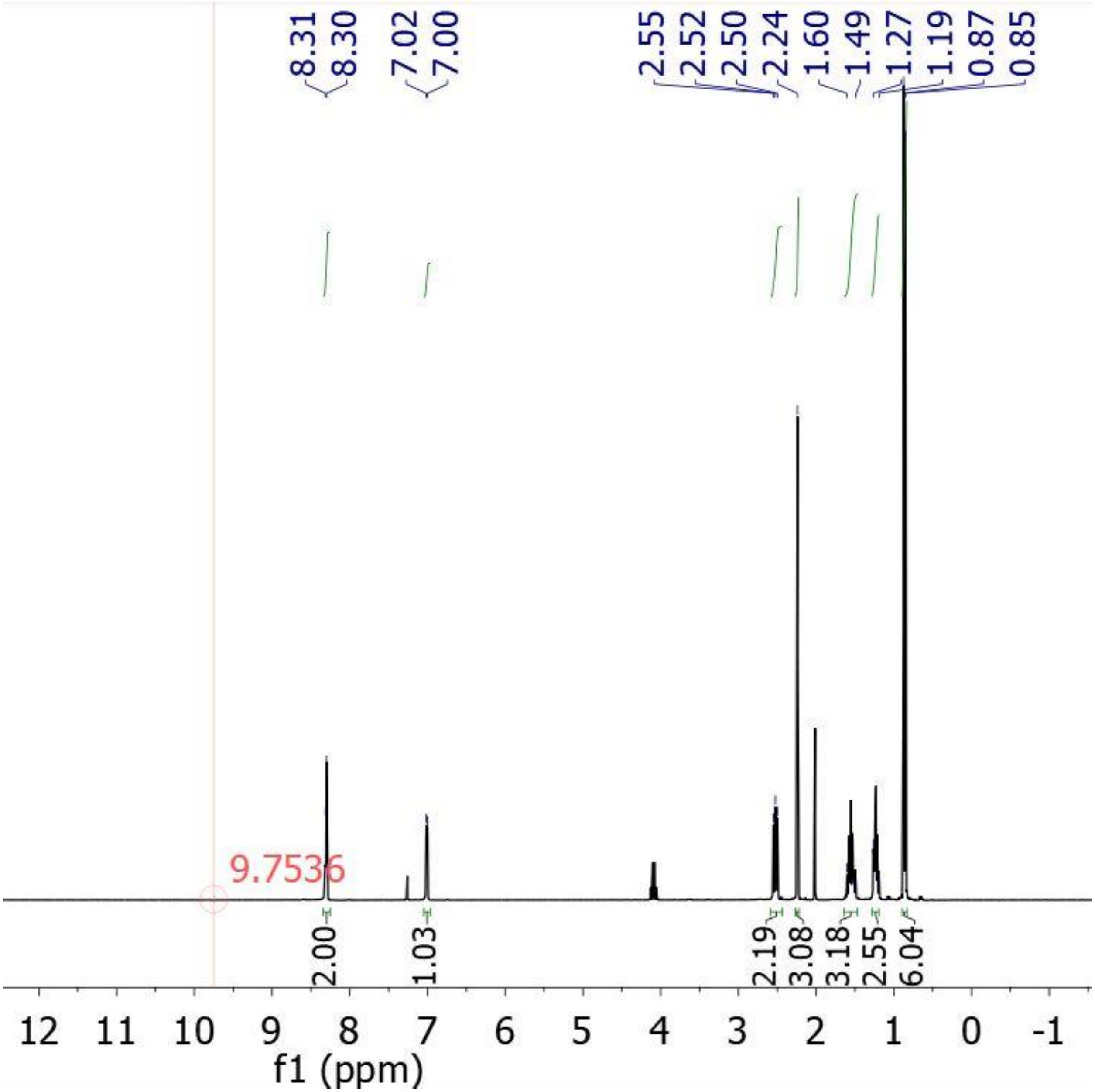

Figure S18: ^{1}H NMR of 3-methyl-4-(4-methylpentyl)pyridine

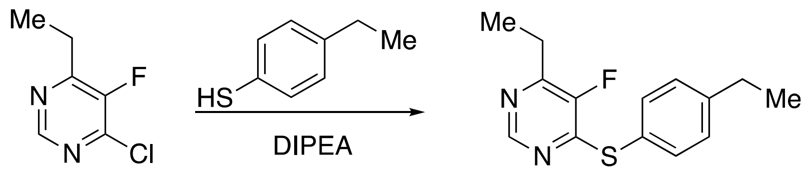

4-ethyl-6-(4'-ethylphenylthioether)-5-fluoropyrimidine An 8-mL vial was charged with 4-chloro-6-ethyl-5-fluoropyrimidine (300 µL, 2.4 mmol), 4-ethylthiophenol (360 µL, 2.64 mmol), and 4 mL ACN. DIPEA (420 µL, 2.4 mmol) was then added dropwise. The vial was left to stir at room temperature for two hours. The reaction was quenched with 2 M aq. NaOH and extracted with dichloromethane, dried over $MgSO_4$ and concentrated *in vacuo*. Column chromatography over silica gel (10:1 hexane:EtOAc) afforded 581 mg (2.2 mmol, 93%) of a pale yellow oil.

^{1}H NMR ($CDCl_3$) δ = 8.55 (d, *J* = 2.3 Hz, 1H), 7.52 – 7.43 (m, 2H), 7.30 (d, *J* = 8.1 Hz, 2H), 2.82 (qd, *J* = 7.6, 2.2 Hz, 2H), 2.71 (q, *J* = 7.6 Hz, 2H), 1.31 (t, *J* = 7.6 Hz, 3H), 1.28 (t, *J* = 7.6 Hz, 3H). ^{13}C NMR ($CDCl_3$) δ = 157.36 (d, *J* = 15.6 Hz), 155.82 (d, *J* = 14.4 Hz), 153.62 (d, *J* = 9.1 Hz), 151.41, 146.41, 135.87, 129.22, 122.89, 28.81, 24.13, 15.26, 11.90. HRMS m/z calcd. for $C_{14}H_{15}FN_2S$ $[M+H]^+$ 263.1015, found 263.1007

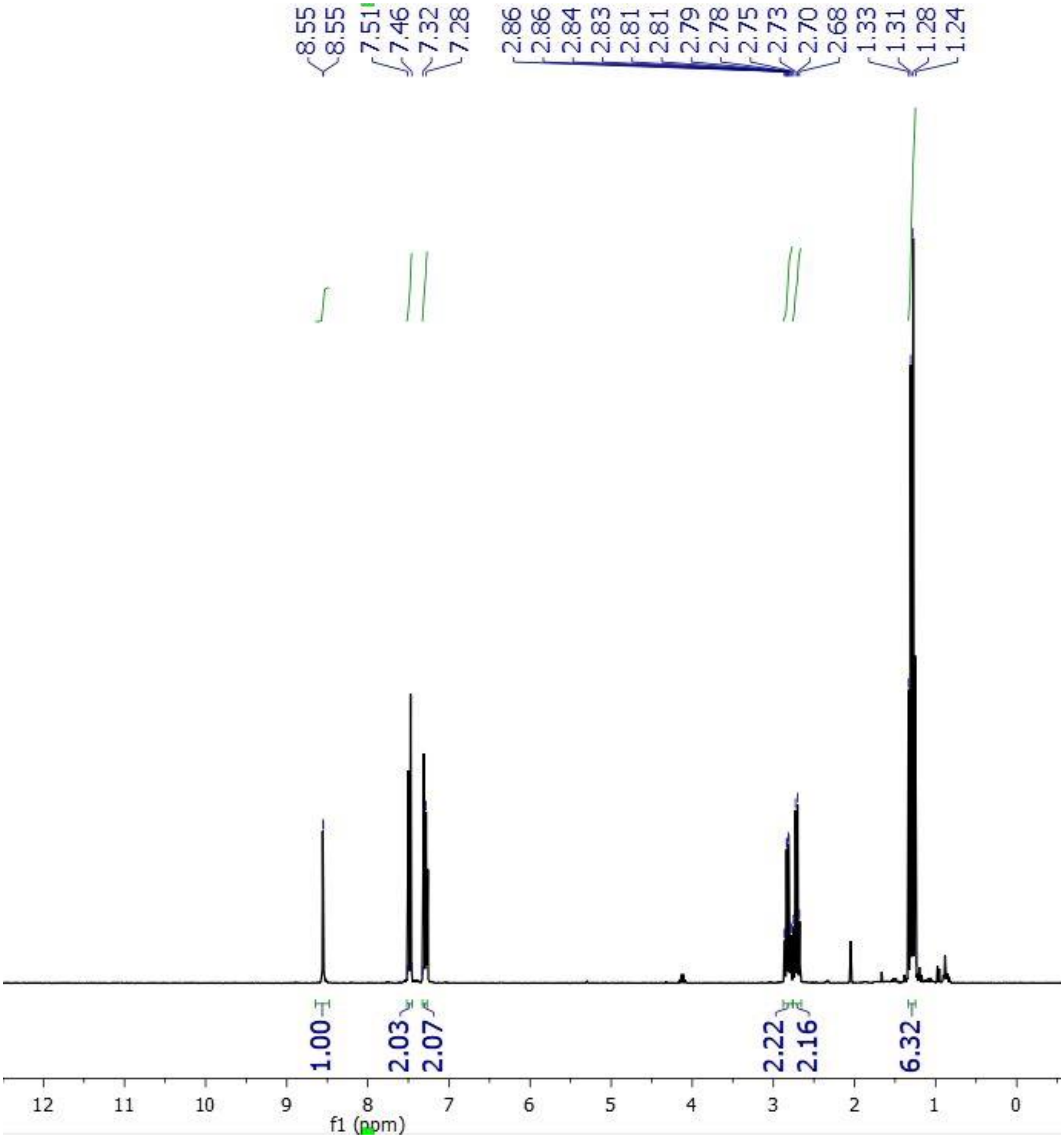

Figure S19: ^{1}H NMR of 4-ethyl-6-(4’-ethylphenylthioether)-5-fluoropyrimidine

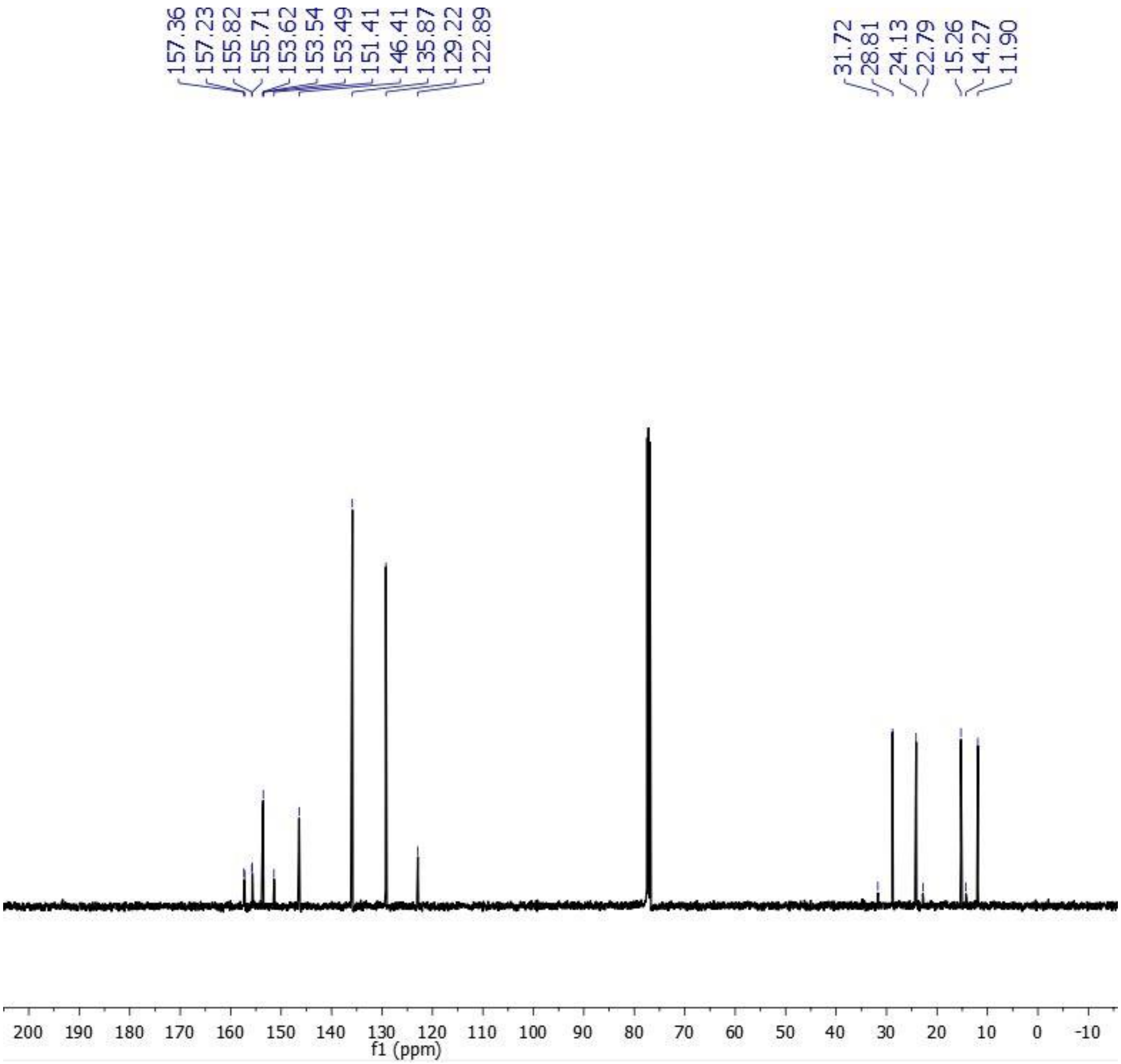

Figure S20: ^{13}C NMR of 4-ethyl-6-(4'-ethylphenylthioether)-5-fluoropyrimidine

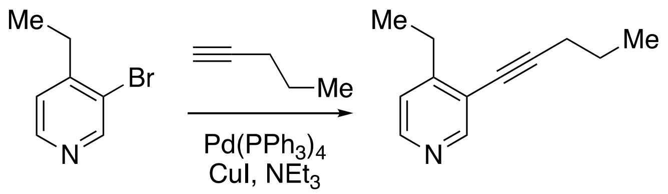

4-ethyl-3-(1'-pentynyl)pyridine An oven-dried 20-mL vial was charged with $Pd(PPh_3)_4$ (231 mg, 0.2 mmol) and CuI (38 mg, 0.2 mmol). The vial was evacuated and then refilled with nitrogen 3 times, then 3-bromo-4-ethylpyridine (372 mg, 2 mmol) dissolved in 5.6 mL NEt_3 (20 eq) was added, followed up 1-pentyne (395 µL, 4 mmol). The puncturable vial cap was replaced with a solid cap and the reaction was heated to 80 °C for 36 hours. The reaction was quenched with 1 mL of MeOH, concentrated *in vacuo*, filtered through celite using dichloromethane, washed with $NaHCO_3$ and brine, dried with $MgSO_4$ and concentrated *in vacuo*. Column chromatography over silica gel (10:1 DCM:EtOAc) afforded 202 mg (1.2 mmol, 58%) of a light yellow oil.

^{1}H NMR ($CDCl_3$) δ = 8.54 (s, 1H), 8.37 (d, *J* = 5.2 Hz, 1H), 7.10 (d, *J* = 5.2 Hz, 1H), 2.77 (q, *J* = 7.6 Hz, 2H), 2.45 (t, *J* = 6.9 Hz, 2H), 1.66 (h, *J* = 7.3 Hz, 2H), 1.24 (t, *J* = 7.6 Hz, 3H), 1.07 (t, *J* = 7.2 Hz, 3H). ^{13}C NMR ($CDCl_3$) δ = 154.25, 152.61, 148.09, 122.46, 120.71, 97.16, 76.22, 27.09, 22.25, 21.66, 13.66, 13.64. HRMS m/z calcd. for $C_{12}H_{15}N$ $[M+H]^+$ 174.1277, found 174.1276.

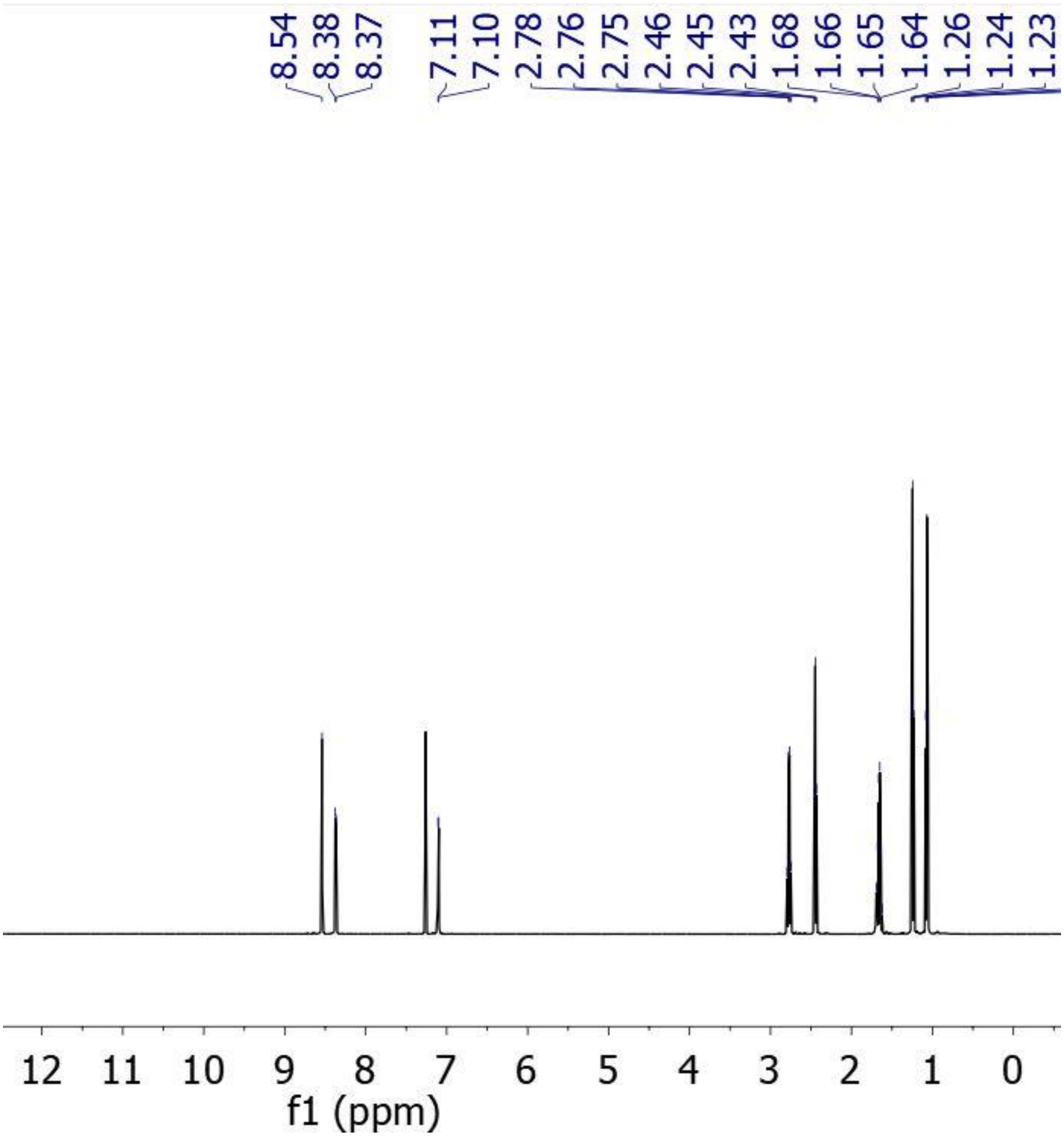

Figure S21: ^{1}H NMR of 4-ethyl-3-(1'-pentynyl)pyridine

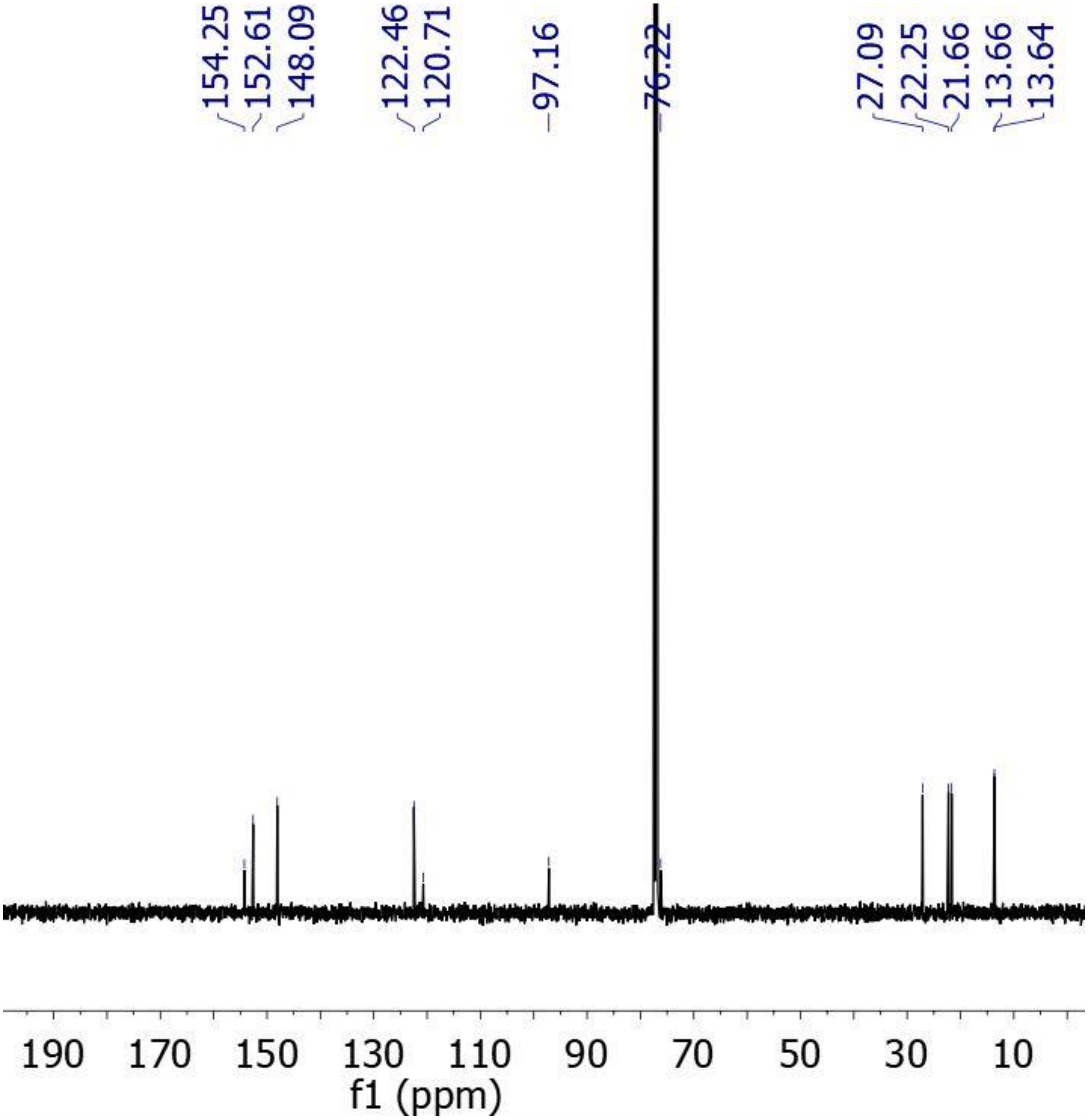

Figure S22: ^{13}C NMR of 4-ethyl-3-(1'-pentynyl)pyridine

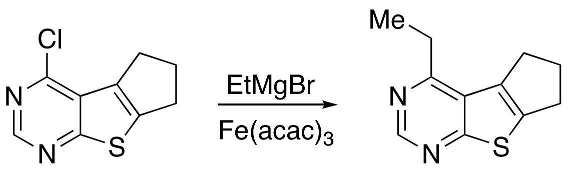

4-Ethyl-2,3-dihyrdo-1H-8-thia-5,7-diazacyclopenta[α]indene A flame dried 2-neck flask under N_2 was charged with 4-chloro-2,3-dihyrdo-1H-8-thia-5,7-diazacyclopenta[α]indene (500 mg, 2.37 mmol), $Fe(acac)_3$ (41.9 mg, 0.12 mmol), 18 mL anhydrous THF, and 2 mL anhydrous NMP. The reaction mixture was cooled to 0 °C and EtMgBr (3M in diethyl ether, 0.95 mL, 2.85 mmol) was added dropwise. The reaction mixture was warmed to room temparture and stirred for 2 hours. The reaction was quenched with aqueous NH_4Cl and extracted with dichloromethane. The combined organic layers were dried over $MgSO_4$ and concentrated *in vacuo*. Column chromatography over silica gel (2:1 hexane:EtOAc) afforded 322 mg (1.57 mmol, 66%) of a colorless oil.

^{1}H NMR ($CDCl_3$) δ = 8.88 (s, 1H), 3.17-3.02 (m, 6H), 2.56 (p, *J* = 7.4 Hz, 2H), 1.39 (t, *J* = 7.6 Hz, 3H). ^{13}C NMR ($CDCl_3$) δ = 173.29, 164.70, 152.25, 142.64, 136.04, 126.12, 29.97, 28.92, 27.56, 13.56. HRMS m/z calcd. for $C_{11}H_{12}N_2S$ $[M+H]^+$ 205.0794, found 205.0791.

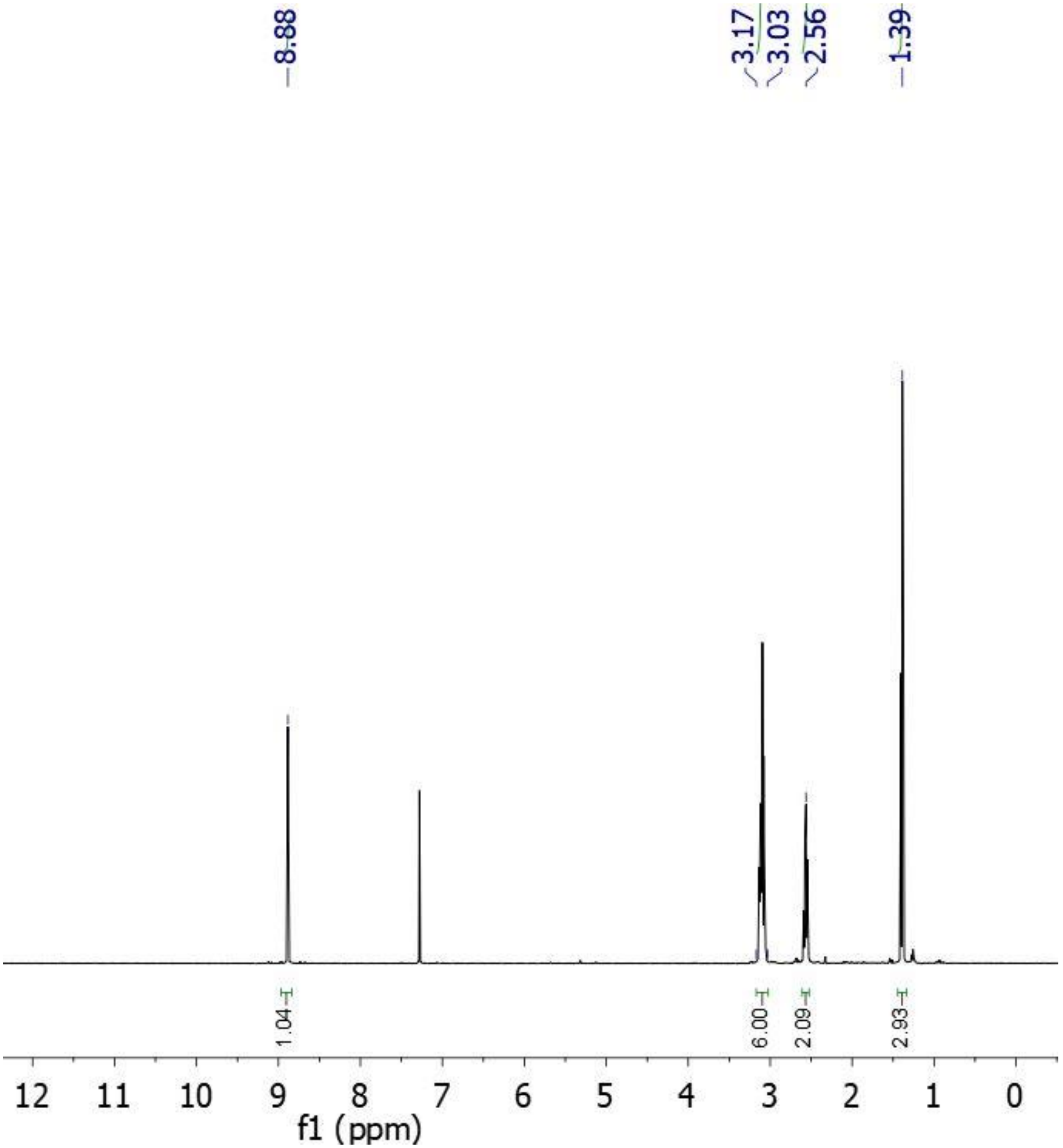

Figure S23: ^{1}H NMR of 4-Ethyl-2,3-dihyrdo-1H-8-thia-5,7-diazacyclopenta[α]indene

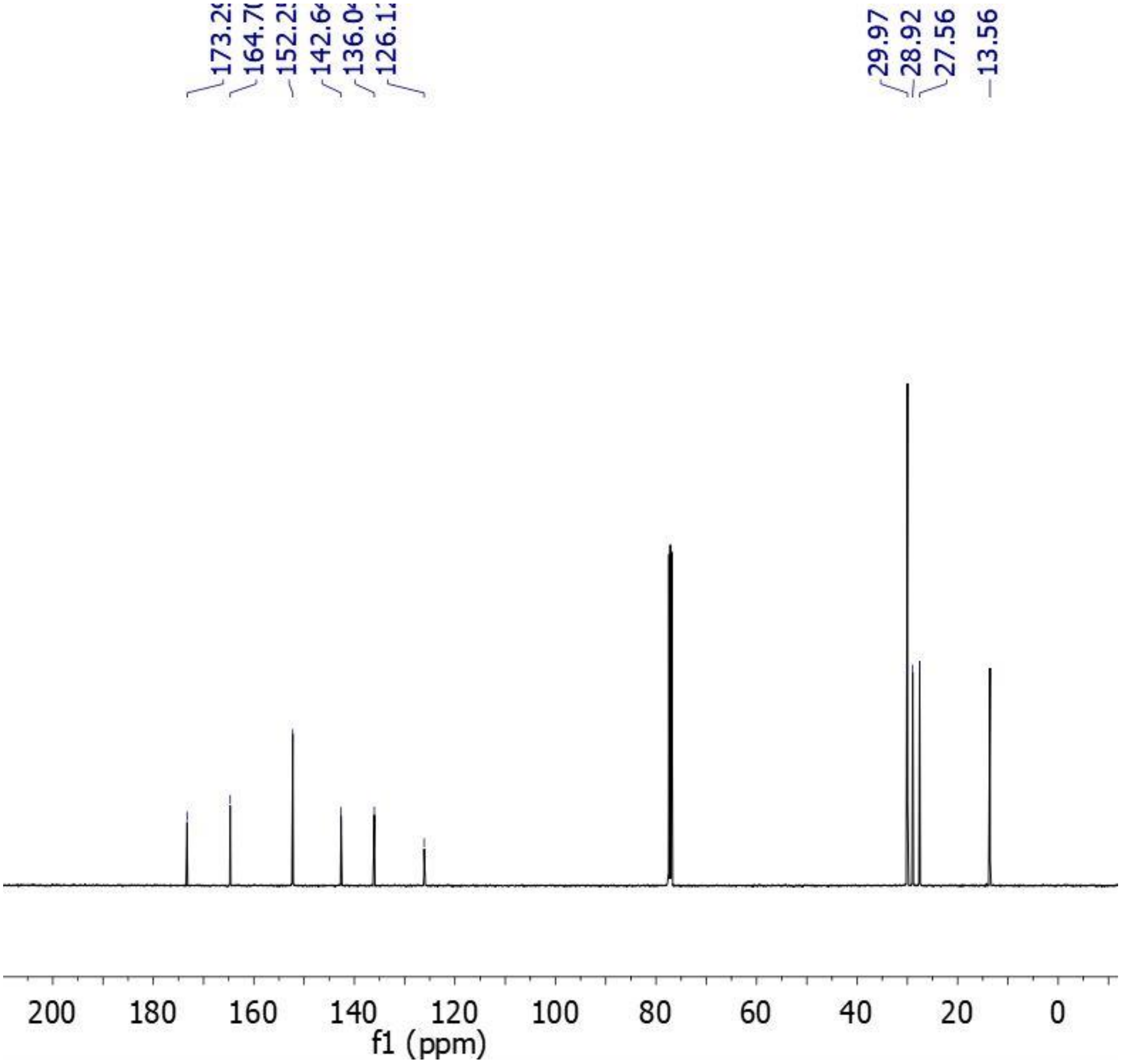

Figure S24: ^{13}C NMR of 4-Ethyl-2,3-dihyrdo-1H-8-thia-5,7-diazacyclopenta[α]indene

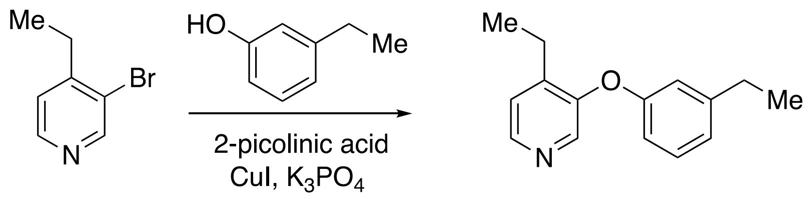

4-Ethyl-3-(3'-ethylphenylether)pyridine A flame dried 2-neck flask under N_2 was charged with 3-bromo-4-ethylpyridine (400 mg, 2.15 mmol), 3-ethylphenol (311 μL, 2.58 mmol), CuI (20.4 mg, 0.11 mmol), 2-picolinic acid (26.4 mg, 0.22 mmol), K_3PO_4 (910 mg, 4.29 mmol) and 12 mL anhydrous DMSO. The reaction mixture was heated to 80 °C and stirred overnight. The reaction mixture was cooled to room temperature, quenched with aqueous $NaHCO_3$ and extracted with dichloromethane. The combined organic layers were dried over $MgSO_4$ and concentrated *in vacuo*. Column chromatography over silica gel (3:1 hexane:EtOAc) afforded 360 mg (1.58 mmol, 74%) of a colorless oil.

^{1}H NMR ($CDCl_3$) δ = 8.32 (d, *J* = 5.0 Hz, 1H), 8.19 (s, 1H), 7.24 – 7.20 (m, 2H), 6.93 (d, *J* = 7.6 Hz, 1H), 6.79 (s, 1H), 6.72 (dd, *J* = 8.1, 2.5 Hz, 1H), 2.67 (q, *J* = 7.5 Hz, 3H), 2.62 (q, *J* = 7.7 Hz, 2H), 1.24 – 1.19 (m, 6H). ^{13}C NMR ($CDCl_3$) δ = 157.67, 151.41, 146.65, 145.35, 144.41, 141.89, 129.75, 124.20, 122.82, 116.95, 114.54, 28.88, 22.64, 15.53, 13.41. HRMS m/z calcd. for $C_{15}H_{17}NO$ $[M+H]^+$ 228.1383, found 228.1387.

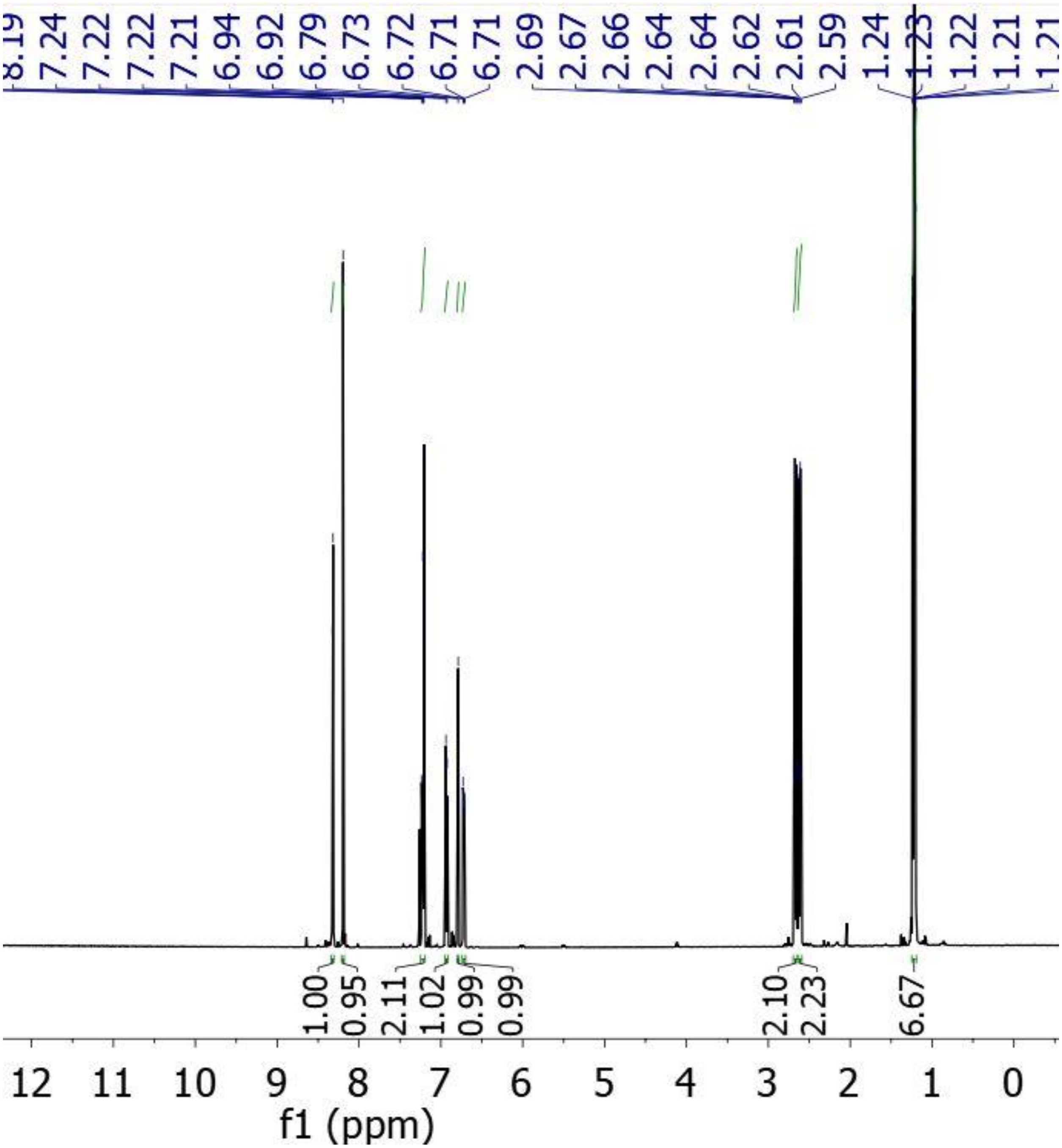

Figure S25: ^{1}H NMR of 4-Ethyl-3-(3'-ethylphenylether)pyridine

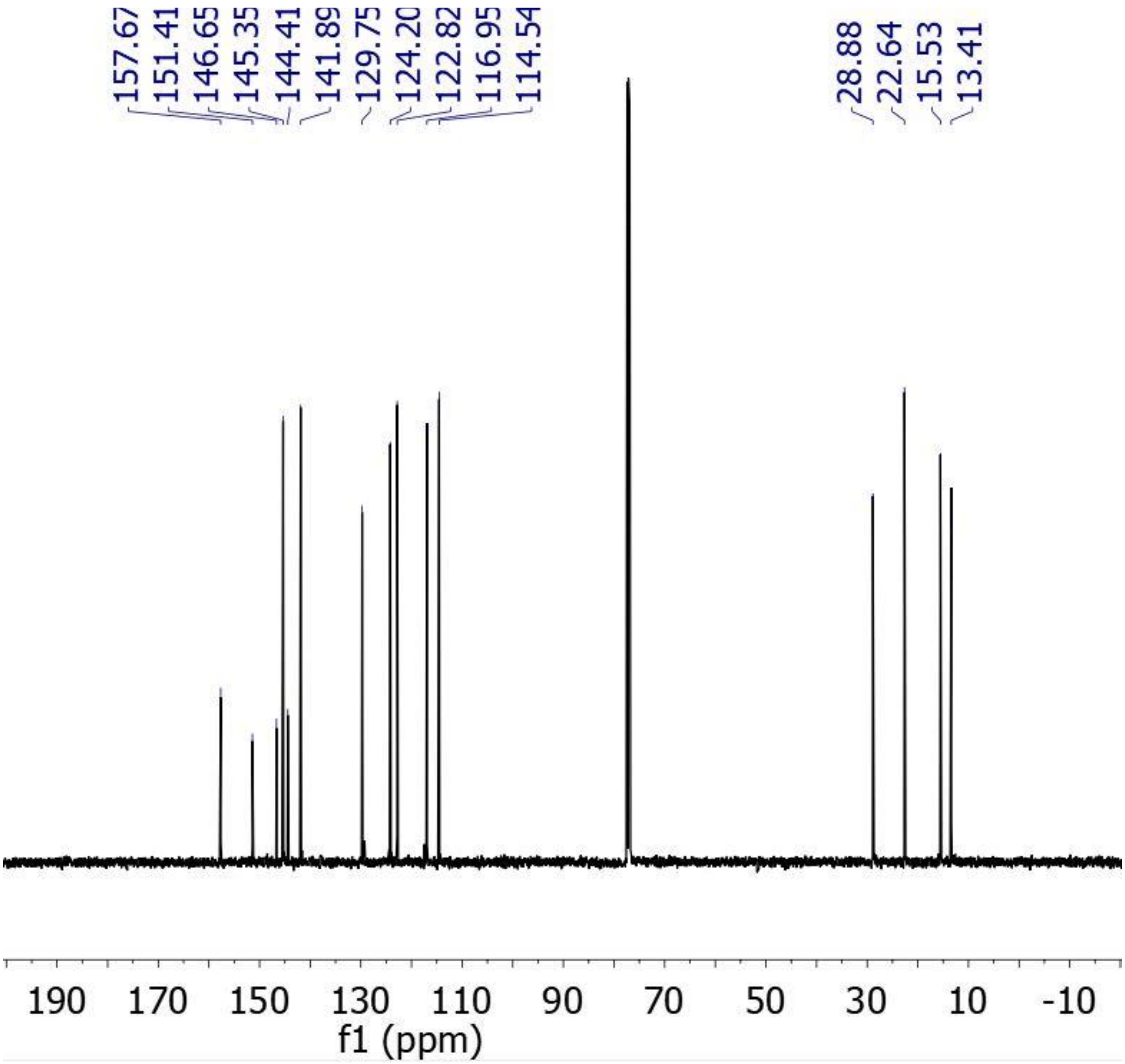

Figure S26: ^{13}C NMR of 4-Ethyl-3-(3’-ethylphenylether)pyridine

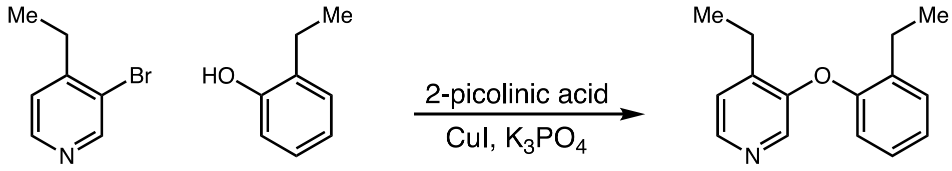

4-Ethyl-3-(2'-ethylphenylether)pyridine: A flame dried 2-neck flask under N_2 was charged with 3-bromo-4-ethylpyridine (400 mg, 2.15 mmol), 2-ethylphenol (300 μL, 2.55 mmol), CuI (40.8 mg, 0.22 mmol), 2-picolinic acid (52.8 mg, 0.44 mmol), K_3PO_4 (910 mg, 4.29 mmol) and 12 mL anhydrous DMSO. The reaction mixture was heated to 90 °C and stirred overnight. The reaction mixture was cooled to room temperature, quenched with aqueous $NaHCO_3$ and extracted with ethyl acetate. The combined organic layers were dried over $MgSO_4$ and concentrated *in vacuo*. Column chromatography over silica gel (gradient elution, 4:1 to 3:1 hexane:EtOAc) afforded 115 mg (0.51 mmol, 24%) of a colorless oil. ^{1}H NMR (400 MHz, Chloroform-*d*) ∂ 8.29 (d, *J* = 4.9 Hz, 1H), 8.05 (s, 1H), 7.30 (dd, *J* = 7.4, 1.9 Hz, 1H), 7.22 (d, *J* = 4.9 Hz, 1H), 7.11 (dtd, *J* = 22.7, 7.5, 1.5 Hz, 2H), 6.71 (dd, *J* = 7.9, 1.3 Hz, 1H), 2.72 (coincident q, *J* = 7.6 Hz, 4H), 1.27 (nearly coincident t, *J* = 7.6, 6H). ^{13}C NMR (101 MHz, $CDCl_3$) ∂ 154.56, 151.94, 144.66, 143.35, 140.03, 134.58, 129.92, 127.18, 124.01, 123.76, 117.24, 23.31, 22.63, 14.48, 13.36. HRMS m/z calcd. for $C_{15}H_{17}NO$ $[M+H]^+$ 228.1383, found 228.1383.

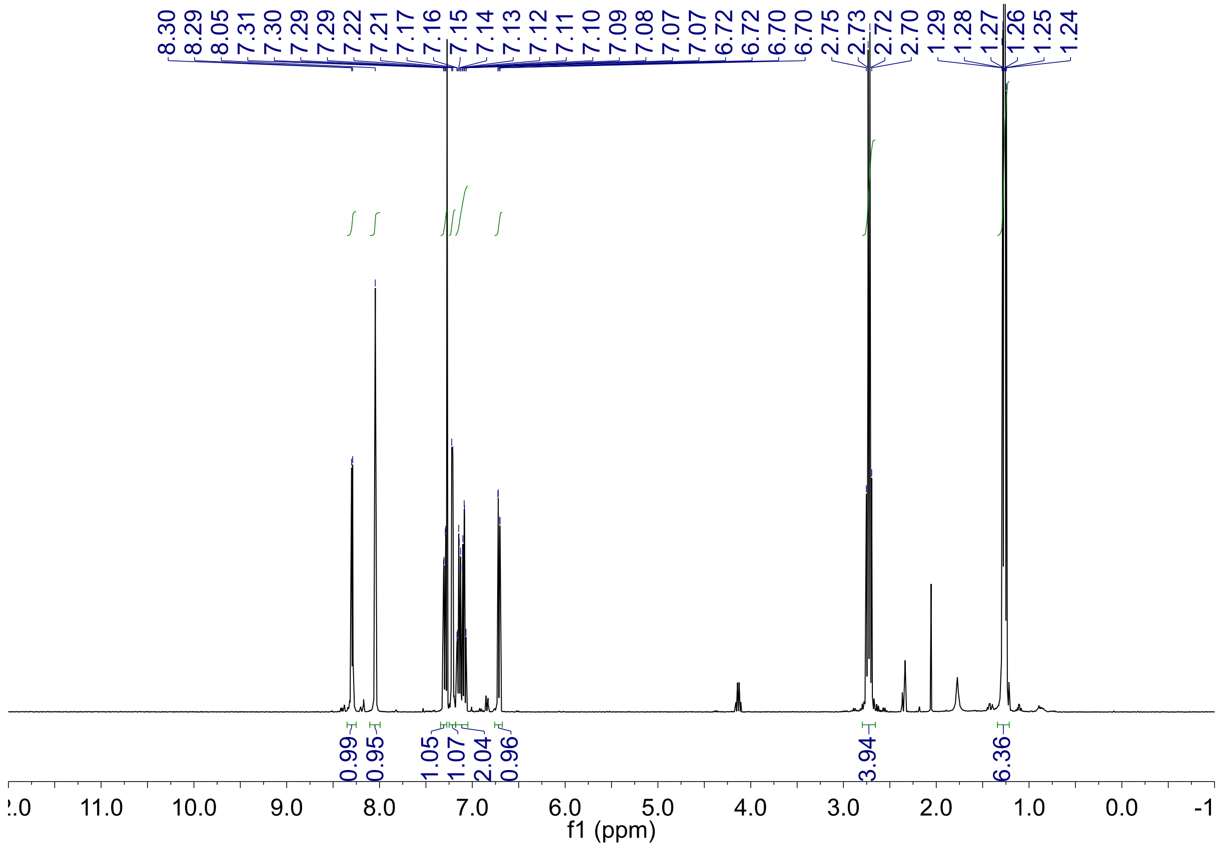

Figure S27: ^{1}H NMR spectrum of 4-Ethyl-3-(2'-ethylphenylether)pyridine

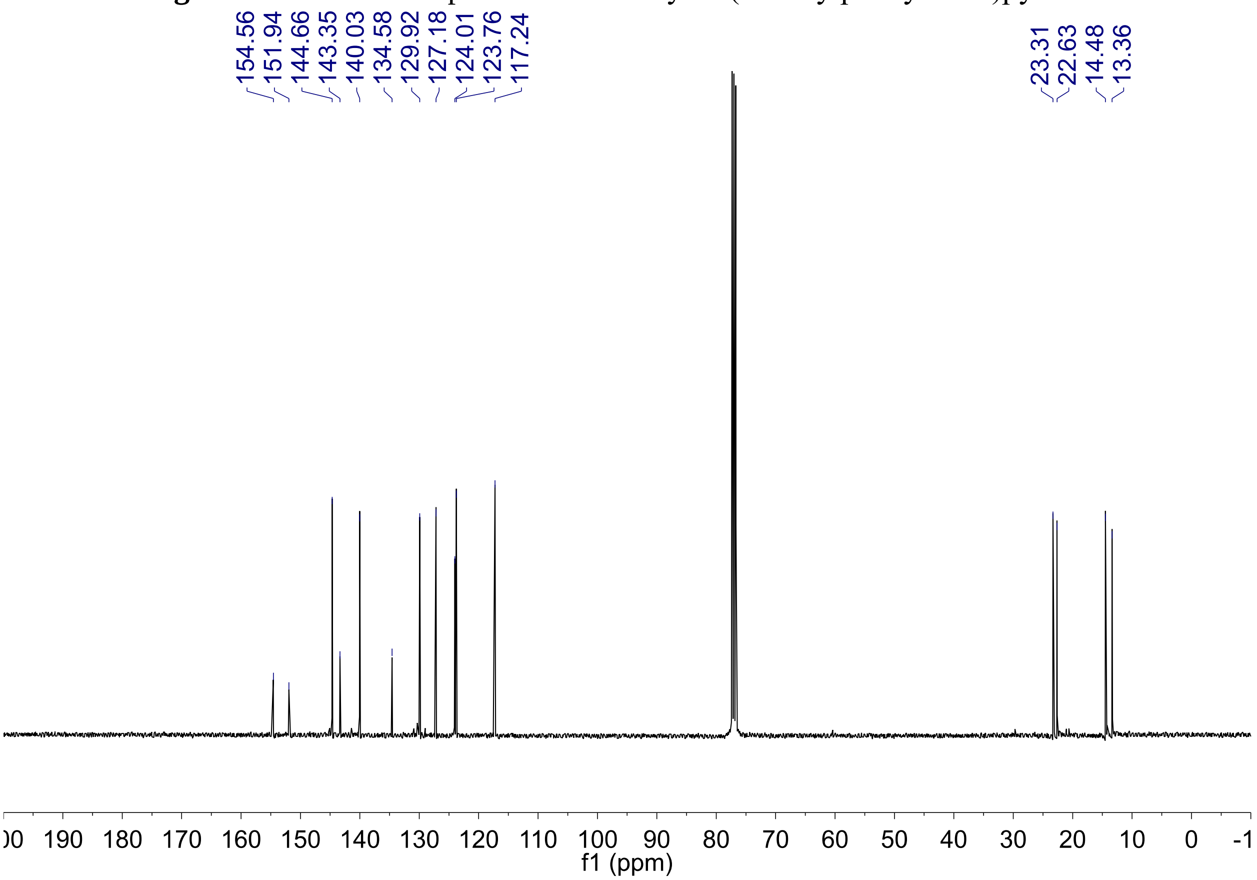

Figure S28: ^{13}C NMR spectrum of 4-Ethyl-3-(2'-ethylphenylether)pyridine

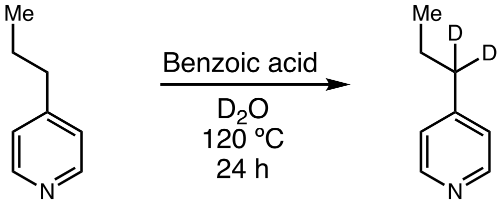

Deuteration of 4-propylpyridine [4-(propyl-1,1-*d*$_2$)pyridine]: adapted from the literature procedure of Yin.[11] 4-Propylpyridine (1 mL, 7.67 mmol) was suspended in 10 mL degassed D_2O in a pressure vessel. Benzoic acid (200 mg, 1.64 mmol) was added, the vessel was sealed, and was heated to 120 ºC for 24 h. After cooling to room temperature, the contents were transferred to a separation funnel with the aid of ethyl acetate. The organic layer was washed with 10% K_2CO_3, dried over $MgSO_4$, filtered and concentrated to yield 4-propylpyridine (875 mg) that had approximately 92% deuterium incorporation at the benzylic position as judged by quantitative ^{1}H NMR integration (0.16H integration when 2.00H was expected).

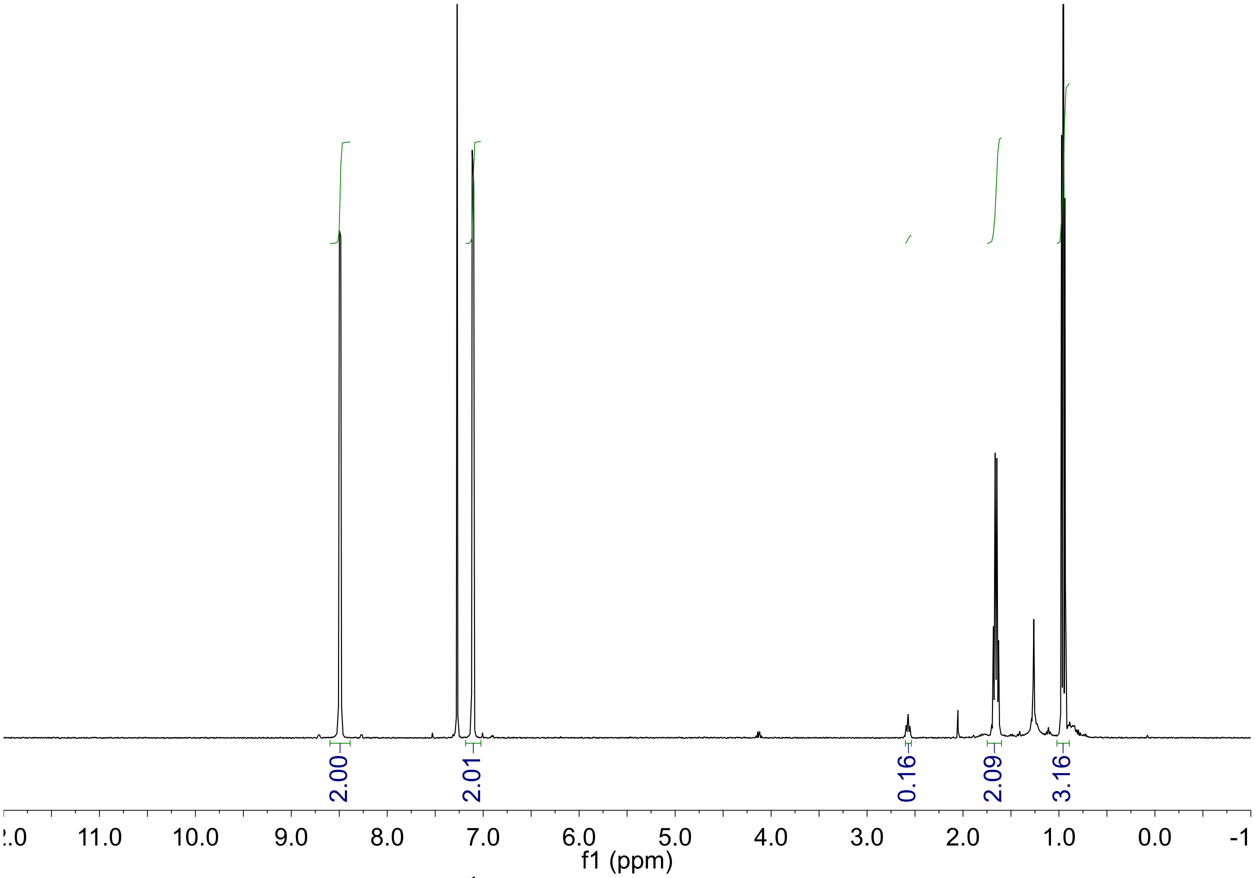

Figure S29: ^{1}H NMR of 4-(propyl-1,1-d_2)pyridine

3. Initial metal and organic catalyst screening experiments

A 2-dram vial with a puncturable cap and stir bar was oven-dried and cooled to room temperature under purging N_2. The heterocycle was added to the vial under air (0.5 mmol of 2-propylthiazole, 6-chloro-5-fluoro-4-ethylpyrimidine, or 4-propylpyridine), followed by the organic catalyst (0.05 mmol of diethyl hydrazinedicarboxylate, *N*-hydroxyphthalimide, or 1-hydroxy-7-azabenzotriazole), the metal triflate salt (0.05 mmol of nickel(II), iron(II), zinc(II), copper(II), or scandium(III)), and internal standard (1,3,5-tri-tert-butylbenzene, 12.3 mg, 10 mol%). Control experiments that lacked either the organic catalyst, the metal triflate salt, or both, were also prepared. The vials were sealed with Teflon tape on their threads and purged with N_2 for ~5 minutes. Anhydrous ACN (1 mL) was added, followed by DEAD (99 μL, 0.625 mmol). The vial was sealed with electrical tape and the septa sealed with melted parafilm. The reaction mixture was heated to 80 °C and stirred for 24 hrs. After cooling to room temperature, 100 µL aliquots were injected into a quenching solution that was made with 1 mL ethyl acetate and 1 mL sodium ascorbate solution (1 M). After shaking briefly and allowing the layers to separate, 0.5 mL of the ethyl acetate layer was injected on top of a one inch deep layer of dry silica held in a Pasteur pipette. The quenched reaction mixture was filtered through the silica directly into a 2 mL gas chromatography vial with the aid of 1.8 mL pure ethanol, and was analyzed by GC relative to internal standard. All products were calibrated with conversion factors as described below. The results from these experiments were used to construct Table 1 in the text, as well as

to select $Sc(OTf)_3$ for mechanistic comparisons, given the results observed for reaction with 4-propylpyridine shown below.

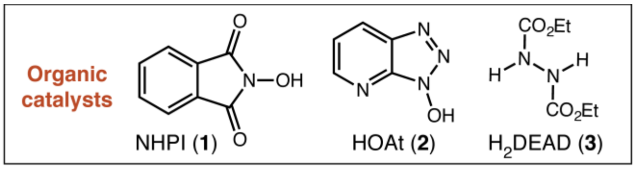

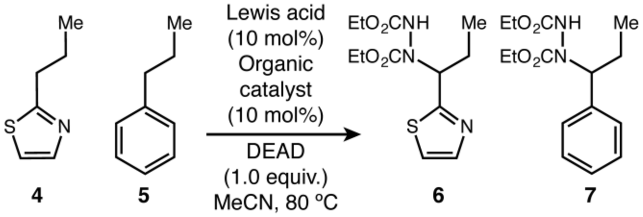

Lewis acid	Organic catalyst (yield, selectivity as 6:7 ratio)			
	none	NHPI (1)	HOAt (2)	H$_2$DEAD (3)
None	N/D	8% (1:6)	7% (1.2:1)	N/D
Ni(OTf)$_2$	3% (N/A)[b]	3% (N/A)[b]	10% (30:1)	3% (N/A)[b]
Zn(OTf)$_2$	N/D	N/D	3% (N/A)[b]	N/D
Fe(OTf)$_2$	6% (>20:1)[c]	5% (1:1)	13% (>99:1)	6% (>20:1)
Sc(OTf)$_3$	7% (>20:1)[c]	7% (1:2.5)	13% (2.2:1)	8% (>20:1)
Cu(OTf)$_2$	3% (N/A)[b]	5% (1:5)	49% (>99:1)	3% (N/A)[b]

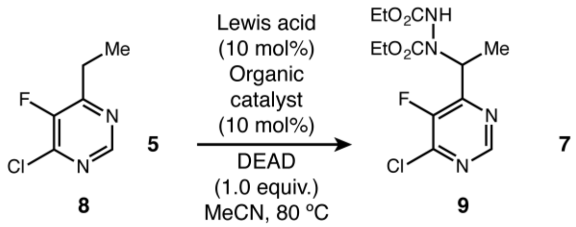

Lewis acid	Organic catalyst (yield, selectivity as 9:7 ratio)			
	none	NHPI (1)	HOAt (2)	H$_2$DEAD (3)
None	N/D	N/D	N/D	N/D
Ni(OTf)$_2$	3% (N/A)[b]	13% (1:4)	14% (8.6:1)	5% (>20:1)[c]
Zn(OTf)$_2$	N/D	19% (1:1.3)	3% (N/A)[b]	N/D
Fe(OTf)$_2$	N/D	12% (1:2.6)	14% (>99:1)	5% (>20:1)[c]
Sc(OTf)$_3$	N/D	4% (N/A)[b]	6% (3.5:1)	<1% (N/A)[b]
Cu(OTf)$_2$	78% (>99:1)	64% (4.9:1)	37% (>99:1)	80% (>99:1)

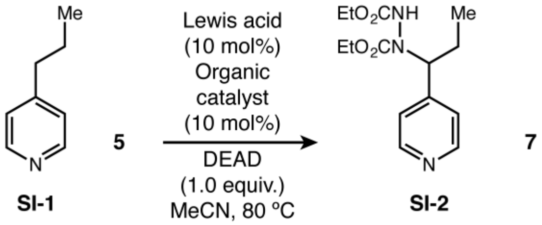

Lewis acid	Organic catalyst (yield, selectivity as SI-2:7 ratio)			
	none	NHPI (1)	HOAt (2)	H$_2$DEAD (3)
None	1% (N/A)[b]	N/D	N/D	N/D
Ni(OTf)$_2$	1% (N/A)[b]	7% (1:1)	22% (45:1)	1% (N/A)[b]
Zn(OTf)$_2$	42% (>99:1)	20% (>99:1)	33% (>99:1)	43% (>99:1)
Fe(OTf)$_2$	53% (>99:1)	47% (87:1)	47% (>99:1)	57% (>99:1)
Sc(OTf)$_3$	99% (>99:1)	54% (19:1)	96% (88:1)	96% (>99:1)
Cu(OTf)$_2$	65% (>99:1)	59% (49:1)	63% (>99:1)	73% (>99:1)

Table S1. Initial screening experiments varying organic catalyst and metal triflate structure.

All yields and selectivities determined by gas chromatographic analysis were calibrated by a unique conversion factor for each substrate. The conversion factor was determined by GC-FID and ^{1}H NMR against 1,3,5-triisopropylbenzene as a standard. A typical procedure for determining the conversion factor is described for compound **10**. A mixture of **10** (17.7 mg, 0.05 mmol) and 1,3,5-tri-*tert*-butylbenzene (12.3 mg, 0.05 mmol) were dissolved in 1 mL $CDCl_3$. ^{1}H NMR was collected using a 10 s relaxation delay. The integrations of the methyl protons of the standard (δ = 1.31) were compared to the methine proton of **10** (δ = 5.44). The spectra were baseline corrected (Bernstein polynomials) and phase corrected. The sample was then transferred to a GC vial, diluted, and analyzed by GC-FID. The integrations of the standard peak and the product peak were compared, and the corresponding ratio was corrected to match the ratio calculated by NMR using Equation 1, where CF = the conversion factor.

$$\frac{A[\mathbf{8}]_{NMR}}{A[std]_{NMR}} = CF * \frac{A[\mathbf{8}]_{GC}}{A[std]_{GC}}$$

Equation 1

4. Amination of *N*-heterocycles with diethyl azodicarboxylate (DEAD)

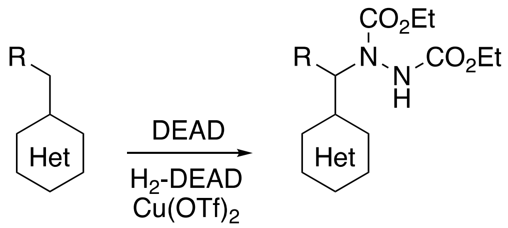

Yield Procedure A: A 2-dram vial with a puncturable cap and stir bar was oven-dried and cooled to room temperature under purging N_2. The heterocycle was added to the vial under air (0.5 mmol), followed by H_2-DEAD (4.4 mg, 0.025 mmol) and $Cu(OTf)_2$ (18.1 mg, 0.05 mmol). The vial was sealed and purged with N_2 for ~5 minutes. Anhydrous ACN (1 mL) was added, followed by DEAD (99 µL, 0.625 mmol). The vial was sealed with electrical tape and the septa sealed with melted parafilm. The reaction mixture was heated to 85 °C and stirred for 24 hrs. After cooling to room temperature, the reaction was quenched with EtOAc and washed with 1M aqueous sodium ascorbate. The organic layer was dried over $MgSO_4$ and concentrated *in vacuo*. All products were purified by column chromatography over silica gel.

Yield Procedure B: Additional DEAD was shown to increase the yield for select substrates. The procedure was carried out as described in **A** with 2 equivalents DEAD used (157 µL, 1.0 mmol).

Yield Procedure C: Exactly 1 equivalent DEAD was used for select substrates to mitigate lower yields due to overreaction. The procedure was carried out as described in **A** with 1 equivalent DEAD used (78 µL, 0.5 mmol).

Yield Procedure D: For select substrates, 1-hydroxy-7-azabenzotriazole (HOAt) was used instead of H_2-DEAD as the radical initiator. The procedure was carried out as described in **A** with 1 equivalent DEAD used (78 µL, 0.5 mmol).

$Cu(OTf)_2$ procedure: The reaction was set up identically to that described in "Yield Procedure" (A-D), but the following adjustments were made. At the same time $Cu(OTf)_2$ was added to the vial, 1,3,5-tri-tert-butylbenzene internal standard was added (12.3 mg, 0.05 mmol). At the conclusion of the reaction, a small sample (200 µL) of the reaction mixture was injected into a quenching solution composed of ethyl acetate and 1M sodium ascorbate solution (1 mL + 1 mL). After vigorously shaking, 0.5 mL of the organic layer was removed and injected on top of a one inch plug of dry silica gel inside of a Pasteur pipette. With the aid of pure ethanol, the sample was filtered directly into a 2 mL GC vial and analyzed via gas chromatography, to provide the reaction selectivity described in Table 2.

NHPI procedure: For comparison, the procedure of Inoue was also investigated.[12] Similar to the $Cu(OTf)_2$ procedure, 1,3,5-tri-tert-butylbenzene was used as an internal standard, and the reaction selectivity was analyzed by gas chromatography after quenching and filtration through silica gel in an identical manner.

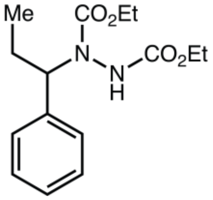

Compound 7: So that a gas chromatography conversion factor could be obtained, the authentic propylbenzene product was synthesized with a modification of the procedure of Inoue.[12] An oven-dried 25 mL 2-neck flask under N_2 was charged with N-hydroxyphthalimide (65 mg, 0.4 mmol, 0.2 equiv), anhydrous DCE (0.3 M, 6 mL), propylbenzene (280 µL, 2 mmol, 1 equiv), and DEAD (775 µL of 40 % solution in toluene, 2 mmol, 1 equiv). The reaction mixture was set heating to 60°C and stirred for 24 hours. The excess solvent was removed *in vacuo* and column chromatography over silica gel (3:1 hexane:EtOAc) afforded 102 mg (0.4 mmol, 20%) of a pale yellow oil.

^{1}H NMR ($CDCl_3$) δ 7.34 – 7.23 (m, 5H), 6.14 (s, 1H), 5.32 – 5.00 (m, 1H), 4.31 – 4.01 (m, 4H), 2.10 – 1.95 (m, 1H), 1.88 (dp, *J* = 14.5, 7.4 Hz, 1H), 1.39 – 1.08 (m, 6H), 0.94 (q, *J* = 17.5, 12.5 Hz, 3H). ^{13}C NMR ($CDCl_3$) δ 156.70, 156.25, 139.24, 128.57, 128.15, 127.89, 62.58, 61.95, 29.77, 23.79, 14.59, 14.51, 11.23. HRMS m/z calcd. for $C_{15}H_{22}N_2O_4$ $[M+H]^+$ 295.1652, found 295.1649.

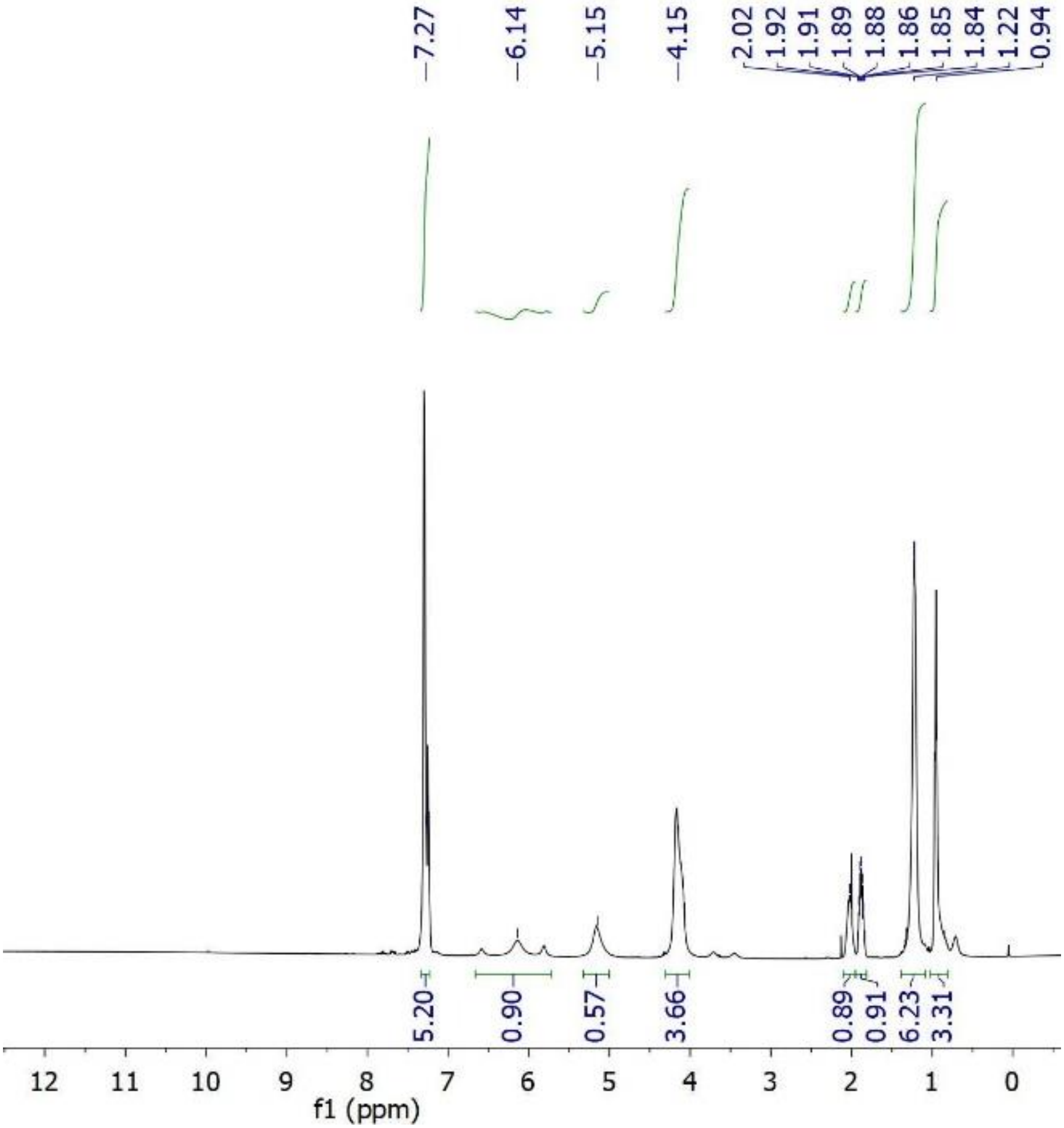

Figure S30: ^{1}H NMR of **7**

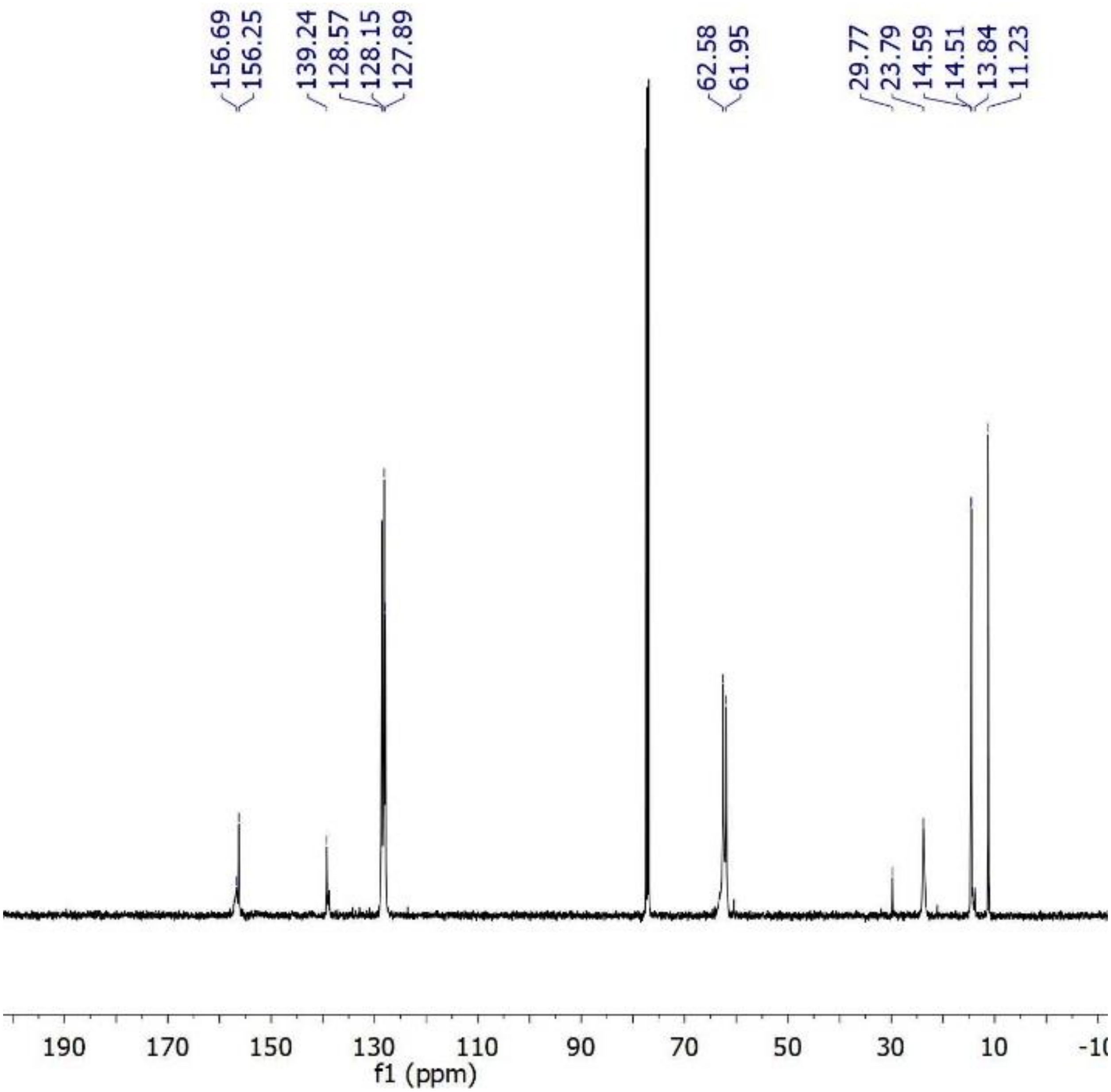

Figure S31: ^{13}C NMR of **7**

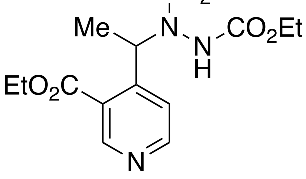

Compound 10: Amination was accomplished using procedure **A**. Column chromatography over silica gel (3:1 hexane:EtOAc) afforded 105.2 mg (0.30 mmol, 60%) of a colorless oil.

^{1}H NMR ($CDCl_3$) δ = 8.96-8.91 (m, 1H), 8.62-8.57 (m, 1H), 7.2307.18 (m, 1H), 6.02-5.89 (m, 1H), 4.35 (q, *J* = 6.7 Hz, 2H), 4.18-4.11 (m, 2H), 4.05-3.98 (m, 2H), 1.49(m, 3H), 1.35 (t, *J* = 6.7 Hz, 3H), 1.25-

1.20 (m, 3H), 1.12-1.03 (m, 3H). ^{13}C NMR ($CDCl_3$) δ = 166.17, 157.21, 155.73, 152.48, 150.87, 145.29, 125.29, 121.65, 62.68, 62.19, 61.76, 54.39, 29.73, 18.17, 14.49, 14.24. HRMS m/z calcd. for $C_{16}H_{23}N_3O_4$ $[M+H]^+$ 354.1660, found 354.1647

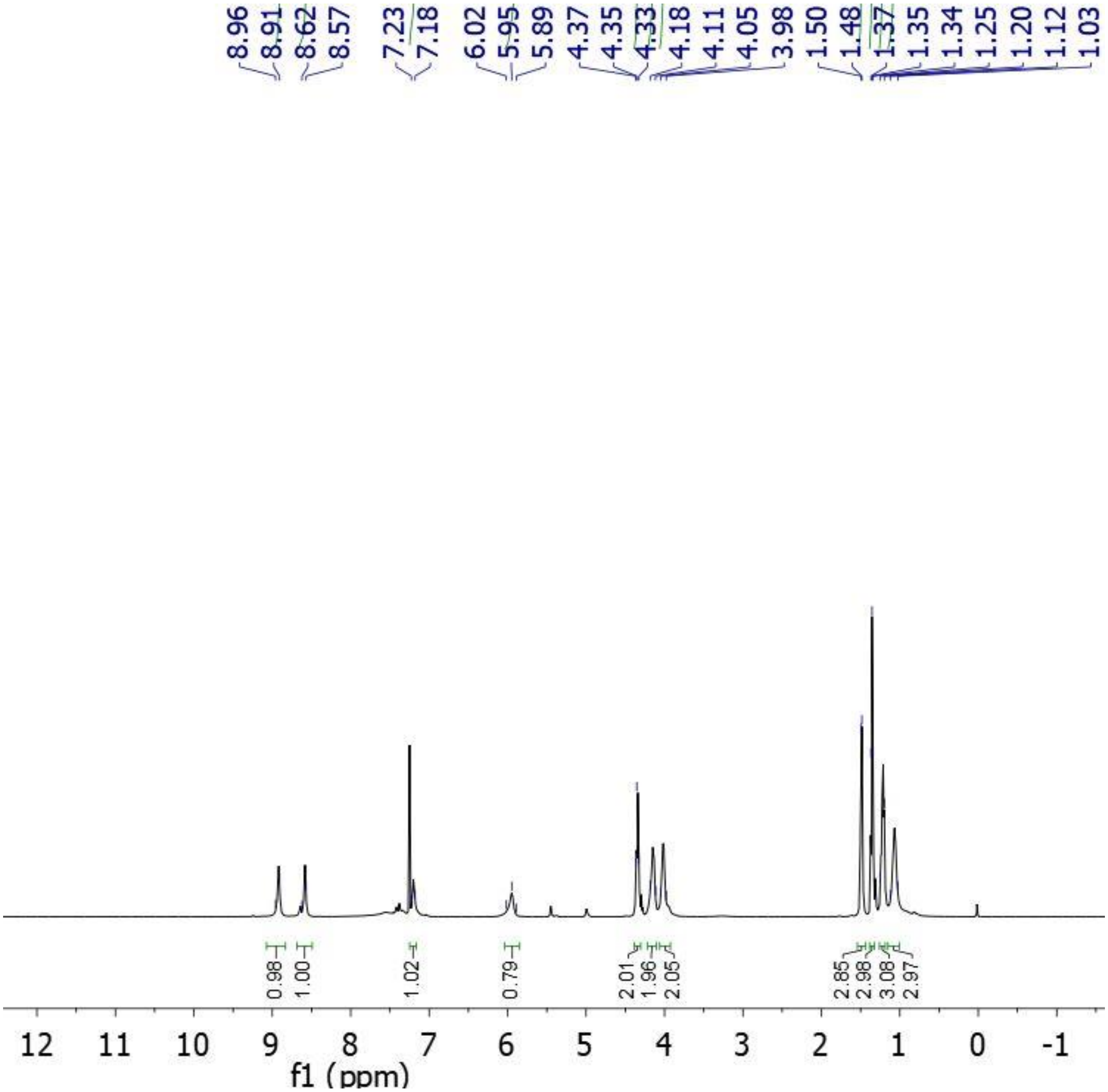

Figure S32: ^{1}H NMR of **10**

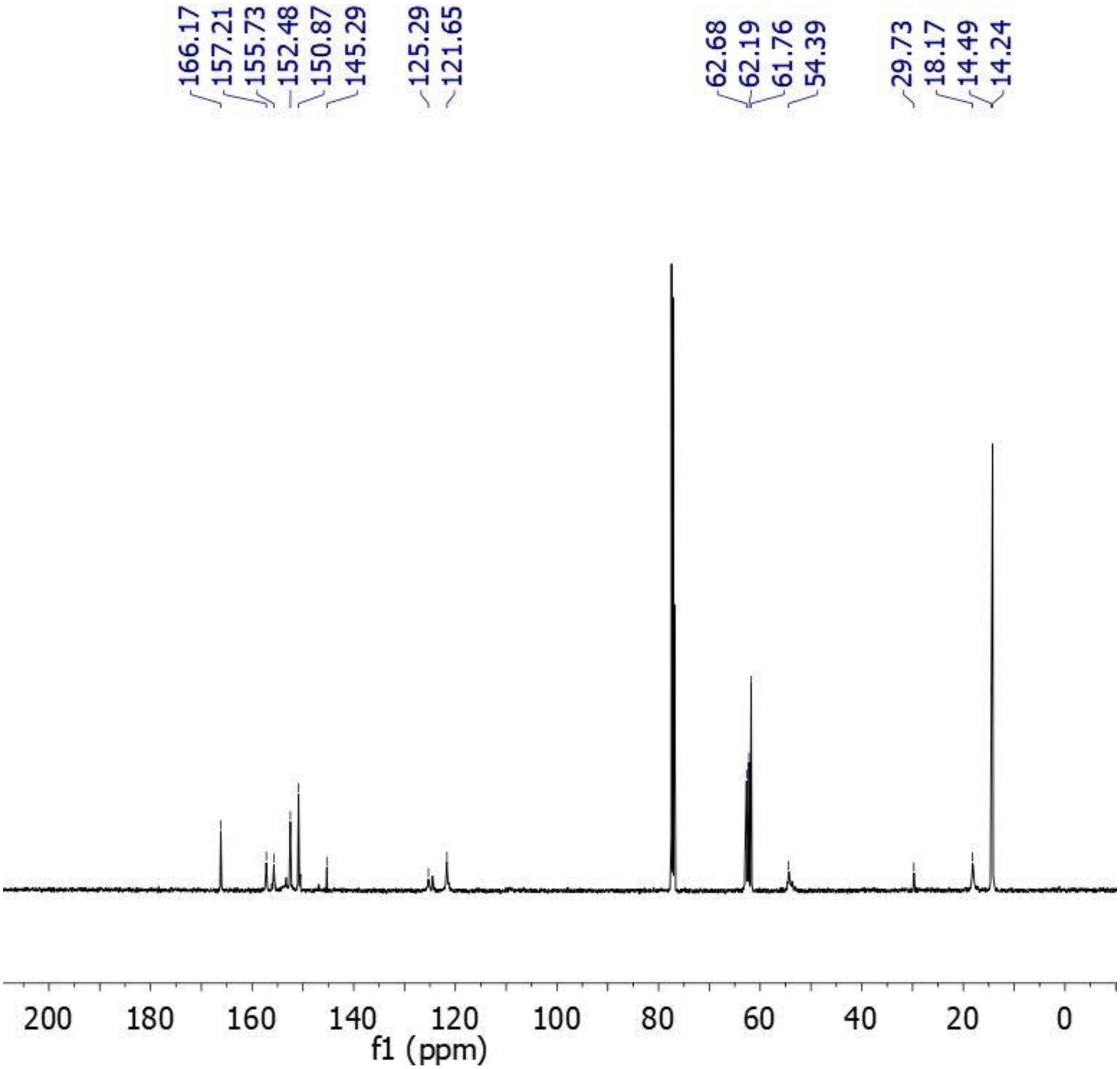

Figure S33: ^{13}C NMR of **10**

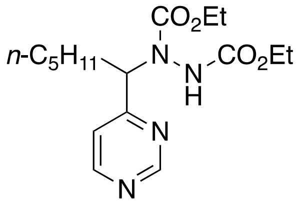

Compound 11: Amination was accomplished using procedure **B**. Column chromatography over silica gel (2:1 hexane:EtOAc) afforded 107.3 mg (0.32 mmol, 63%) of a light yellow oil.

^{1}H NMR ($CDCl_3$) δ = 9.12-9.01 (m, 1H), 8.69-8.58 (m, 1H), 7.37-7.24 (m, 1H), 5.24-5.01 (1H), 4.23-3.95 (m, 4H), 1.97-1.69 (m, 2H), 1.57-1.01 (m, 12H), 0.85-0.77 (m, 3H). ^{13}C NMR ($CDCl_3$) δ = 169.25, 158.67, 157.05, 120.59, 119.13, 62.90, 62.15, 62.07, 62.15, 62.07, 61.57, 31.64, 26.02, 22.51, 14.77, 14.58, 14.10. HRMS m/z calcd. for $C_{16}H_{26}N_4O_4$ $[M+H]^+$ 339.2027, found 339.2016

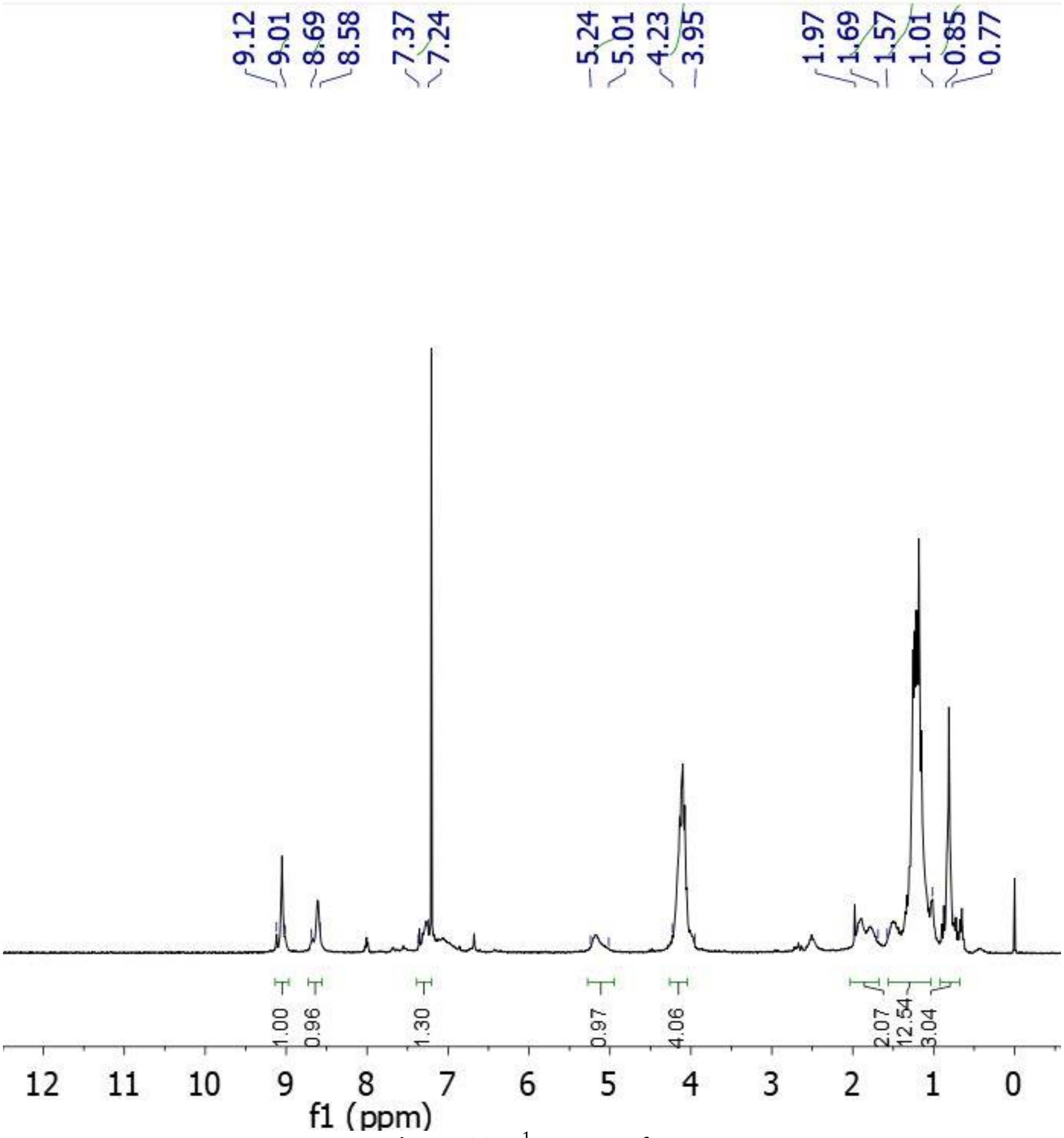

Figure S34: ^{1}H NMR of **11**

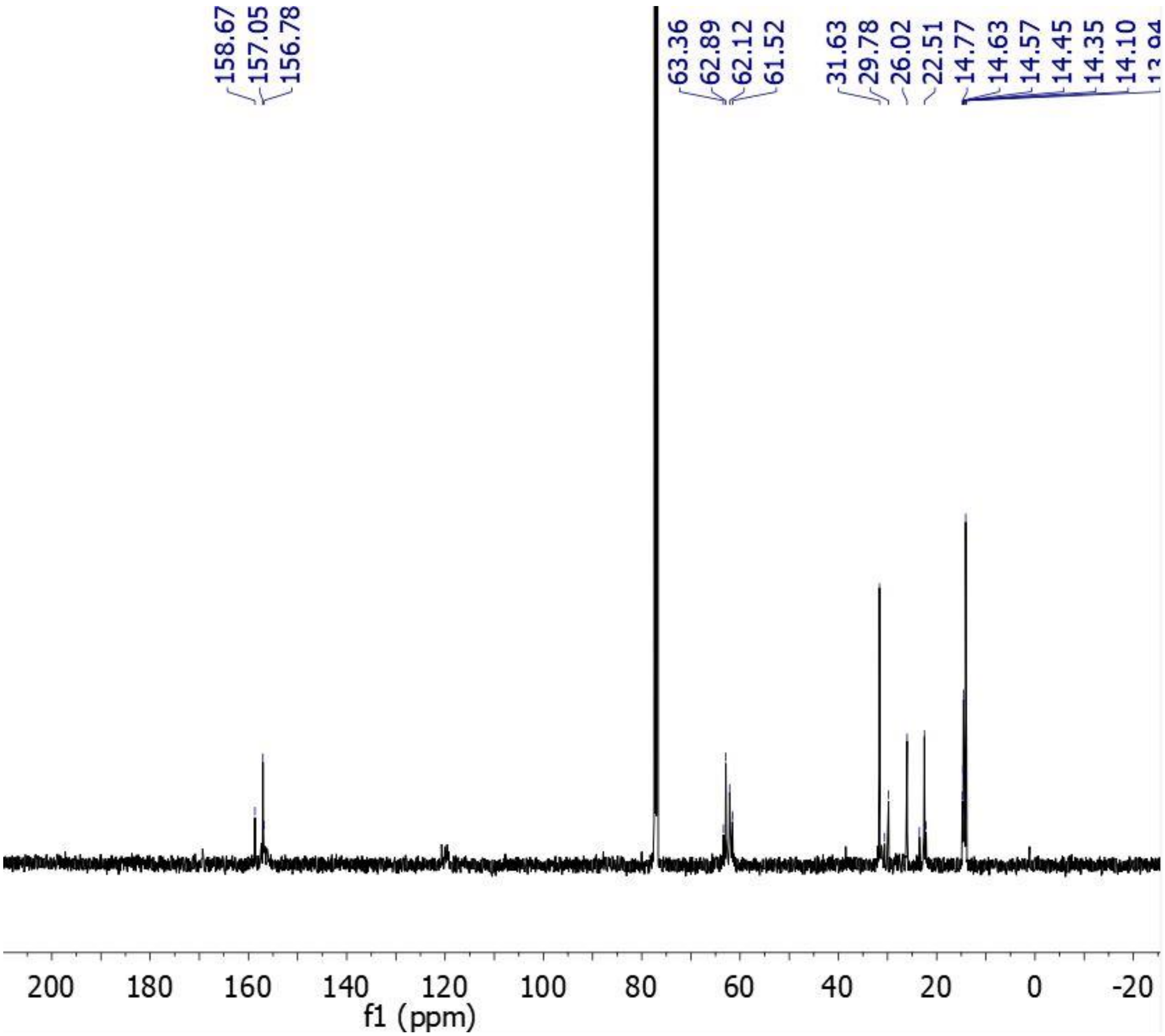

Figure S35: ^{13}C NMR of **11**

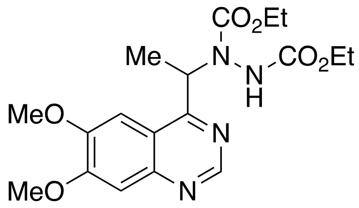

Compound 12: Amination was accomplished using procedure **A**. Column chromatography over silica gel (20:1 EtOAC:MeOH) afforded 141.5 mg (0.36 mmol, 72%) of a white solid.

^{1}H NMR ($CDCl_3$) δ = 8.91 (br s, 1H), 7.85 – 7.42 (br, split due to rotamers, 1H) 7.27-7.15 (br m, 2H) 6.19-5.91 (br m, 1H), 4.20-4.06 (m, 4H), 4.00-3.97 (m, 6H), 1.62-1.61 (br d, 3H, J = 6.5 Hz), 1.30-1.20 (m, 6H). ^{13}C NMR ($CDCl_3$) δ = 167.39, 166.84, 156.76, 156.38, 155.92, 153.00, 150.59, 117.66, 107.05, 101.18, 62.82, 61.84, 56.32, 53.61, 53.46, 16.52, 14.58, 14.48. HRMS m/z calcd. for $C_{18}H_{24}N_4O_6$ $[M+H]^+$ 393.1769, found 393.1751

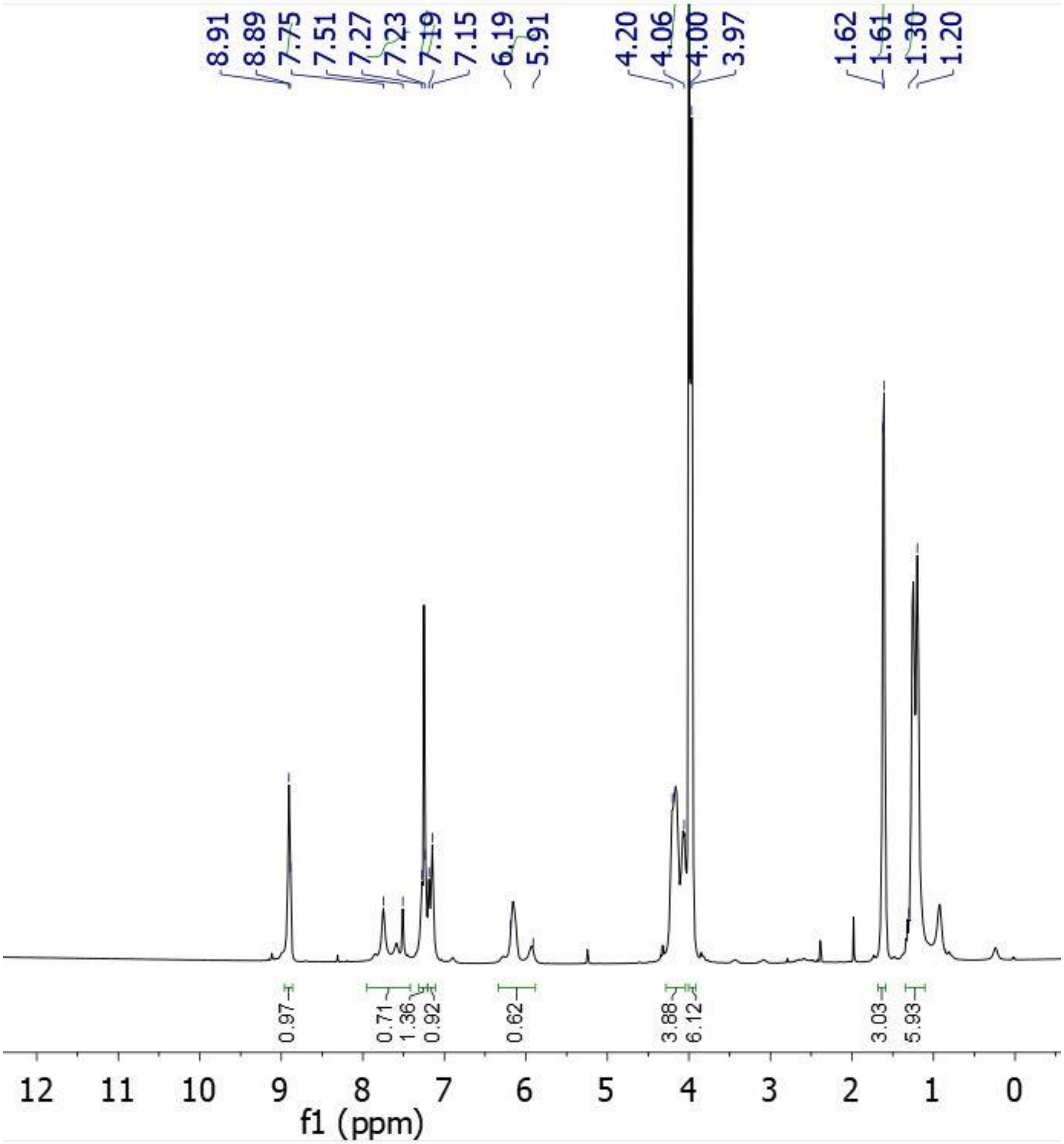

Figure S36: ^{1}H NMR of **12**

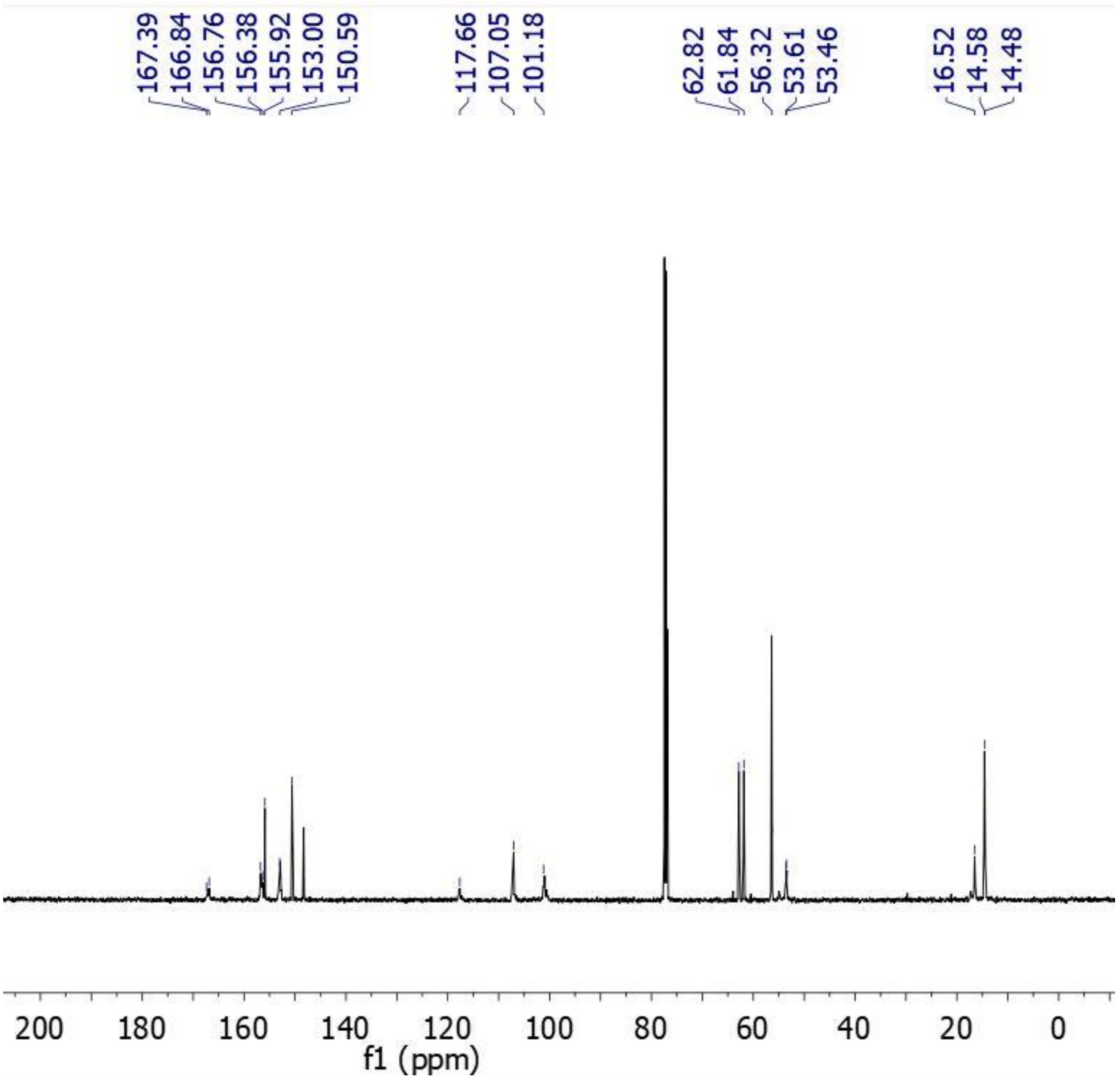

Figure S37: ^{13}C NMR of **12**

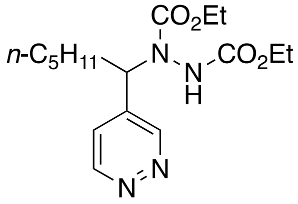

Compound 13: Amination was accomplished using procedure **A**. Column chromatography over silica gel (4:1 EtOAc:hexane) afforded 65.4 mg (0.19 mmol, 39%) of a yellow oil.
^{1}H NMR ($CDCl_3$) δ = 9.31-9.12 (m, 2H), 7.59-7.45 (m, 1H), 6.84-6.65 (m, 1H), 5.39-5.10 (br s, 1H), 4.31-4.10 (m, 4H), 2.37-2.23 (m, 1H), 2.09-2.04 (m, 1H), 1.90-1.83 (m, 1H), 1.55-0.73 (m, 14H). ^{13}C NMR ($CDCl_3$) δ = 169.19, 158.67, 157.05, 120.70, 120.66, 119.33, 63.36, 62.90, 62.12, 61.52, 31.64, 29.78, 26.02, 22.51, 14.58, 14.10. HRMS m/z calcd. for $C_{16}H_{26}N_4O_4$ $[M+H]^+$ 339.2027, found 339.2038

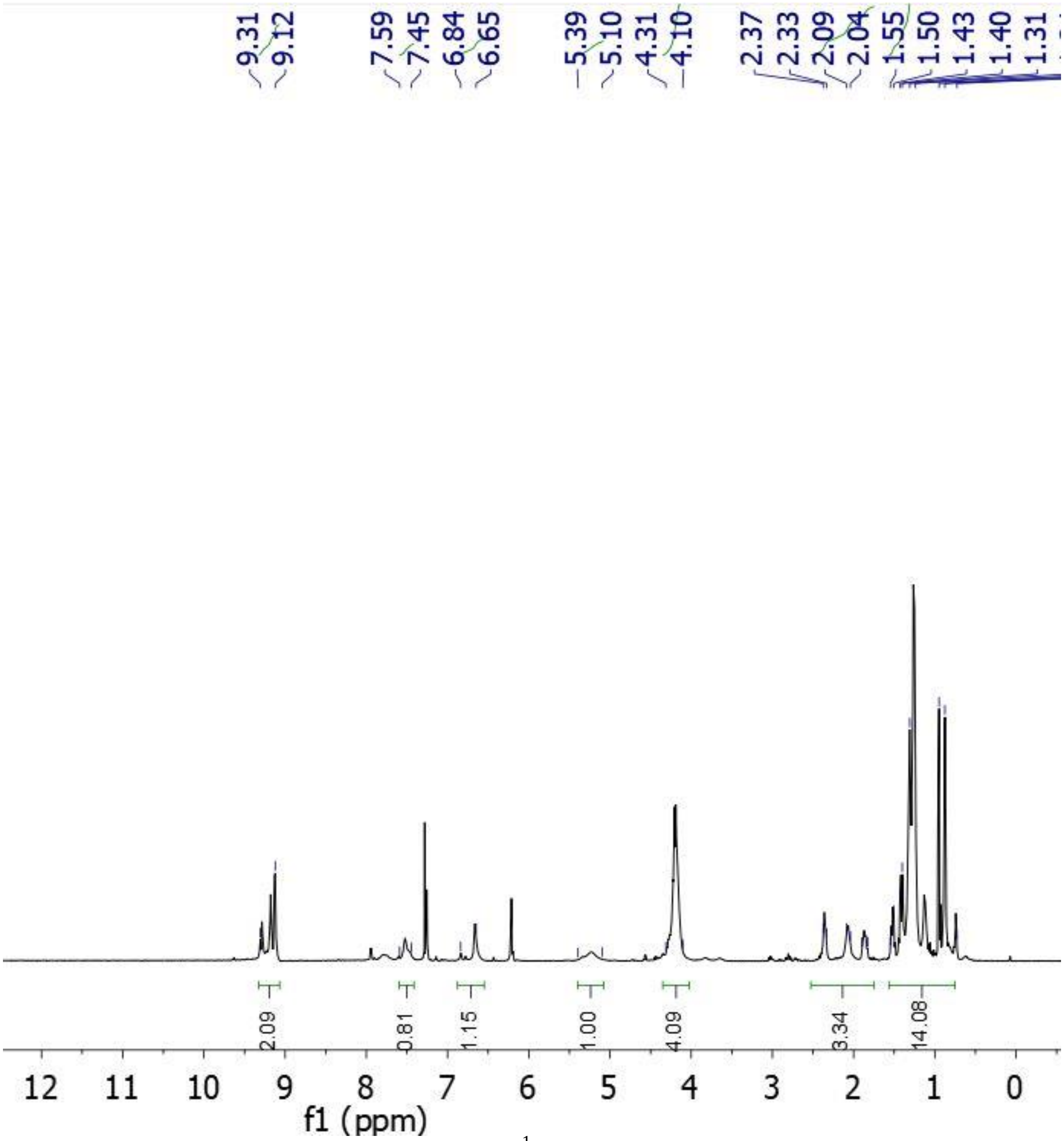

Figure S38: ^{1}H NMR of **13**

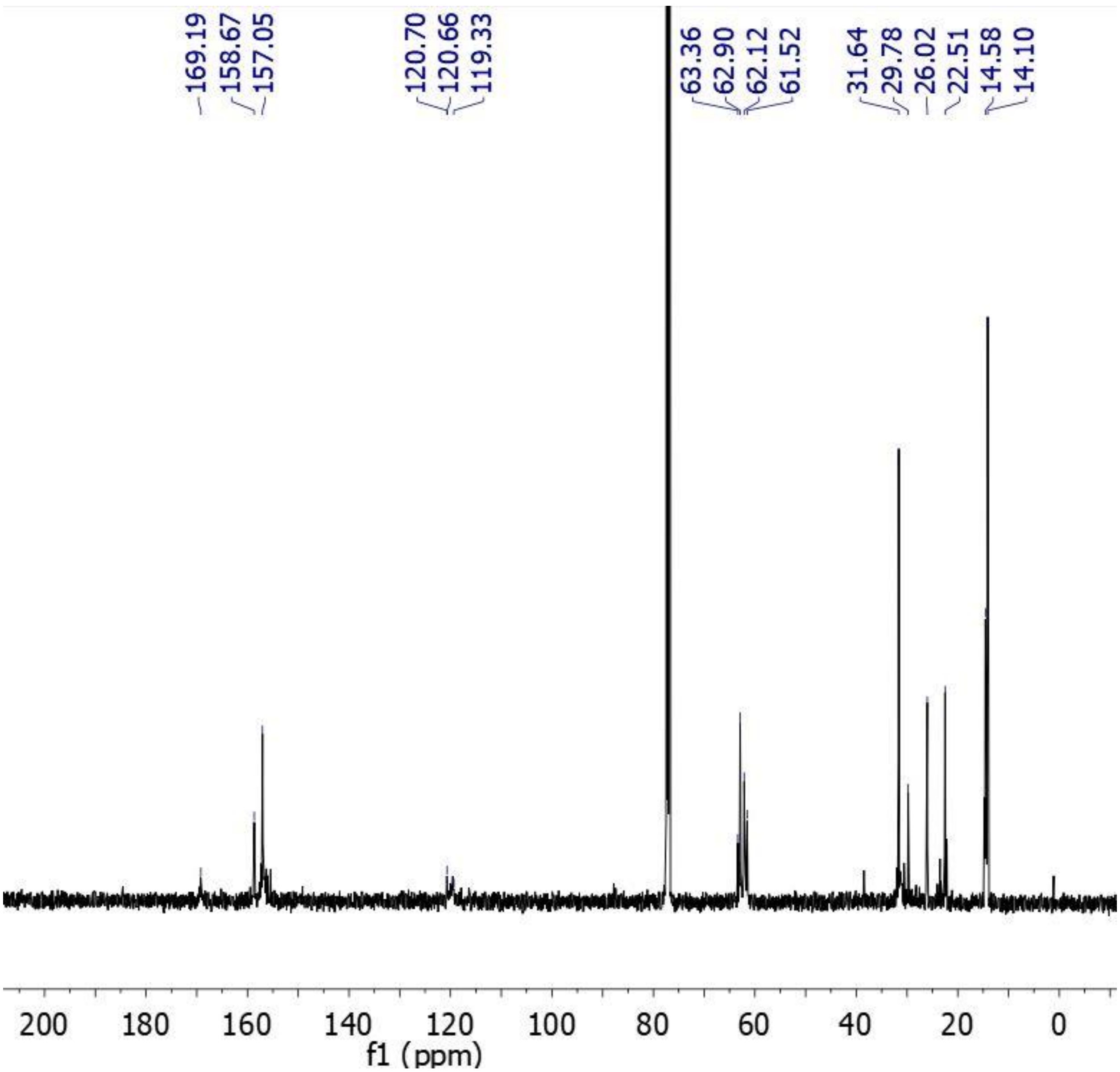

Figure S39: ^{13}C NMR of **13**

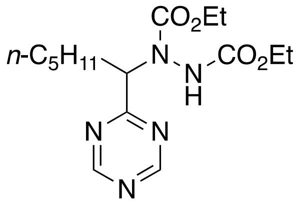

Compound 14: Amination was accomplished using procedure **B**. Column chromatography over silica gel (2:1 hexane:EtOAc) afforded 90.5 mg (0.27 mmol, 53%) of a colorless oil. ^{1}H NMR ($CDCl_3$) δ = 9.08 (s, 2H), 7.05-6.96 (m, 1H), 5.36-5.20 (m, 1H), 4.26-4.13 (m, 4H), 2.11-1.45 (m, 4H), 1.45-1.30 (m, 4H), 1.22-1.11 (m, 6H), 0.89-0.79 (m, 3H). ^{13}C NMR ($CDCl_3$) δ = 178.70, 166.10, 157.26, 76.90, 64.19, 63.62, 63.00, 62.23, 61.93, 31.53, 30.65, 26.09, 22.45, 14.48, 14.11. HRMS m/z calcd. for $C_{15}H_{22}N_4O_6$ $[M+H]^+$ 354.1561, found 355.1551

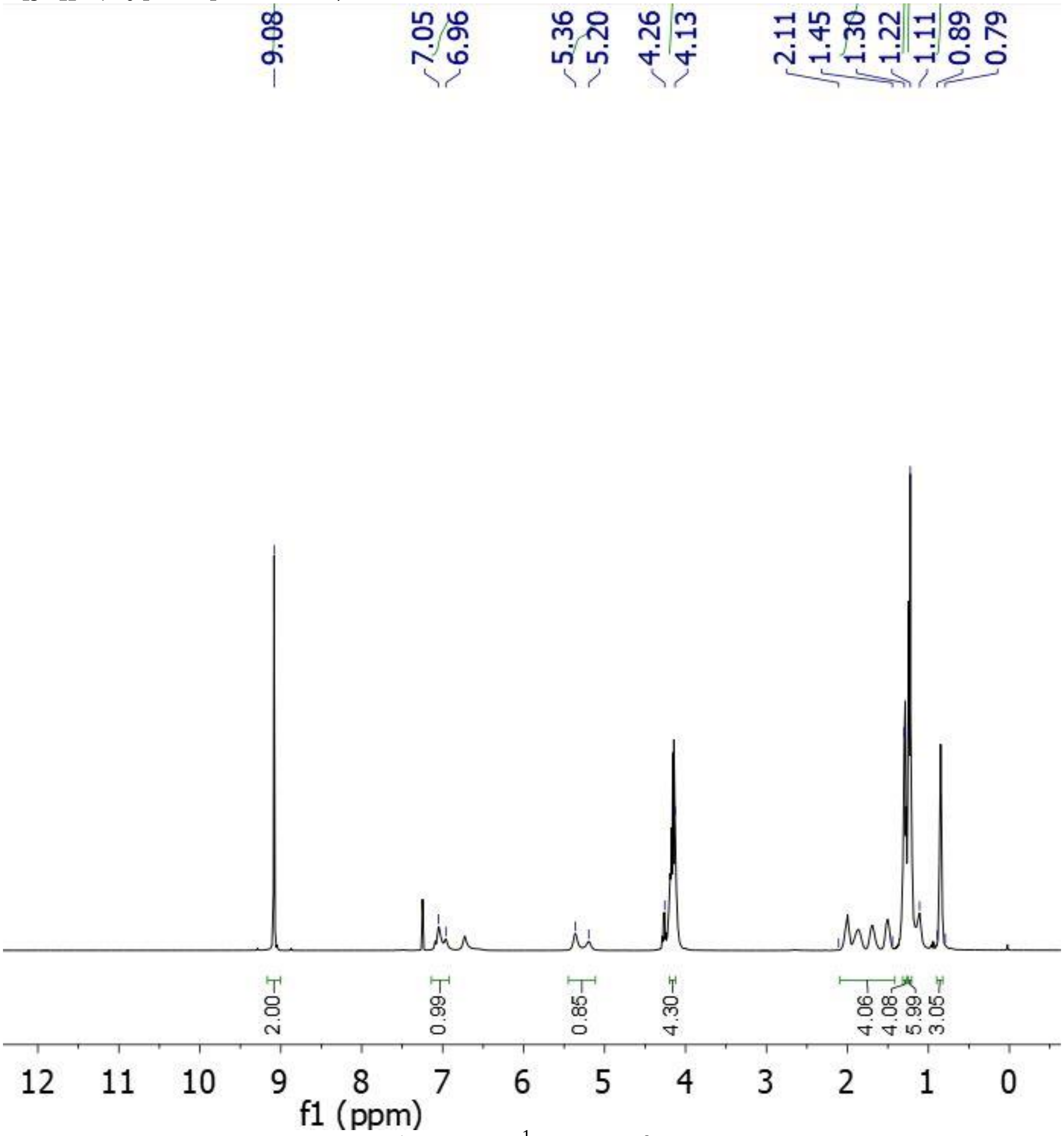

Figure S40: ^{1}H NMR of **14**

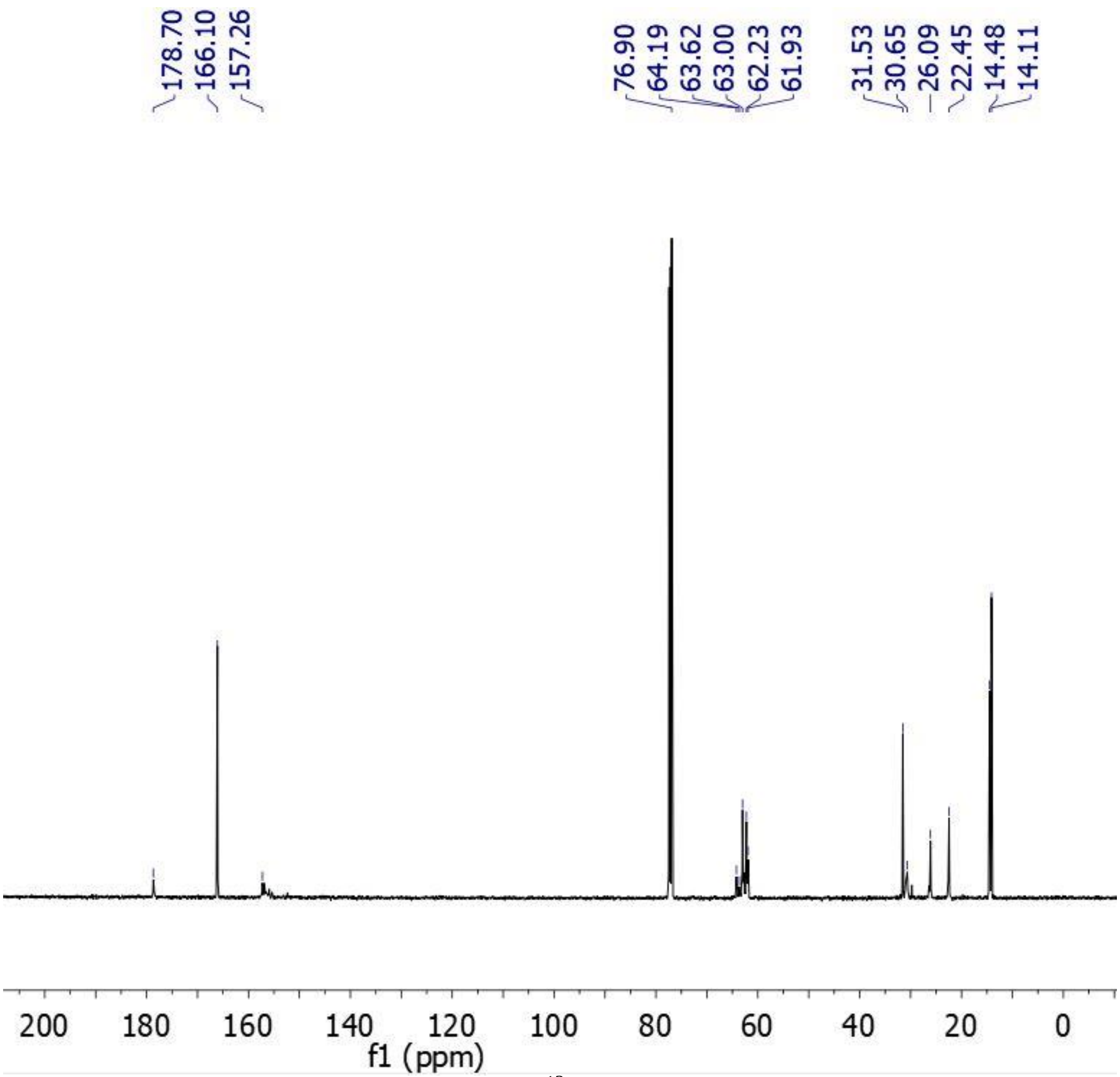

Figure S41: ^{13}C NMR of **14**

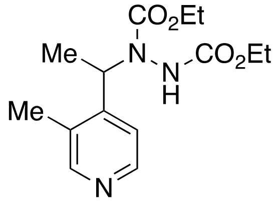

Compound 15: Amination was accomplished using procedure **A**. Column chromatography over silica gel (20:1 dichloromethane:MeOH) afforded 97.3 mg (0.35 mmol, 69%) of a colorless oil.

^{1}H NMR ($CDCl_3$) δ = 8.28-8.24 (m, 2H), 7.25-7.05 (m, 1H), 5.53-5.48 (m, 1H), 4.17-4.13 (m, 4H), 2.30 (s, 3H), 1.46 (d, *J* = 1.5 Hz, 3H), 1.24-1.19 (m, 6H). ^{13}C NMR ($CDCl_3$) δ = 157.0, 156.8, 155.6, 150.8, 147.9, 147.4, 121.2, 62.8, 62.1, 52.5, 29.8, 16.8, 16.1, 14.5. HRMS m/z calcd. for $C_{14}H_{21}N_3O_4$ $[M+H]^+$ 296.1605, found 296.159

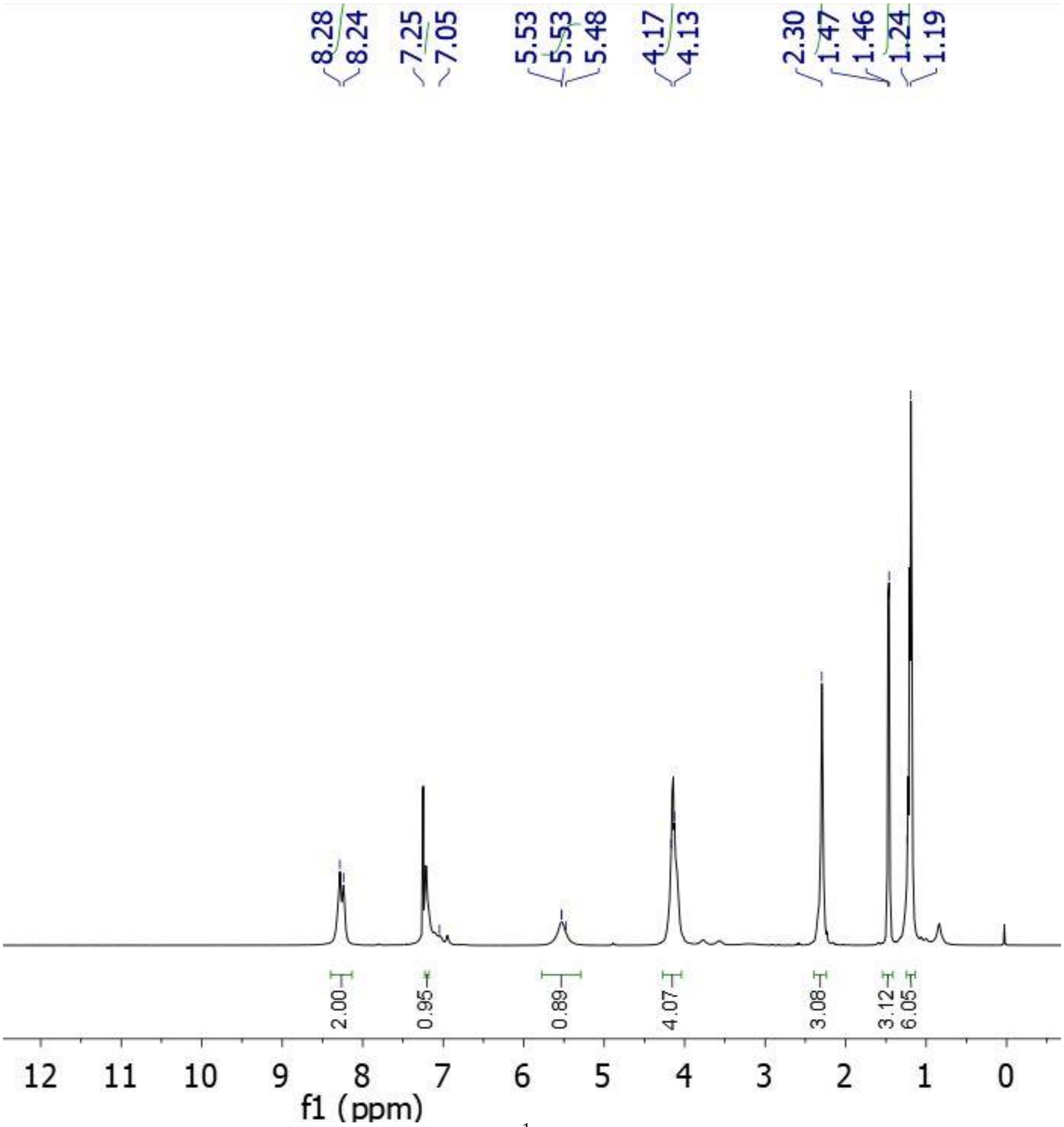

Figure S42: ^{1}H NMR of **15**

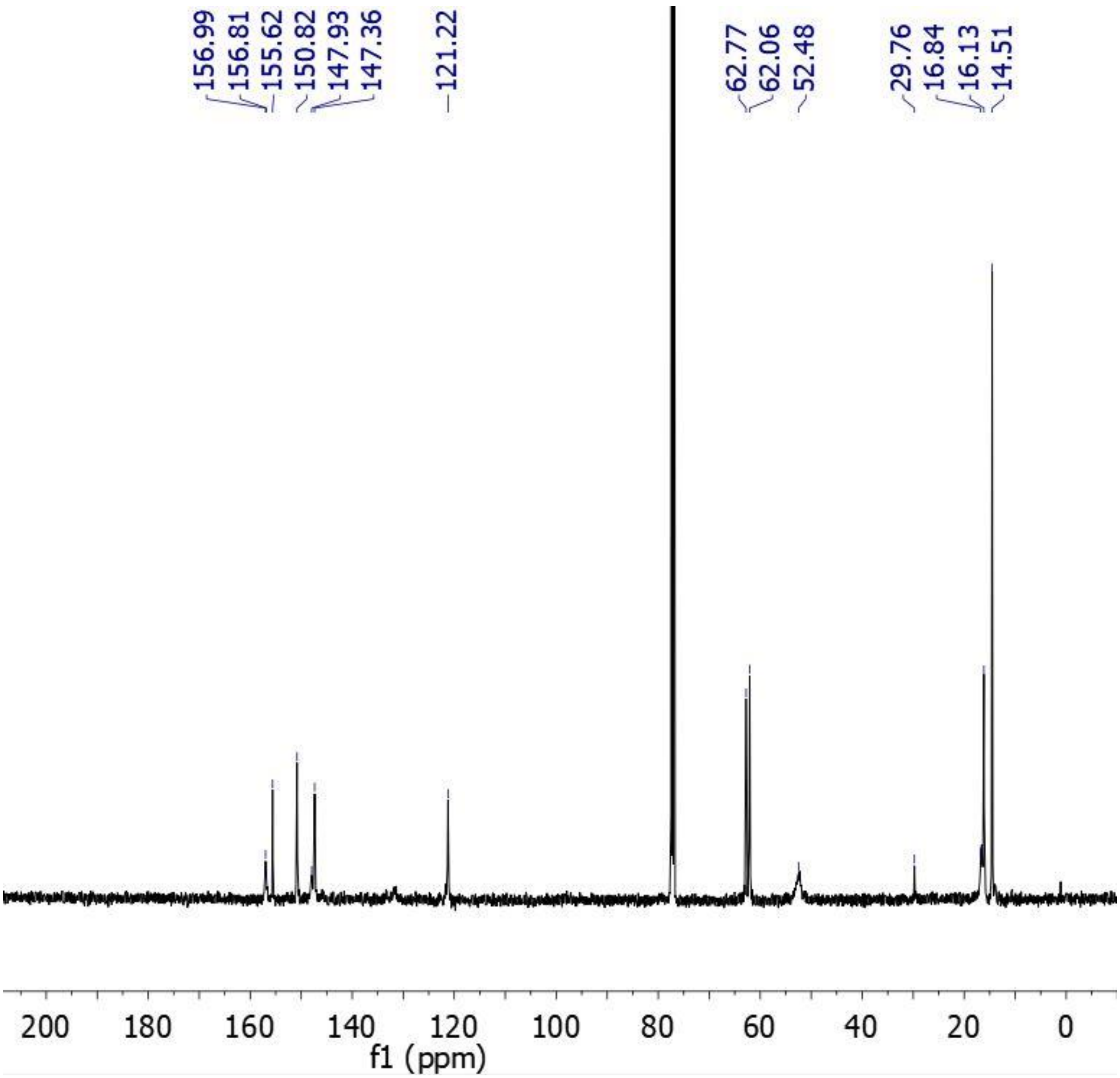

Figure S43: ^{13}C NMR of **15**

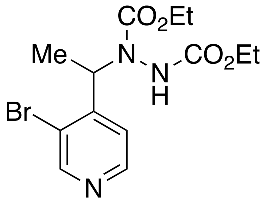

Compound 16: Amination was accomplished using procedure **B**. Column chromatography over silica gel (2:1 EtOAc:hexane) afforded 104.2 mg (0.29 mmol, 58%) of a colorless oil.

^{1}H NMR ($CDCl_3$) δ = 8.66-8.57 (m, 1H), 8.41-8.40 (m, 1H), 6.98-6.89 (m, 1H), 5.59-5.91 (m, 1H), 4.14-4.11 (m, 4H), 1.48 (d, *J* = 1.5 Hz, 3H), 1.24-1.17 (m, 6H). ^{13}C NMR ($CDCl_3$) δ = 157.2, 155.6, 152.1, 150.0, 148.4, 123.1, 121.7, 62.9, 62.3, 56.8, 29.8, 17.1, 14.5. HRMS m/z calcd. for $C_{13}H_{18}BrN_3O_4$ $[M+H]^+$ 360.0553, found 360.0528

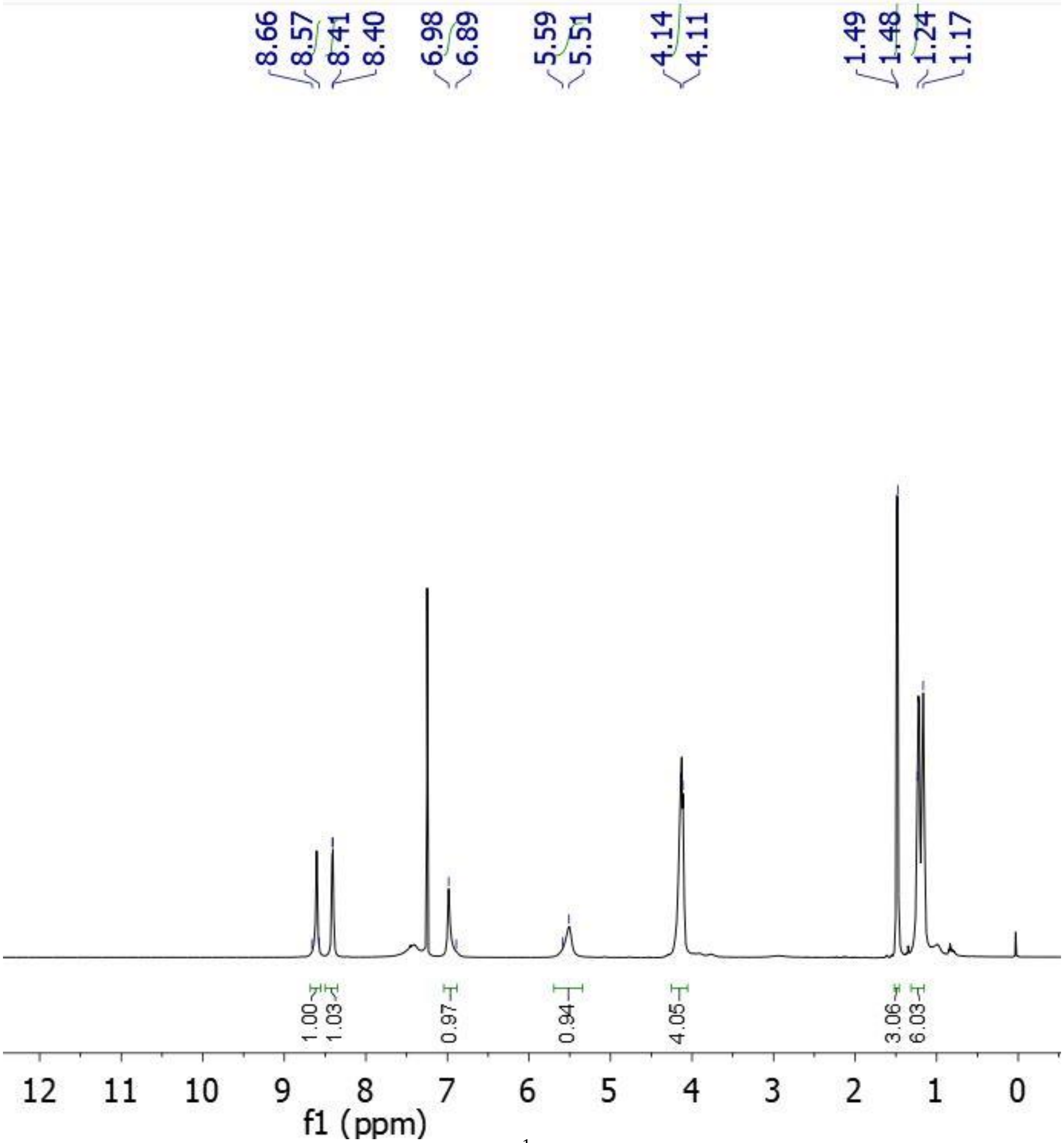

Figure S44: ^{1}H NMR of **16**

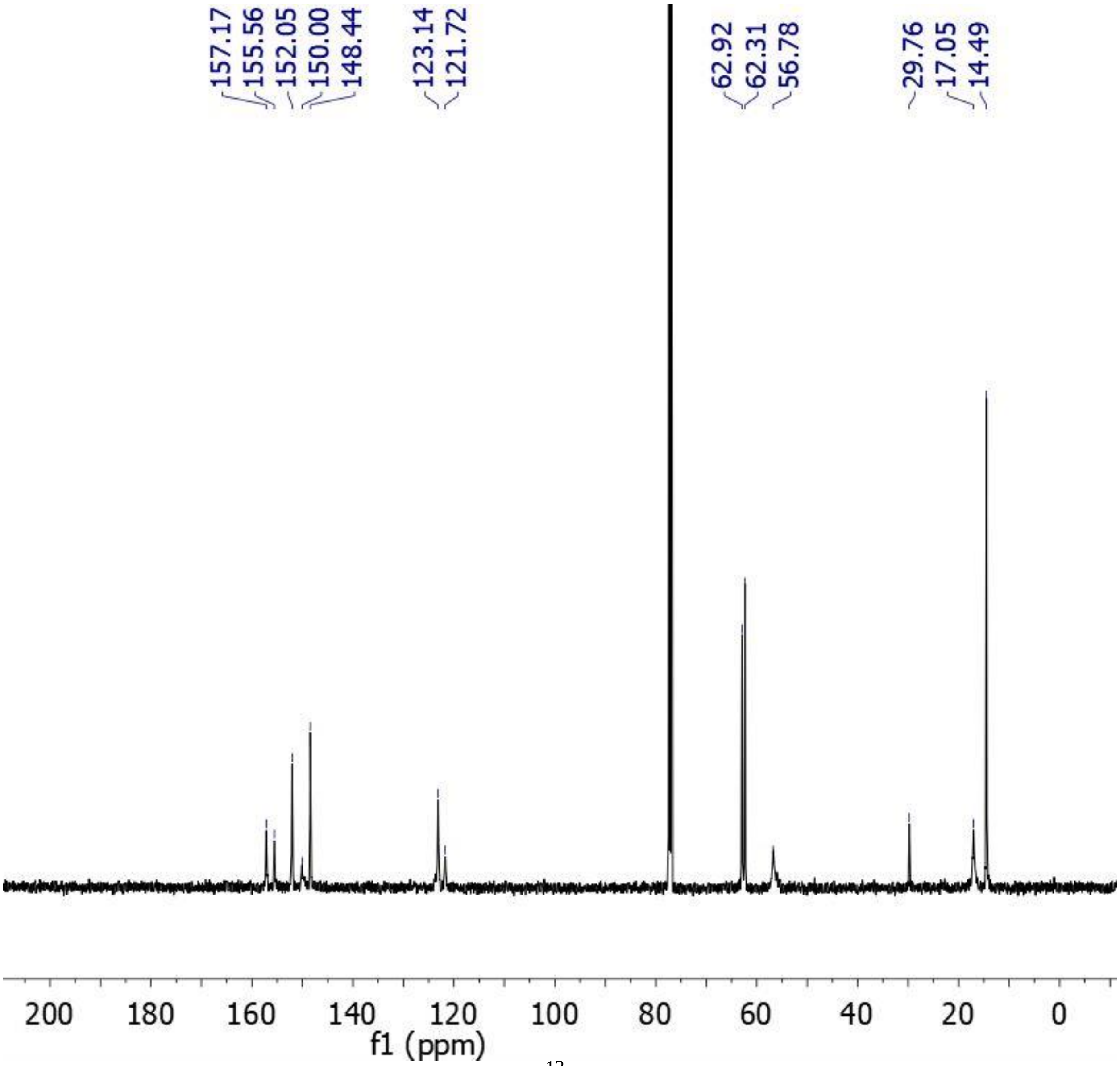

Figure S45: ^{13}C NMR of **16**

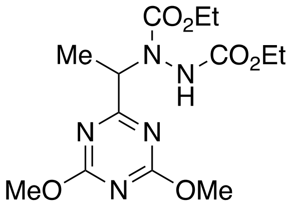

Compound 17: Amination was accomplished using procedure **A**. Column chromatography over silica gel (2:1 EtOAc:hexane) afforded 110.2 mg (0.32 mmol, 64%) of a colorless oil.

^{1}H NMR ($CDCl_3$) δ = 7.28 (s, 1H), 5.51-5.14 (m, 1H), 4.24-4.03 (m, 4H), 3.51 (s, 3H), 3.30 (s, 3H), 1.48 (d, *J* = 6.9 Hz, 3H), 1.23 (q, *J* = 7.0 Hz, 6H). ($CDCl_3$) δ = 169.69, 156.84, 156.35, 154.73, 150.97, 63.37, 62.12, 53.94, 31.33, 29.26, 14.70, 14.49, 14.37. HRMS m/z calcd. for $C_{13}H_{21}N_5O_6$ $[M+H]^+$ 344.1565, found 344.155

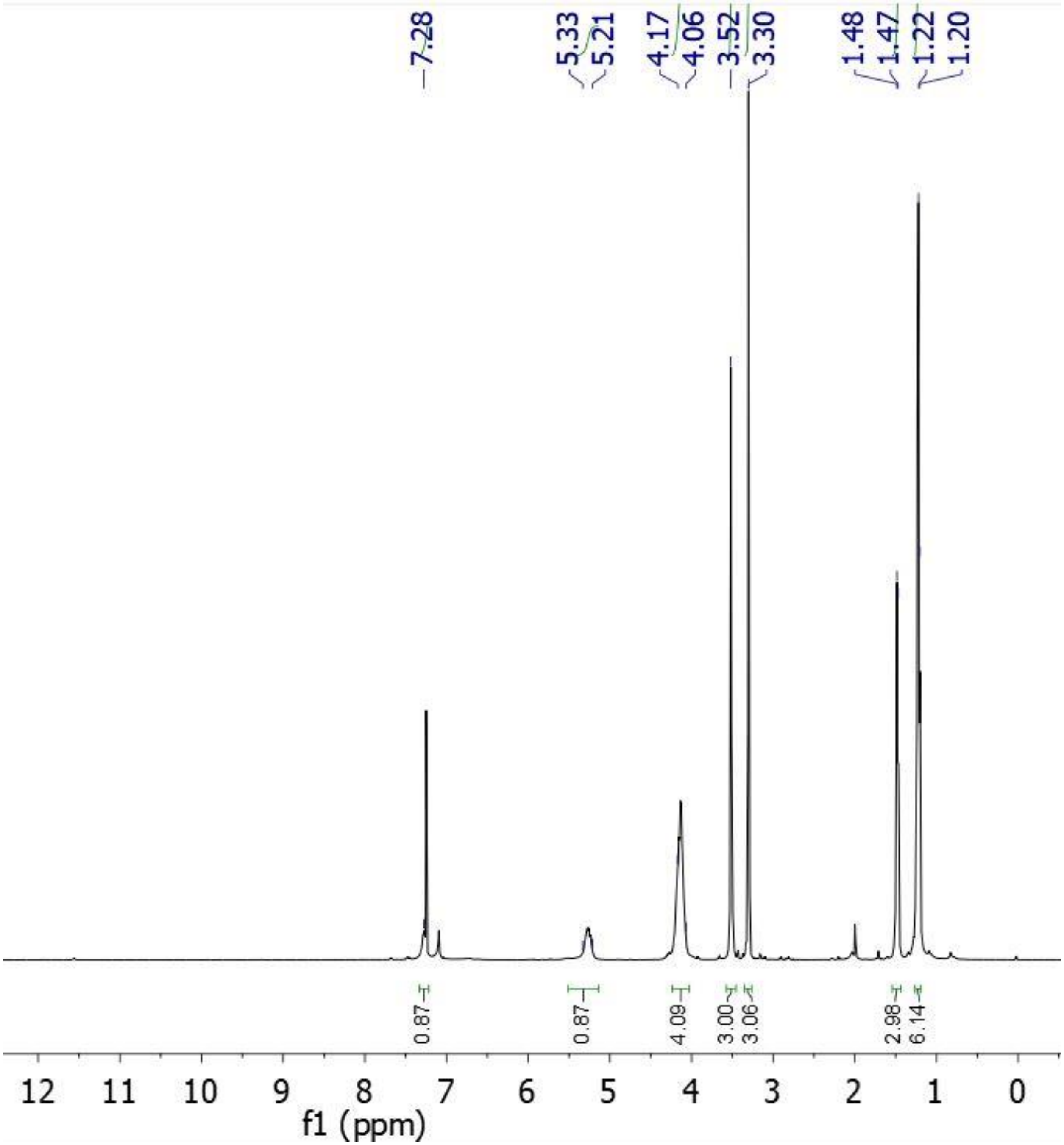

Figure S46: ^{1}H NMR of **17**

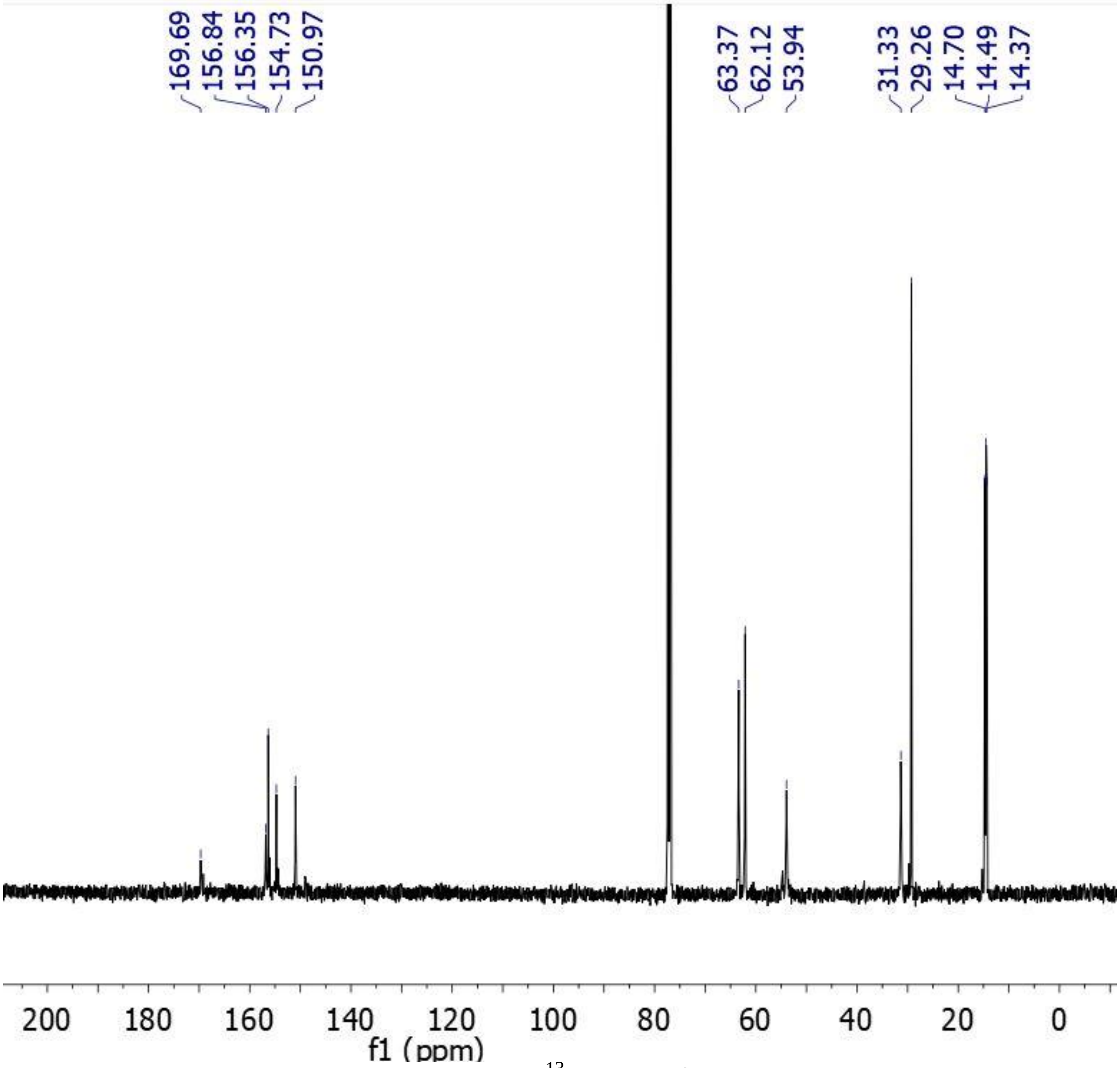

Figure S47: ^{13}C NMR of **17**

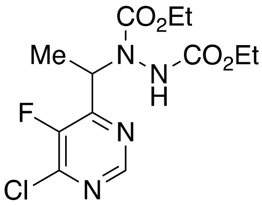

Compound 9: Amination was accomplished using procedure **A**. Column chromatography over silica gel (2:1 hexane:EtOAc) afforded 121.8 mg (0.36 mmol, 76%) of a colorless oil.
^{1}H NMR ($CDCl_3$) δ = 8.63 (s, 1H), 7.08-6.95 (m, 1H), 5.73-5.59 (m, 1H), 4.26-4.03 (m, 4H), 1.59-1.47 (m, 3H), 1.30-1.13 (m, 6H). ($CDCl_3$) δ = 158.93, 156.58, 156.24, 153.25, 148.32, 148.18, 63.02, 62.09, 52.47, 15.85, 14.53, 14.40. HRMS m/z calcd. for $C_{12}H_{16}ClFN_4O_4$ $[M+H]^+$ 335.0917, found 335.0926

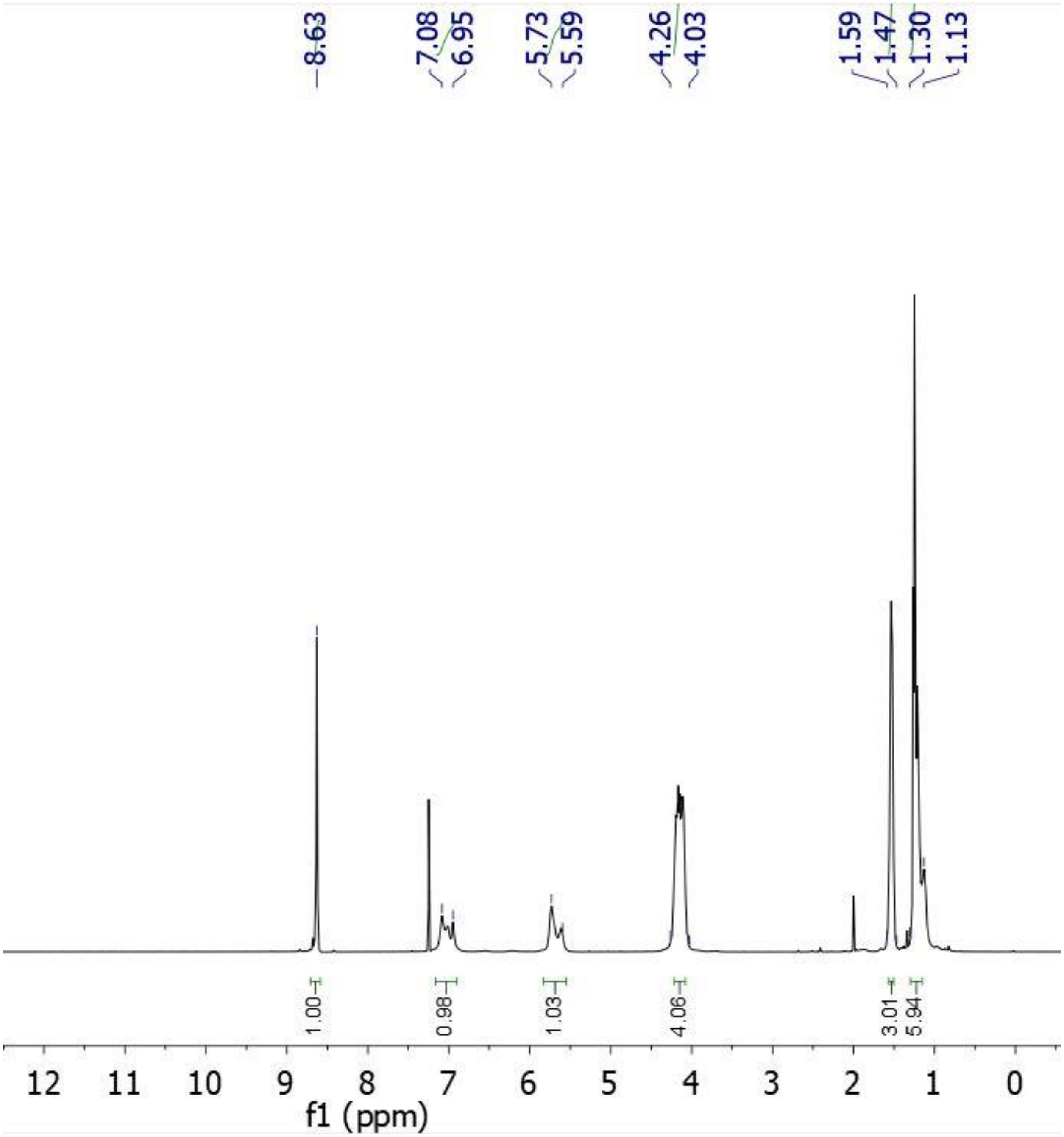

Figure S48: ^{1}H NMR of **9**

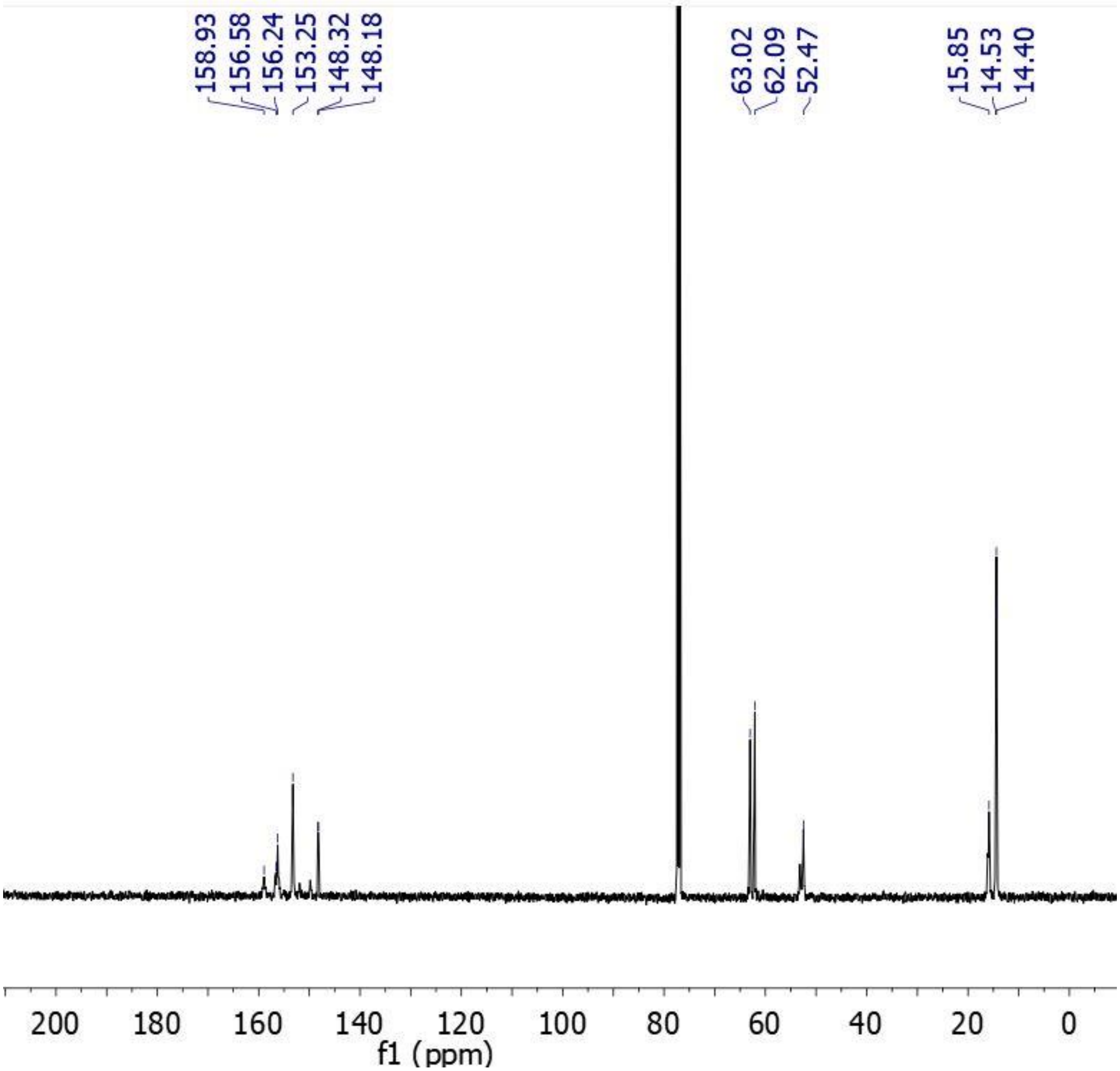

Figure S49: ^{13}C NMR of **9**

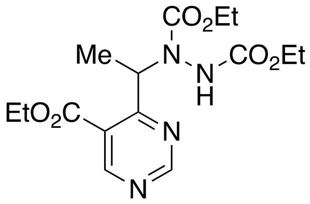

Compound 18: Amination was accomplished using procedure **A**. Column chromatography over silica gel (2:1 EtOAc:hexane) afforded 108 mg (0.31 mmol, 61%) of a colorless oil.

^{1}H NMR ($CDCl_3$) δ = 9.16-9.15 (m, 2H), 7.36-7.19 (m, 1H), 6.21-6.13 (m, 1H), 4.43 (q, *J* = 7.1 Hz, 2H), 4.25-4.02 (m, 4H), 1.53-1.49 (m, 3H), 1.41 (t, *J* = 7.1 Hz, 3H), 1.30-1.16 (m, 6H). ^{13}C NMR ($CDCl_3$) δ = 172.78, 163.95, 160.28, 159.36, 159.16, 156.70, 121.83, 62.71, 62.30, 61.89, 56.20, 55.67, 16.90, 14.61, 14.23. HRMS m/z calcd. for $C_{15}H_{22}N_4O_6$ $[M+H]^+$ 355.1549, found 355.1551

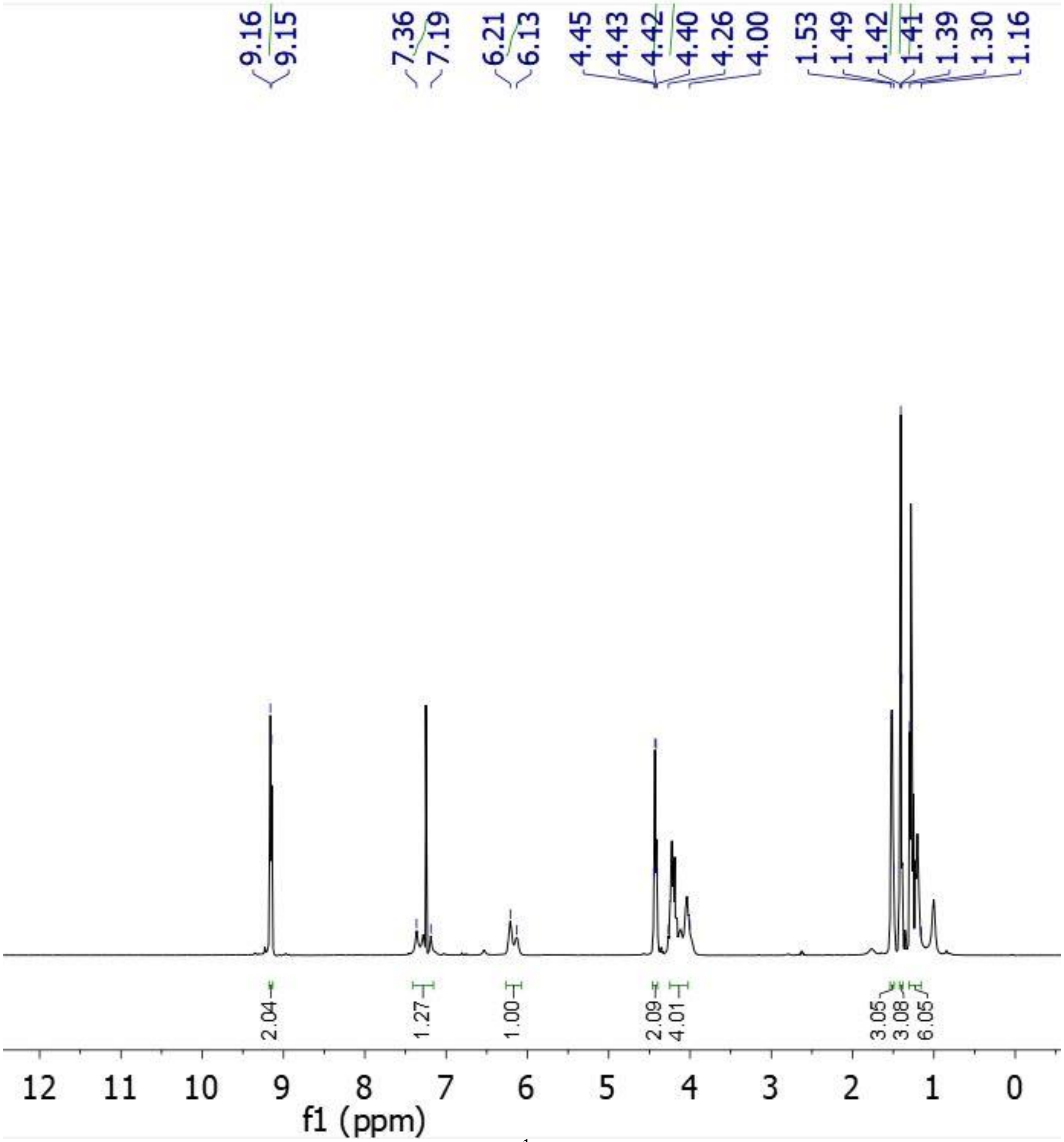

Figure S50: ^{1}H NMR of **18**

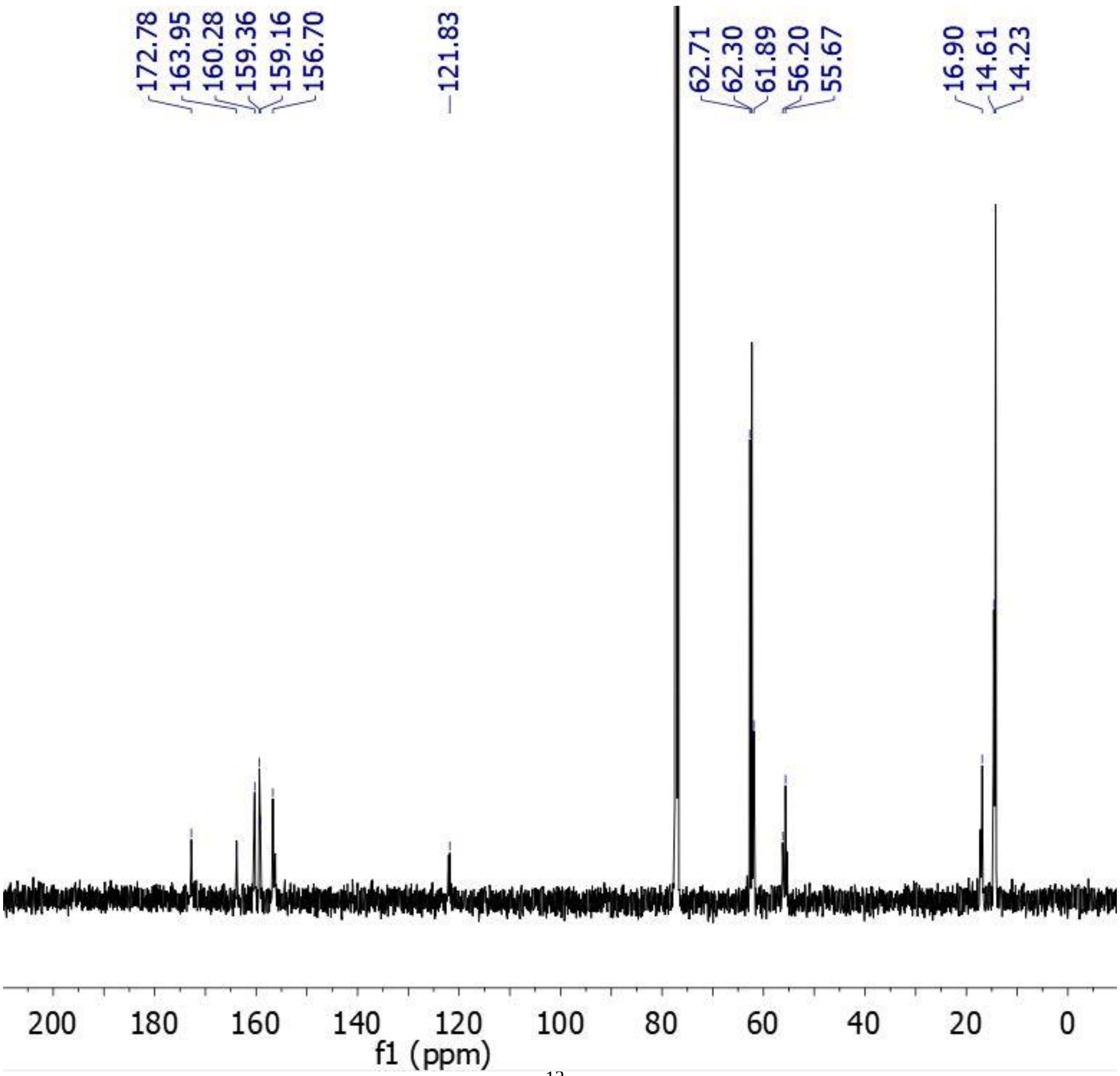

Figure S51: ^{13}C NMR of **18**

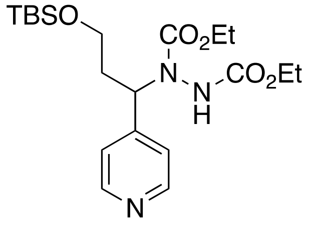

Compound 19: Amination was accomplished using procedure **A**. Column chromatography over silica gel (20:1 dichloromethane:MeOH) afforded 137 mg (0.32 mmol, 64%) of a colorless oil.

^{1}H NMR ($CDCl_3$) δ = 8.50-8.37 (m, 2H), 7.29-7.19 (m, 2H), 5.45-5.31 (m, 1H), 4.19-4.02 (m, 4H), 3.76-3.56 (m, 2H), 2.22-1.92 (m, 2H), 1.20-1.17 (m, 6H), 0.86-0.83 (m, 9H), 0.01-0.00 (m, 6H).). ^{13}C NMR ($CDCl_3$) δ = 156.95, 156.14, 149.65, 148.72, 122.96, 62.78, 62.07, 61.89, 59.56, 57.68, 32.92, 25.93, 18.24, 14.48, -5.51. HRMS m/z calcd. for $C_{19}H_{33}N_3O_5Si$ $[M+H]^+$ 426.2419, found 426.2421

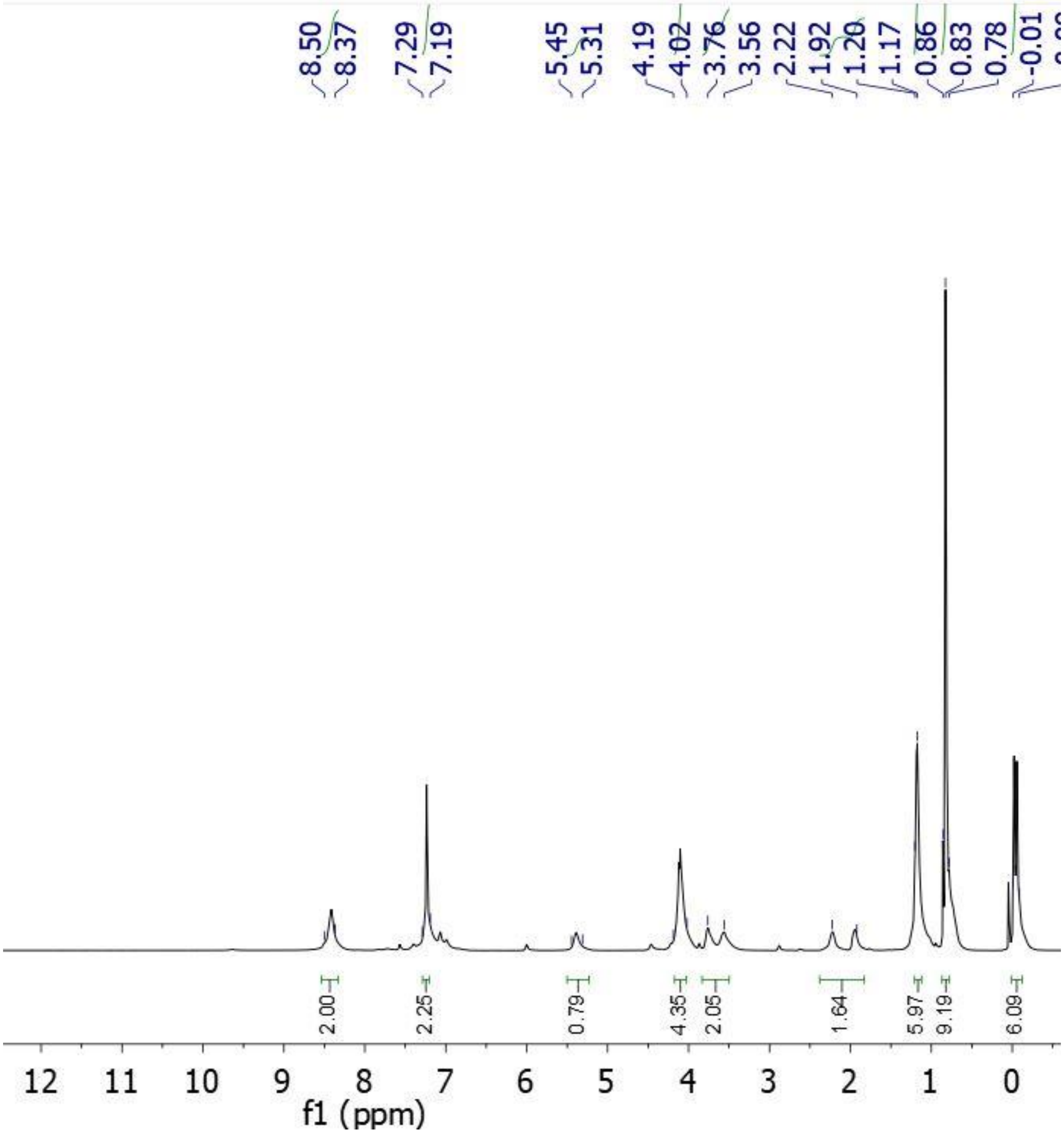

Figure S52: ^{1}H NMR of **20**

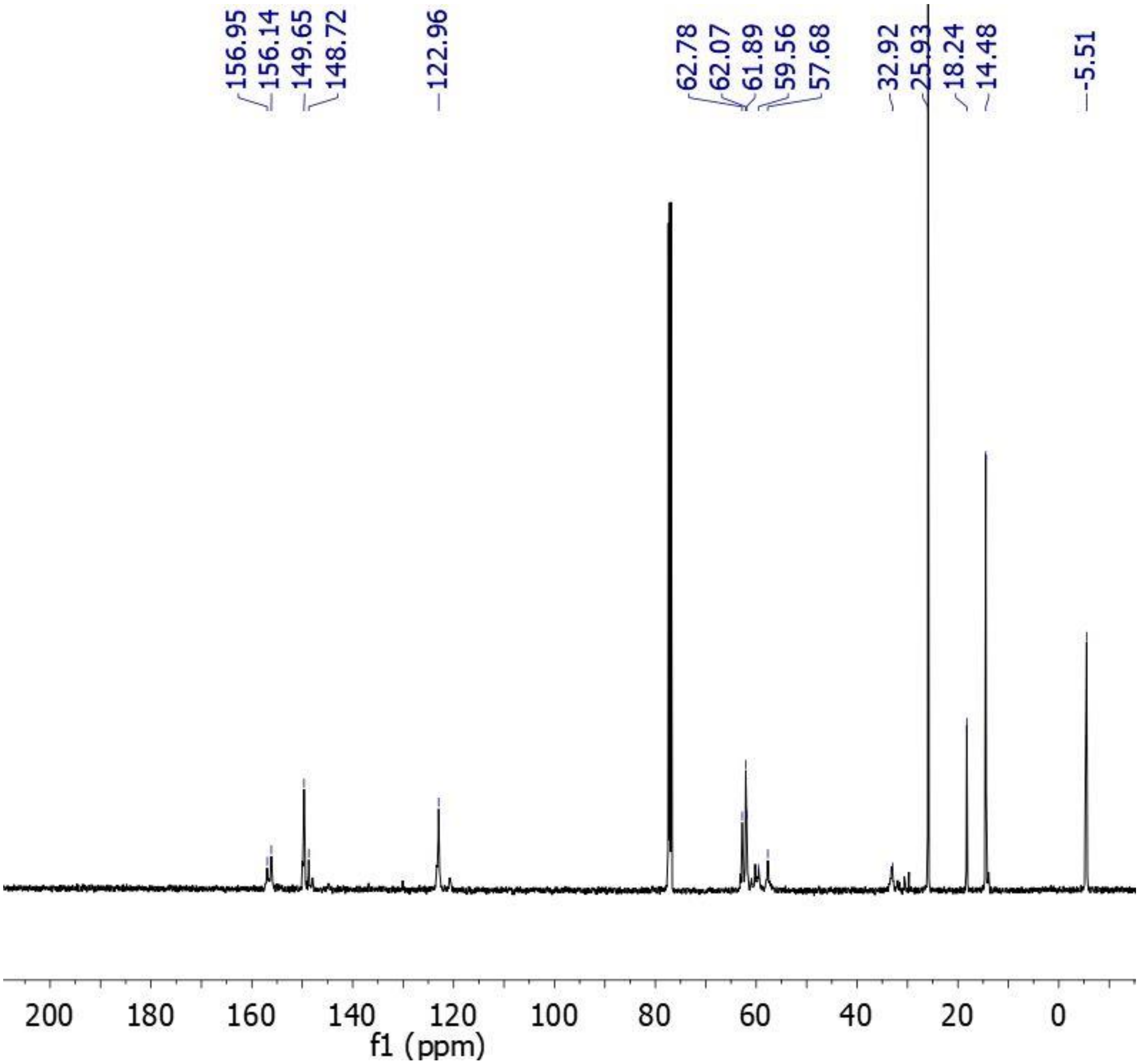

Figure S53: ^{13}C NMR of **20**

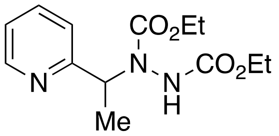

Compound 20: Amination was accomplished using procedure **D**. Column chromatography over silica gel (1:1 hexane:EtOAc) afforded 44.5 mg (0.16 mmol, 32%) of a colorless oil.

^{1}H NMR ($CDCl_3$) δ = 8.51-8.46 (m, 1H), 7.66-7.60 (m, 1H), 7.21-7.13 (m, 2H), 5.55-5.32 (m, 1H), 4.33-4.07 (m, 4H), 1.55-1.46 (m, 3H), 1.32-1.08 (m, 6H).). ^{13}C NMR ($CDCl_3$) δ = 161.42, 156.88, 149.16, 148.84, 136.78, 122.33, 121.53, 63.73, 62.56, 61.78, 17.79, 14.58, 14.49. HRMS m/z calcd. for $C_{13}H_{19}N_3O_4$ $[M+H]^+$ 282.1448, found 282.1450

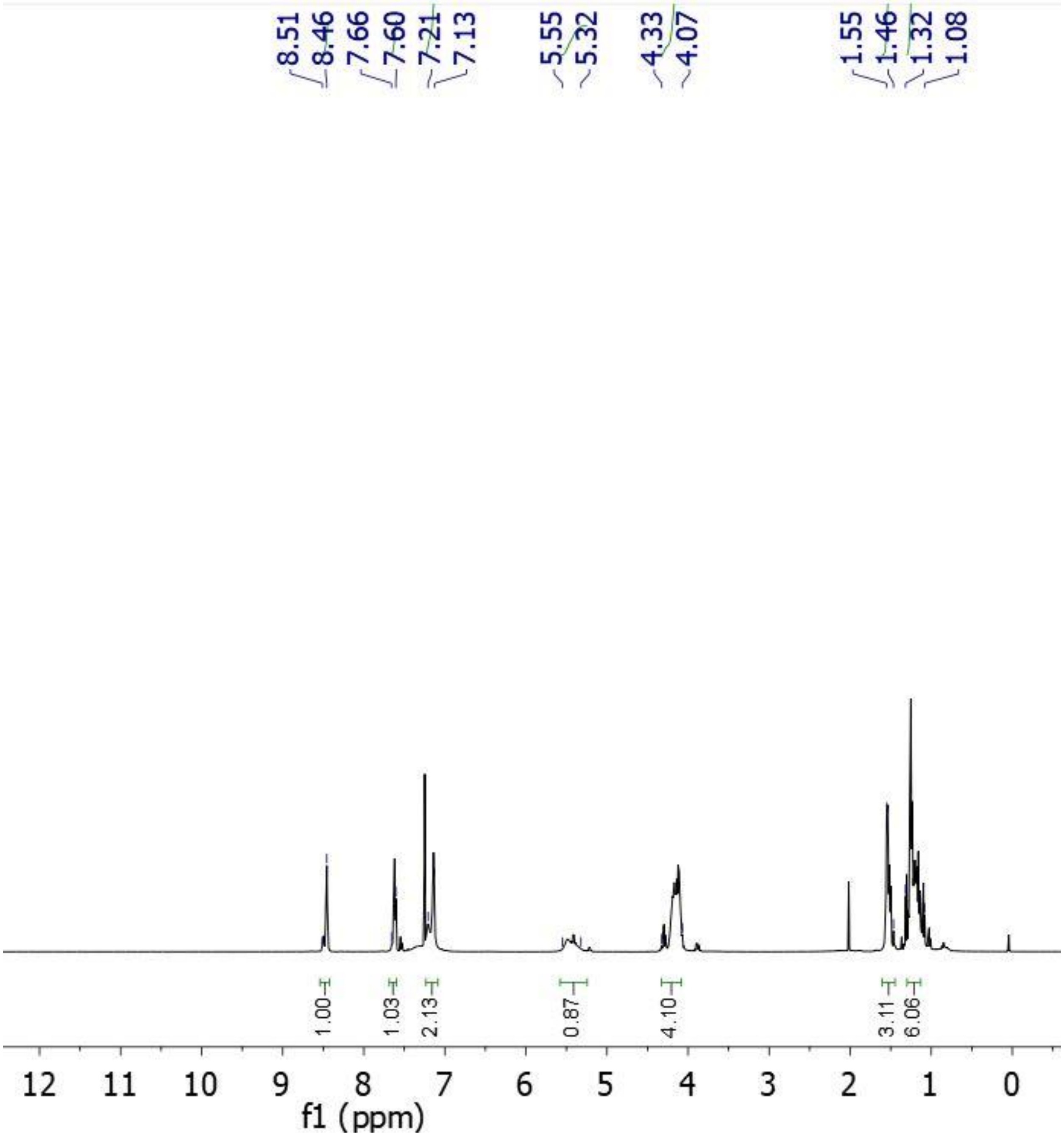

Figure S54: ^{1}H NMR of **21**

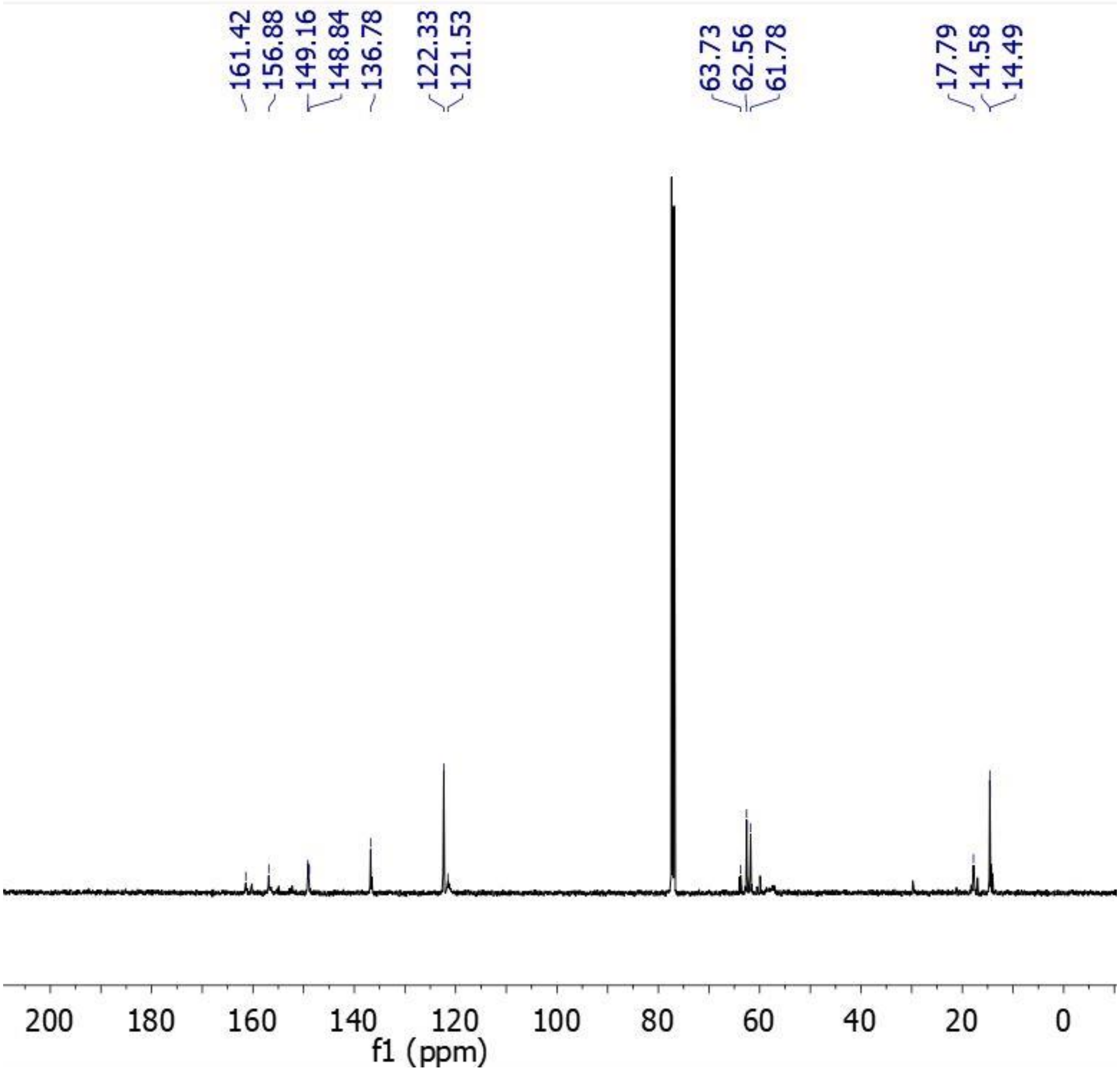

Figure S55: ^{13}C NMR of **21**

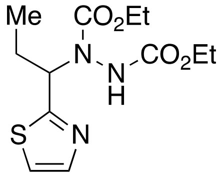

Compound 6: Amination was accomplished using procedure **D**. Column chromatography over silica gel (1:1 hexane:EtOAc) afforded 70.2 mg (0.23 mmol, 47%) of a colorless oil.

^{1}H NMR ($CDCl_3$) δ = 7.67 (d, *J* = 3.3 Hz, 1H), 7.26 (d, *J* = 3.4 Hz, 1H), 6.76 (s, 1H), 5.56-5.28 (m, 1H), 4.16 (q, *J* = 7.0 Hz, 4H), 2.08-1.96 (m, 2H), 1.23 (d, *J* = 5.2Hz, 6H), 1.05-1.08 (m, 3H). ^{13}C NMR ($CDCl_3$) δ = 169.20, 166.94, 156.57, 142.31, 119.40, 64.05, 63.01, 62.24, 29.76, 25.74, 14.49, 11.16. HRMS m/z calcd. for $C_{12}H_{19}N_3O_4S$ $[M+H]^+$ 302.1169, found 302.1163.

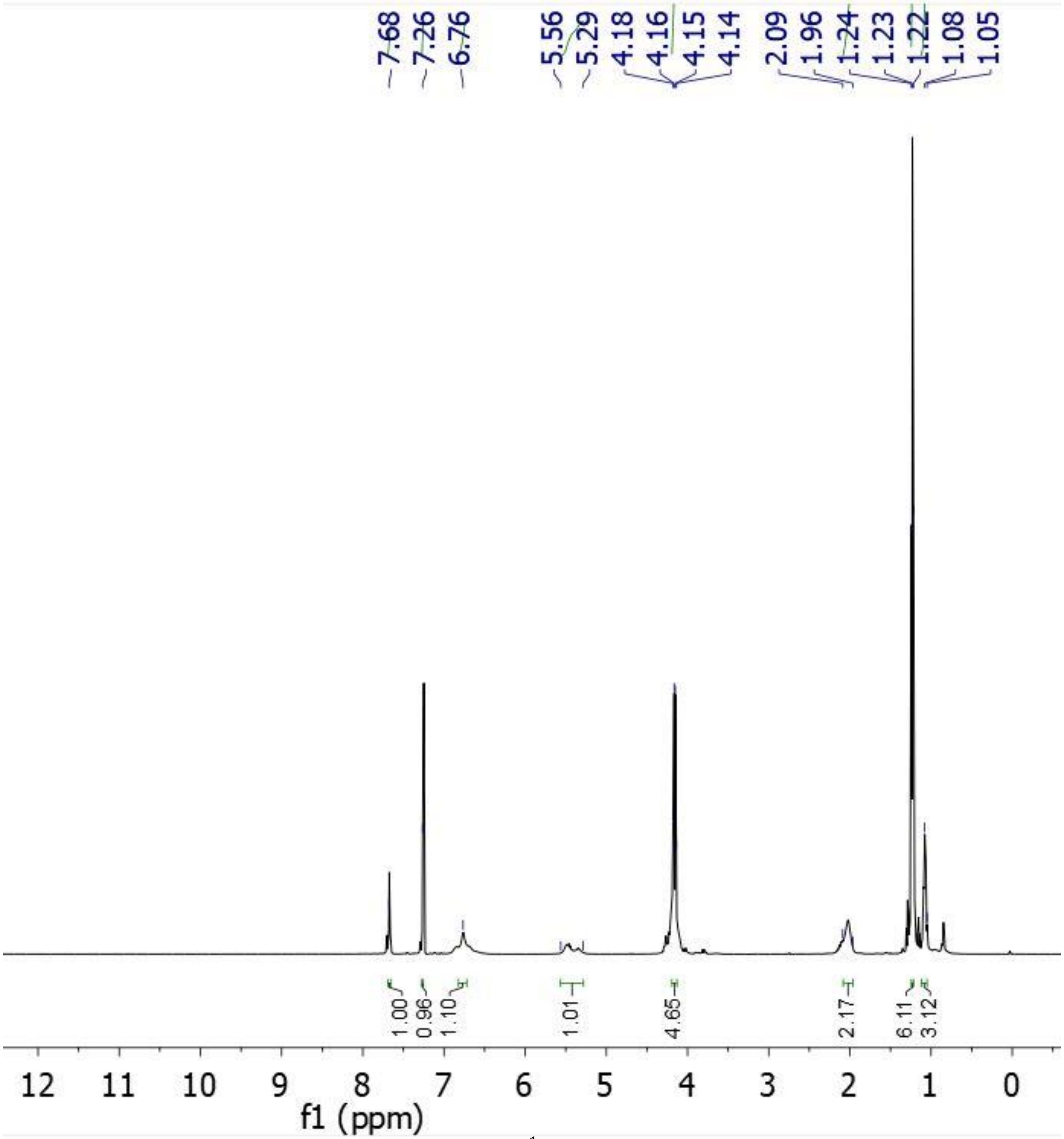

Figure S56: ^{1}H NMR of **6**

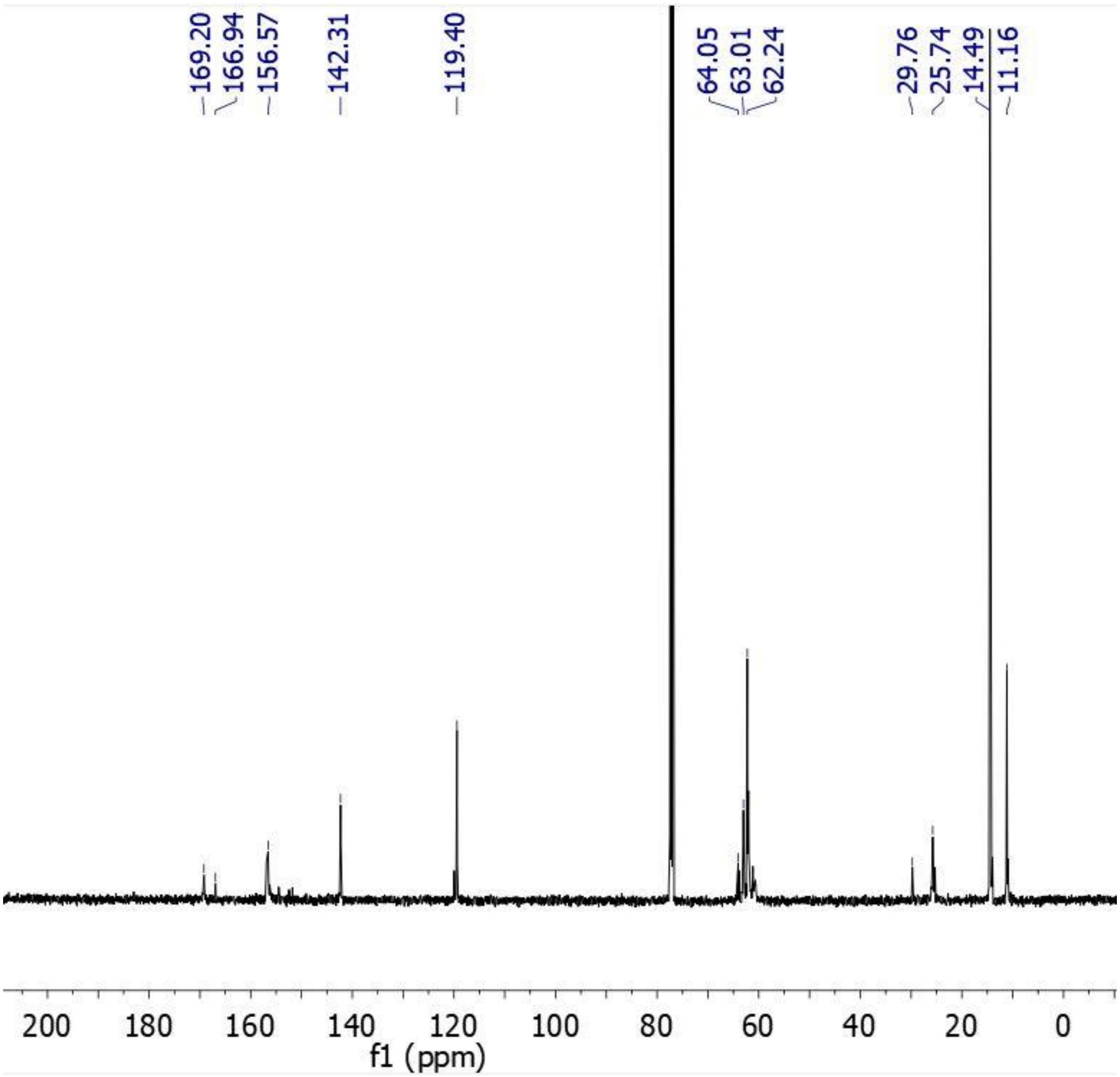

Figure S57: ^{13}C NMR of **6**

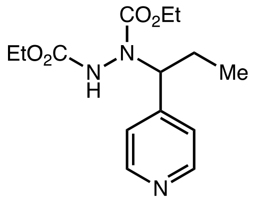

Compound SI-2: Amination was accomplished using procedure **A**. Column chromatography (30 % ethyl acetate in hexanes) afforded 103.4 mg (0.35 mmol, 70%) of a clear oil. ^{1}H NMR ($CDCl_3$) δ = 8.51 (br s, 2H), 7.65 – 7.2 (br, 2H), 6.85 – 6.15 (br, 1H), 5.40 – 4.90 (br s, 1H), 4.37 – 3.97 (br s, 4H) 2.10 –1.60 (br, 2H), 1.35 – 1.15 (br s, 6H), 0.97 (br t, J = 6.5 Hz, 3H). ^{13}C NMR ($CDCl_3$) δ = 156.92, 156.07, 149.60, 148.91, 123.31, 63.31, 62.89, 62.18, 23.38, 14.53, 10.99. $[M+H]^+$ 296.1605, found 296.1602.

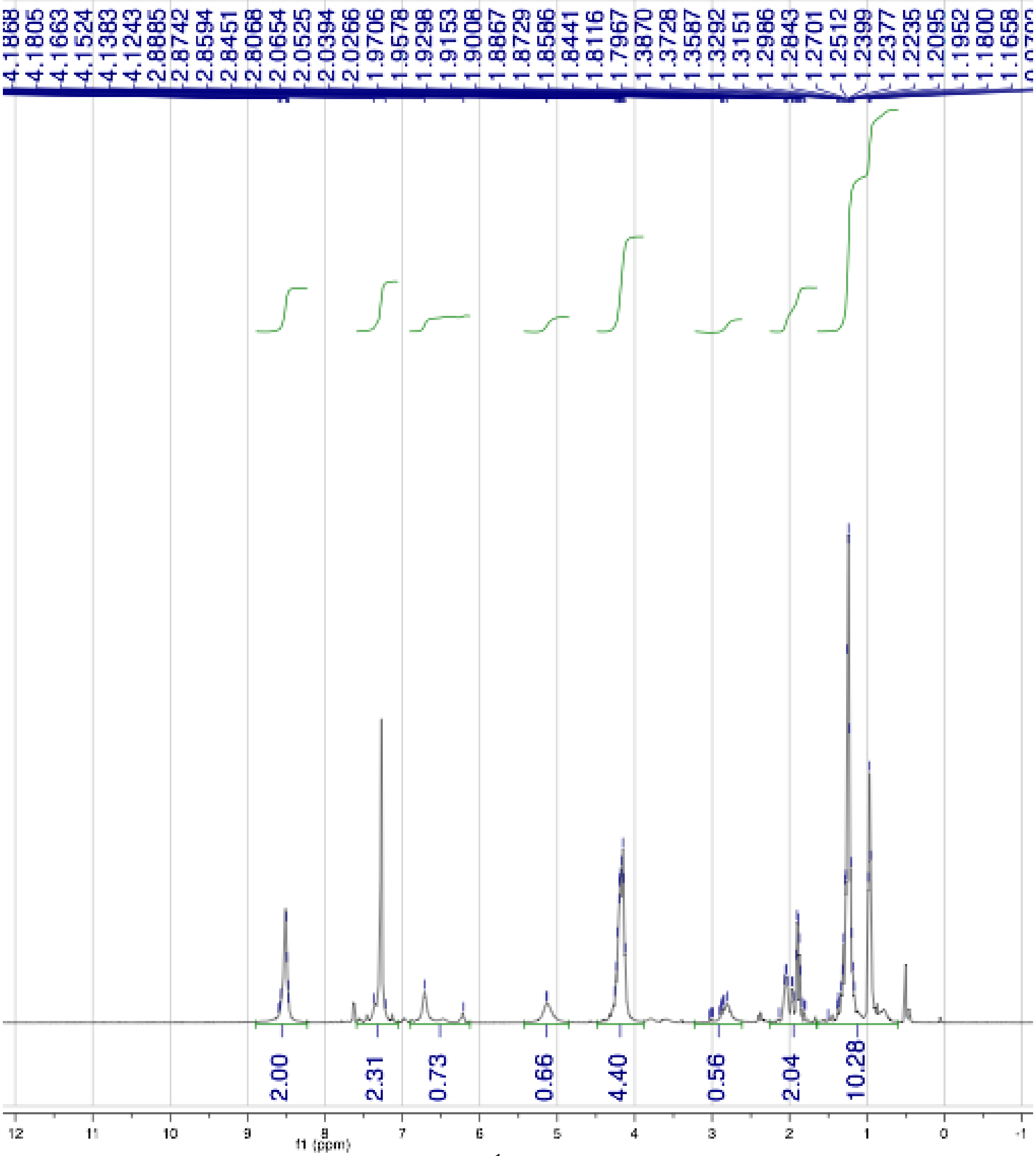

Figure S58. ^{1}H NMR of **SI-2**.

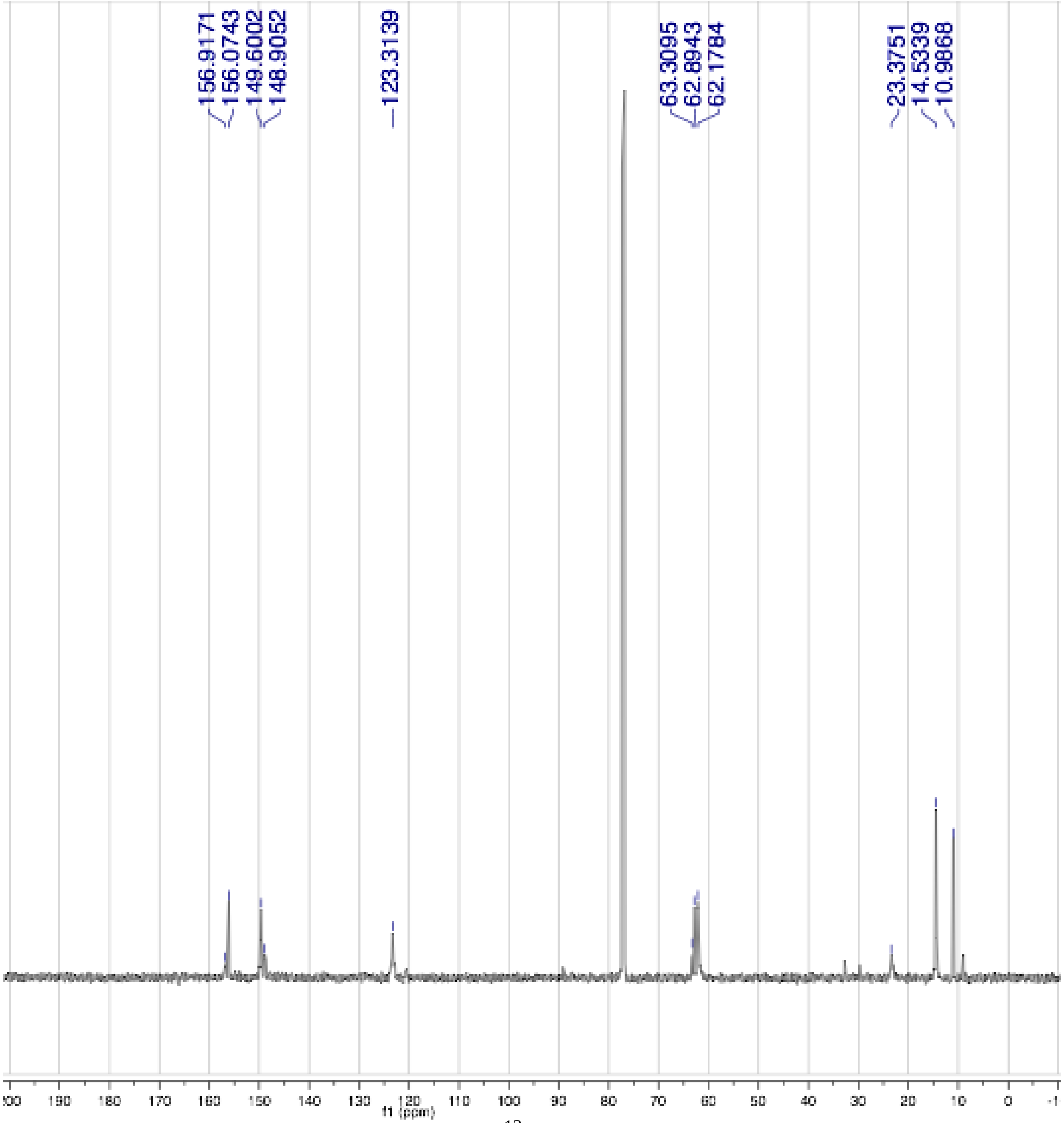

Figure S59. ^{13}C NMR of **SI-2**.

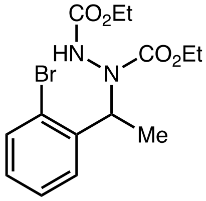

Compound SI-3: So that a gas chromatography conversion factor could be obtained, the authentic 2-ethyl-1-bromobenzene product was synthesized with a modification of the procedure of Inoue.[12] An oven-dried 8 mL vial under N_2 was charged with N-hydroxyphthalimide (49 mg, 0.3 mmol, 0.2 equiv), anhydrous ethyl acetate (5 mL), 2-ethyl-1-bromobenzene (210 µL, 1.5 mmol, 1 equiv), and DEAD (470 µL 3 mmol, 2 equiv). The reaction mixture was set

heating to 80°C and stirred for 48 hours. The excess solvent was removed *in vacuo* and column chromatography over silica gel (gradient elution of 4:1 to 2:1 hexane:EtOAc) afforded 86 mg of a clear oil.

^{1}H NMR (400 MHz, Chloroform-*d*) ∂ 7.57 (dd, *J* = 8.0, 1.2 Hz, 1H), 7.44 (s, 1H), 7.31 (t, *J* = 7.5 Hz, 1H), 7.15 (td, *J* = 7.8, 1.6 Hz, 1H), 5.6–6.3 (br, 2H), 4.19 (br, 4H), 1.57 (d, *J* = 6.2 Hz, 3H), 1.25 (t, *J* = 7.1 Hz, 6H). ^{13}C NMR (101 MHz, $CDCl_3$) ∂ 156.78, 155.43, 133.06, 129.06, 128.64, 127.39, 62.50, 62.02, 56.93, 17.45, 14.44, 14.36. Two aromatic carbon resonances are unobserved, and may be coincident or significantly broadened due to rotamers associated with the two carbamates. HRMS m/z calcd. for $C_{14}H_{20}BrN_2O_4$ $[M+H]^+$ 359.0606, found 359.0591.

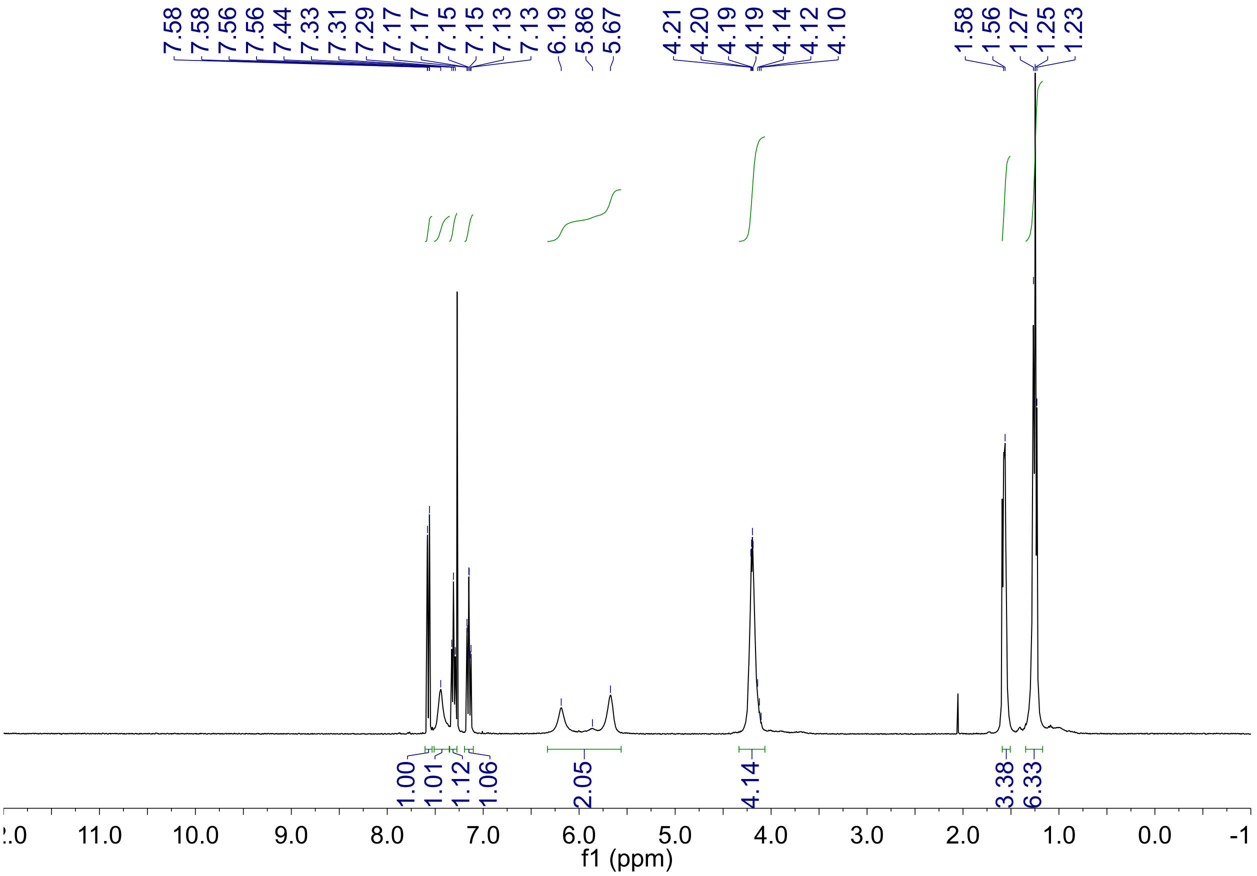

Figure S60: ^{1}H NMR of **SI-3**

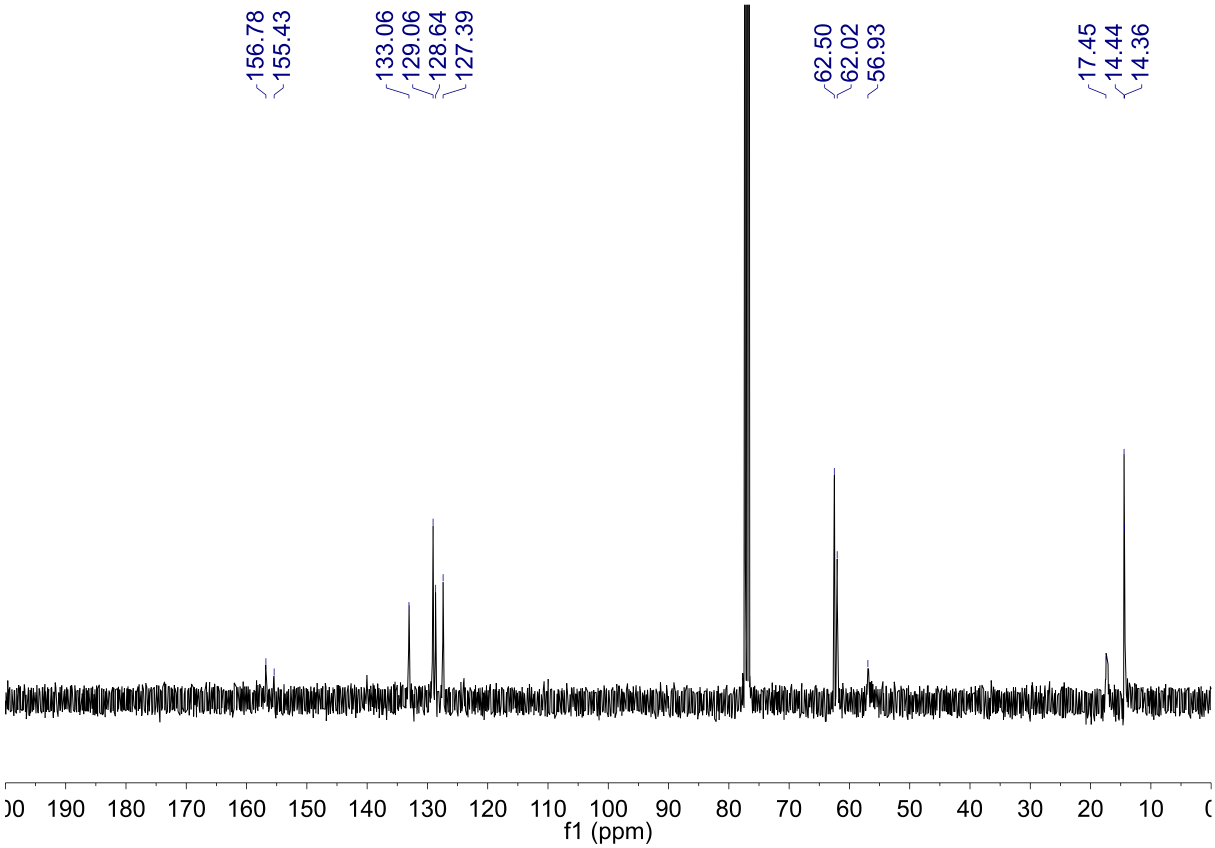

Figure S61: ^{13}C NMR of **SI-3**

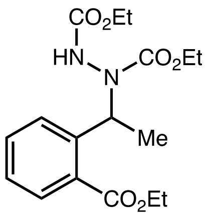

Compound SI-4: So that a gas chromatography conversion factor could be obtained, the authentic ethyl 2-ethylbenzoate product was synthesized with a modification of the procedure of Inoue.[12] An oven-dried 8 mL vial under N_2 was charged with N-hydroxyphthalimide (6.4 mg, 0.04 mmol, 0.2 equiv), anhydrous ethyl acetate (650 µL), 2-ethyl-1-bromobenzene (36 mg, 0.2 mmol, 1 equiv), and DEAD (61 µL 0.4 mmol, 2 equiv). The reaction mixture was set heating to 80°C and stirred for 48 hours. The excess solvent was removed *in vacuo* and column chromatography over silica gel (gradient elution of 2:1 hexane:EtOAc to 100% EtOAc) afforded 23 mg of a clear oil containing the desired product. ^{1}H NMR (400 MHz, Chloroform-*d*) δ 7.73 (d, *J* = 7.6 Hz, 1H), 7.52 (br, 1H), 7.47 (t, *J* = 7.0 Hz, 1H), 7.37-7.28 (m, 1H), 4.37 (q, *J* = 7.1 Hz, 2H), 6.5-5.9 (br, 2H), 4.11 (q, *J* = 7.1 Hz, 2H), 1.60 (d, *J* = 6.8 Hz, 3H), 1.39 (t, *J* = 7.1 Hz, 2H), 1.27 (t, *J* = 6.8 Hz, 3H), 1.17 (t, *J* = 6.9 Hz, 3H). ^{13}C NMR (101 MHz, $CDCl_3$) δ 168.20, 156.86, 155.63, 131.46, 130.67, 129.68, 127.17, 62.38, 62.03, 61.32, 54.25, 18.09, 14.43, 14.33, 14.21. Two aromatic carbon resonances are unobserved, and may be coincident or significantly broadened due to rotamers associated with the two carbamates. HRMS m/z calculated for $C_{17}H_{25}N_2O_6$ $[M+H]^+$ 353.1713, found 353.1714.

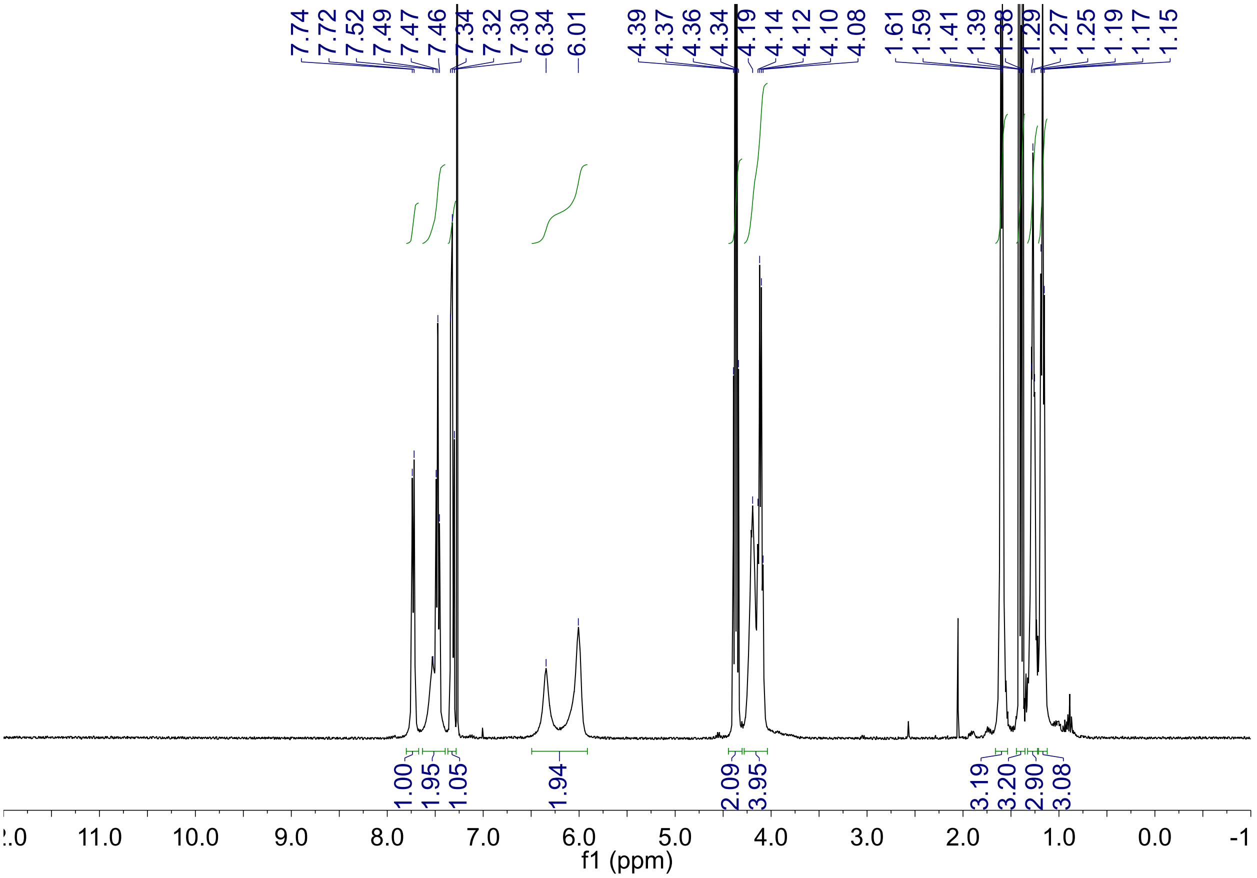

Figure S62: ^{1}H NMR of **SI-4**

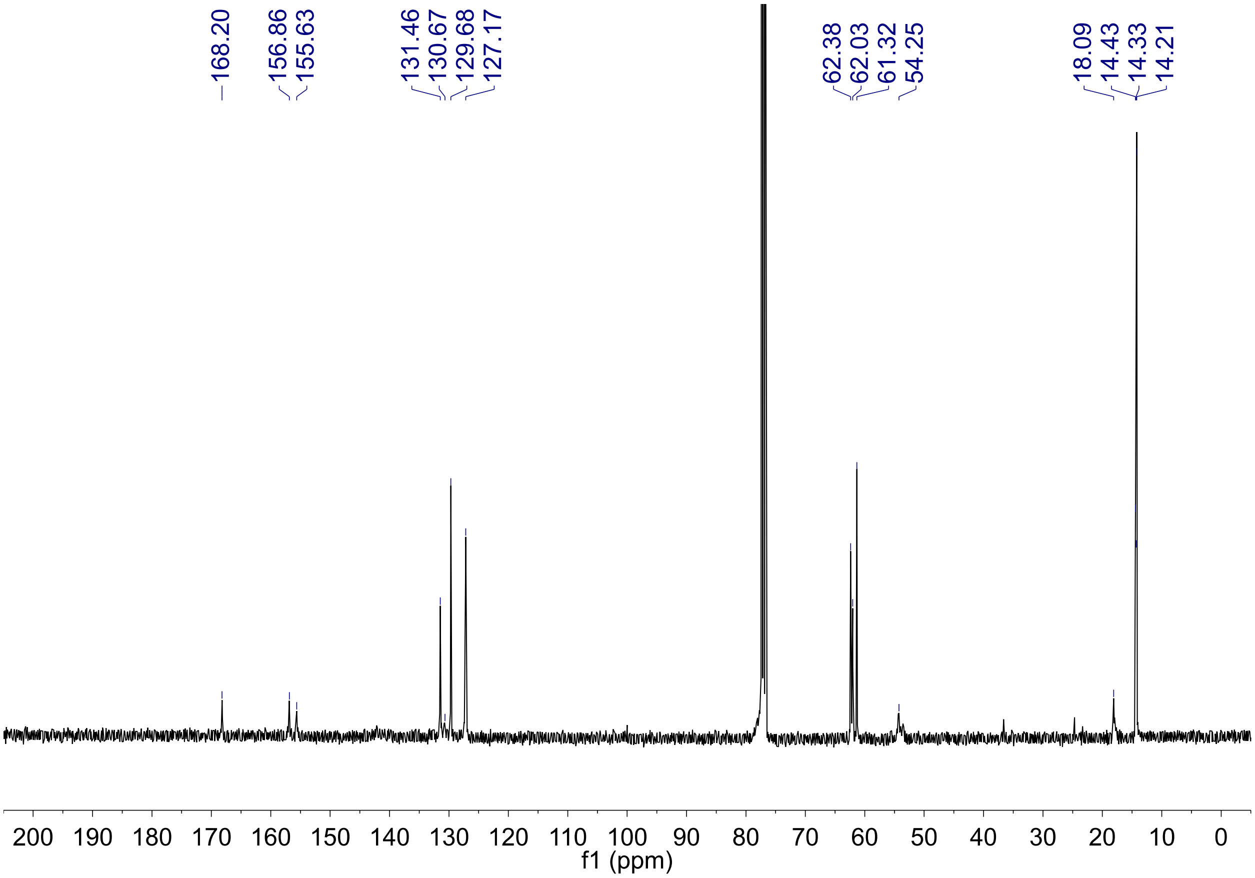

Figure S63: ^{13}C NMR spectrum of **SI-5**

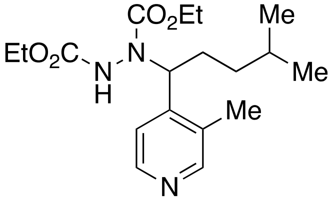

Compound 21: Amination was accomplished using procedure **A**. Column chromatography over silica gel (2.5:1 hexane:EtOAc) afforded 124.5 mg (0.29 mmol, 57%) of a yellow oil.

^{1}H NMR ($CDCl_3$) δ = 8.31-8.25 (m, 2H), 7.19-7.06 (m, 2H), 5.40 (bs, 1H), 4.18-4.06 (m, 4H), 2.36-2.31 (m, 3H), 2.06-1.97 (m, 1H), 1.84-1.76 (m, 1H), 1.57-1.49 (m, 1H), 1.33-1.07 (m, 8H), 0.85-0.83 (m, 6H). ^{13}C NMR ($CDCl_3$) δ = 156.76, 155.82, 151.01, 150.79, 147.19, 146.20, 121.90, 62.70, 61.92, 56.05, 35.08, 28.64, 27.99, 22.66, 22.47, 16.41, 14.54, 13.73. HRMS m/z calcd. for $C_{18}H_{29}N_3O_4$ $[M+H]^+$ 352.2219 found 352.2215.

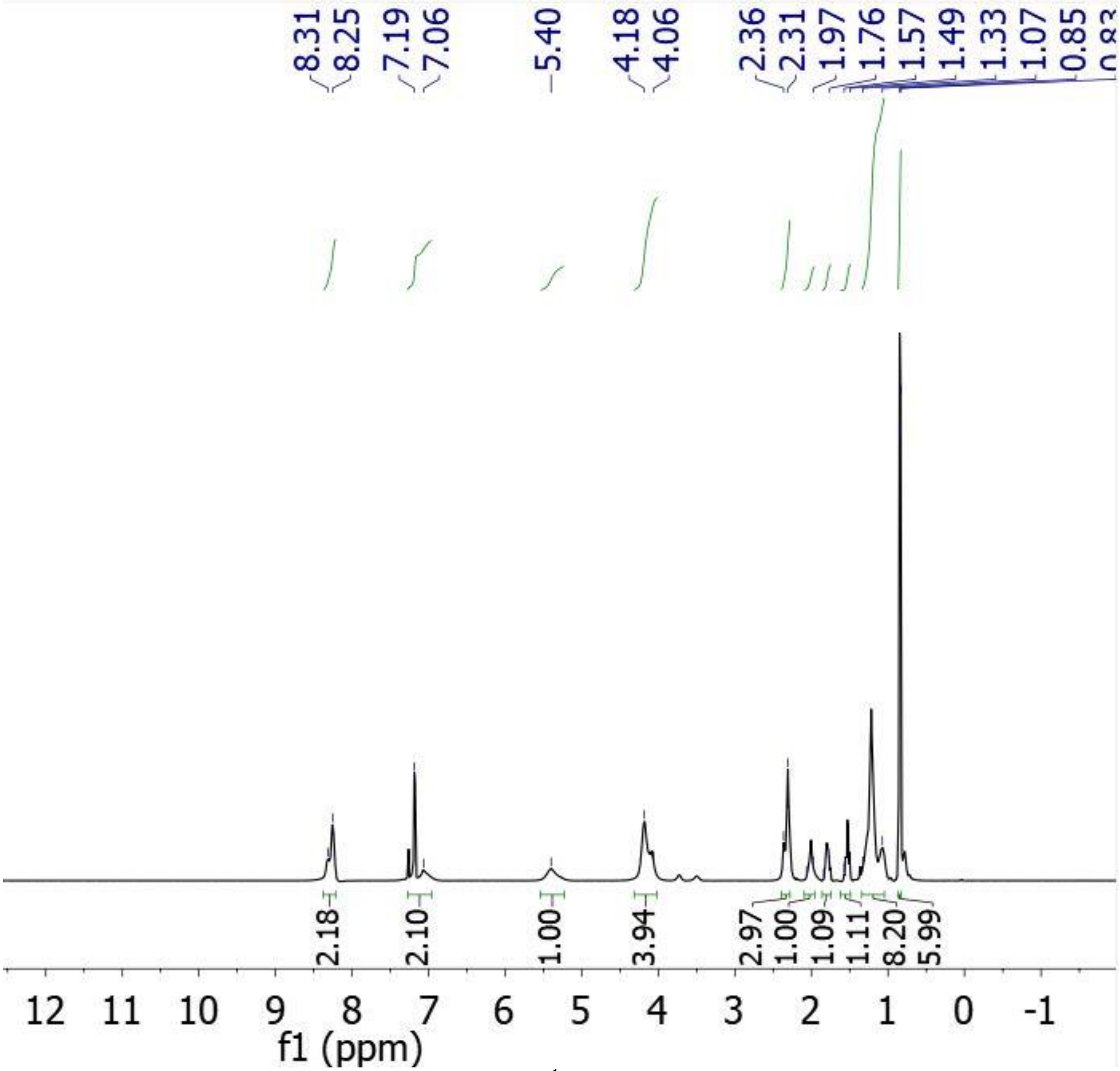

Figure S64: ^{1}H NMR of **21**

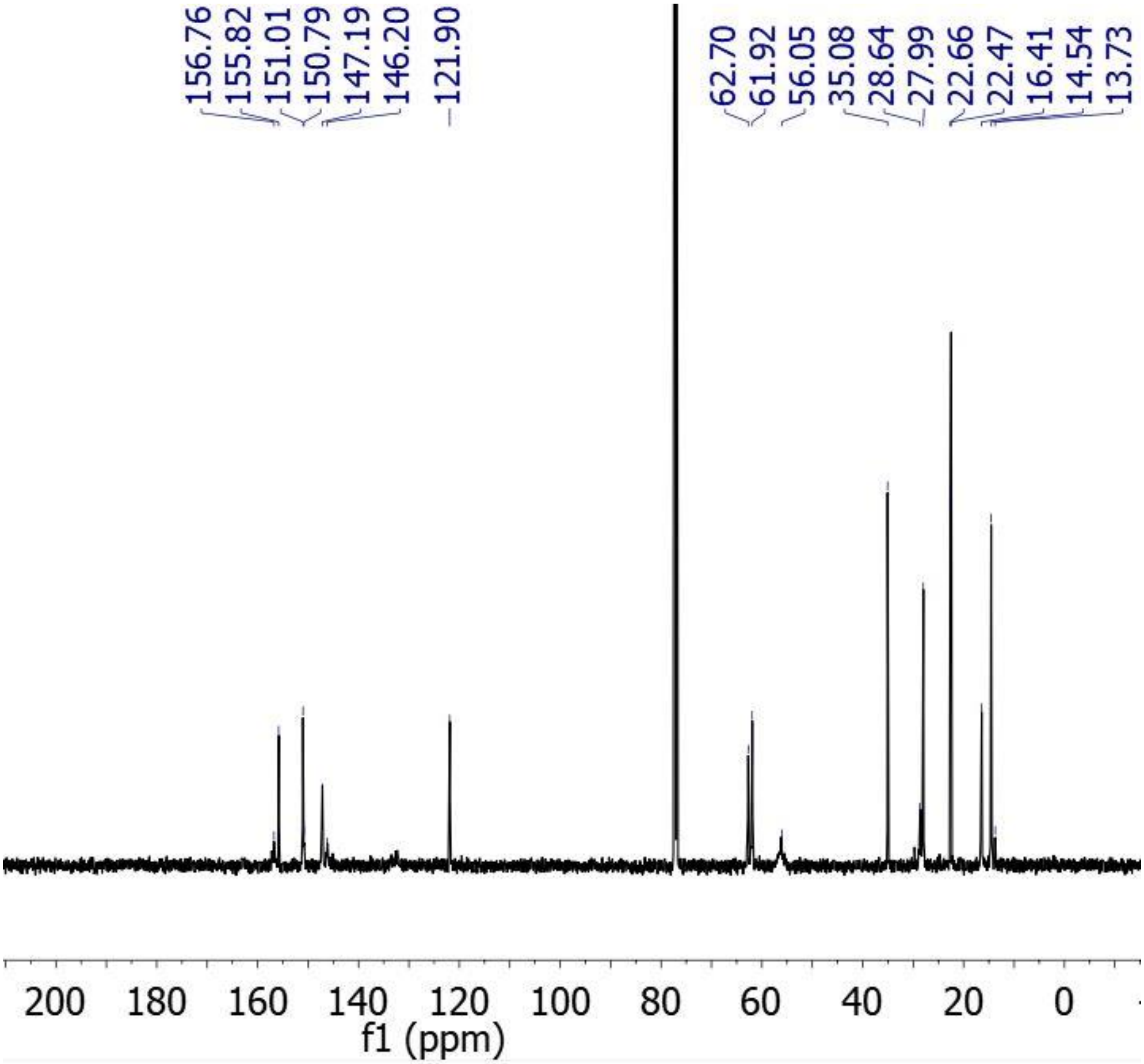

Figure S65: ^{13}C NMR of **21**

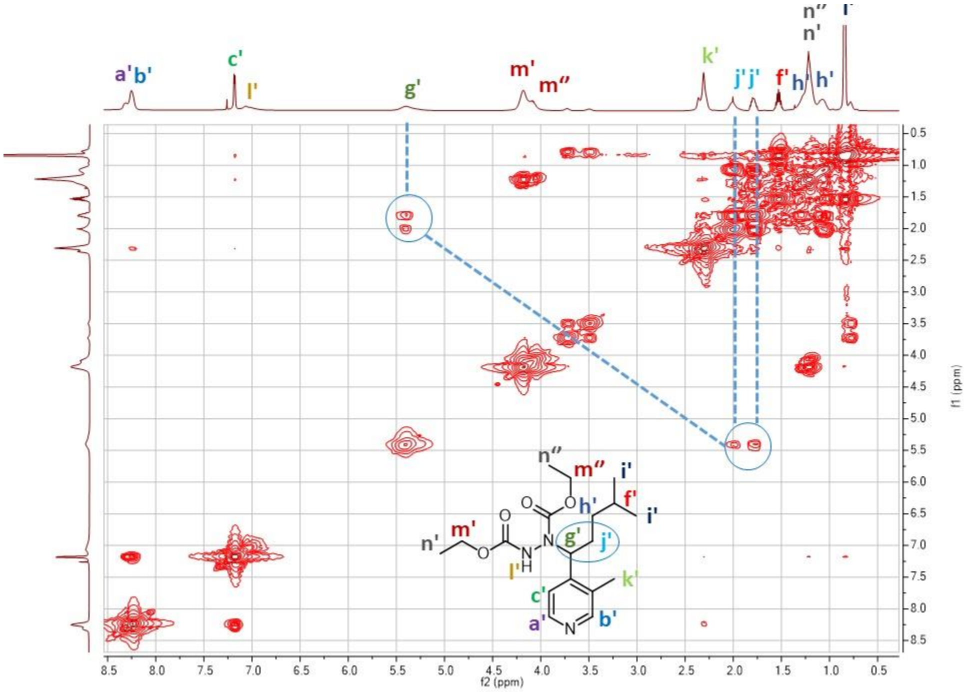

Figure S66: Correlation of g' and j' by ^{1}H-^{1}H COSY of **21**

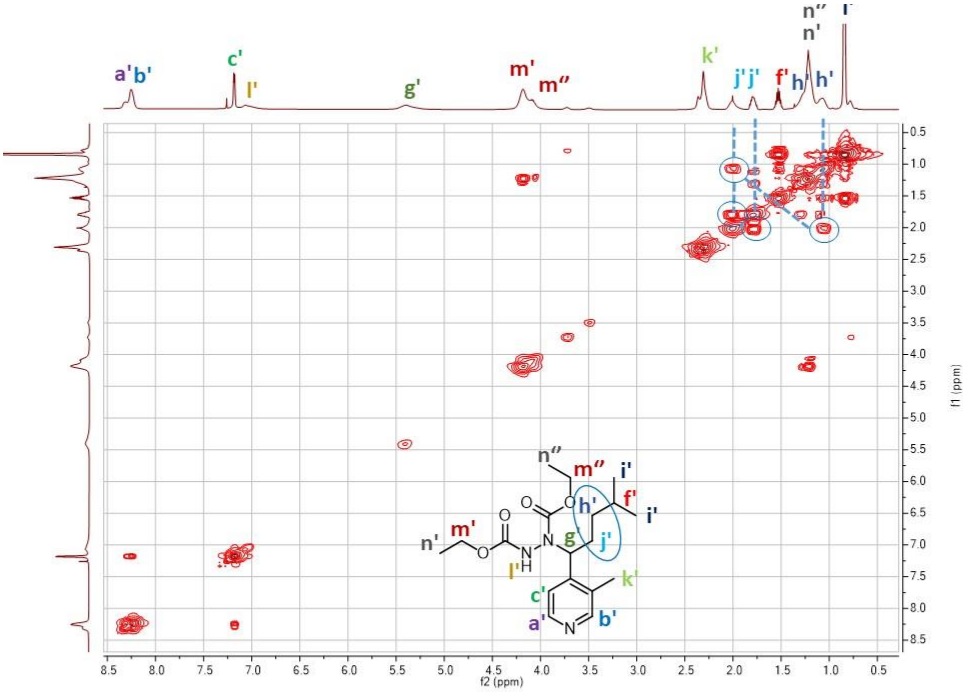

Figure S67: Correlation of j' and h' by ^{1}H-^{1}H COSY of **21**

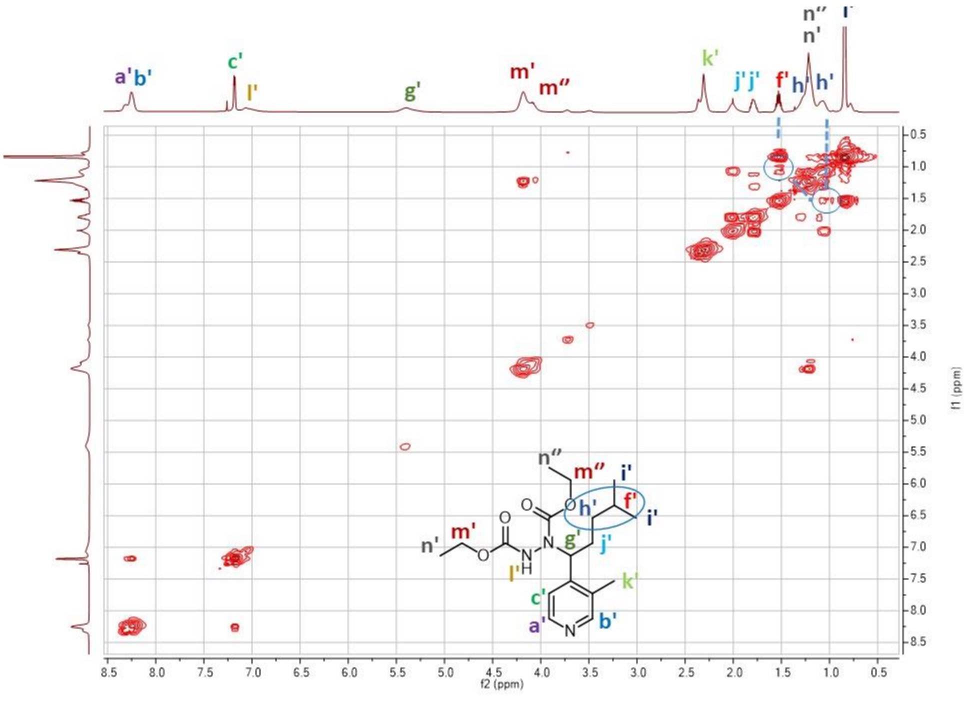

Figure S68: Correlation of j’ and h’ by ^{1}H-^{1}H COSY of **21**

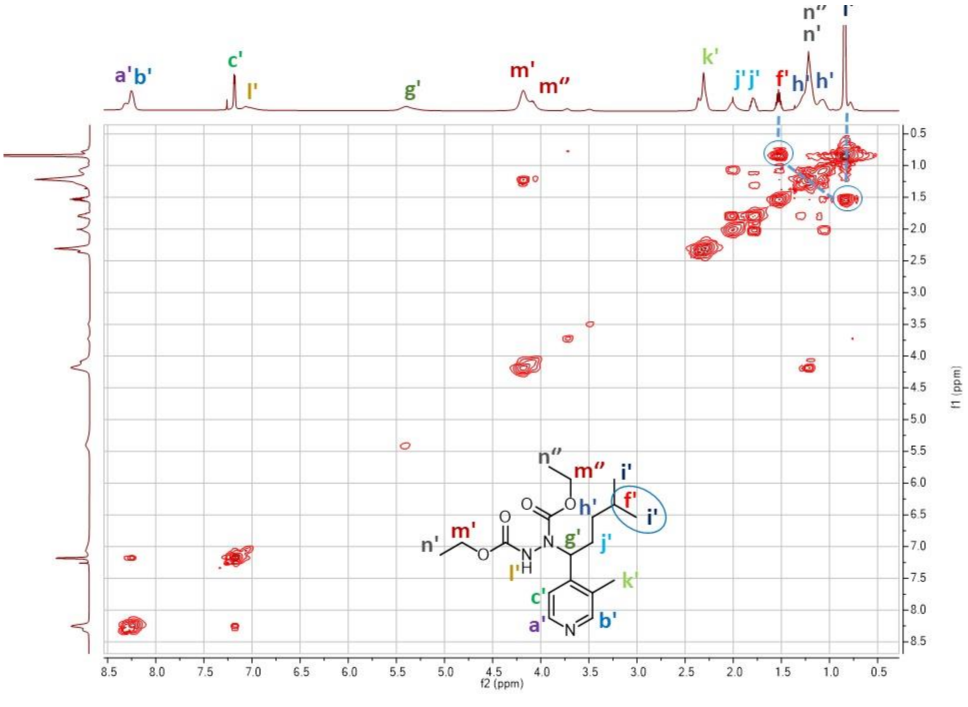

Figure S69: Correlation of f’ and i’ by ^{1}H-^{1}H COSY of **21**

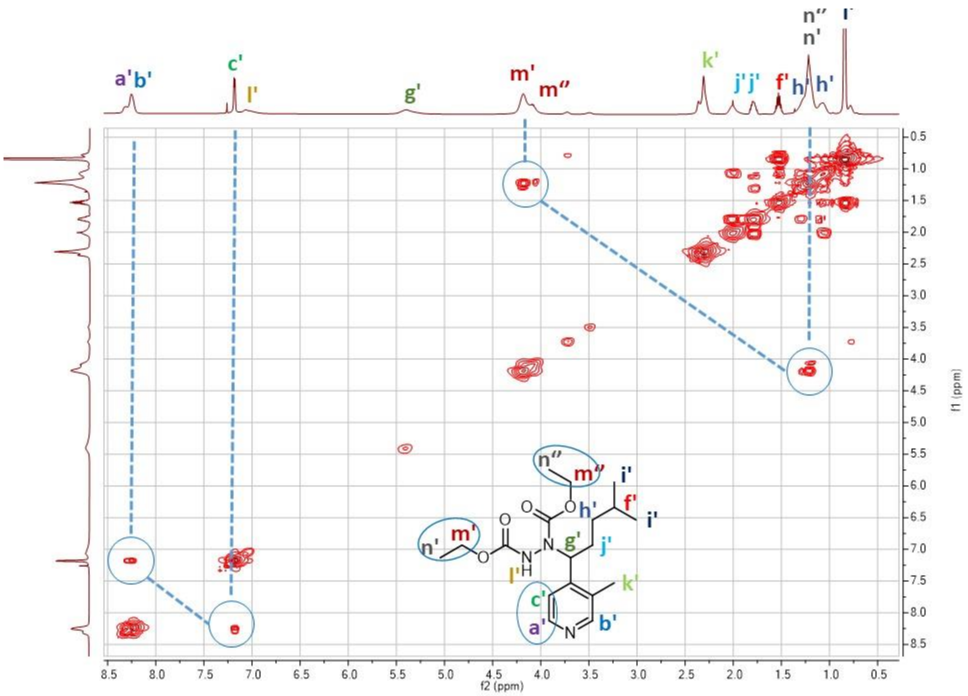

Figure S70: Correlation of a' and c' and correlation of m'/m" and n'/n" by ^1H-^1H COSY of **21**

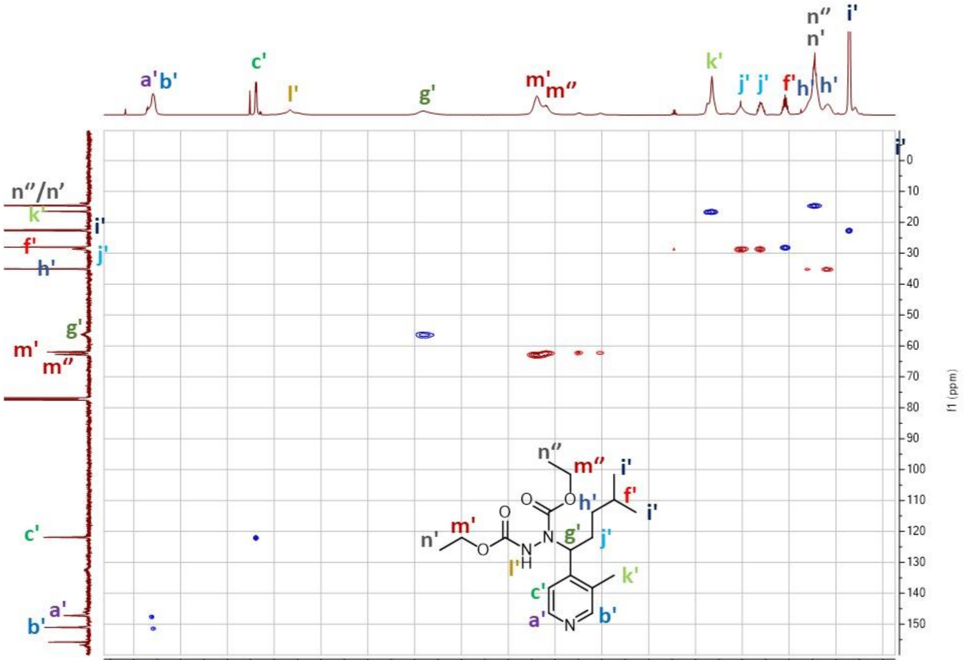

Figure S71: HSQC of **21**

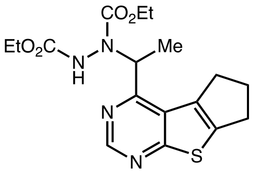

Compound 22: Amination was accomplished using procedure **B**. Column chromatography over silica gel (10:1 EtOAc:dichloromethane) afforded 233 mg (0.40 mmol, 80%) of a light yellow solid.

^{1}H NMR ($CDCl_3$) δ = 8.82 – 8.65 (m, 1H), 7.83 – 7.50 (m, 1H), 5.91 – 5.64 (m, 1H), 4.26 – 3.94 (m, 4H), 3.18 – 2.94 (m, 4H), 2.49 (m, 2H), 1.51 – 1.46 (m, 3H), 1.31 – 1.19 (m, 3H), 1.20 – 1.11 (m, 3H). ^{13}C NMR ($CDCl_3$) δ = 173.67, 163.47, 163.23, 156.52, 156.26, 151.63, 144.18, 144.11, 135.24, 135.03, 124.31, 77.36, 62.53, 62.45, 61.88, 61.82, 61.71, 55.60, 54.57, 54.38, 29.91, 29.51, 27.45, 18.14, 17.75, 14.54, 14.43, 14.35, 14.27. HRMS m/z calcd. for $C_{17}H_{22}N_4O_4$ $[M+H]^+$ 379.1435, found 379.1420

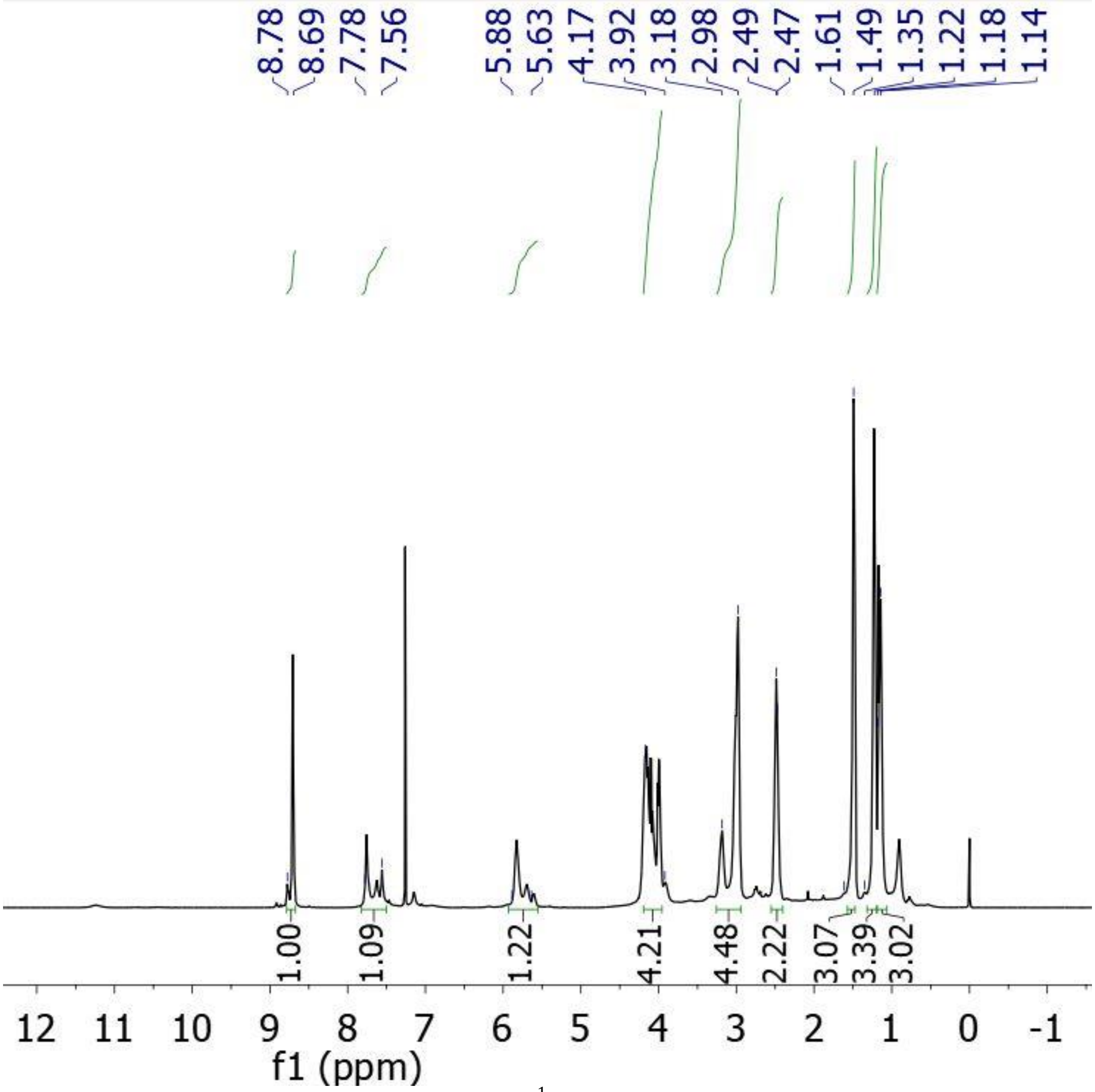

Figure S72: ^{1}H NMR of **22**

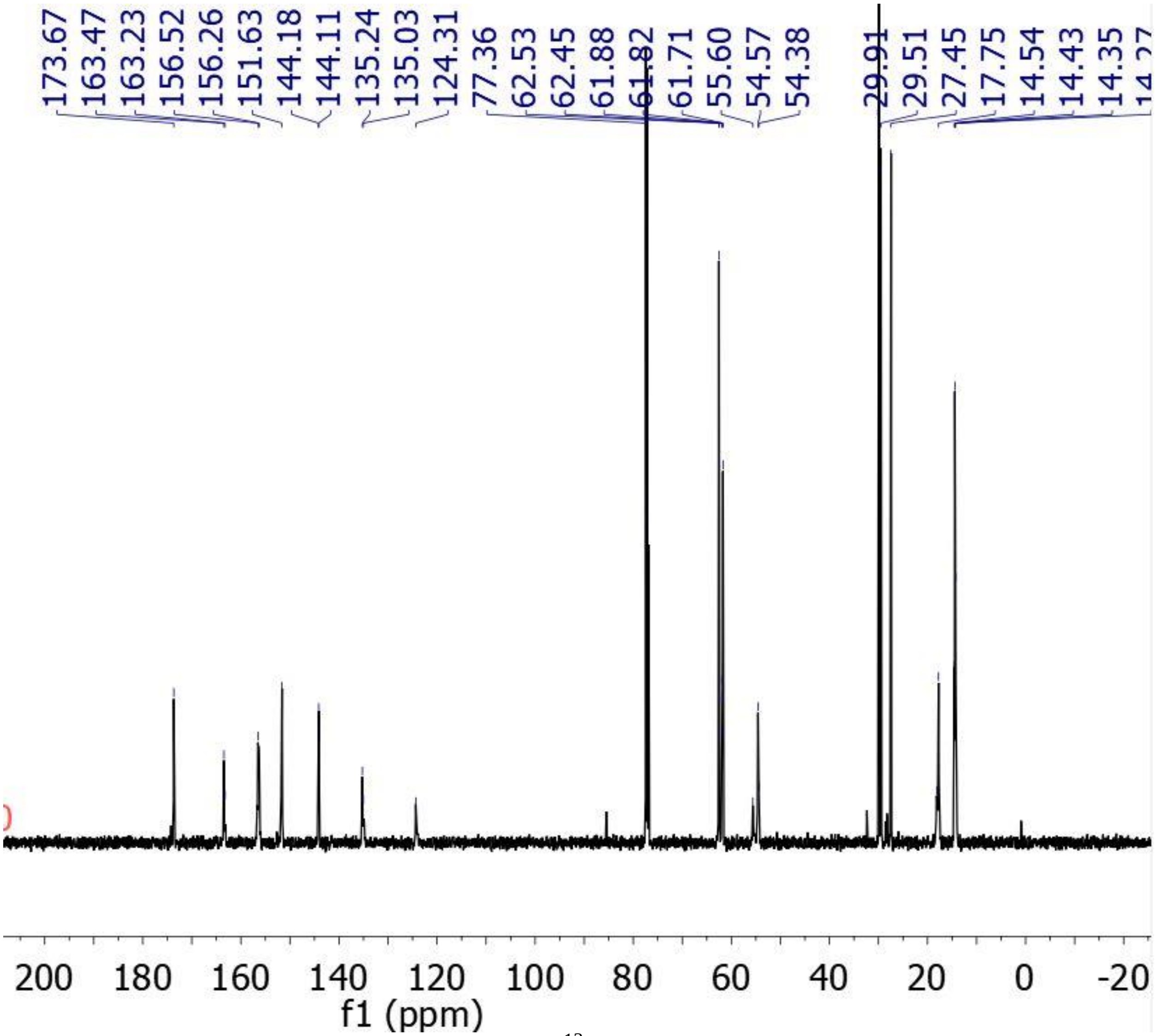

Figure S73: ^{13}C NMR of **22**

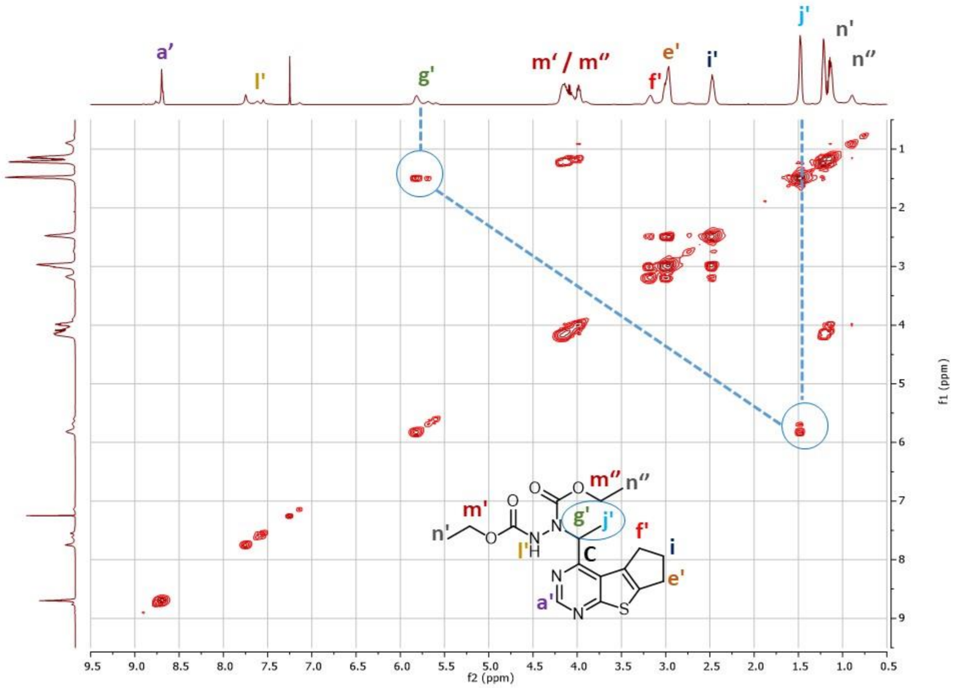

Figure S74: Correlation of g' and j' by ^{1}H-^{1}H COSY of **22**

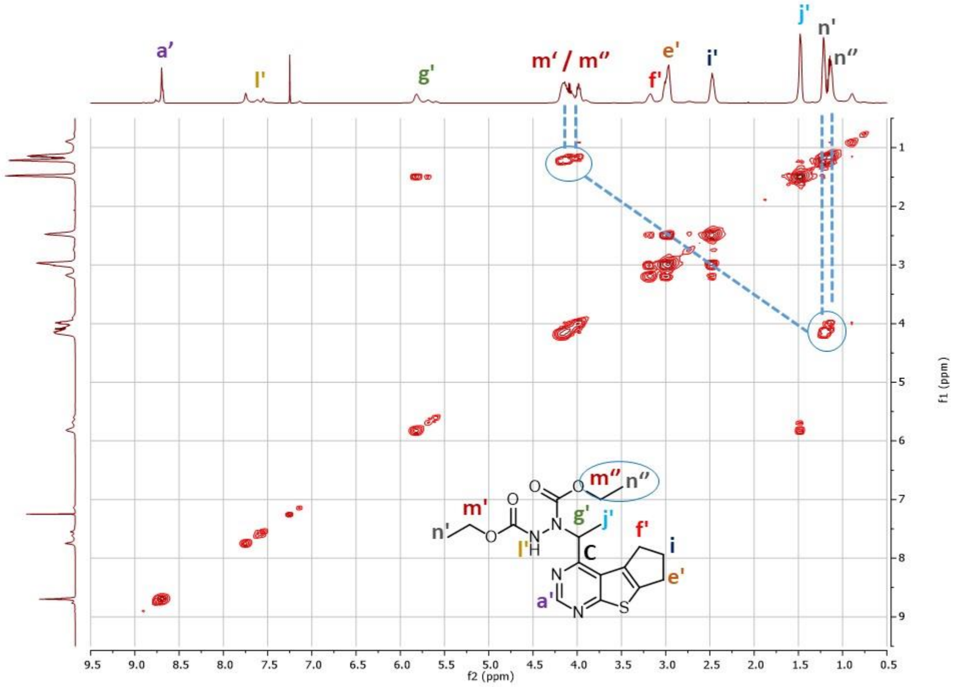

Figure S75: Correlation of m'/m'' and n'/n'' by ^{1}H-^{1}H COSY of **22**

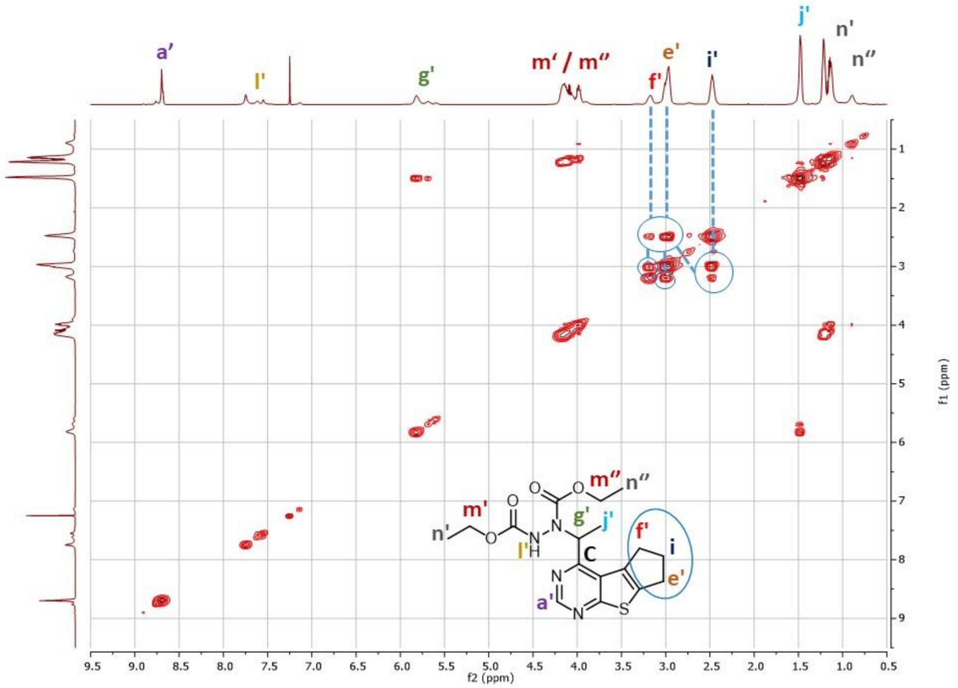

Figure S76: Correlation of f', e', and i' by ^1H-^1H COSY of **22**

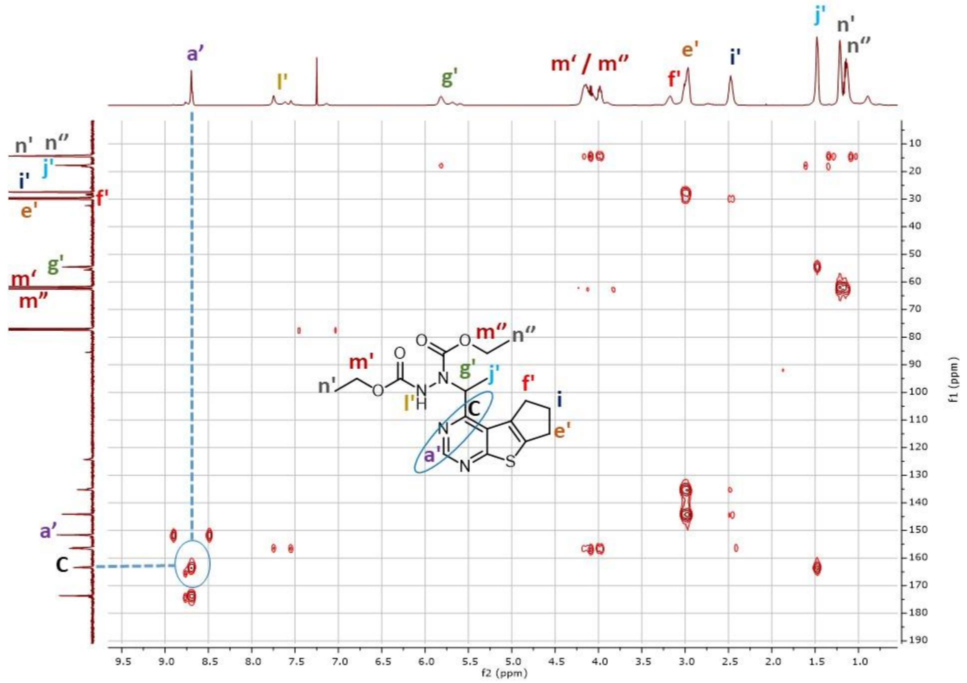

Figure S77: Correlation of a' and C by HMBC of **22**

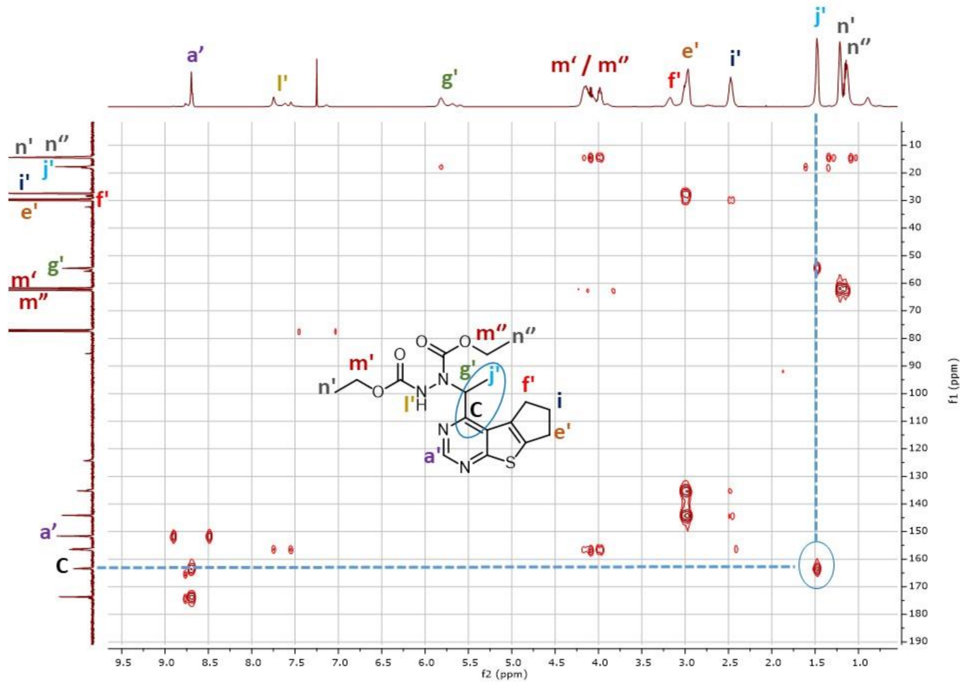

Figure S78: Correlation of j' and C by HMBC of **22**

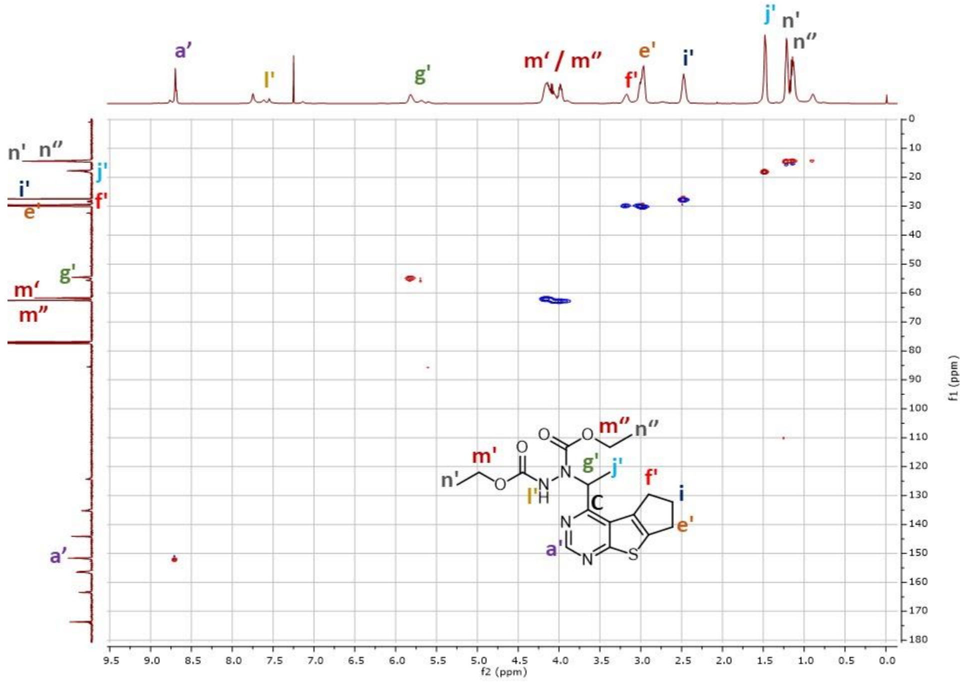

Figure S79: HSQC of **22**

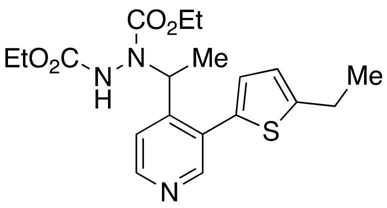

Compound 23: Amination was accomplished using procedure **A**. Column chromatography over silica gel (2:1 EtOAc:hexane) afforded 101 mg (0.26 mmol, 52%) of a yellow oil.

^{1}H NMR ($CDCl_3$) δ = 8.48 (s, 1H), 8.46 (d, *J* = 5.1 Hz, 1H), 7.46 (s, 1H), 6.94 (s, 1H), 6.86 (s, 1H), 6.77 (d, *J* = 3.6 Hz, 1H), 5.58 (s, 1H), 4.18 – 4.09 (m, 2H), 4.07 – 3.94 (m, 2H), 2.85 (q, *J* = 7.5 Hz, 2H), 1.44 (d, *J* = 6.9 Hz, 3H), 1.32 (t, *J* = 7.5 Hz, 3H), 1.22 (t, *J* = 7.2 Hz, 3H), 1.07 (s, 3H). ^{13}C NMR ($CDCl_3$) δ = 157.21, 155.47, 151.26, 149.82, 149.10, 134.41, 129.95, 127.82, 123.90, 121.28, 62.65, 62.21, 54.04, 23.48, 18.45, 15.94, 14.50, 14.42. HRMS m/z calcd. for $C_{19}H_{25}N_3O_4S$ $[M+H]^+$ 392.1639, found 392.1640

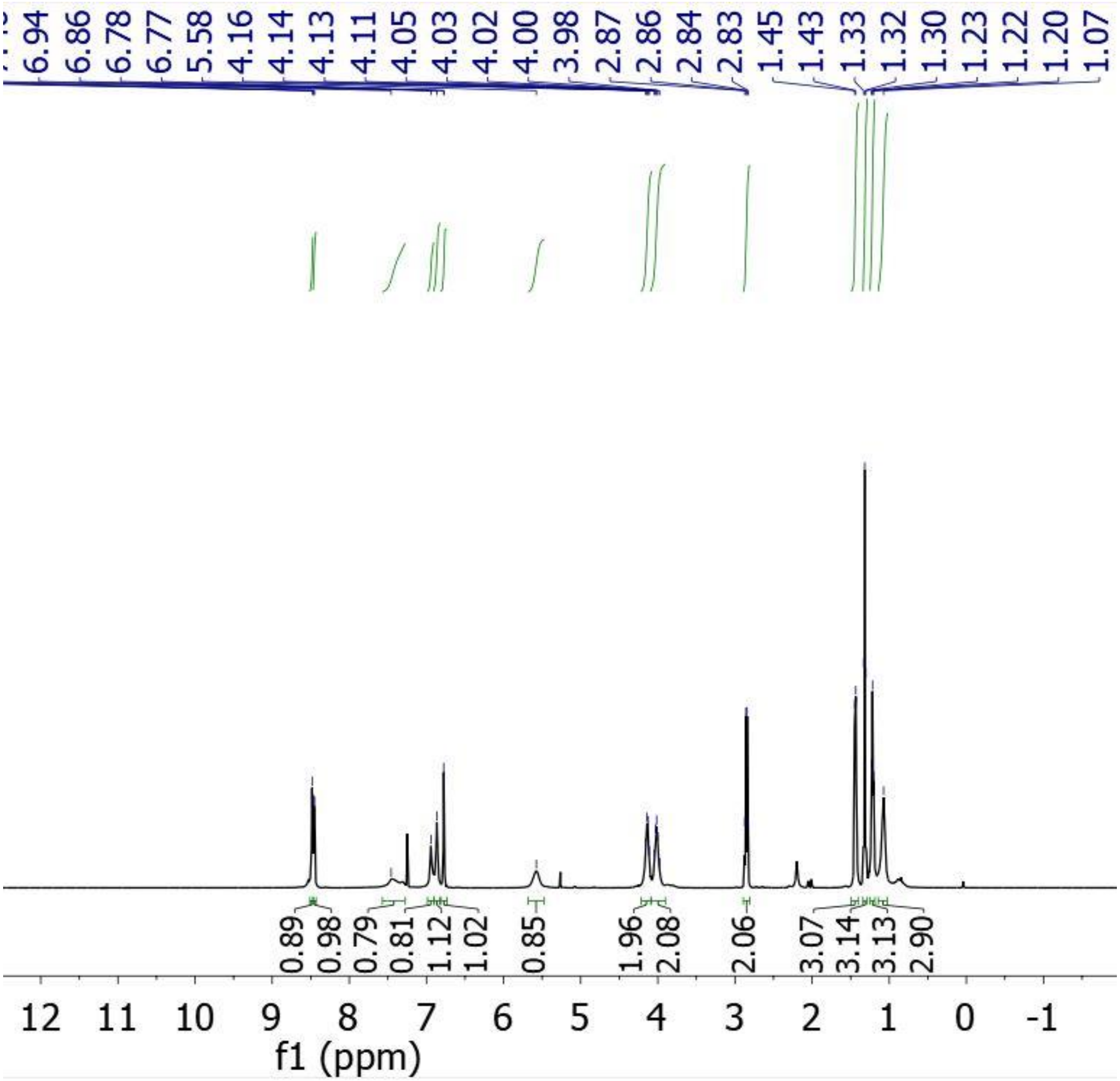

Figure S80: ^{1}H NMR of **23**

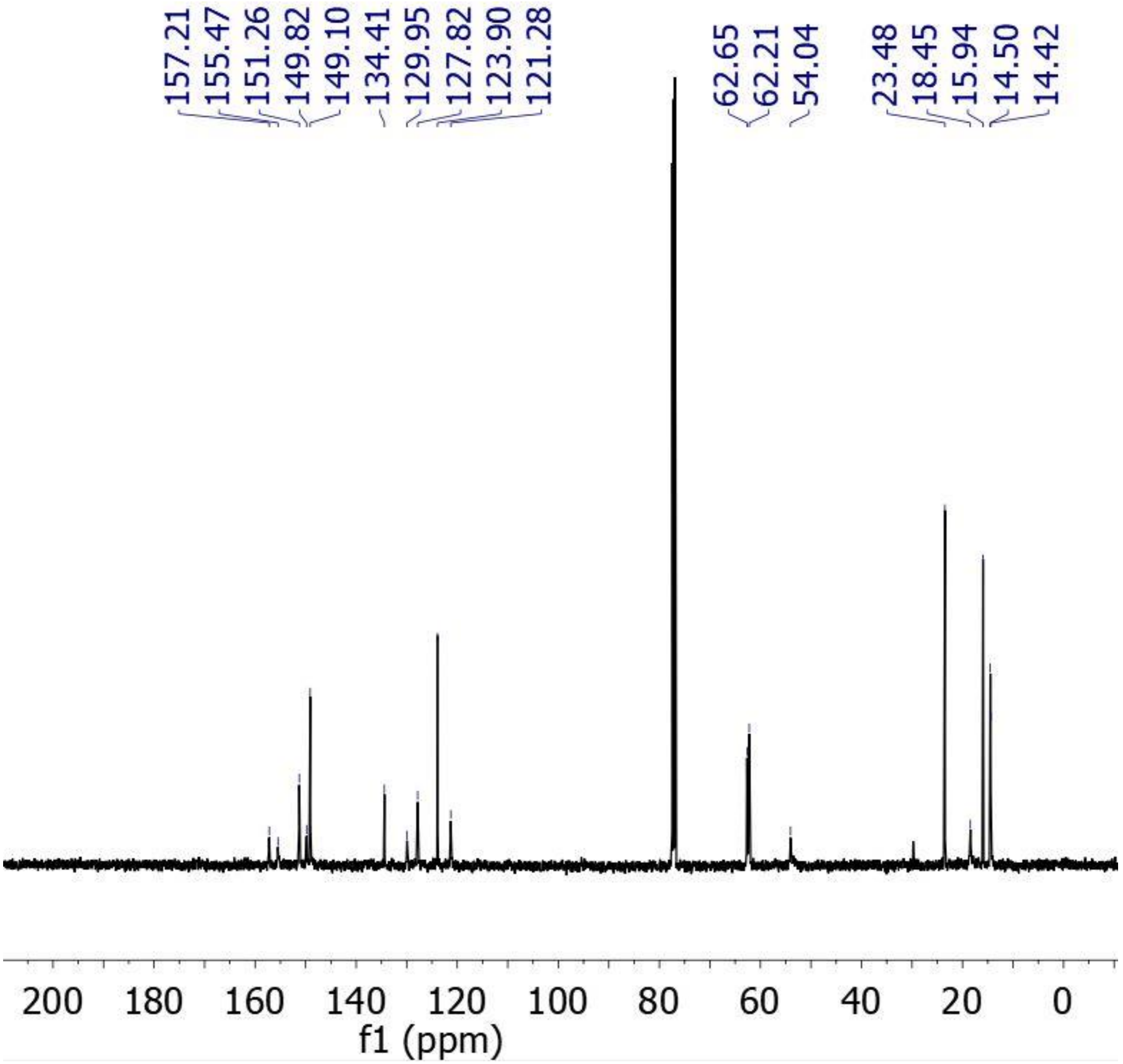

Figure S81: ^{13}C NMR of **23**

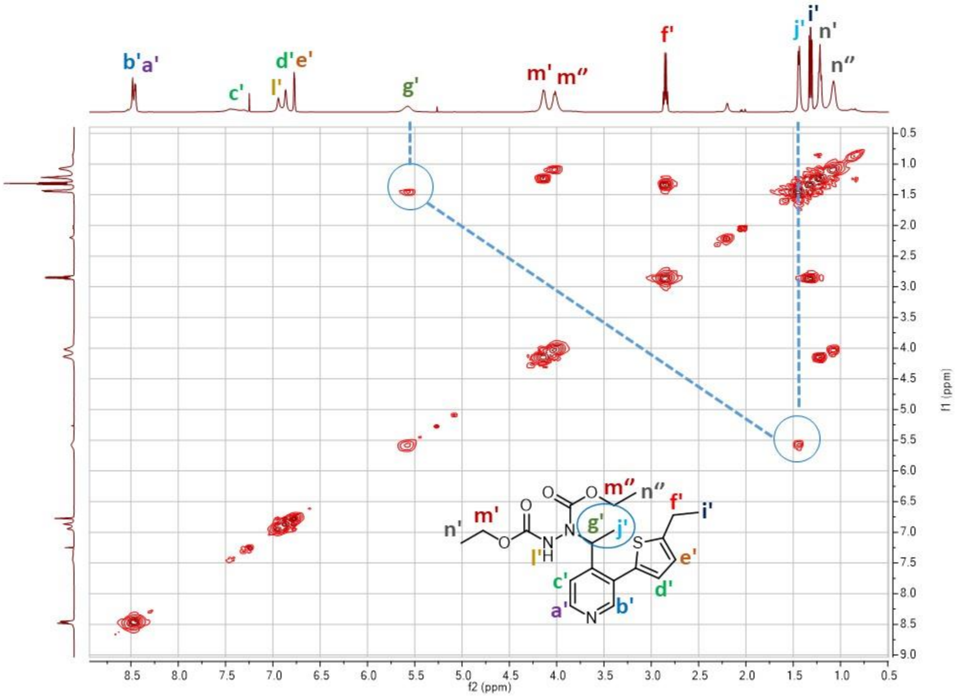

Figure S82: Correlation of g' and j' by ^{1}H-^{1}H COSY of **23**

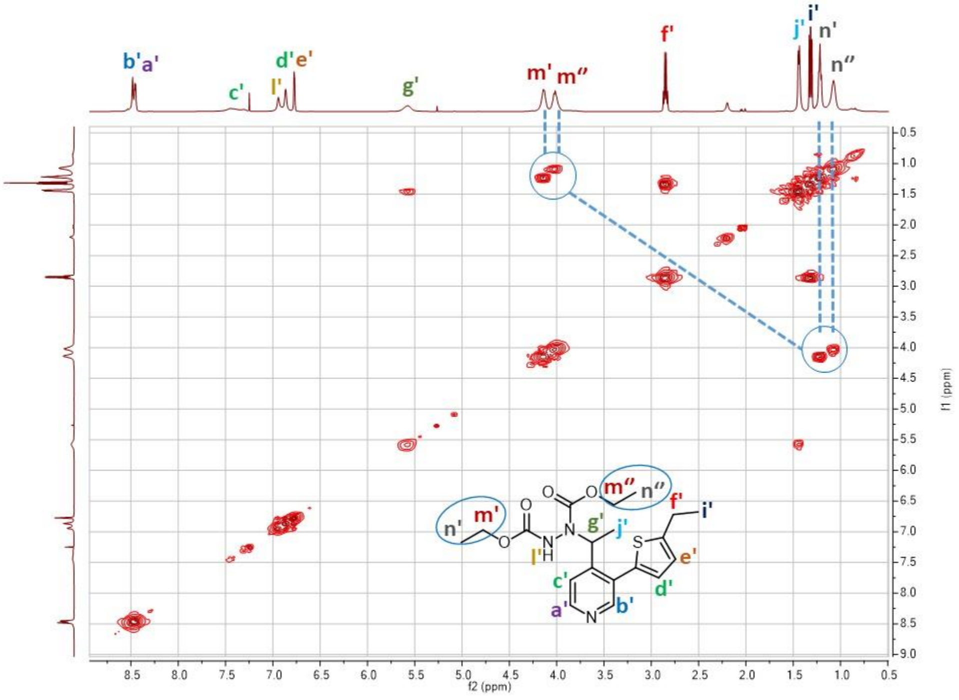

Figure S83: Correlation of m'/m'' and n'/n'' by ^{1}H-^{1}H COSY of **23**

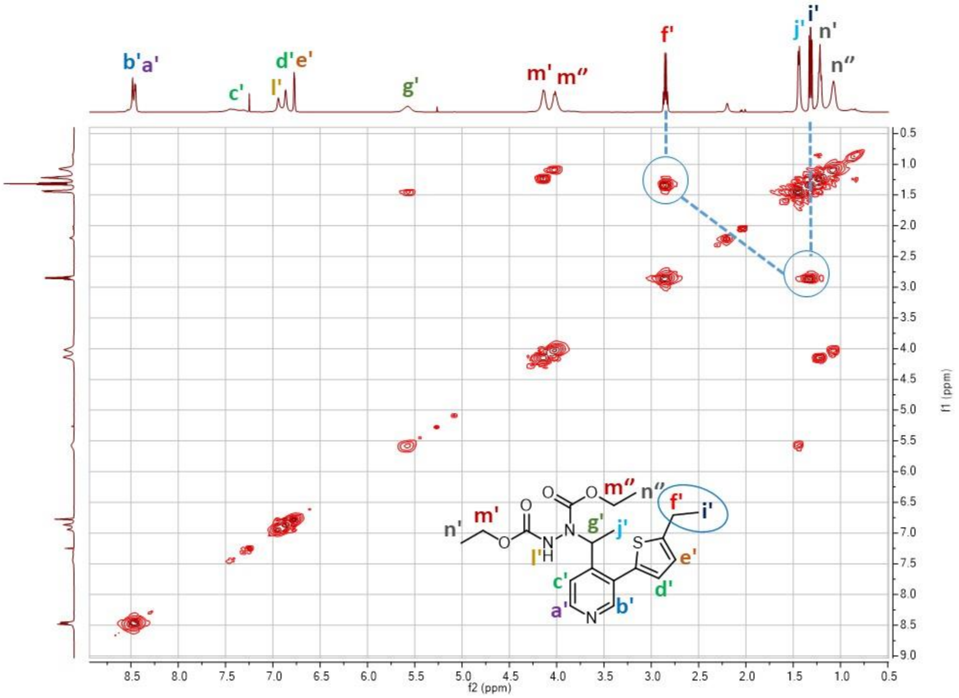

Figure S84: Correlation of f’ and i’ by ^{1}H-^{1}H COSY of **23**

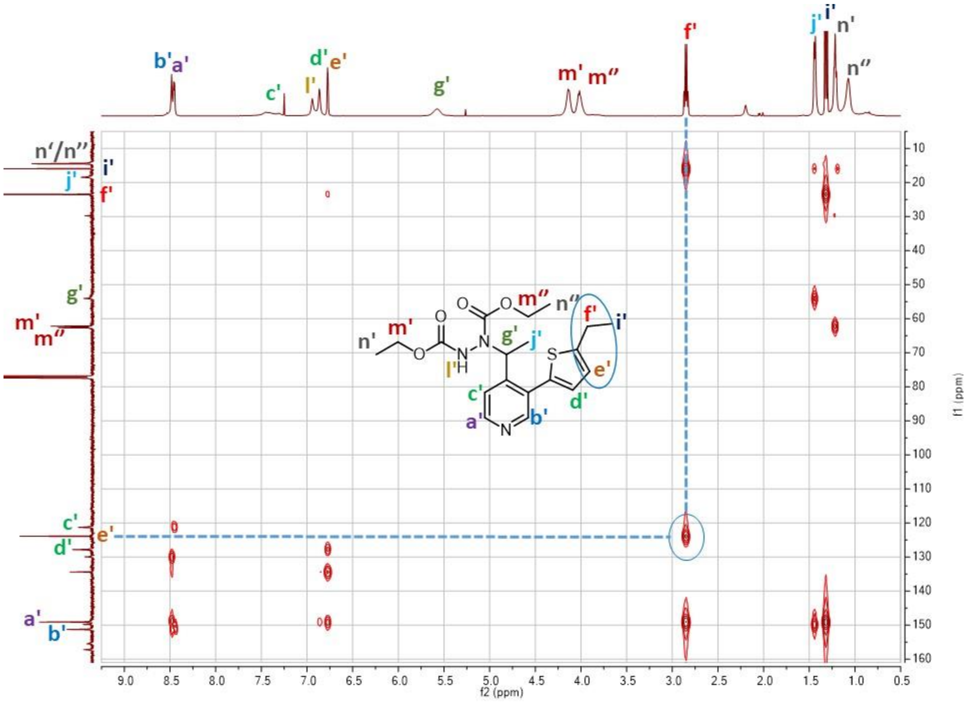

Figure S85: Correlation of f’ and e’ by HMBC of **23**

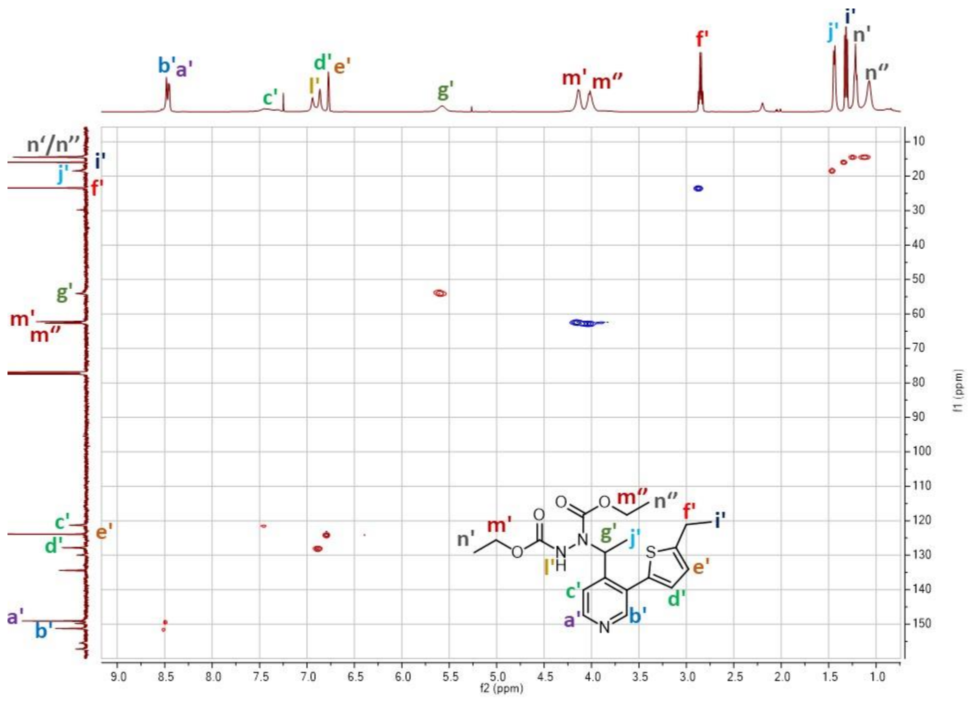

Figure S86: HSQC of **23**

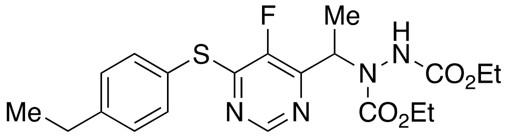

Compound 24: Amination was accomplished using procedure **A**. Column chromatography over silica gel (3:1 hexane:EtOAc) afforded 124.4 mg (0.29 mmol, 57%) of a yellow oil.

^{1}H NMR ($CDCl_3$) δ = 8.47 (s, 1H), 7.45 (d, *J* = 7.9 Hz, 2H), 7.29 (d, *J* = 7.9 Hz, 2H), 7.15 – 7.08 (bs, 1H), 5.78 – 5.54 (m, 1H), 4.28 – 4.10 (m, 4H), 2.70 (q, *J* = 7.6 Hz, 2H), 1.55 (bs, 3H), 1.32 – 1.12 (m, 9H). ^{13}C NMR ($CDCl_3$) δ = 158.64 (d, *J* = 16.1 Hz), 156.21 (d, *J* = 38.6 Hz), 153.94, 153.39, 151.76, 149.81, 146.55, 135.83, 129.22, 122.30, 62.85, 61.92, 52.81, 52.09, 28.73, 16.00, 15.17, 14.55, 14.41. HRMS m/z calcd. for $C_{20}H_{25}FN_4O_4S$ $[M+H]^+$ 437.1656, found 437.1650.

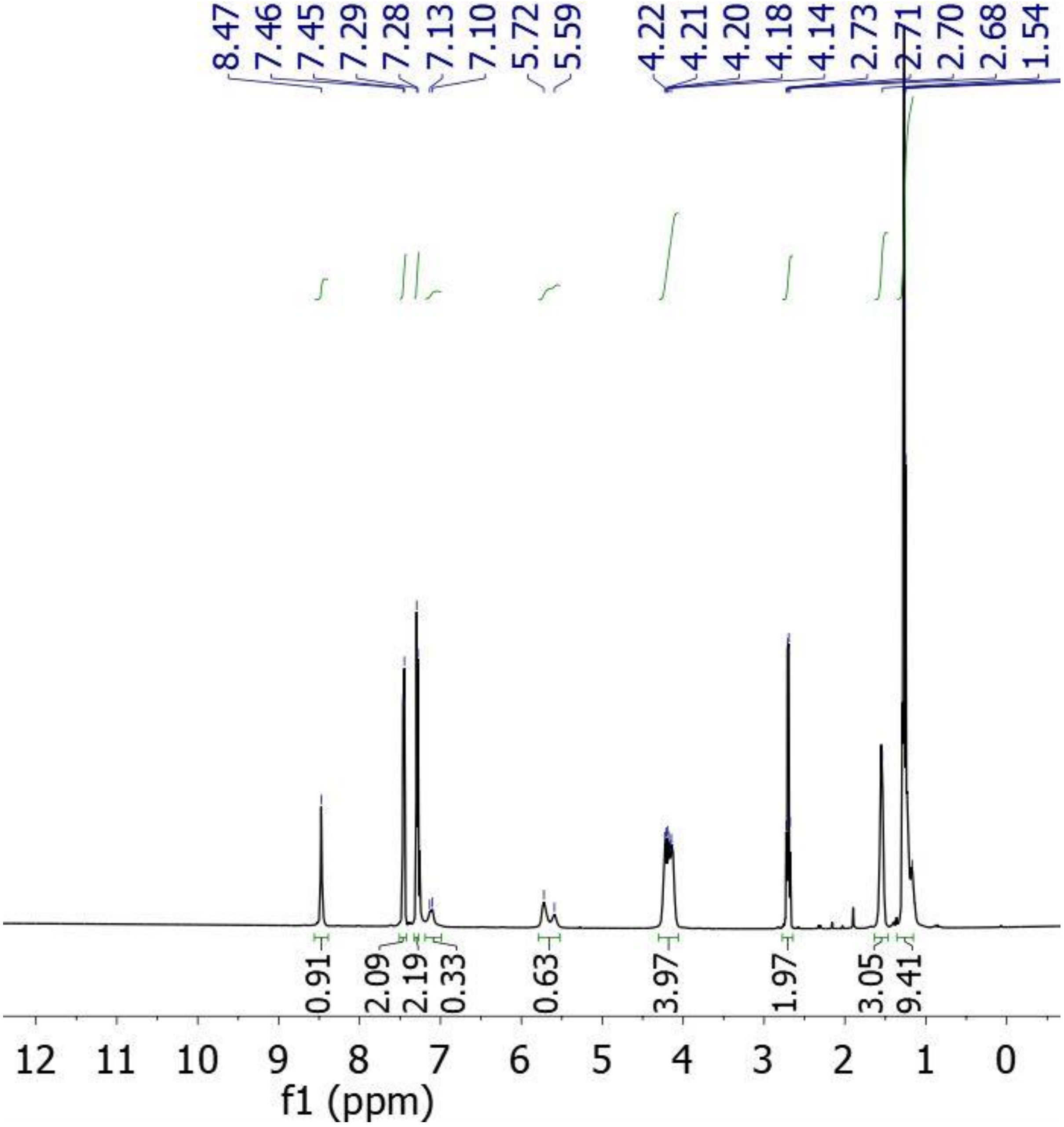

Figure S87: ^{1}H NMR of **24**

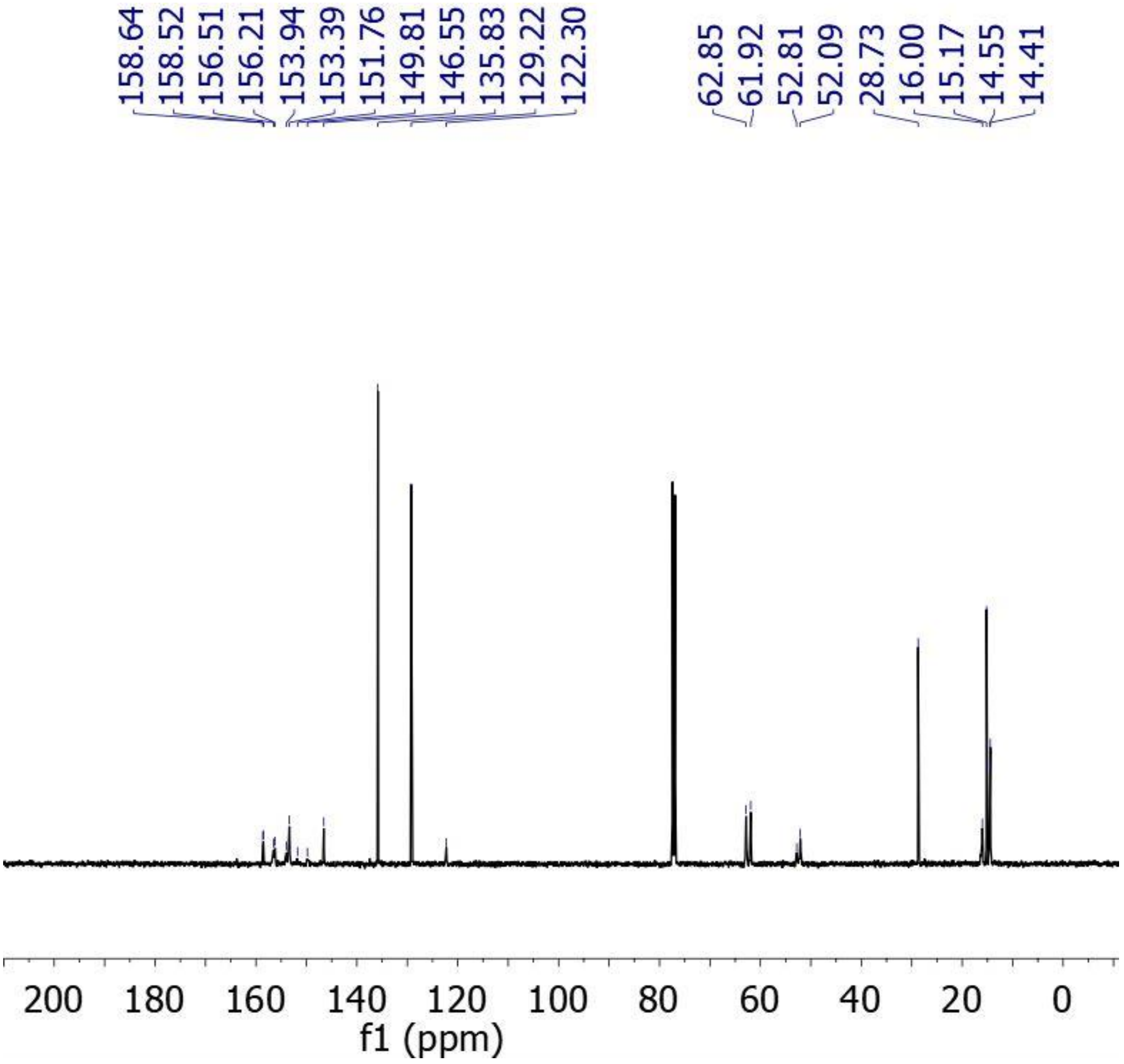

Figure 88: ^{13}C NMR of **24**

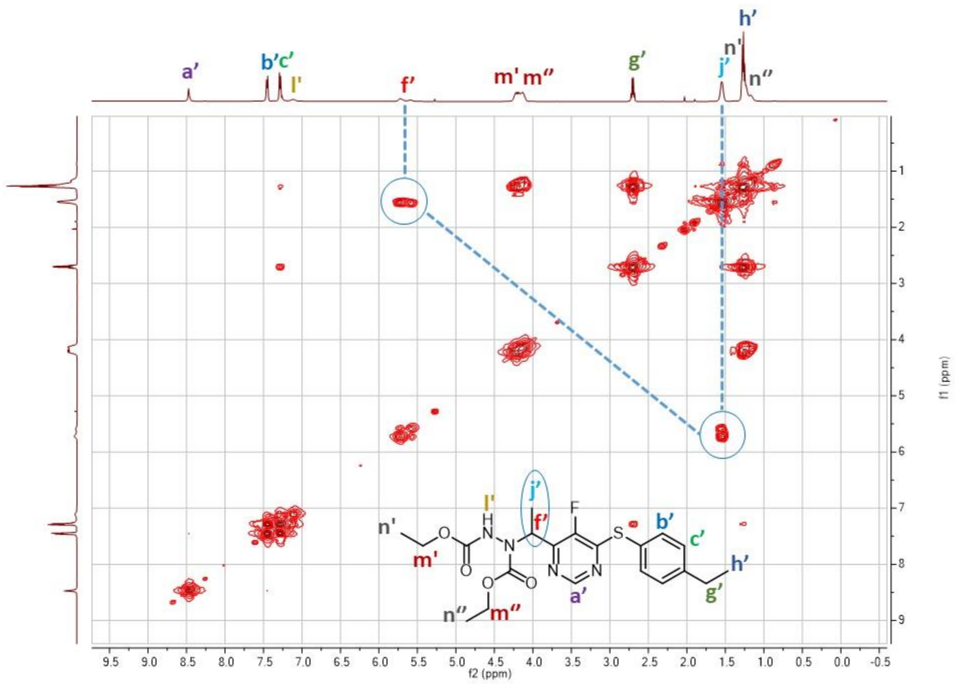

Figure S89: Correlation of f’ and j’ by ^{1}H-^{1}H COSY of **24**

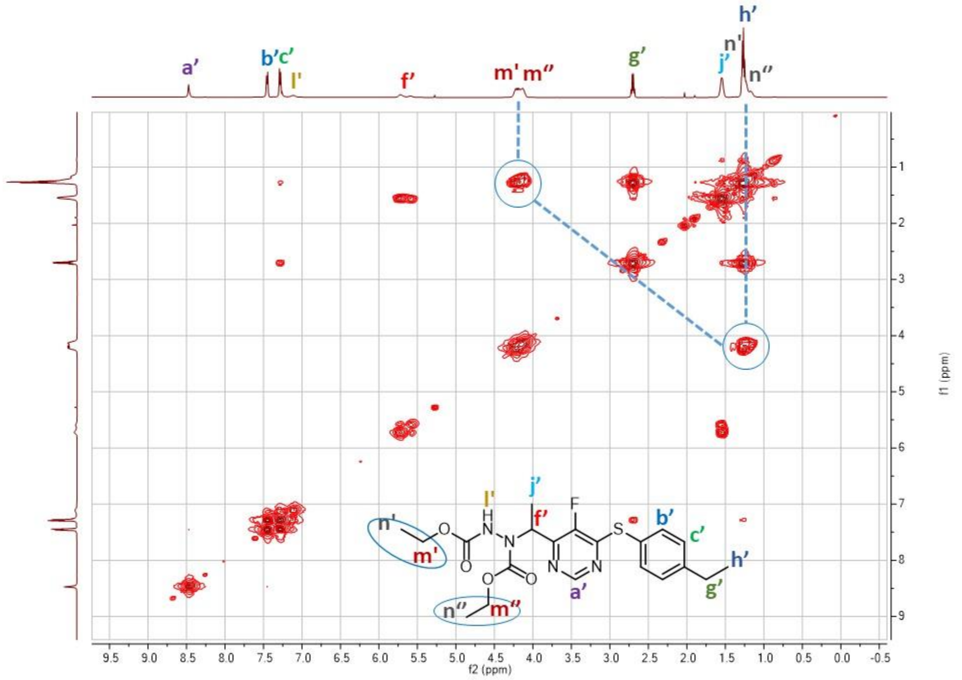

Figure S90: Correlation of n’ and m’/m” by ^{1}H-^{1}H COSY of **24**

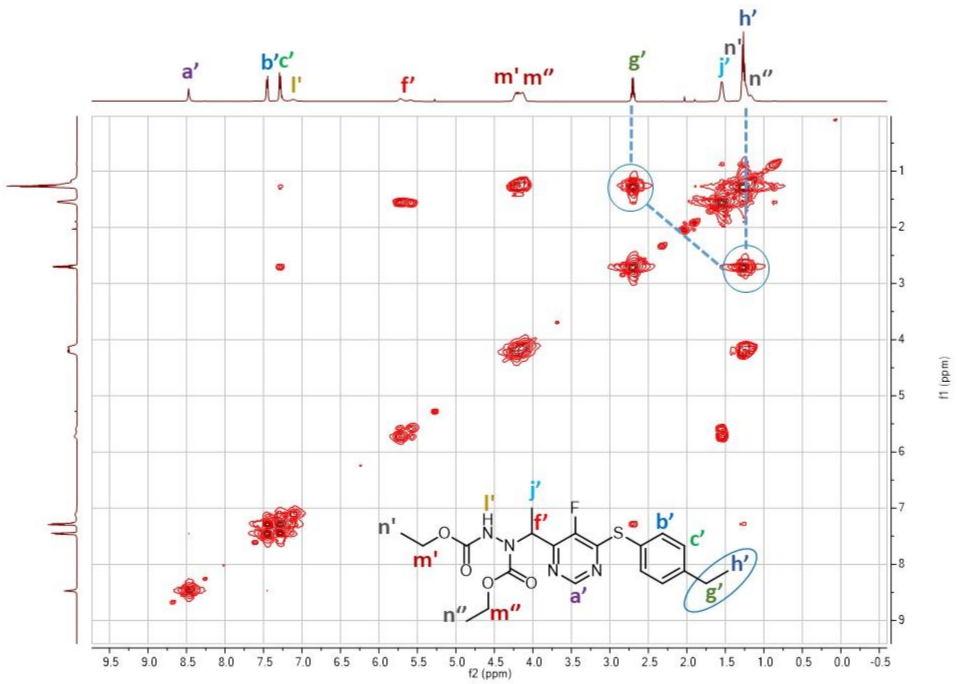

Figure S91: Correlation of h' and g' by ^{1}H-^{1}H COSY of **24**

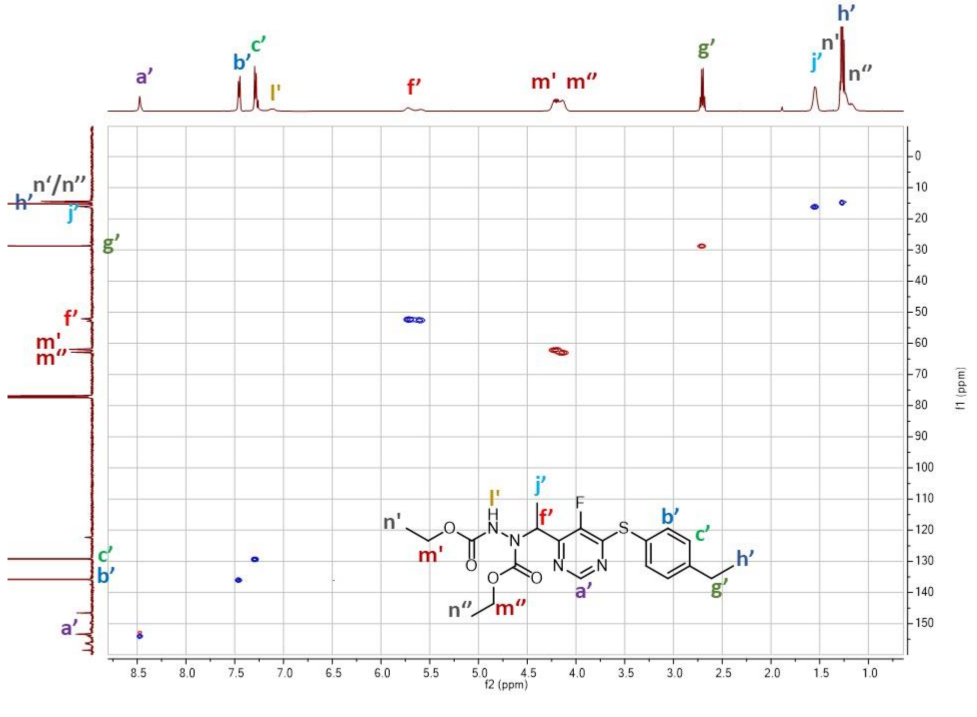

Figure S92: HSQC of **24**

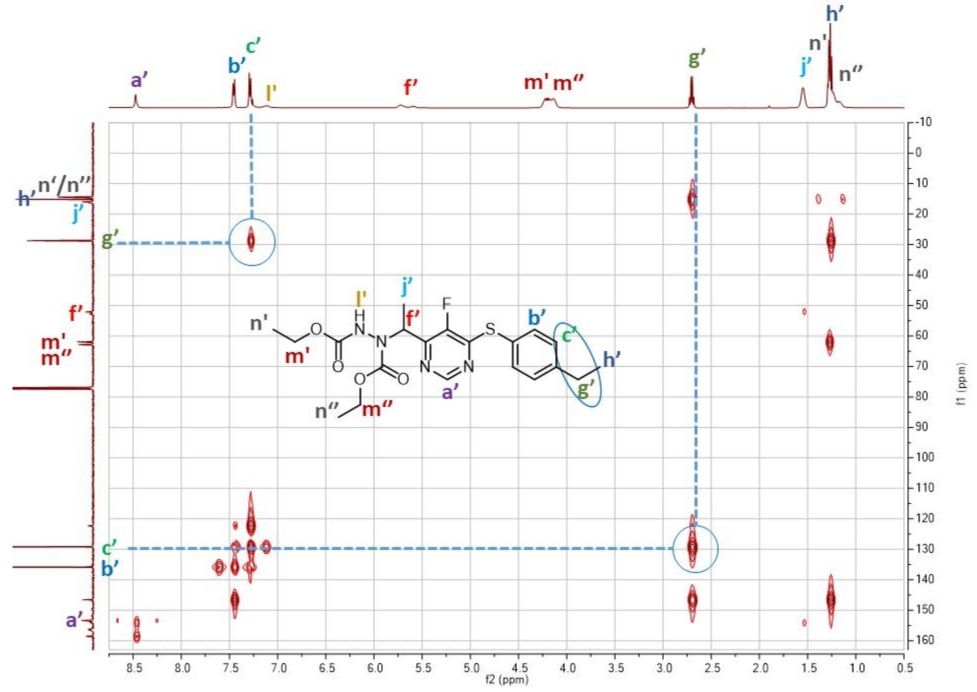

Figure S93: Correlation of c’ and g’ by HMBC of **24**

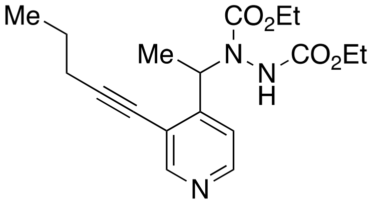

Compound 25: Amination was accomplished using procedure **A**. Column chromatography over silica gel (1:1 hexane:EtOAc) afforded 77 mg (0.22 mmol, 44%) of a yellow oil.

^{1}H NMR ($CDCl_3$) δ = 8.53 (s, 1H), 8.38 (d, *J* = 5.3 Hz, 1H), 7.40 – 7.11 (m, 1H), 6.90 (s, 1H), 5.57 (s, 1H), 4.30 – 3.97 (m, 4H), 2.42 (t, *J* = 7.1 Hz, 2H), 1.63 (h, *J* = 7.4 Hz, 2H), 1.51 (d, *J* = 7.2 Hz, 3H), 1.24 (t, *J* = 7.1 Hz, 3H), 1.17 (s, 3H), 1.03 (t, *J* = 7.3 Hz, 3H). ^{13}C NMR ($CDCl_3$) δ = 157.08, 153.18, 148.15, 120.91, 119.76, 98.68, 75.60, 62.63, 62.19, 55.82, 22.10, 21.79, 21.74, 21.68, 14.50, 13.70. HRMS m/z calcd. for $C_{18}H_{25}N_3O_4$ $[M+H]^+$ 348.1918, found 348.1914

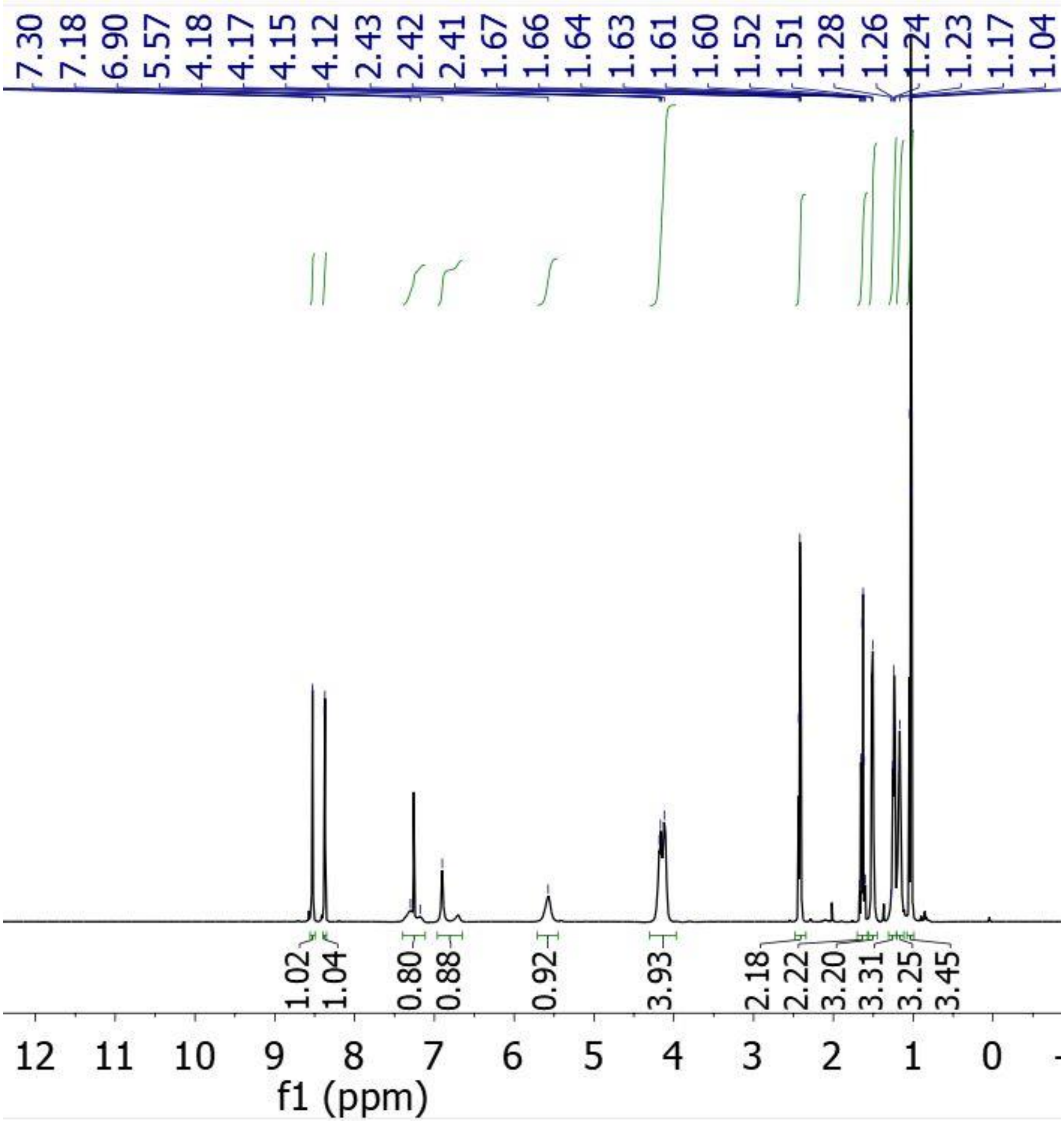

Figure S94: ^{1}H NMR of **25**

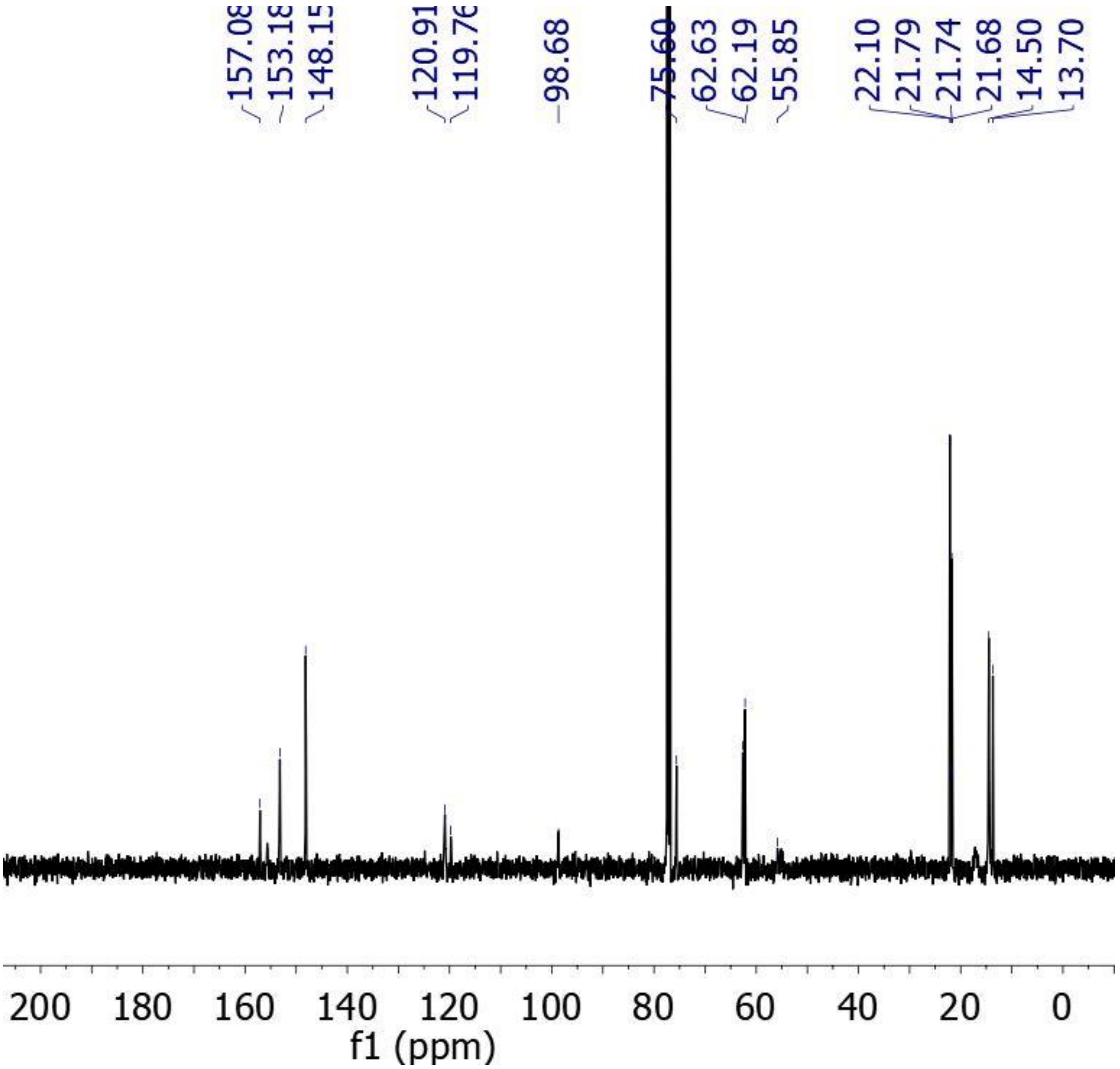

Figure S95: ^{1}H NMR of **25**

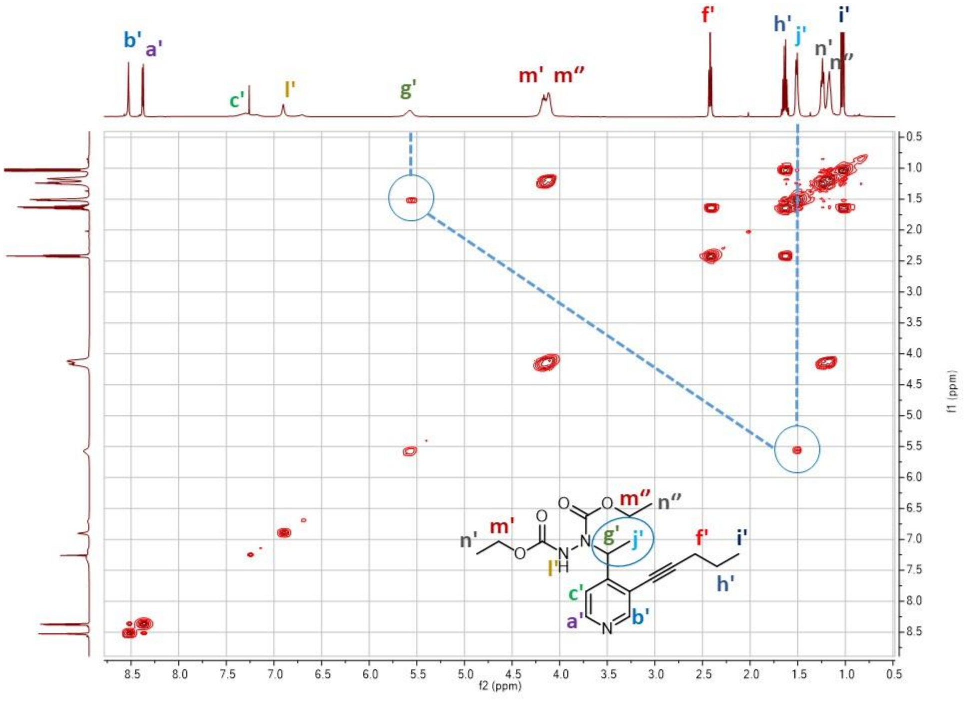

Figure S96: Correlation of j’ and g’ by ^{1}H-^{1}H COSY of **25**

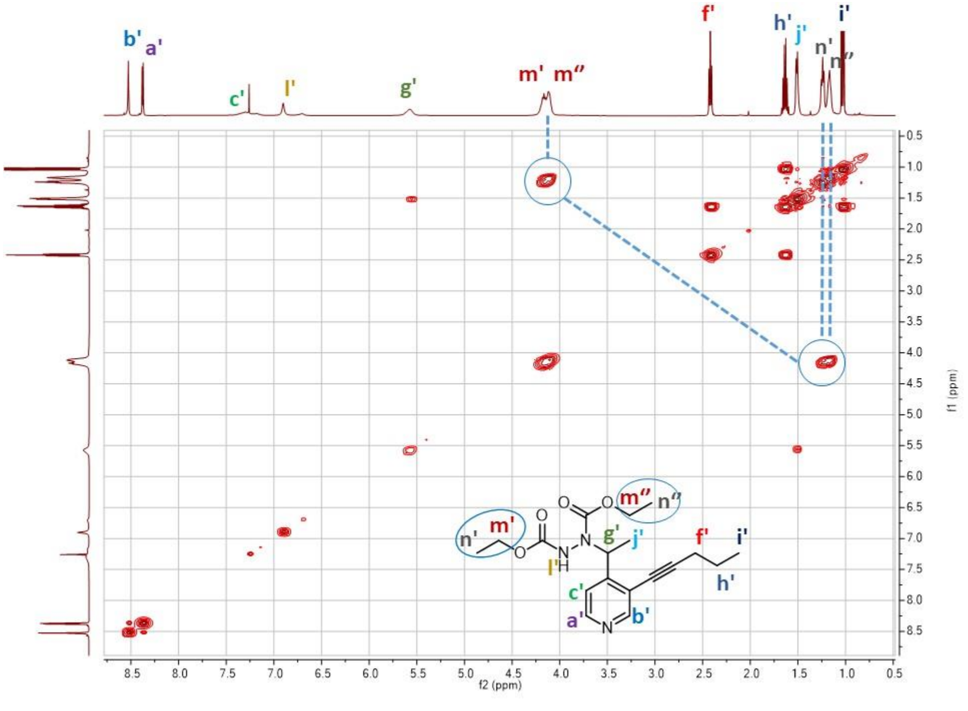

Figure S97: Correlation of m’/m” and n’/n” by ^{1}H-^{1}H COSY of **25**

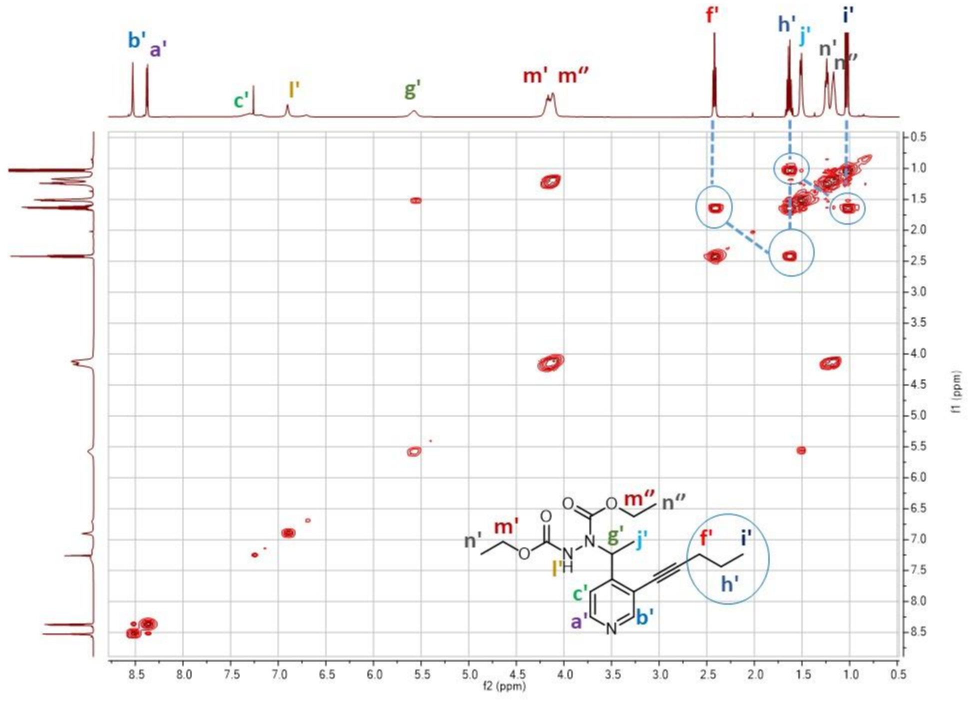

Figure S98: Correlation of f’, h’ and i’ by ^{1}H-^{1}H COSY of **25**

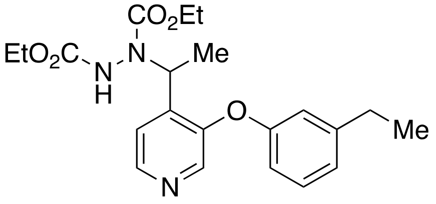

Compound 26: Amination was accomplished using procedure **A**. Column chromatography over silica gel (1:1 hexane:EtOAc) afforded 92.6 mg (0.23 mmol, 46%) of a colorless oil.

^{1}H NMR ($CDCl_3$) δ = 8.33 (d, *J* = 4.9 Hz, 1H), 8.18 (s, 1H), 7.39 (s, 1H), 7.23 (t, *J* = 7.8 Hz, 1H), 6.96 (d, *J* = 7.6 Hz, 1H), 6.81 (s, 1H), 6.74 (d, *J* = 8.2 Hz, 1H), 6.54 (s, 1H), 5.61 (s, 1H), 4.27 – 4.10 (m, 2H), 4.03 (s, 2H), 2.61 (q, *J* = 7.6 Hz, 2H), 1.55 (d, *J* = 7.3 Hz, 3H), 1.23 (dt, *J* = 15.4, 7.4 Hz, 7H), 1.10 (t,
^{13}C NMR ($CDCl_3$) δ = 157.03, 155.45, 150.87, 146.84, 145.01, 141.83, 141.14, 129.92, 129.41, 123.46, 122.70, 117.49, 115.04, 62.68, 62.23, 52.58 – 51.99 (m), 28.85, 16.89, 15.51, 14.52, 14.43. HRMS m/z calcd. for $C_{21}H_{27}N_3O_5$ $[M+H]^+$ 402.2023, found 402.2018

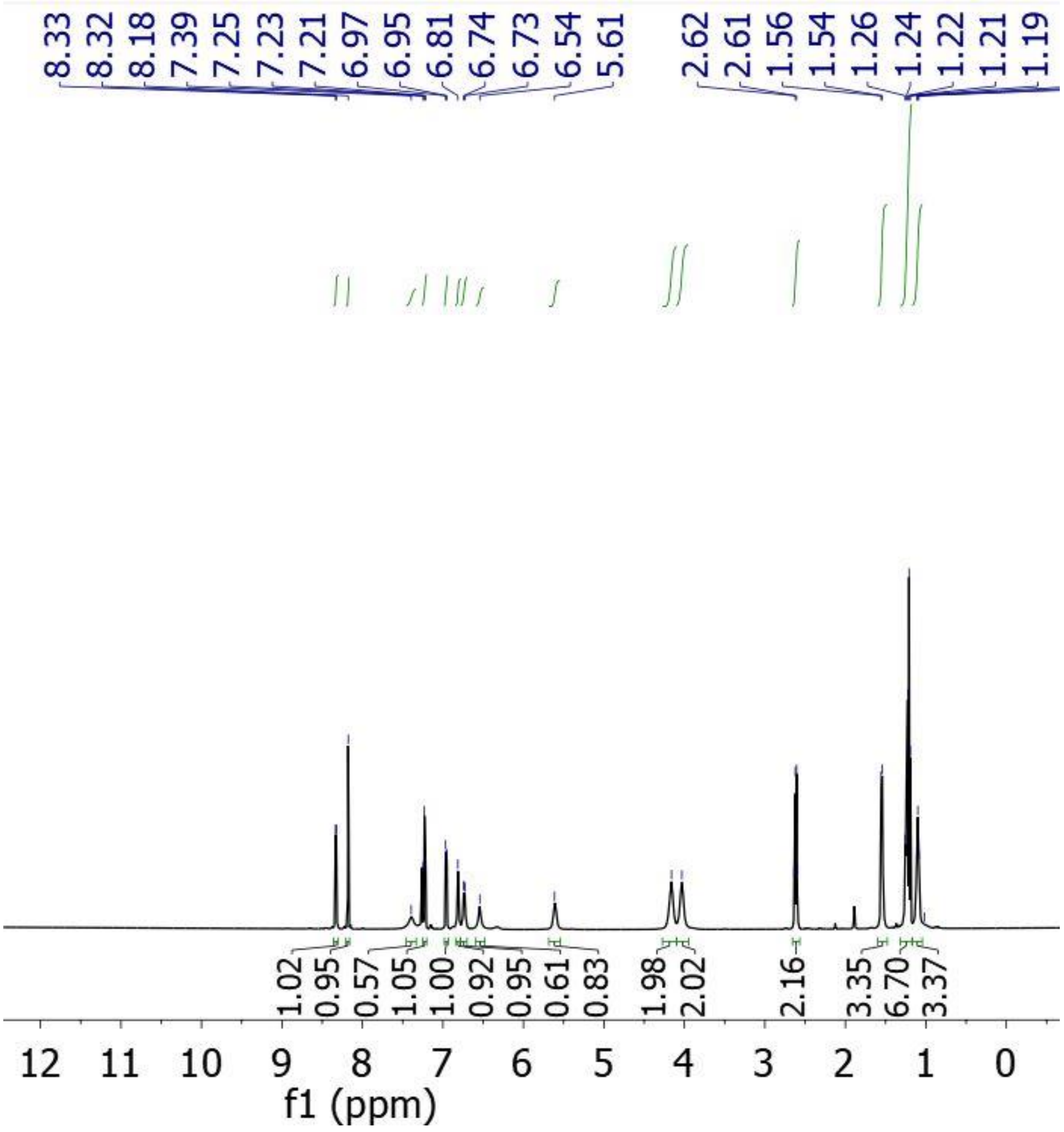

Figure S99: ^{1}H NMR of **26**

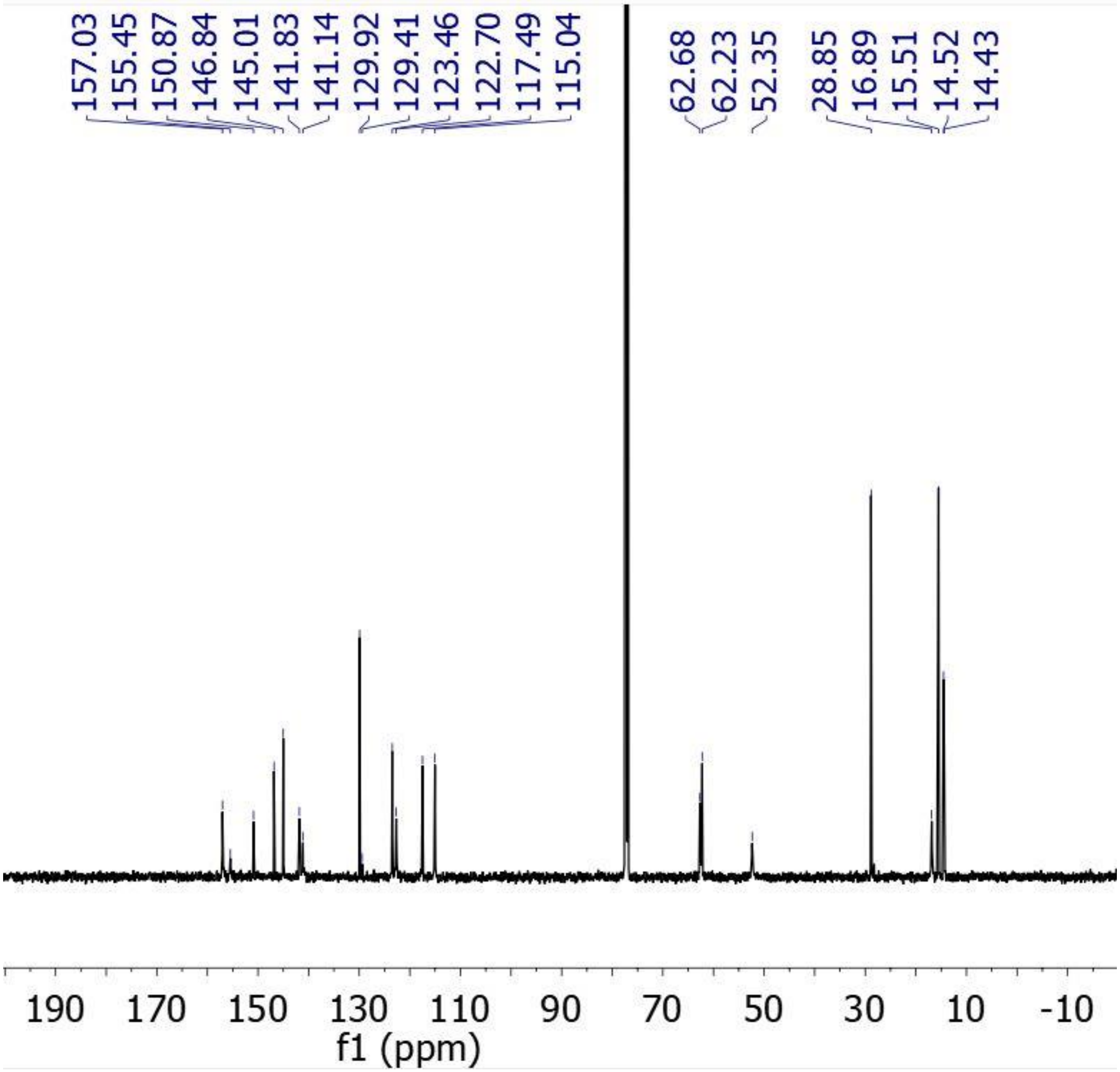

Figure S100: ^{13}C NMR of **26**

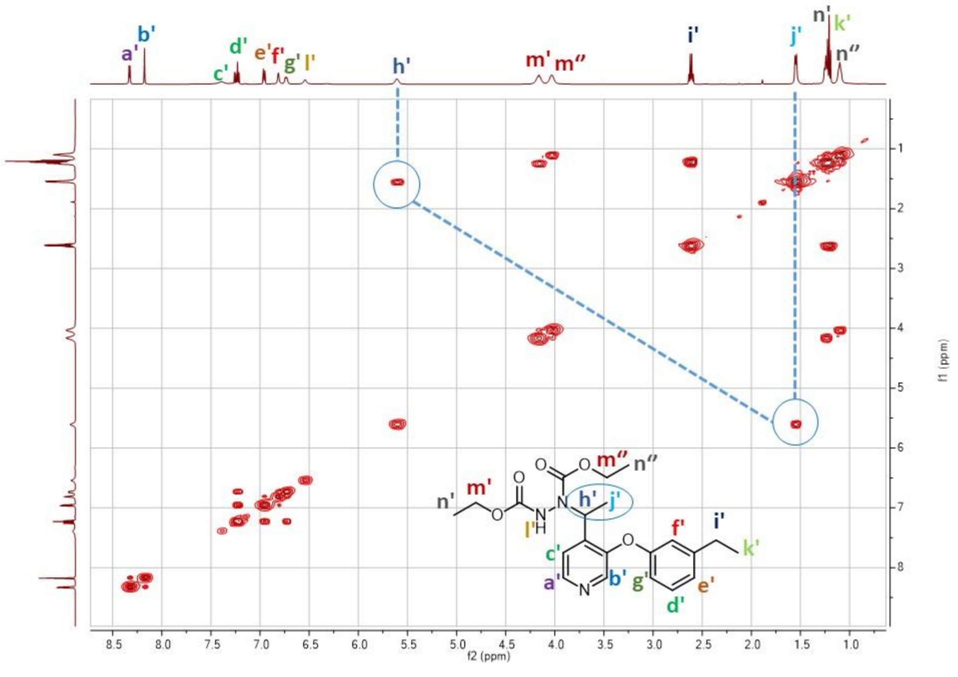

Figure S101: Correlation of h' and j' by ^{1}H-^{1}H COSY of **26**

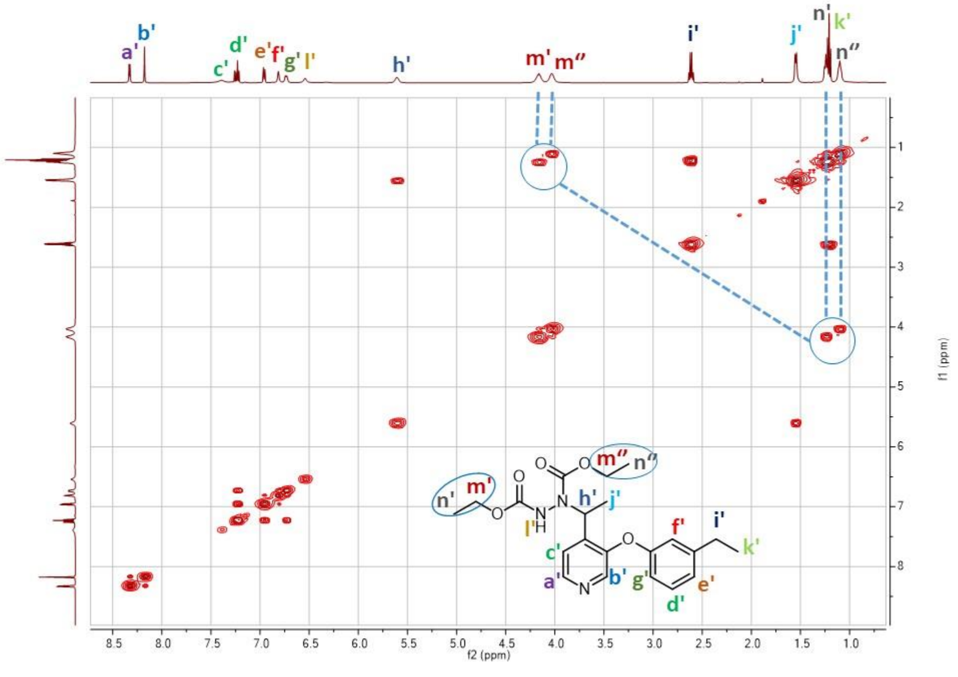

Figure S102: Correlation of m'/m" and n'/n" by ^{1}H-^{1}H COSY of **26**

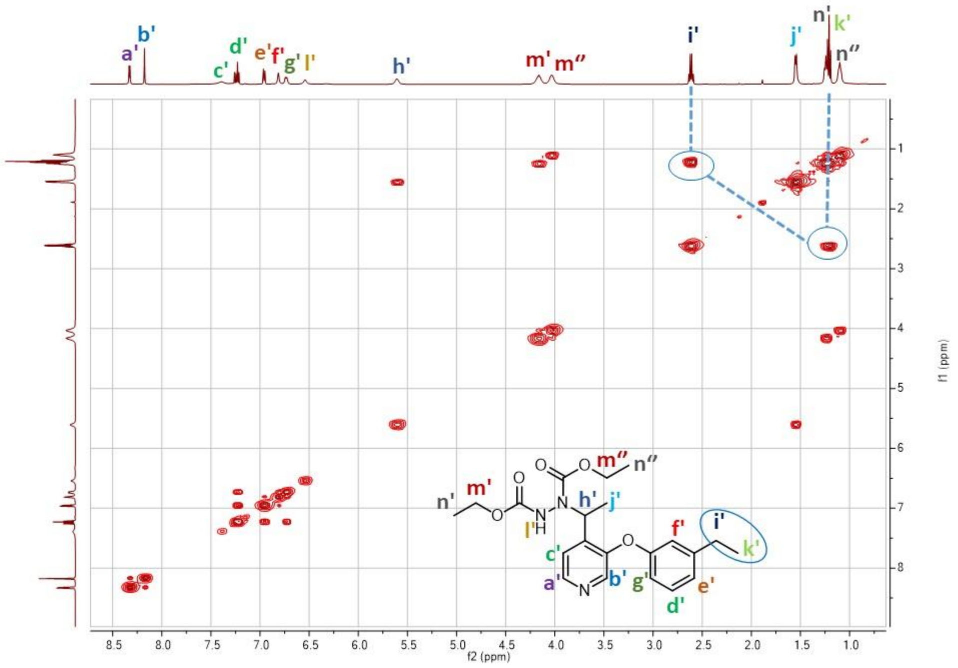

Figure S103: Correlation of i’ and k’ by ^1H-^1H COSY of **26**

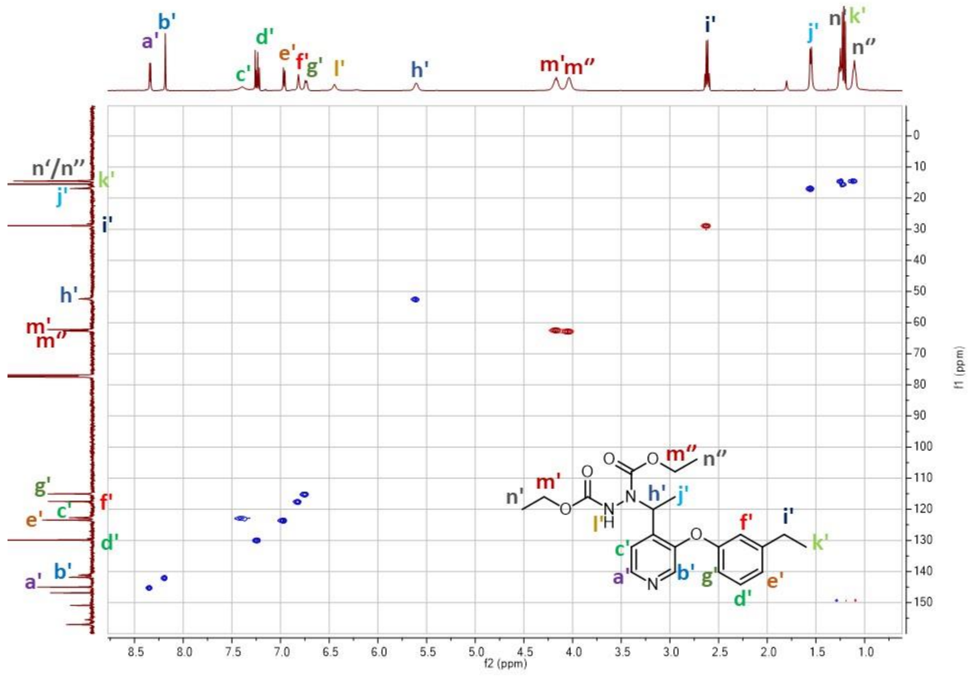

Figure S104: HSQC of **26**

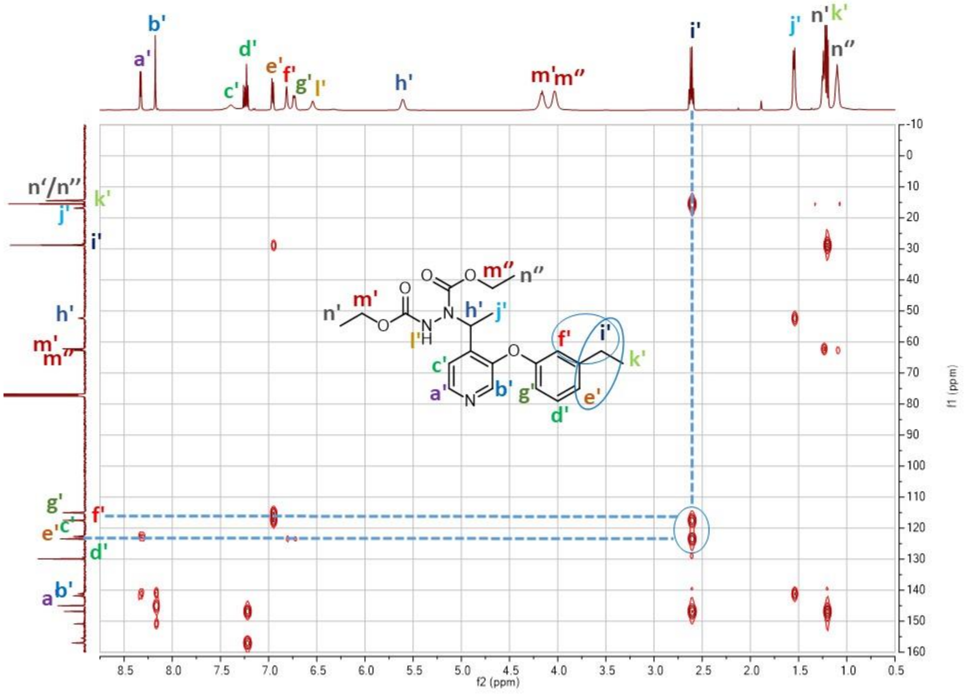

Figure S105: Correlation of i’ and e’/f’ by HMBC of **26**

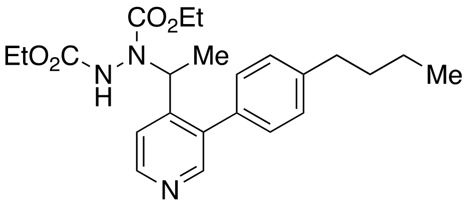

Compound 27: Amination was accomplished using procedure **A**. Column chromatography over silica gel (1:1 hexane:EtOAc) afforded 107.5 mg (0.26 mmol, 52%) of a white solid.

^{1}H NMR ($CDCl_3$) δ = 8.47 (d, *J* = 5.1 Hz, 1H), 8.37 (s, 1H), 7.52 – 7.30 (m, 1H), 7.28 – 7.14 (m, 4H), 7.05 (s, 1H), 5.38 (s, 1H), 4.12 (s, 2H), 4.06 – 3.79 (m, 2H), 2.64 (t, *J* = 7.7 Hz, 2H), 1.63 (p, *J* = 8.1, 7.6 Hz, 2H), 1.37 (p, *J* = 7.5 Hz, 5H), 1.20 (t, *J* = 7.0 Hz, 3H), 1.05 (s, 3H), 0.93 (t, *J* = 7.3 Hz, 3H). ^{13}C NMR ($CDCl_3$) δ = 157.12, 155.31, 150.61, 148.63, 142.68, 136.67, 134.43, 129.05, 128.56, 121.11, 62.47, 62.05, 53.95, 35.42, 33.62, 22.46, 18.16, 14.45, 14.36, 14.02. HRMS m/z calcd. for $C_{23}H_{31}N_3O_4$ $[M+H]^+$ 414.2387, found 413.2381

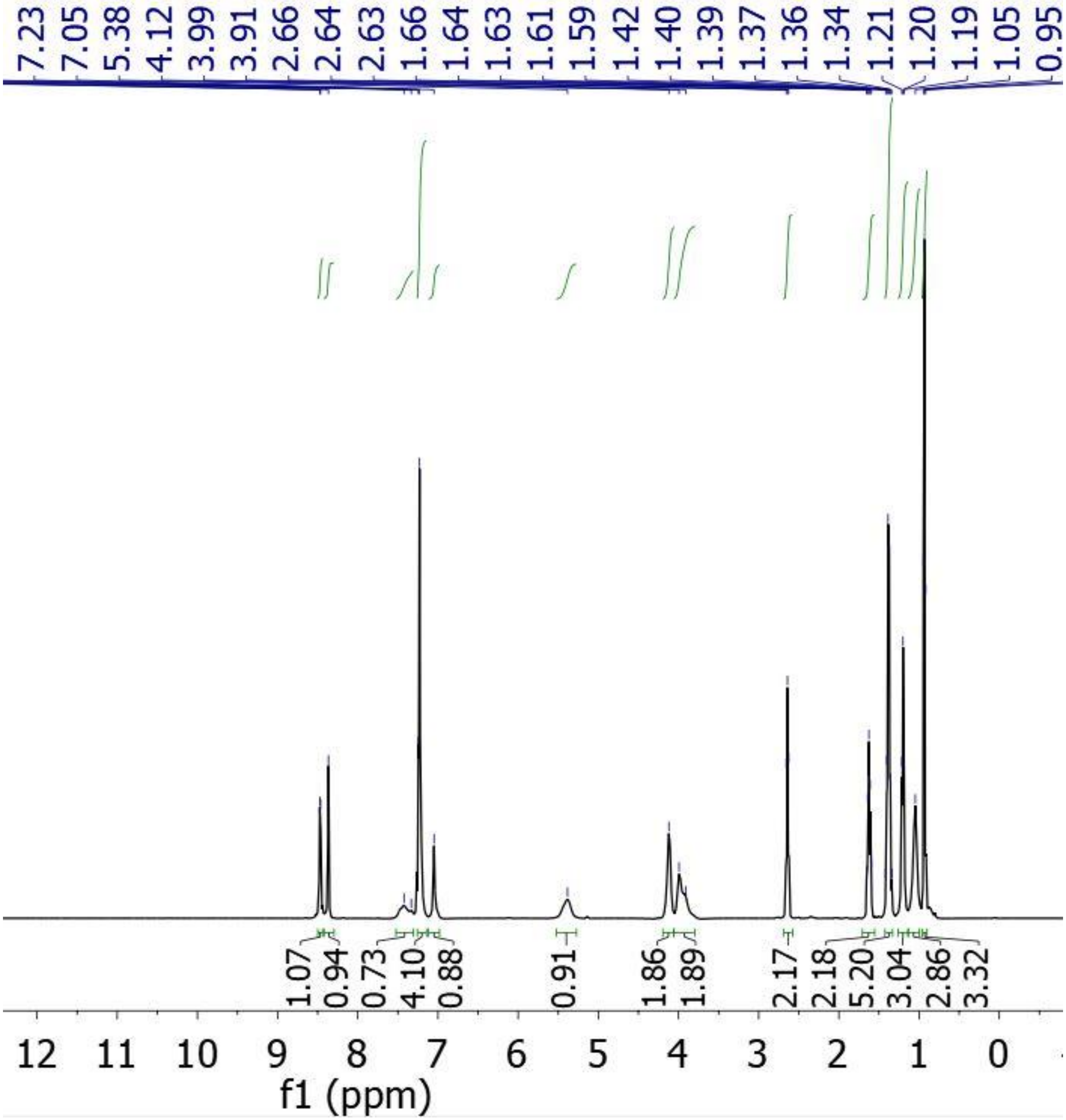

Figure S106: ^{1}H NMR of **27**

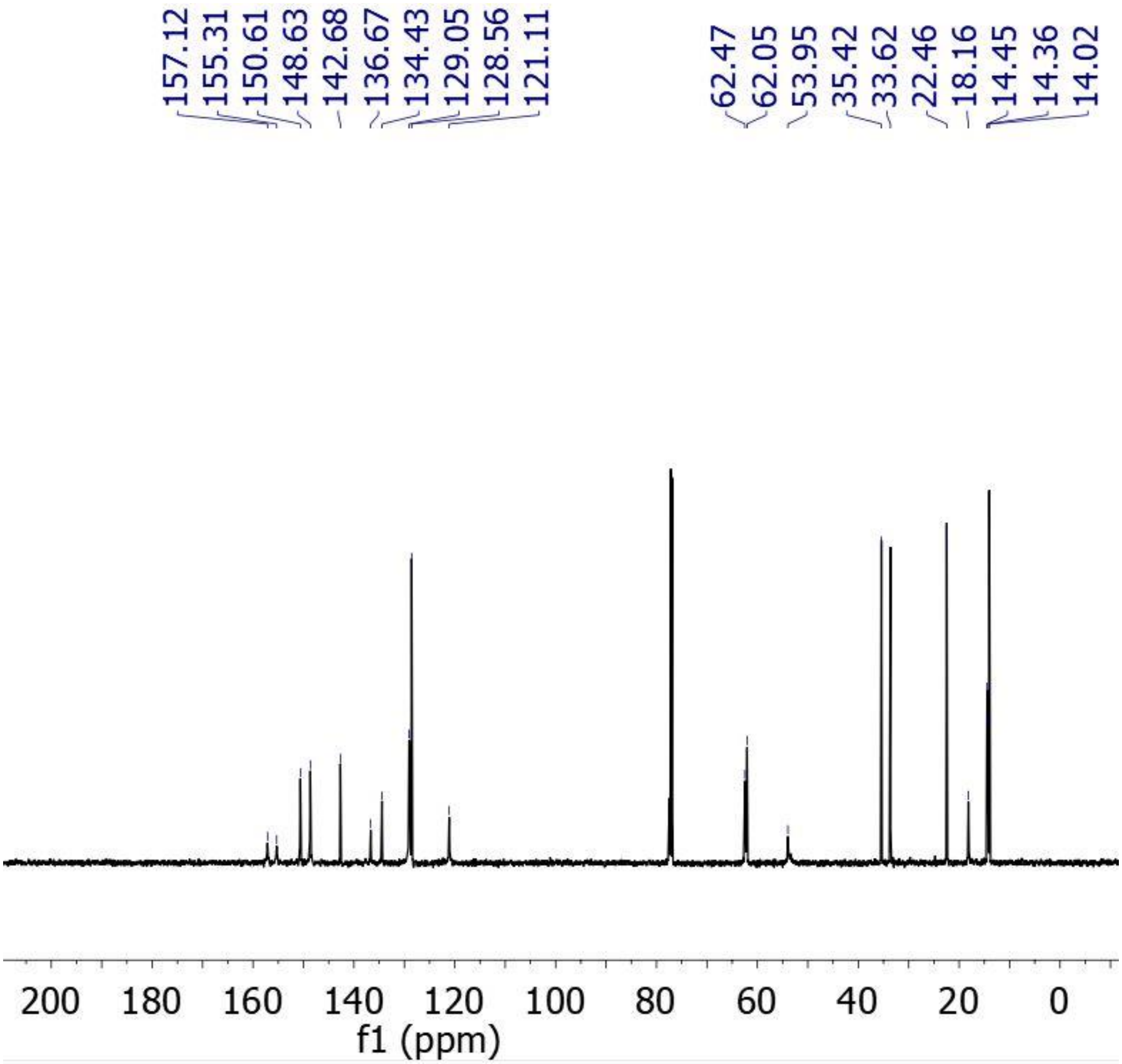

Figure S107: ^{13}C NMR of **27**

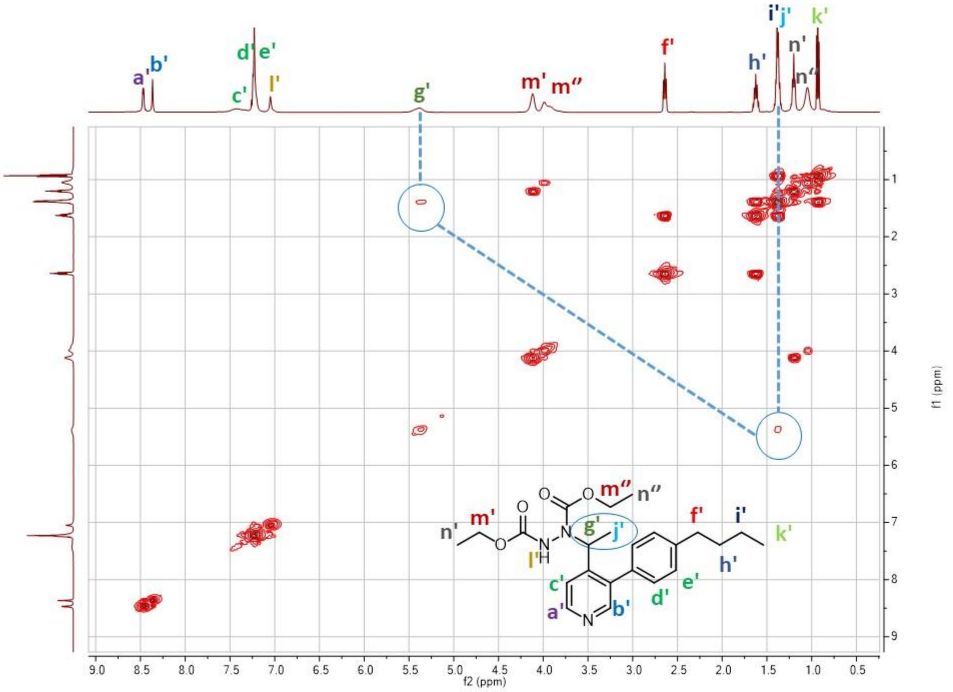

Figure S108: Correlation of g' and j' by ^{1}H-^{1}H COSY of **27**

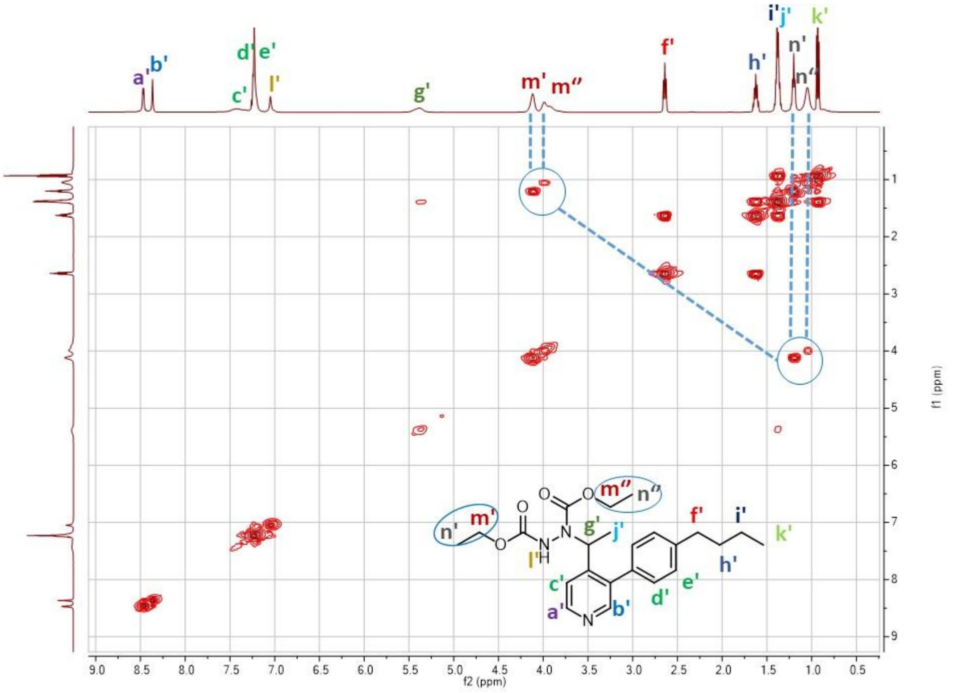

Figure S109: Correlation of m'/m'' and n'/n'' by ^{1}H-^{1}H COSY of **27**

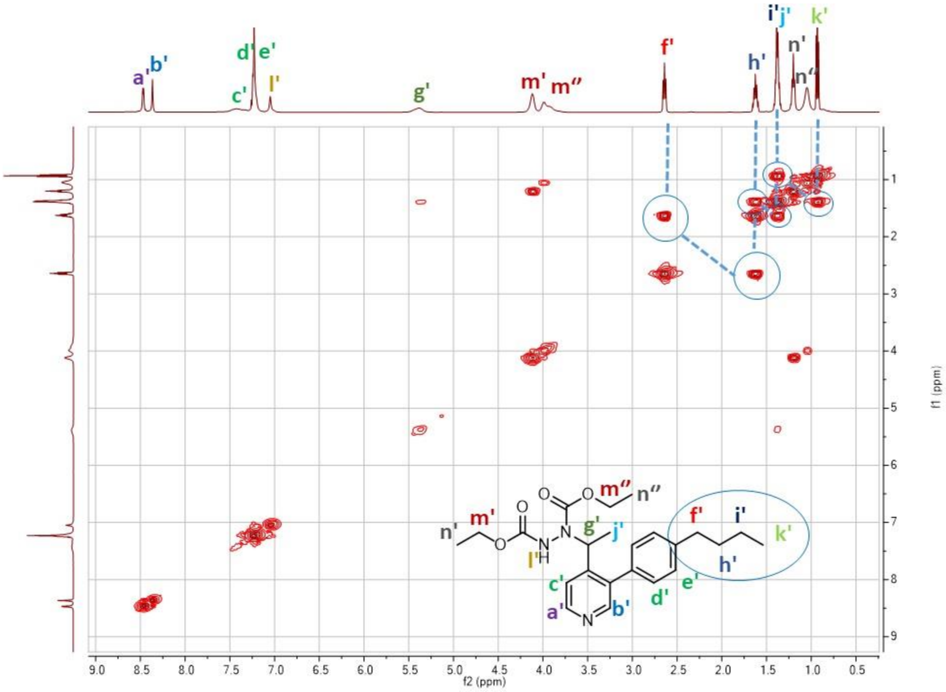

Figure S110: Correlation of f’ and h’ and correlation of i’ and k’ by ^{1}H-^{1}H COSY of **27**

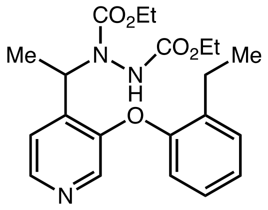

Compound 28: Amination was accomplished using procedure **A** on 0.2 mmol scale. Column chromatography over silica gel (gradient elution from 2:1 to 1:1 hexane:EtOAc) afforded 42.0 mg (0.11 mmol, 52%) of a clear oil.

^{1}H NMR (400 MHz, Chloroform-*d*) ∂ 8.29 (d, *J* = 4.9 Hz, 1H), 8.00 (s, 1H), 7.47-7.21 (m, 1H), 7.13 (p, *J* = 7.2, 6.6 Hz, 2H), 6.83 ? 6.55 (m, 2H), 5.70 (s, 1H), 4.23-4.00 (m, 6H), 2.68 (q, *J* = 7.4 Hz, 2H), 1.59 (d, *J* = 6.9 Hz, 3H), 1.23 (t, *J* = 7.5 Hz, 6H), 1.11 (s, 3H). ^{13}C NMR (101 MHz, $CDCl_3$) ∂ 156.86, 153.85, 151.42, 144.25, 140.03, 139.69, 134.96, 129.91, 127.35, 124.47, 122.34, 118.22, 62.51, 62.08, 52.22, 23.03, 16.75, 14.37, 14.28. HRMS calculated for $[C_{21}H_{28}N_3O_5]^+$ 402.2029, found 402.2029.

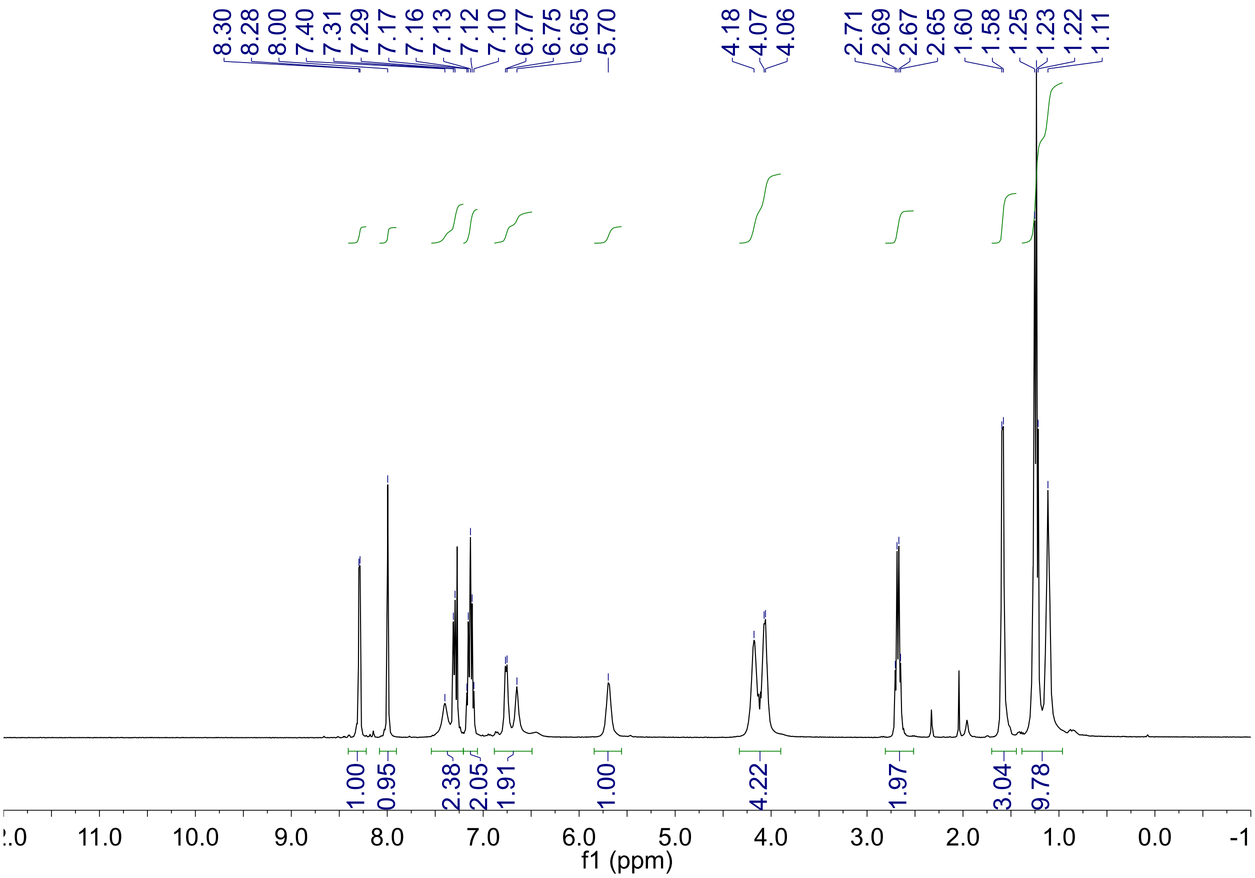

Figure S111: ^{1}H NMR spectrum of **28**

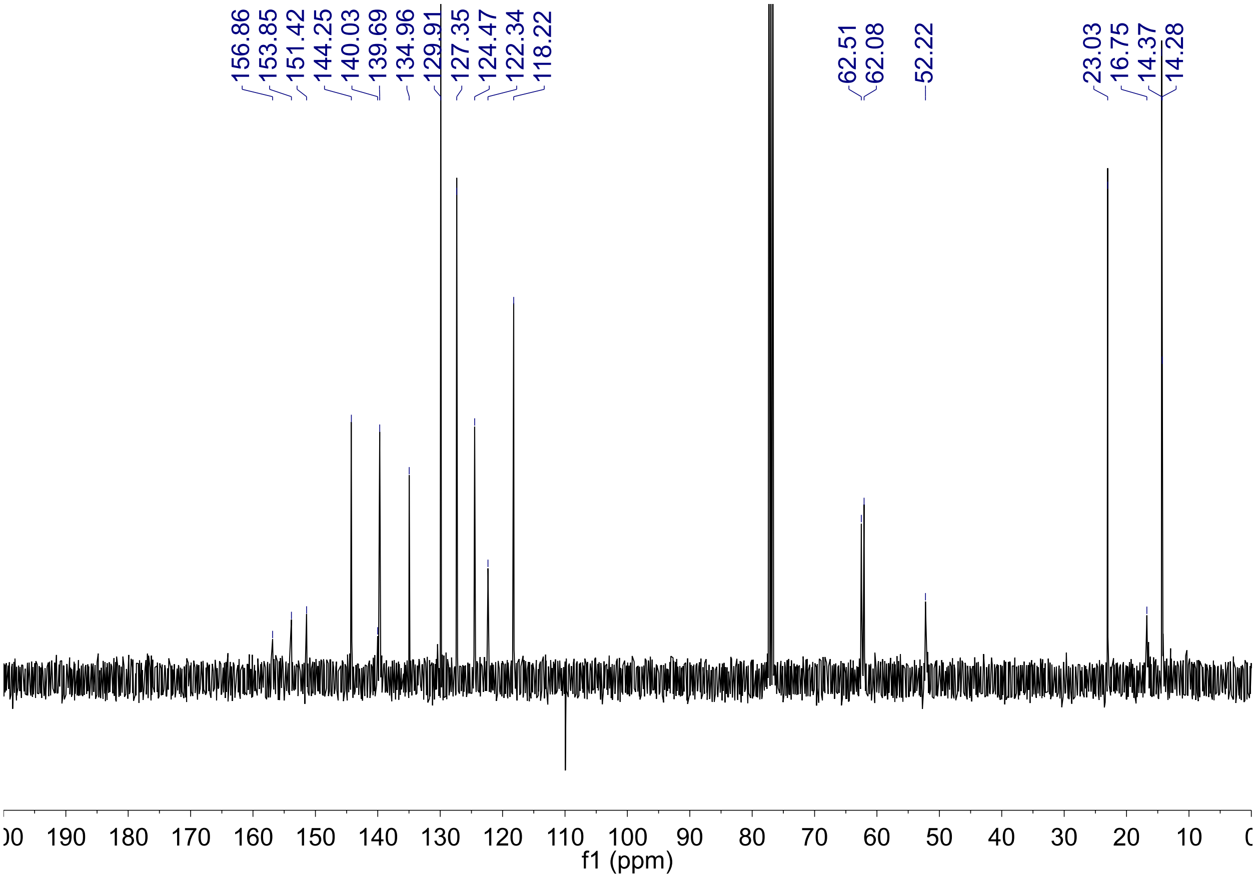

Figure S112: ^{13}C NMR spectrum of **28**

5. Isotope exchange measurements

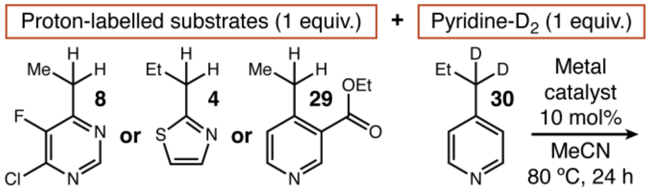

Figure S113. Benzylic isotope exchange experiments

Into six oven-dried 2 dram vials with Teflon-faced septa dried under purging nitrogen, copper(II) triflate and scandium(III) triflate were charged (3 vials each, 24.6 mg Sc(III) and 18.1 mg Cu(II), 0.05 mmol each). The vials were purged again with flowing nitrogen for five minutes, followed by the addition of 1 mL dry acetonitrile. Into each of the vials was charged a substrate with protons at the benzylic position (2-propylthiazole, 1 vial with each metal, 62 µL; 6-chloro-5-fluoro-4-ethylpyrimidine, 1 vial with each metal, 62 µL; ethyl 4-ethylnicotinate, 1 vial with each metal, 80 µL). Next, 4-(propyl-1,1-d_2)pyridine (65 µL) was added to each vial. The vials were sealed with melted parafilm, and then heated to 80 °C for 24 hours. After cooling to room temperature, each of the vials was loaded directly onto silica gel and eluted using a solvent

gradient 2:1 to 1:1 hexanes:ethyl acetate to recover the thiazole, pyrimidine, and nicotinic ester starting materials. NMR analysis using a ten second d1 delay time was used to analyze the amount of deuterium incorporation at the benzylic position, giving the results presented in Scheme 1 of the manuscript. Assuming there was no thermodynamic preference for deuterium to populate either benzylic position, 100% exchange would result in (on average) 1 deuterium incorporated per functionalized substrate (as a mixture of mono- and di-deuterated).

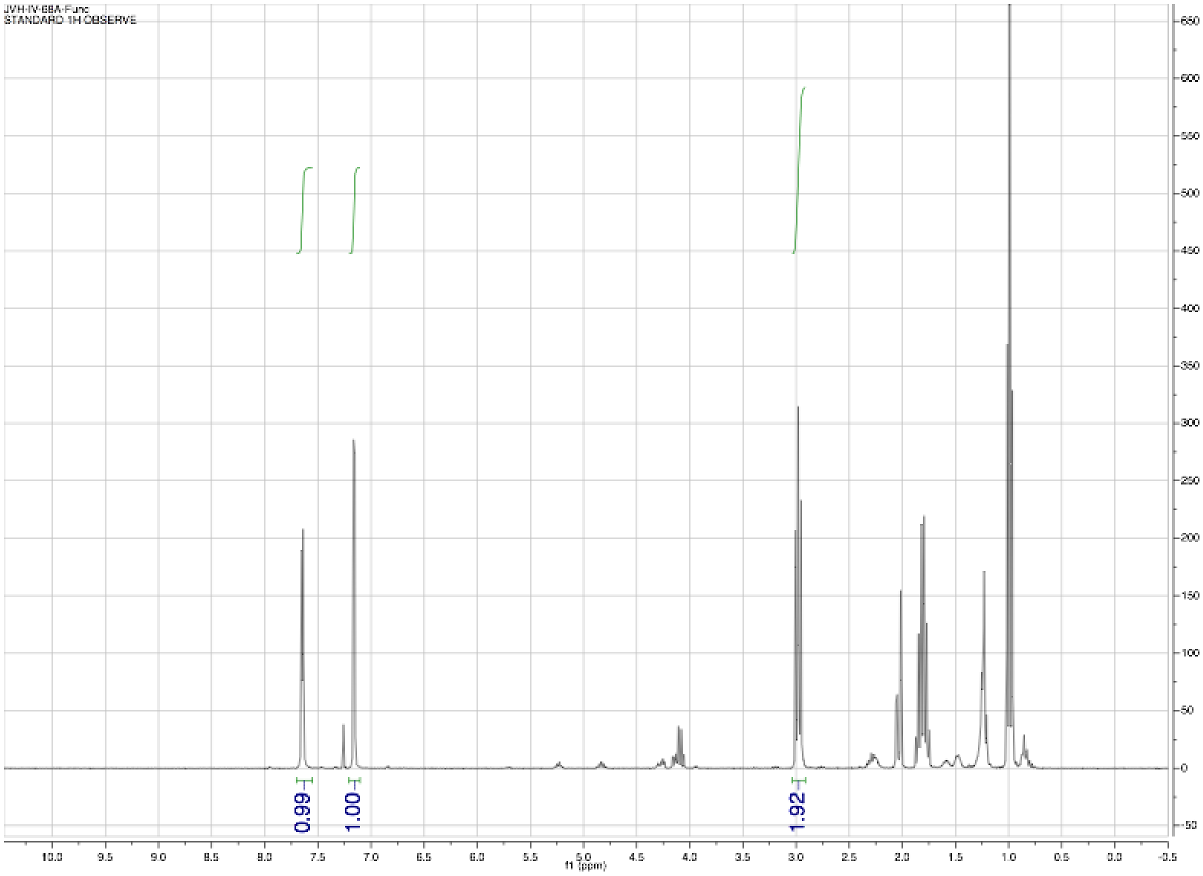

Figure S114. 2-propythiazole isotope exchange catalyzed by Sc(III)

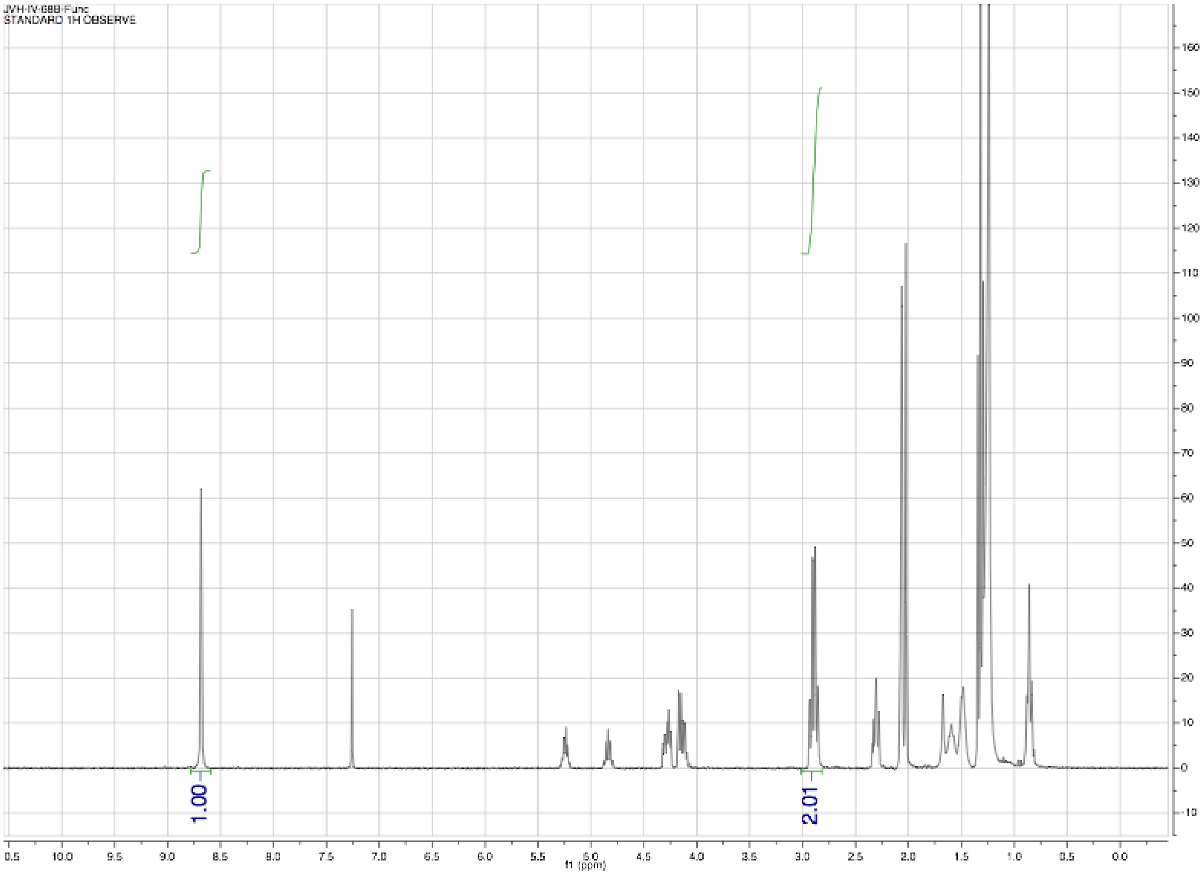

Figure S115. 6-chloro-5-fluoro-4-ethylpyrimidine isotope exchange catalyzed by Sc(III)

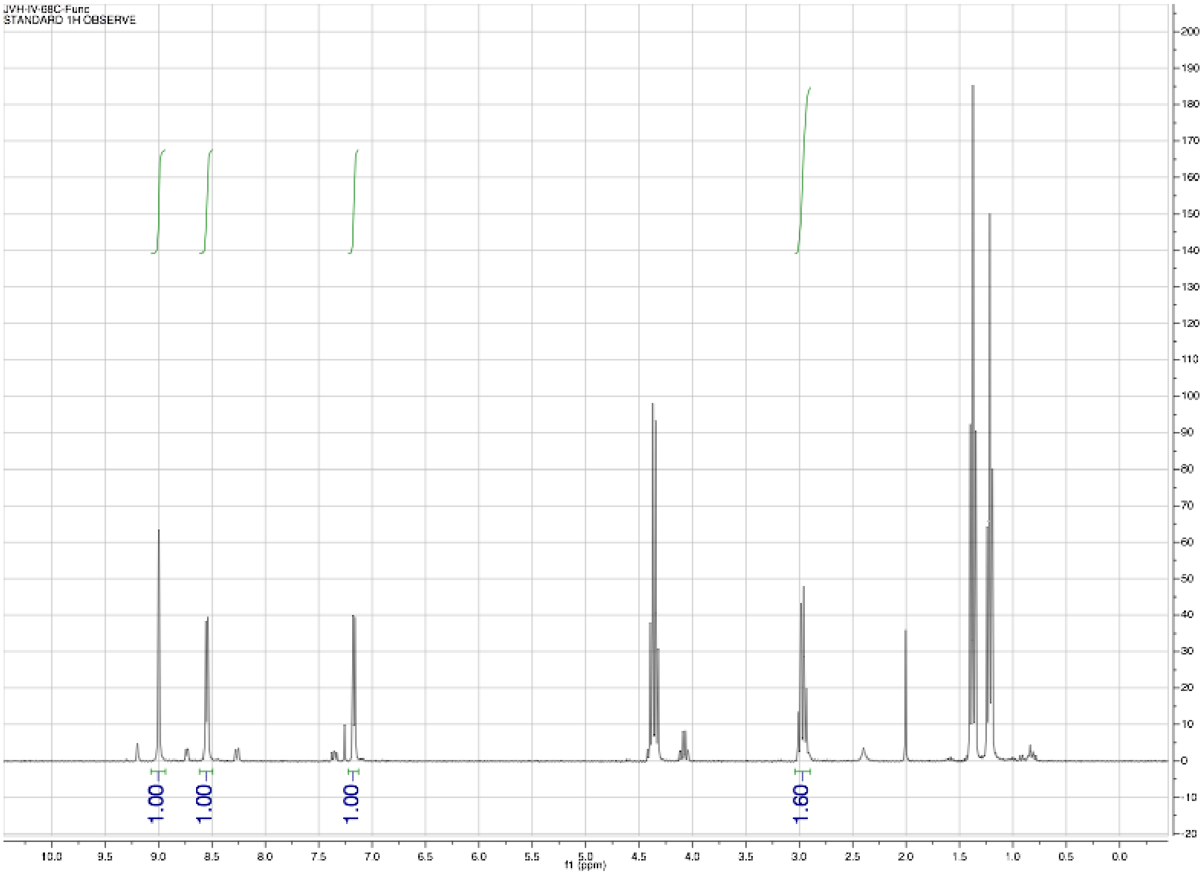

Figure S116. Ethyl 4-ethylnicotinate isotope exchange catalyzed by Sc(III)

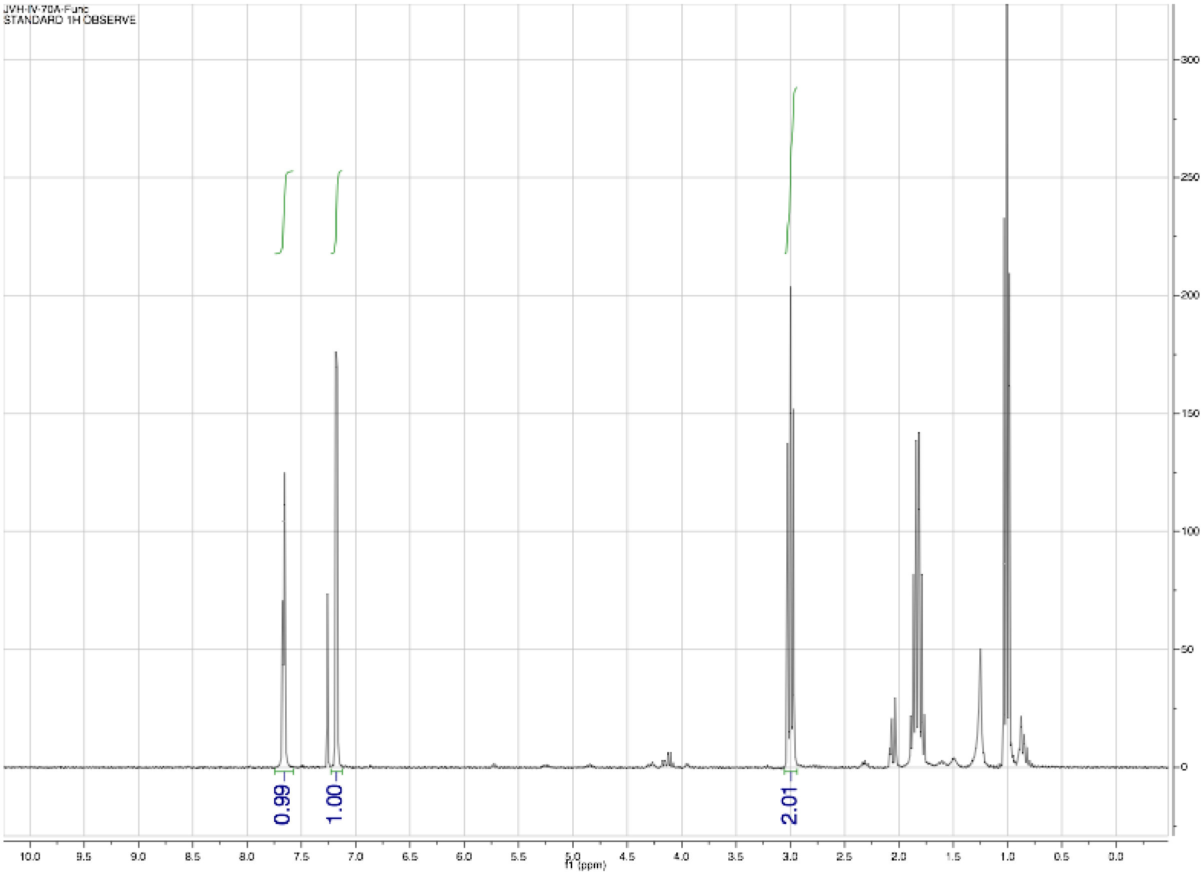

Figure S117. 2-Propylthiazole isotope exchange catalyzed by Cu(II)

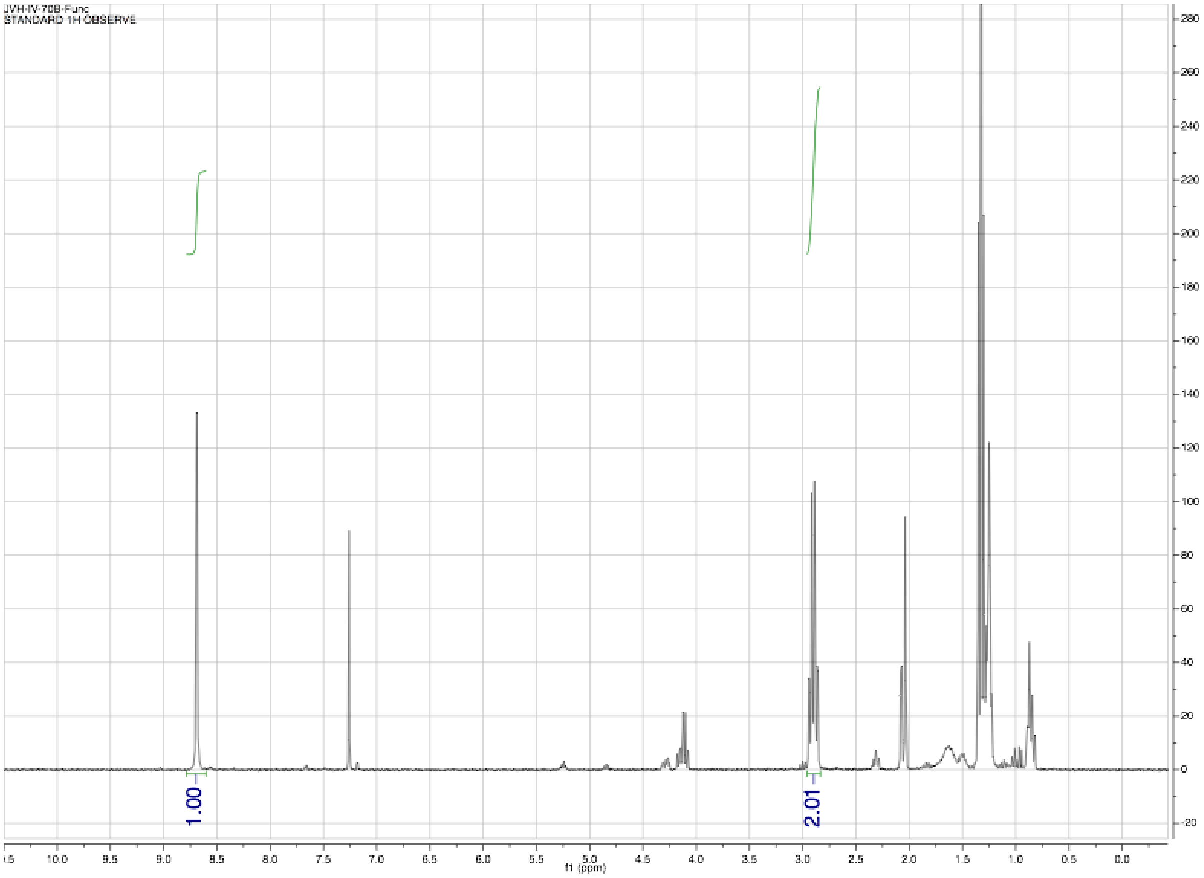

Figure S118. 6-chloro-5-fluoro-4-ethylpyrimdine isotope exchange catalyzed by Cu(II)

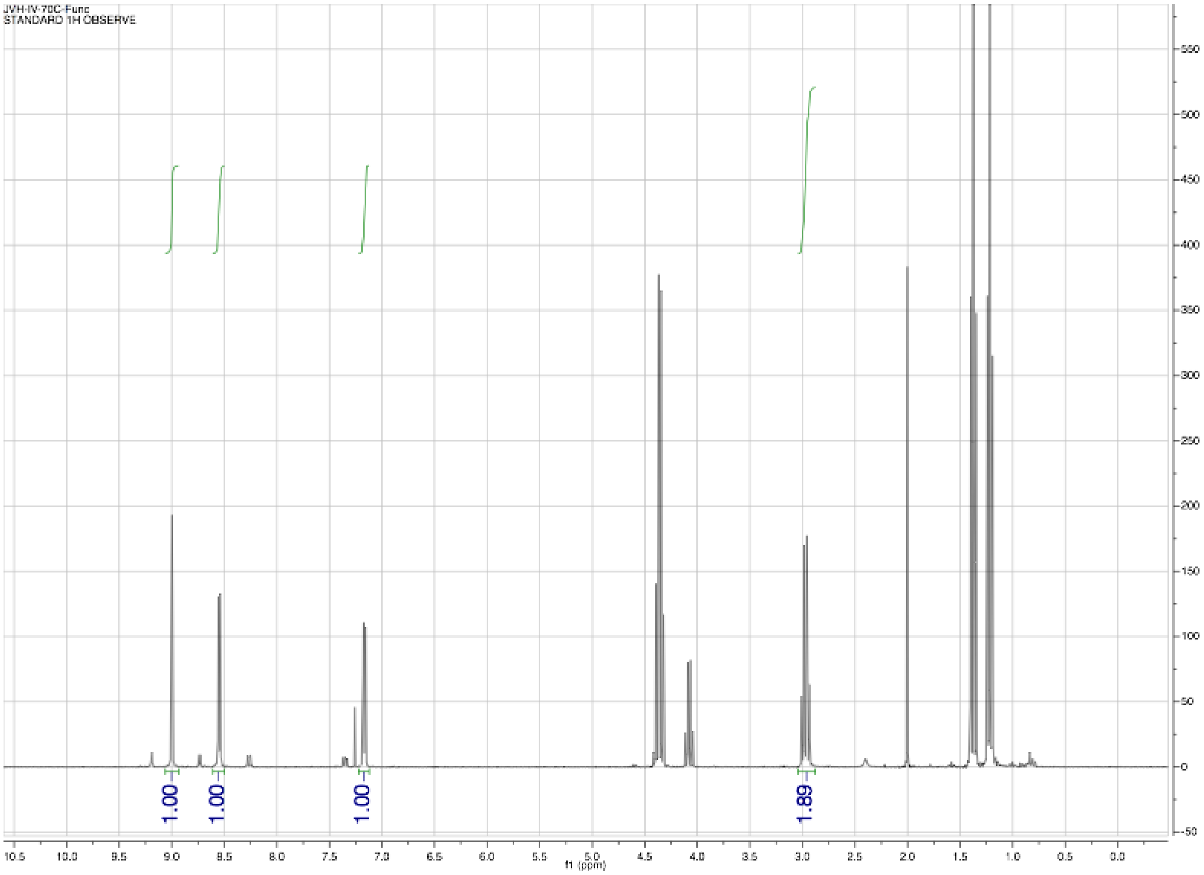

Figure S119. Ethyl 4-ethylnicotinate isotope exchange catalyzed by Cu(II)

6. Kinetic measurements, isotope effects, and supporting experiments

The effect of diethyl hydrazinedicarboxylate (H_2DEAD) was assessed using the following experiment: In an oven-dried 100 mL round bottomed flask equipped with a septum, a stock solution was prepared by first charging 1,3,5-tert-butylbenzene internal standard (247 mg, 0.1 equiv.) and copper(II) triflate (362 mg, 0.1 equiv.). The flask was purged with flowing nitrogen for five minutes, then dry acetonitrile (19 mL) and 6-chloro-5-fluoro-4-ethylpyrimidine (1.25 mL). Simultaneously, six oven-dried 8 mL vials, cooled under flowing nitrogen and capped with Telfon-faced septa were prepared. Three were charged with diethyl hydrazinedicarboxylate (26.4 mg, 10 mol%). 3.0 mL of the prepared stock solution was added to each vial, and the reactions were pre-heated to 80 ºC for five minutes. Then, DEAD (300 µL, 1.25 equiv.) was added to each of the vials, with the additions spaced by precisely 30 seconds. 200 µL aliquots were removed and injected into a quenching solution made from 1 mL ethyl acetate and 1 mL sodium ascorbate (1 M), according to the following schedule (5 min, 10 min, 15 min, 20 min, 30 min, 40 min, 50 min, 60 min, 75 min, 90 min, 120 min, 180 min). Each reaction could be sampled in a single experiment, with the other five vials on the same schedule (+ 30 second, + 1 minute, + 90 seconds, + 2 minutes, and +2 minutes 30 seconds). After all aliquots were quenched, 0.5 mL of the organic layers were removed and filtered through a one inch dry silica plug contained in a Pasteur pipette. 1.5 mL pure ethanol were used to elute the product, which was analyzed by gas chromatography to yield the data shown in Scheme 2 of the manuscript.

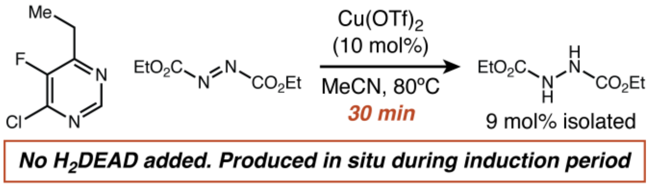

Figure S120. Isolation of H_2DEAD (Figure 5 in manuscript)

The generation of H_2DEAD during the initial reaction induction period was assessed in the following experiment: In a flame-dried 8 mL vial, cooled under flowing nitrogen, copper(II) triflate (18.1 mg, 0.1 equiv.) was added. The vial was purged for a further fives minutes with flowing nitrogen, then dry acetonitrile (1 mL) and 6-chloro-5-fluoro-4-ethylpyrimidine (65 µL, 1 equiv.) were added. The vial was pre-heated to 80 ºC for five minutes, before DEAD was added (100 µL, 1.25 equiv.). After 30 minutes, the vial was cooled to room temperature, loaded directly onto a silica gel column, and eluted with 1:1 hexanes:ethyl acetate to deliver 8 mg of diethyl hydrazinedicarboxylate with a ^{1}H NMR spectrum that matched the known material, as well as matching an authentic sample by TLC co-spot (ceric ammonium molybdate stain).

Kinetic isotope effects for copper(II) and scandium(III) catalyzed reactions were each measured as follows (one identical experiment for each metal): Two stock solutions (one for 4-propylpyridine and one for 4-(propyl-1,1-d_2)pyridine) were prepared as follows: in a 20 mL oven-dried vial, cooled under flowing nitrogen, 1,3,5-tri-tertbutylbenzene (99 mg, 0.1 equiv.), pyridine substrate (520 µL, 1 equiv.), and dry acetonitrile were added (7.0 mL), and finally, DEAD (790 µL, 1.25 equiv.) To each of six oven-dried 8 mL vials was added 10 mol% of the appropriate metal catalyst ($Cu(OTf)_2$ in all 6 in one experiment and $Sc(OTf)_3$ to all 6 in another). In the case of Cu(II) catalyzed reactions, 5 mol% H_2DEAD was also added. The vials were pre-heated to 80 ºC for five minutes, and 2.5 mL of the appropriate stock solution was added (3 for H_2, 3 for D_2), in intervals of 30 seconds. 200 µL aliquots were removed and injected into a quenching solution made from 1 mL ethyl acetate and 1 mL sodium ascorbate (1 M), according to the following schedule (5 min, 10 min, 15 min, 20 min, 30 min, 40 min, 50 min, 60 min, 75 min, 90 min). Each reaction could be sampled in a single experiment, with the other five vials on the same schedule, but adjusted (+ 30 second, + 1 minute, + 90 seconds, + 2 minutes, and +2 minutes 30 seconds). After all aliquots were quenched, 0.5 mL of the organic layers were removed and filtered through a one inch dry silica plug contained in a Pasteur pipette. 1.5 mL pure ethanol were used to elute the product, which was analyzed by gas chromatography to yield the data shown below. Graphical fitting to the apparent initial slop for both H_2 and D_2 substrates yields the reported kinetic isotope effects shown in the manuscript. Both metals display small induction periods, which may reflect the time required for the added stock solution to reach reactive temperature. As such, the line of fit was not forced through the origin.

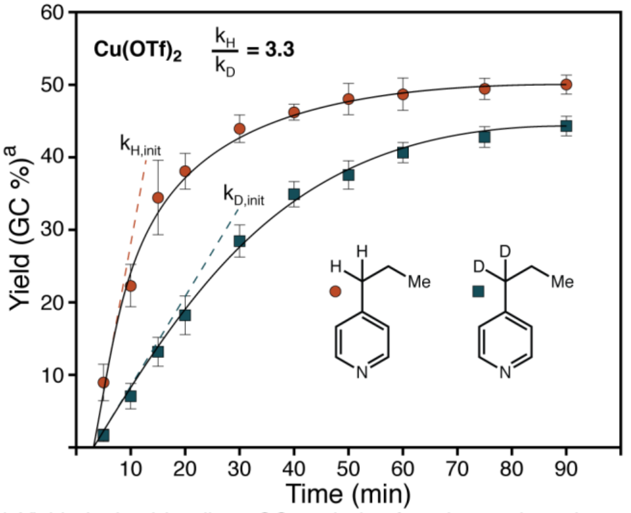

a) Yield obtained by direct GC analysis of crude reaction mixture.
Each reaction was performed in triplicate.

Figure S121. Kinetic isotope effect measurement for copper(II) catalyzed reactions.

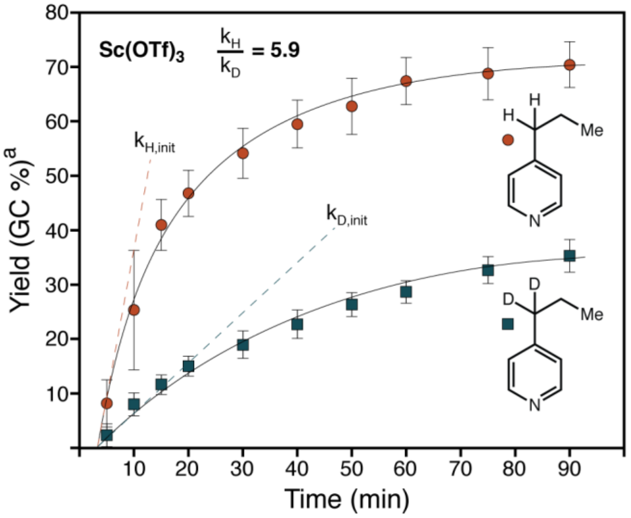

a) Yield obtained by direct GC analysis of crude reaction mixture.
Each reaction was performed in triplicate.

Figure S122. Kinetic isotope effect measurement for scandium(III) catalyzed reactions.

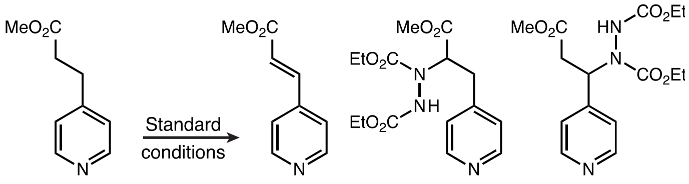

Compound SI-5: Amination was accomplished using the general procedure **A**. Column chromatography over silica gel (20:1 EtOAc:MeOH) afforded 89.9 mg of a colorless oil, with small amount of the presumed regioisomeric product also present, leading to the additional CO_2**Me** singlet peak clearly visible slightly upfield from the CO_2**Me** peak in **SI-5**. Additionally, in the crude NMR for this reaction, signals perfectly matching the known resonances for the unsaturated compound—presumably formed from E_{1CB} elimination via the desired **SI-5**—could be observed.

^{1}H NMR ($CDCl_3$) δ = 8.57-8.43 (m, 2H), 7.37-7.28 (m, 2H), 5.74-5.58 (m, 1H), 4.26-4.05 (m, 4H), 3.13-3.06 (m, 1H), 2.97-2.86 (m, 1H), 1.29-1.13 (m, 6H). ^{13}C NMR ($CDCl_3$) δ = 170.86, 165.88, 157.09, 155.54, 149.85, 149.14, 122.69, 110.57, 63.78, 63.11, 62.31, 57.23, 52.16, 51.55, 35.40, 29.76, 14.50, 13.86. HRMS m/z calcd. for $C_{15}H_{21}N_3O_6$ $[M+H]^+$ 340.1503, found 340.1495

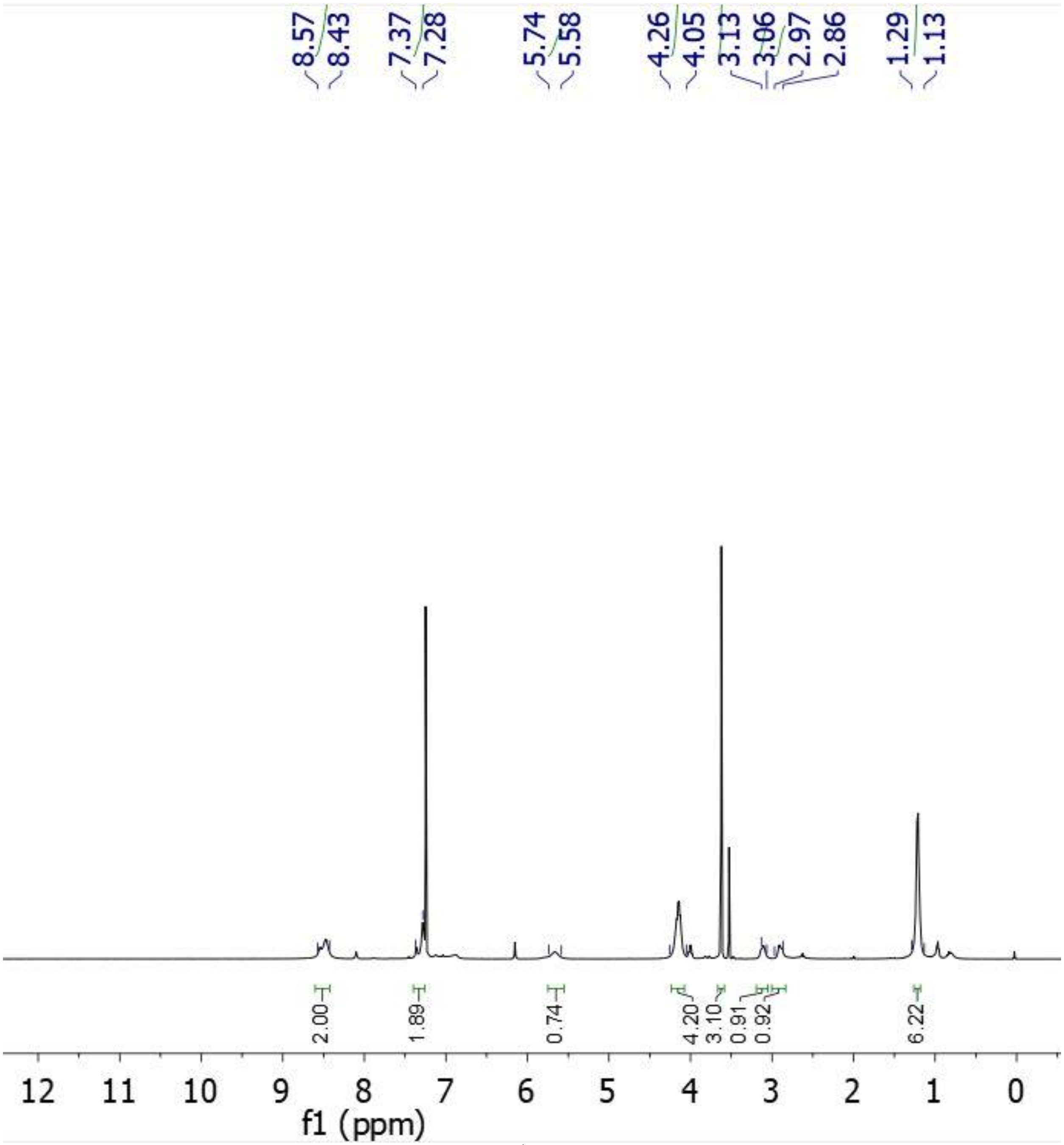

Figure S123: ^{1}H NMR of **SI-5**

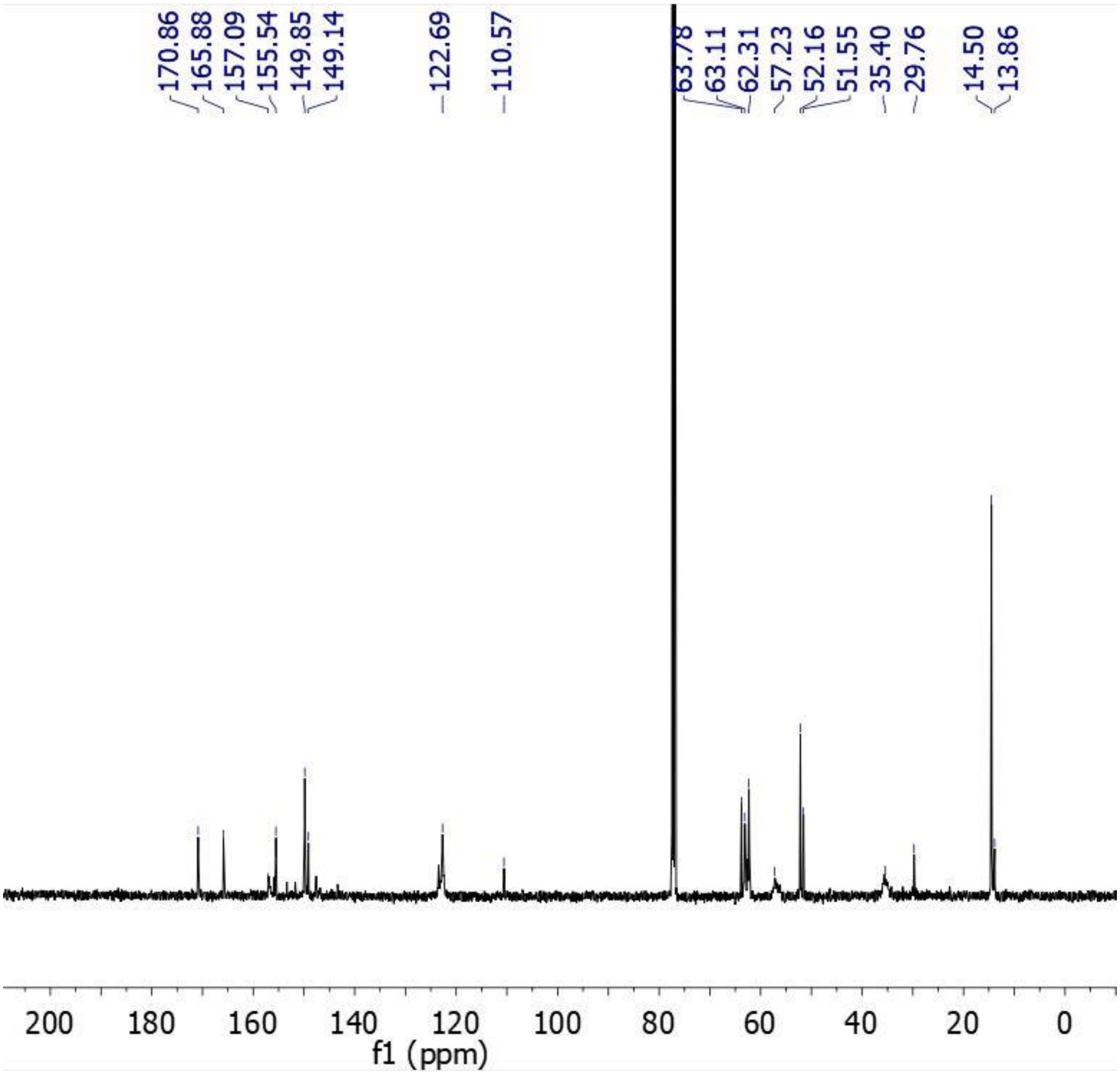

Figure S124: ^{13}C NMR of **SI-5**

7. References

[1] Sperger, C. A.; Wanner, K. T. *Tetrahedron* **2009**, *65*, 5824

[2] Kreighbaum, W. E.; Comer, W. T. Antitumor quinazoline compounds. U.S. Patent 4343940, Aug 10, 1982.

[3] Mauro, M.; Procopio, E. Q.; Sun, Y.; Chien, C.-H.; Donghi, D.; Panigati, M.; Mercandelli, P.; Mussini, P.; D'Alfonso, G.; De Cola, L. *Adv. Funct. Mater.* **2009**, *19*, 2607

[4] Graves, A. P.; Shivakumar, D. M.; Boyce, S. E.; Jacobsen, M. P.; Case, D. A.; Shoichet, B. K. *J. Mol. Bio.* **2008**, *377*, 914

[5] Ishikura, M.; Ohta, T.; Masanao, T. *Chem. Pharm. Bull.* **1985**, *33*, 4755
[6] Samaritani, S.; Signore, G.; Malanga, C.; Menicagli, R. *Tetrahedron* **2005**, *61*, 4475
[7] Felix, R. A.; Chin, H.-L. M.; Woodlard, F. X.; Lee, D. L.; Kanne, D. B. Preparation of 4-cylcoalkyl-5-substituted pyrimidines as crop protection agents. U.S. Patent 5707930, Jan 13, 1998.
[8] Leow, D.; Chen, Y.-H.; Hung, T.-H.; Su, Y.; Lin, Y.-Z. *Eur. J. Org. Chem.* **2014**, *33*, 7347
[9] Bonfianzi, A.; Yano, H.; Del Bello, F.; Farande, A.; Quaglia, W.; Petrelli, R.; Matucci, R.; Nesi, M.; Vistoli, G.; Ferre, S.; Piergentili, A. *J. Med. Chem.* **2014**, *57*, 9065
[10] Howell, J. M.; Feng, K.; Clark, J. R.; Trzepkowski, L. J.; White, M. C. *J. Am. Chem. Soc.* **2015**, *137*, 14590.
[11] Liu, M.; Chen, X.; Chen, T.; Yin, S.-F. *Org. Biomol. Chem.* **2017**, *15*, 2507.
[12] Amaoka, Y.; Kamjio, S.; Hoshikawa, T.; Inoue, M. *J. Org. Chem.* **2012**, *77*, 9959.
